# Toward Expanded Genetic Alphabets: Insights into Functionalized Ds–Px Unnatural Base Pairs

**DOI:** 10.1021/acsphyschemau.6c00082

**Published:** 2026-08-06

**Authors:** Andreu Bermejo, Josep Maria Bofill, Jordi Poater

**Affiliations:** † Departament de Química Inorgànica i Orgànica & IQTCUB, 16724 Universitat de Barcelona, Martí i Franquès 1-11, 08028 Barcelona, Spain; ‡ ICREA, Passeig Lluís Companys 23, 08010 Barcelona, Spain

**Keywords:** unnatural base pair, DNA, noncovalent interaction, density functional theory, π–π stacking

## Abstract

Hydrophobic unnatural base pairs (UBPs) expand the genetic alphabet but the molecular factors governing their stability remain unclear. Herein, we report a quantum chemical study of the unnatural base pair Ds–Px and its 13 variants in a B-DNA environment, previously synthesized by Hirao and co-workers. Base-pair interactions are weak but favorable and primarily dispersion-driven, unlike hydrogen-bond-dominated Watson–Crick pairs. Selected modifications, such as incorporation of an additional nitrogen in the fused ring, modestly enhance electrostatic complementarity. In contrast, π–π stacking interactions are significantly stronger and exceed those of natural stacked pairs. These UBPs present an optimal twist angle that matches the canonical 36° of B-DNA, confirming geometric compatibility. Solvation reduces both pairing and stacking energies but reaches a plateau at high dielectric constants. Overall, these findings demonstrate that Ds–Px-type UBPs are stabilized mainly by dispersion-driven stacking and provide molecular insights for future design of expanded genetic alphabets.

## Graphical abstract

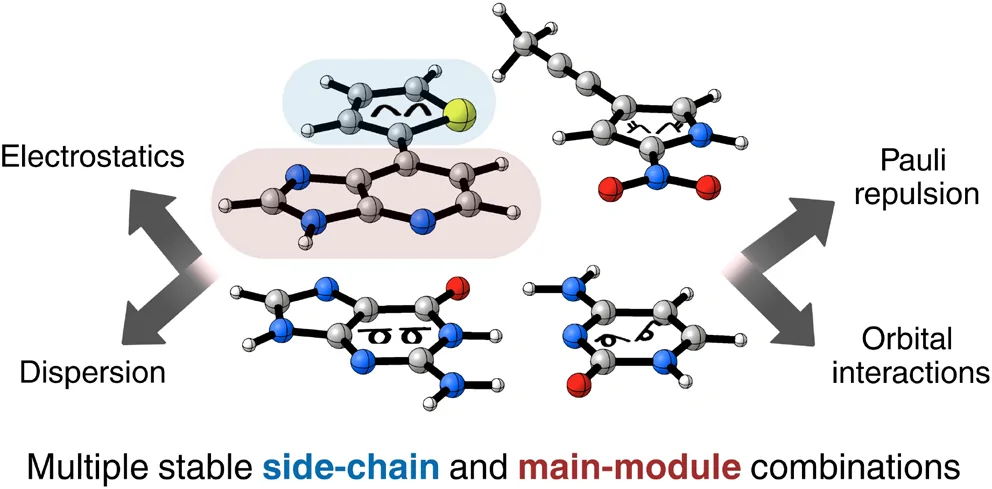

## Introduction

The expansion of the genetic alphabet beyond the canonical nucleobases adenine (A), thymine (T), guanine (G), and cytosine (C) represents a major objective in chemical biology, with the aim of increasing the structural and functional diversity of nucleic acids.^1,2^ Over the past decades, numerous unnatural base pairs (UBPs) have been developed following distinct design strategies, including hydrogen-bonded,^3–6^ metal-mediated,^7,8^ and non-hydrogen-bonded systems.^9,10^ Among these, non-hydrogen-bonded UBPs—whose stability and selectivity rely predominantly on π–π stacking interactions—have attracted particular attention. Notably, in a recent work, we have proven that their pairing selectivity can be governed by intrinsic stacking preferences even in the absence of polymerases,^11^ highlighting the central role of hydrophobic and shape-complementary interactions in these systems. In particular, we successfully reproduced the experimentally observed nucleotide incorporation selectivity of Hirao’s unnatural base pairs,^10^ particularly Ds–Px, even without explicitly modeling the polymerase. The results showed that selectivity arises from stacking interactions within the DNA helix, with electrostatic and dispersion forces playing key roles.^11^ At this point, it must be mentioned that Ds–Px is considered a hydrophobic base pair because its stability and selectivity arise primarily from shape complementarity and dispersion-driven π–π interactions rather than hydrogen bonding, opposite to Watson–Crick base pairs.

The interest in non-hydrogen-bonded UBPs has intensified in the context of DNA aptamers. Aptamers are short single-stranded DNA molecules capable of binding specific molecular targets, including small molecules, proteins, and whole cells, with high selectivity.^12^ Compared with protein-based antibodies, DNA aptamers are hydrophilic and highly water-soluble, avoiding issues related to aggregation and nonspecific adsorption.^13^ However, this intrinsic hydrophilicity also limits hydrophobic interactions with target molecules, often resulting in insufficient binding affinity for practical applications. Conventional aptamers composed solely of natural nucleobases frequently lack the chemical diversity required to establish strong and specific contacts, particularly when hydrophobic interactions are essential for recognition. To overcome this limitation, different approaches have been explored, including the introduction of hydrophobic substituents onto natural nucleobases.^14,15^

An alternative and conceptually distinct strategy was proposed by Hirao and co-workers through the incorporation of a highly hydrophobic, non-hydrogen-bonded unnatural base, 7-(2-thienyl)­imidazo­[4,5-*b*]­pyridine (Ds).^16^ Ds was originally designed to pair selectively with 2-nitro-4-propynylpyrrole (Px), achieving remarkable fidelity (99.9% per cycle) in polymerase chain reaction amplification.^17^ Beyond replication efficiency, the incorporation of Ds into aptamer libraries was shown to enhance target binding with the studied proteins,^16,18^ underscoring the potential of hydrophobic UBPs to expand the functional landscape of nucleic acids. Nevertheless, whether Ds represents the optimal fifth letter for affinity enhancement remains an open question. Noticeably, the same group has just found this last point in a recently published work in which they have developed an approach for generating high-affinity XenoAptamers from six-letter DNA libraries.^19^ The fifth letter, Ds, enhances the structural diversity of DNA, while the sixth letter, Px, enables direct interactions with hydrophobic regions on target proteins. For this latter role, the UBP pyrrole-2-carboxaldehyde (Pa) has also been proposed.^20^

With the aim to prove whether Ds is the best option as a fifth letter, a systematic structural exploration of Ds was undertaken by Hirao and co-workers.^12^ The scaffold was conceptually divided into two modular components: a fused heteroaromatic main core and a heteroaryl side chain (Figure 1). Structural variations of the main module were generated by modifying the fused ring system, including alterations of the six-membered ring (introduction of an additional nitrogen to form pyrimidine analogues or replacement of a ring nitrogen with a CH group to generate benzene derivatives) and modification of the five-membered ring (removal of one nitrogen to yield pyrrole-based systems). This strategy produced a series of distinct cores, namely imidazo­[4,5-*b*]­pyridine (D), pyrrolo­[2,3-*b*]­pyridine (Y), purine (P), indole (I), benzo­[*d*]­imidazole (B), and pyrrolo­[2,3-*d*]­pyrimidine (E). In parallel, multiple side-chain variants were considered, including thienyl (s), furanyl (o), imidazolyl (i), thiazolyl (t), pyridazinyl (p), methylthienyl (sm), and bithiophenyl (ss), generating a broad combinatorial landscape of potential analogues. From the forty-two possible combinations of core and side-chain modules, 13 variants were incorporated into aptamer libraries and experimentally evaluated. Remarkably, substitution of Ds with specific analogues such as 4-(2-thienyl)­benzimidazole (Bs) or 4-(2-thienyl)­pyrrolo­[2,3-*b*]­pyridine (Ys) resulted in enhanced target binding with the tested proteins. These findings suggest that the contribution of unnatural bases to aptamer affinity cannot be attributed solely to increased hydrophobicity, but rather arises from a complex interplay of stacking interactions, shape complementarity, electronic properties, and conformational effects.

**Figure 1:**
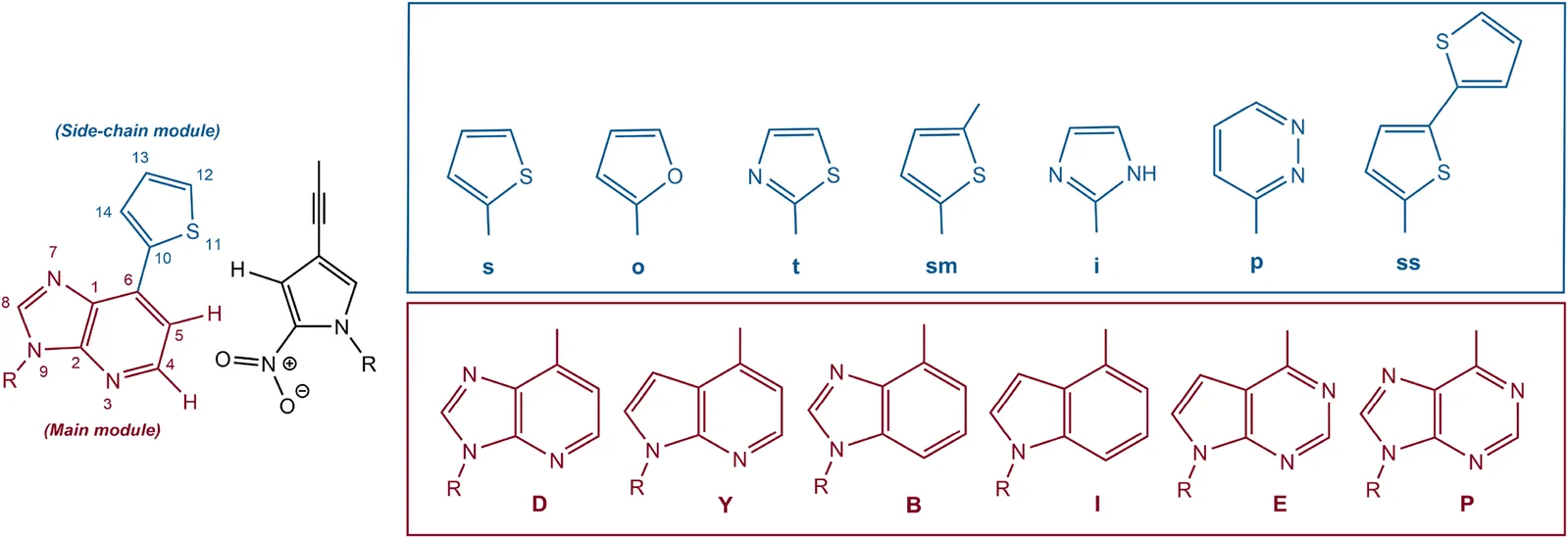
Chemical structure of the unnatural DNA base pair Ds–Px (with R = H, left) and the studied modifications of the side-chain (top right) and main modules (bottom right). Systems taken from Hirao’s experiment.^12^

Together, these results highlight the importance of systematic structural optimization in the development of expanded genetic alphabets and provide a framework for rationally designing next-generation unnatural nucleobases with improved functional performance in aptamer-based applications.^12^ However, despite significant experimental advances, the molecular determinants underlying the behavior of these unnatural systems remain insufficiently understood. Most studies have focused on empirical validation, with limited insight into how specific structural modifications modulate the interactions that govern stability and selectivity. Previous work from our group demonstrated that DNA replication critically depends on π–π stacking interactions and aqueous solvation effects.^21–24^ Building on this foundation, the present study aims to elucidate the structural and energetic factors controlling the stability of the unnatural base pair Ds–Px and its 13 variants within a B-DNA environment. In particular, we systematically analyze base-pairing interactions, π–π stacking contributions, solvation effects, and the influence of the twist angle on stacking efficiency. Noticeably, we are conscious that such UBPs are known to adopt flexible structures; however, the present study focuses on the conformers experimentally characterized in Watson–Crick-like geometries, since these are the relevant structures accommodated within B-DNA.

Notably, template-assisted chemical primer extension has been shown to achieve up to 97% base selectivity in the absence of enzymatic support, indicating that high fidelity can arise from intrinsic nucleobase interactions alone.^25,26^ Accordingly, the DNA model employed in this work operates independently of the polymerase active site, enabling isolation of base–base interactions without interference from amino acid residues. Together, this study provides a comprehensive molecular-level understanding of the structural and energetic principles governing the stability and selectivity of Ds–Px-type unnatural base pairs, offering a rational framework for the design of improved UBPs for expanded genetic alphabets and aptamer applications.

## Results and Discussion

### Base-Pair Interactions

The present study examines the same 13 Ds variants previously evaluated experimentally in aptamer libraries. The calculated base-pair binding energy (Δ*E*) for the original Ds–Px pair is −3.9 kcal mol^–1^, while the variants span a range from −3.6 to −5.7 kcal mol^–1^ (Table 1), indicating weak yet energetically favorable interactions. In contrast, canonical A–T and G–C base pairs exhibit significantly stronger binding energies (−16.5 and –30.3 kcal mol^–1^, respectively), reflecting stabilization by two and three hydrogen bonds.

**Table 1:** Base-Pair Binding Energies (Δ*E*, kcal mol^–1^) Obtained for the Ds–Px Unnatural Base Pair and Its Variants in the Gas Phase^a^

UBP	Δ*E*	UBP	Δ*E*
Ds–Px	–3.9	Dss–Px	–4.1
Bs–Px	–4.2	Dt–Px	–3.8
Is–Px	–4.0	Yo–Px	–4.6
Ys–Px	–3.6	Ps–Px	–5.7
Yi–Px	–3.7	Es–Px	–5.6
Do–Px	–4.9	Ysm–Px	–4.4
Dp–Px	–5.2	Yss–Px	–3.8

a Computed at ZORA-BLYP-D3­(BJ)/TZ2P level in vacuo, with geometries constrained in *C*_*s*_ symmetry. Watson–Crick base pair binding energies amount to –16.5 and –30.3 kcal mol^–1^ for A–T and G–C, respectively.

To assess geometric compatibility with the DNA duplex, the distance between the glycosidic hydrogens (d_H–H_) was analyzed. The computed values range from 10.7 to 11.6 Å (Table S1), slightly longer than those observed for natural Watson–Crick pairs (10.7–11.0 Å), but still within a range compatible with proper incorporation and replication.^2^ Regarding the above lower stability of Ds–Px, recent structural studies by Sawada and co-workers using cryo-EM show that Ds-containing aptamers adopt a range of conformations, with Ds bases frequently stacking against neighboring natural bases in multiple orientations.^27^ These observations suggest that Ds contributes significantly to the structural diversity of aptamers. In this context, the comparatively lower stability of Ds-containing base pairs, relative to natural pairs, may facilitate conformational flexibility.

To gain deeper insight into the origin of the base-pair interaction energies, we analyzed the specific contacts present in the original Ds–Px pair. Three main intermolecular interactions contribute to the overall stabilization (Δ*E* = −3.9 kcal mol^–1^): S···H–C, C–H···H–C, and C–H···O, with respective distances of 2.79, 2.19, and 2.79 Å (Figure 2). Among these, the C–H···O interaction is the only clearly electrostatically favorable contribution, involving the positively polarized hydrogen atom of the pyridine ring in Ds and the partially negatively charged oxygen atom of the nitro group in Px (+59 and –205 milli-e, respectively). The S···H–C contact can be viewed as a weak chalcogen–hydrogen interaction, arising from a combination of dispersion and electrostatic effects associated with the polarizable sulfur atom. The C–H···H–C contact, despite involving two formally nonpolar groups, is not purely repulsive. At the short intermolecular distances found in the Ds–Px pair, attractive London dispersion forces between the electron clouds of the two fragments become significant and can outweigh the weak exchange repulsion. Such dispersion-driven contacts are common in hydrocarbon-rich and aromatic systems and constitute an important component of the hydrophobic effect. Consequently, the stabilization of the Ds–Px pair originates largely from the cumulative effect of multiple weak dispersion-dominated interactions, supplemented by the more directional and electrostatically favorable C–H···O contact.

**Figure 2:**
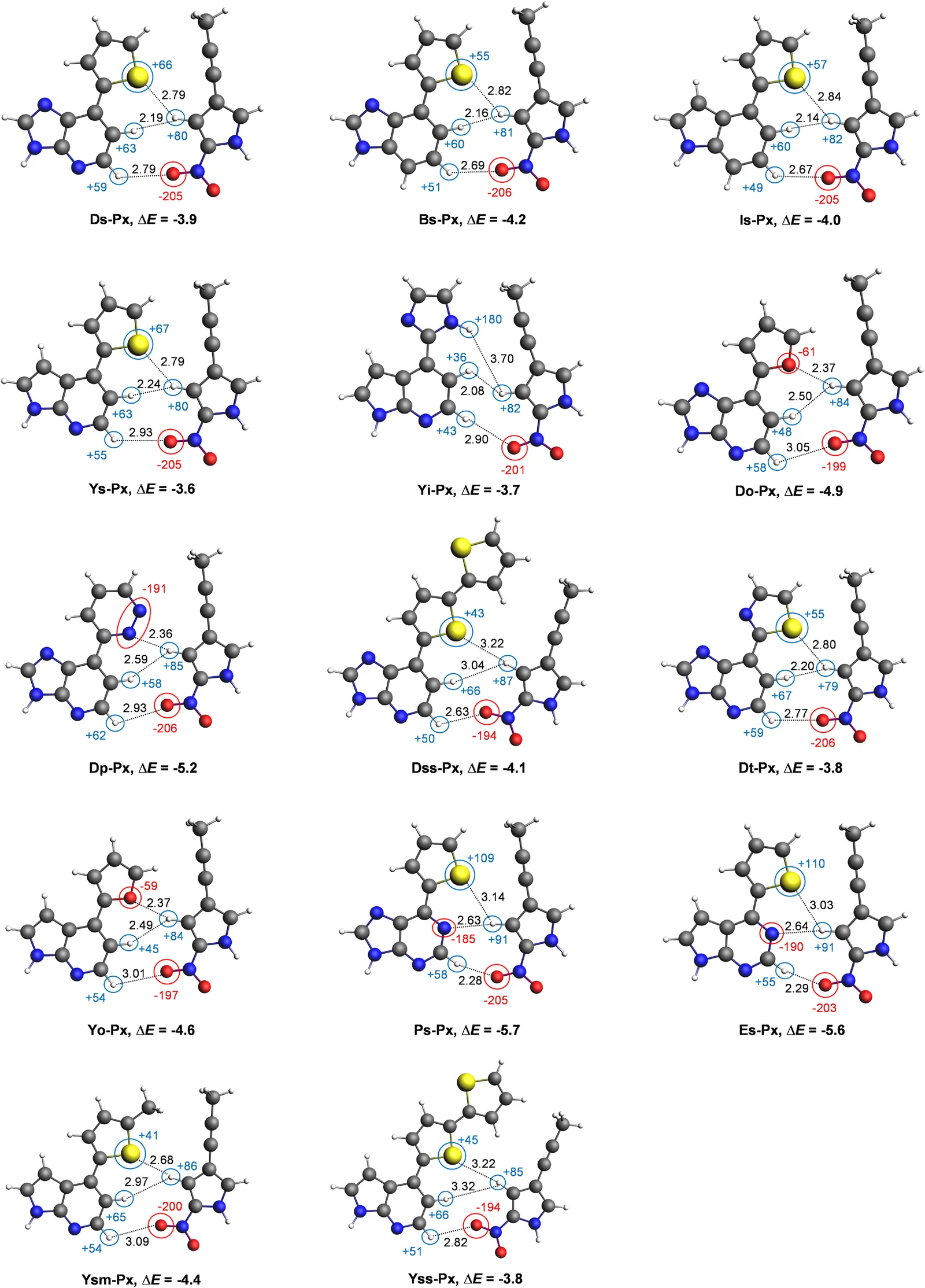
Optimized structures of the unnatural DNA base pair Ds–Px and its variants in the gas phase. Key interatomic distances (Å) are shown along with some of the Voronoi deformation density (VDD) charges (in milli-e).

Among the modified systems, Ps–Px and Es–Px exhibit the strongest binding energies (−5.7 and –5.6 kcal mol^–1^, respectively). In both cases, conversion of the pyridine ring into a pyrimidine introduces an additional nitrogen at position 5, analogous to adenine’s base-pairing interface,^12^ enabling a favorable N···H–C interaction with the α-proton adjacent to the nitro group of Px (2.6 Å, Figure 2). This interaction increases the positive character of the α-proton and shortens the favorable C–H···O contact (from 2.79 Å in Ds–Px to 2.28–2.29 Å). A similar effect is observed for Dp–Px (−5.2 kcal mol^–1^), where a pyridazine side chain forms an N···H–C interaction (2.36 Å), although a slight vertical displacement of Px elongates the C–H···O distance to 2.93 Å.

Replacement of the thiophene ring by furan in Do–Px (−4.9 kcal mol^–1^) and Yo–Px (−4.6 kcal mol^–1^) also enables a favorable O···H–C interaction (2.37 Å), accompanied by a modest increase in the α-proton charge (Figure 2). However, a similar displacement of Px lengthens the C–H···O contact (≈3.0 Å). Introduction of a methyl substituent (Ysm–Px, – 4.4 kcal mol^–1^) slightly enhances stabilization, likely via increased dispersion contributions, although steric effects elongate the C–H···O distance to 3.09 Å. In contrast, incorporation of a bulkier bithiophene (Dss–Px, –4.1 kcal mol^–1^; Yss–Px, –3.8 kcal mol^–1^) has little effect on overall binding energy but induces rotation of Px, increasing the S···H–C distance to 3.22 Å. For these systems, it is worth mentioning the effect of constrained planarity on thiophene. The imidazole-containing Yi–Px (−3.7 kcal mol^–1^) similarly maintains comparable binding energy while elongating the C–H···O distance (2.90 Å). The remaining variants, involving N ↔ C substitutions without direct contact to Px, show interaction energies and geometries close to the reference Ds–Px pair.

To clarify the nature of these interactions, an energy decomposition analysis (EDA) was performed for Ds–Px and compared with canonical Watson–Crick pairs (Table S5). In natural base pairs, stabilization arises primarily from electrostatic and orbital interactions (e.g., in G–C: 56% electrostatic, 36% orbital, 8% dispersion).^22–24,28,29^ In contrast, the modest binding energy of Ds–Px (−3.9 kcal mol^–1^) is dominated by dispersion (60%), with smaller electrostatic (18%) and orbital (21%) contributions. These trends are supported by NCI analyses (Figure S2), which reveal hydrogen-bond-dominated interactions in Watson–Crick pairs and dispersion-driven contacts in Ds–Px (blue vs green isosurfaces, respectively). Additionally, strain energy in Ds–Px is negligible (0.1 kcal mol^–1^), indicating minimal conformational distortion upon pairing.

Because DNA replication occurs in solution, solvation effects were further evaluated in solvents of increasing polarity (chloroform, methanol, and water). We must emphasize that several experimental studies on DNA model systems are routinely also performed in chloroform and related low-polarity solvents, whose dielectric constants are substantially lower than that of water, the reference polar medium.^30–32^ This is why we have taken a range of different polarity solvents. Increasing solvent polarity systematically weakens base-pair binding energies due to stabilization of charge separation, consistent with previous observations for natural and mismatched base pairs (Figure 3 and Tables S2–S4).^21,22,24^ For Ds–Px, solvation also produces a slight elongation of the glycosidic H···H distance d_H–H_ (Figure S4), reflecting subtle structural adjustments in polar environments.

**Figure 3:**
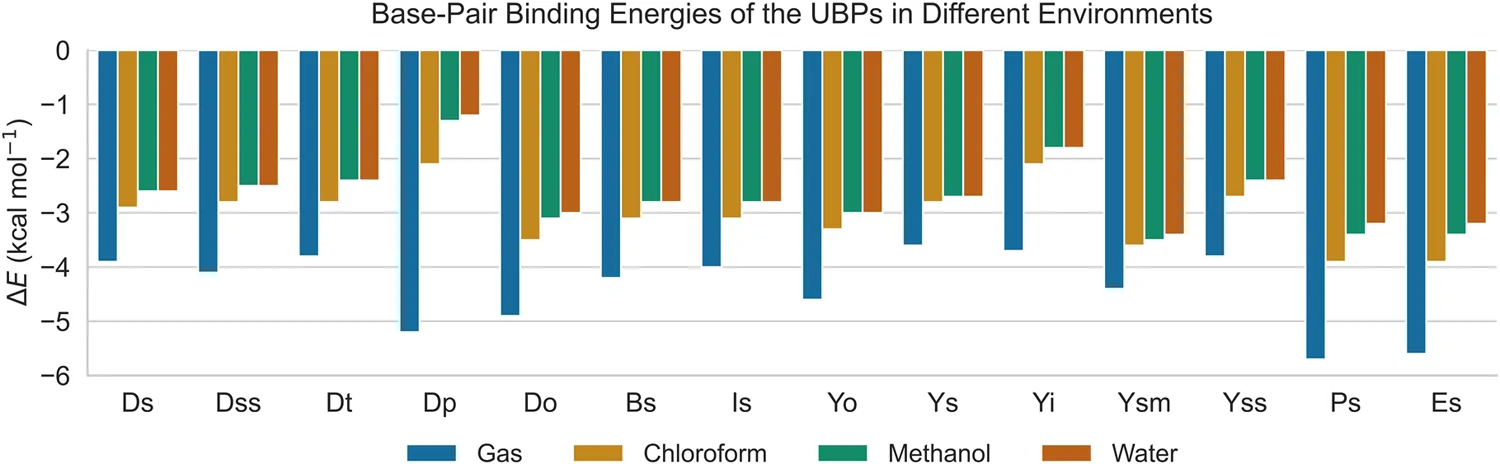
Base-pair binding energies (Δ*E*, kcal mol^–1^) of the unnatural DNA base pair Ds–Px and its variants computed in four different environments: gas phase, chloroform (ε = 4.8), methanol (ε = 32.6), and water (ε = 78.4).

In chloroform, base-pair binding energies range from −2.1 to −3.9 kcal mol^–1^, with the reference Ds–Px pair decreasing to −2.9 kcal mol^–1^—approximately 1 kcal mol^–1^ weaker than in the gas phase. This reduction reflects the greater stabilization of the isolated bases relative to the paired system due to their larger solvent-exposed surface area, consistent with the computed solvation energies (Tables S6–S7). Ps–Px and Es–Px remain the strongest pairs (−3.9 kcal mol^–1^), whereas Dp–Px becomes the weakest (−2.1 kcal mol^–1^). VDD charge analysis shows partial shielding of the favorable electrostatic interaction at the α-proton of Px: its charge decreases from +91 milli-e in vacuo to +85 and +84 milli-e in Ps–Px and Es–Px, respectively. In Dp–Px, the reduction is more pronounced (+75 milli-e), rationalizing its lower stability. The remaining variants follow trends similar to those observed in the gas phase. In methanol, binding energies further decrease (−1.3 to –3.5 kcal mol^–1^), with Ds–Px reaching −2.6 kcal mol^–1^. Results in water are nearly identical to those in methanol (maximum difference of 0.2 kcal mol^–1^), indicating that beyond a certain dielectric constant, solvation effects become essentially constant.

To assess how structural modifications influence solvation, the main and side-chain modules of Ds were analyzed separately. Replacing the pyridine nitrogen with CH (Bs) or removing a nitrogen from the imidazole ring (Ys) reduces solvation energy due to diminished charge accumulation, whereas introducing an additional nitrogen to form a pyrimidine (Ps) increases solvation energy (Figure S3). Side-chain substitutions with bithiophene (Dss) or furan (Do) have little impact, while incorporation of more heteroatom-rich rings such as thiazole (Dt), pyridazine (Dp), or imidazole (Yi) enhances solvation energy owing to increased polarity.

Although the implicit model does not capture specific hydrogen bonds with the solvent, UBPs consistently exhibit smaller solvation energies than natural base pairs, underscoring their hydrophobic character (Tables S6–S7). For instance, in water Ds–Px shows a solvation energy of −17.9 kcal mol^–1^, compared to −24.2 kcal mol^–1^ for G–C; the difference is even more pronounced for the isolated bases. Overall, these results demonstrate that, unlike in canonical Watson–Crick pairs, direct base-pair interactions contribute only modestly to the stability of Ds–Px-type systems within the double helix, and subtle structural changes can slightly strengthen them.

### π–π Stacking Interactions

Having established the intrinsic base-pair interactions of this series of functionalized Ds–Px systems, we next examined their stacked configurations to evaluate π–π stacking contributions. Notably, we have recently demonstrated that π–π stacking interactions can govern the selectivity of unnatural DNA base pairs even in the absence of polymerase.^11^ To isolate these effects, a simplified model was constructed in which the sugar–phosphate backbone was removed and replaced by hydrogen atoms at the glycosidic positions. A *C*_s_ symmetry constraint was applied to reproduce the nearly planar arrangement characteristic of B-DNA, and the interbase-pair distance was fixed at 3.4 Å, corresponding to the average stacking distance in B-DNA (Figure 4). This setup enables direct assessment of stacking interactions independent of enzymatic or backbone influences.^21,22^

**Figure 4:**
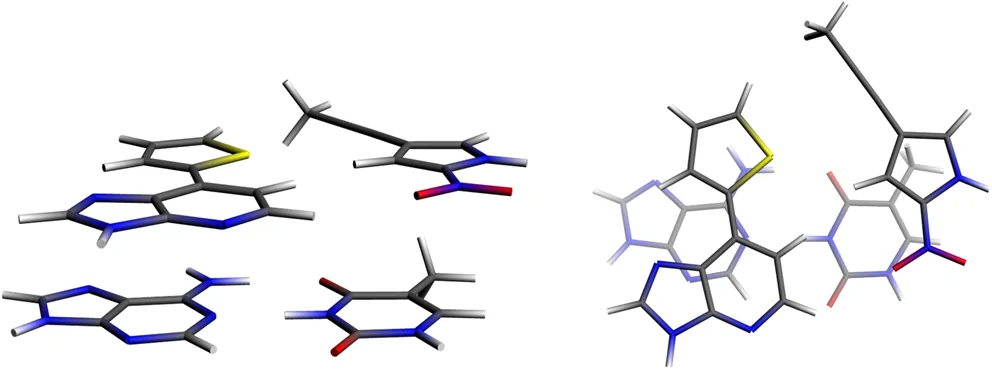
Preparation of the stacked Ds–Px//A–T system. Vertical separation of the base pairs by 3.4 Å (left) followed by a 36° rotation of the upper pair (right).

Although the average twist angle in natural B-DNA is ∼36°,^21,33^ no prior study has evaluated whether this geometry is also optimal for the unnatural Ds–Px pair. To address this question, π–π stacking interactions were calculated for the stacked systems Ds–Px//A–T and Ds–Px//G–C while varying the twist angle from 0° to 75° in 5° increments, without allowing relaxation of the base pairs. Both systems exhibit maximum stabilization within a twist-angle range of approximately 25–40° (Figure 5), with energy differences below 0.5 kcal mol^–1^ across this interval. This optimum closely matches the experimentally observed 36° twist of canonical Watson–Crick base pairs, indicating that Ds–Px is geometrically compatible with the native helical architecture and does not require significant deviation from the natural B-DNA twist to achieve favorable stacking.

**Figure 5:**
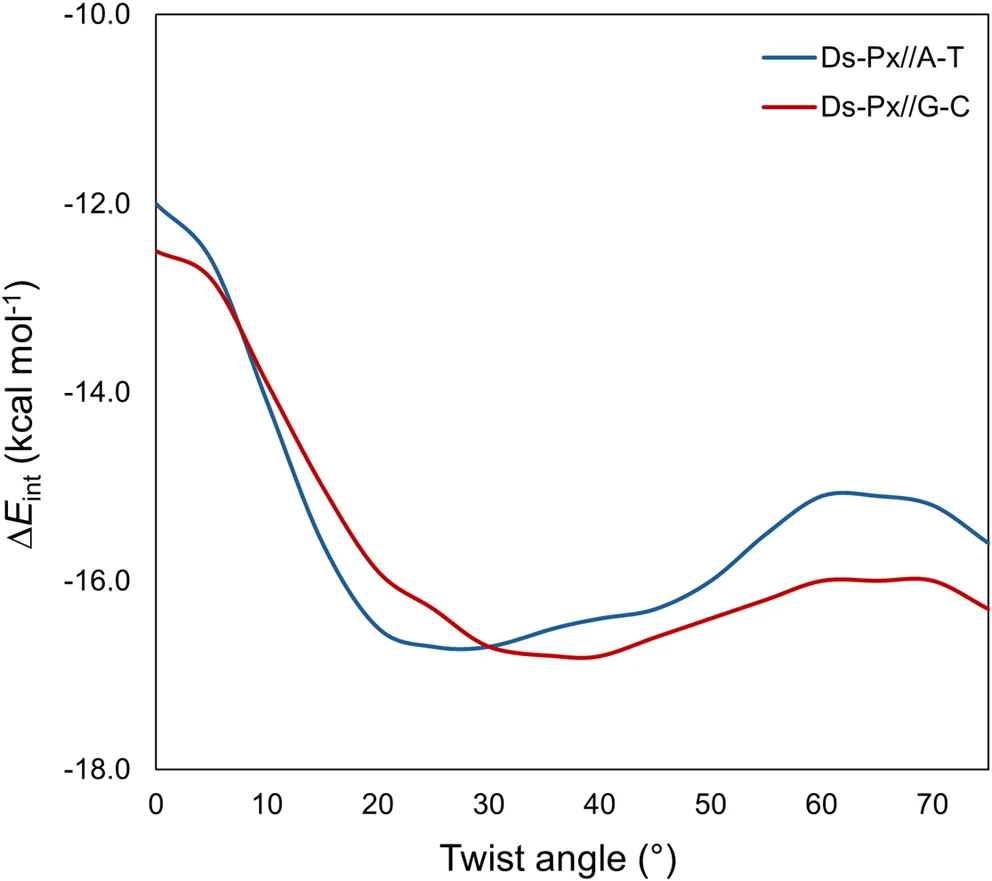
π–π stacking interaction energies of the Ds–Px//A–T and Ds–Px//G–C systems as a function of the twist angle (0–75°) in the gas phase.

Although Ds–Px//A–T and Ds–Px//G–C exhibit comparable overall π–π stacking energies, energy decomposition analysis (Tables S8–S9) reveals notable differences in their individual components (Figure 6). Ds–Px stacked with A–T shows a stronger electrostatic contribution than with G–C. Orbital interactions—primarily associated with π-system polarization—decrease as the twist angle increases, reflecting the growing separation between aromatic rings and the reduced mutual polarization; however, their total contribution remains similar in both systems (differences <0.5 kcal mol^–1^). Dispersion constitutes the dominant stabilizing term in both stacked systems and progressively weakens with increasing twist angle as the intermolecular distance grows. Since dispersion arises from interactions between instantaneous dipoles and decays with distance (*R* ^–6^),^34^ its attenuation upon ring separation is expected. NCI analyses (Figure S5) support this trend, showing a reduction in the characteristic dispersion-driven green surfaces at a 36° twist. Dispersion is slightly more stabilizing in Ds–Px//A–T than in Ds–Px//G–C.

**Figure 6:**
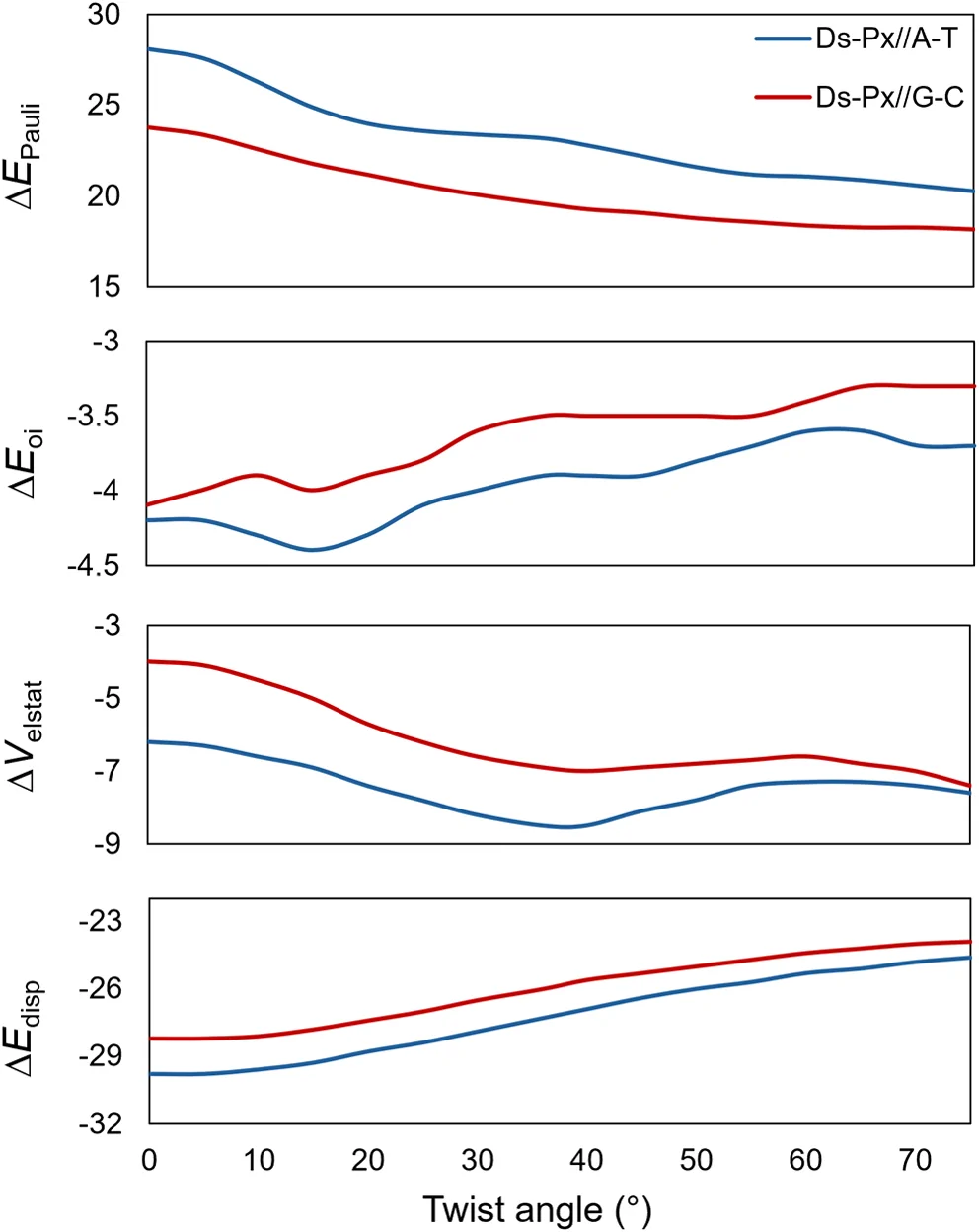
Energy Decomposition Analysis (EDA) of the π–π stacking interaction energies in the Ds–Px//G–C and Ds−Px//A−T systems as a function of the twist-angle (0–75°) in the gas phase. All values are given in kcal mol^–1^.

However, this enhanced stabilization is counterbalanced by greater Pauli repulsion in Ds–Px//A–T. This destabilizing term, originating from interactions between occupied orbitals, reflects steric effects. The increased repulsion can be attributed to the methyl group of thymine, which protrudes from the base plane and interacts unfavorably with the adjacent stacked pair. In both systems, Pauli repulsion decreases with increasing twist angle as the larger separation between aromatic rings reduces occupied–occupied orbital overlap.

Having established 36° as a representative twist angle for stacked geometries, π–π stacking interaction energies of the Ds–Px variants with canonical base pairs were evaluated at this angle to enable direct comparison with natural systems. In the gas phase, stacking energies with A–T range from −14.9 to −16.8 kcal mol^–1^, and from −15.6 to −19.0 kcal mol^–1^ with G–C (Table S10). For reference, natural stacked pairs under identical conditions exhibit weaker interactions: −14.1 kcal mol^–1^ (A–T//A–T), –12.5 kcal mol^–1^ (G–C//G–C), and –13.3 kcal mol^–1^ (A–T//G–C). Notably, these stacking energies are substantially stronger than the corresponding base-pair binding energies, highlighting the dominant role of π–π stacking in stabilizing Ds–Px-type systems within a B-DNA environment.

To rationalize the subtle differences among variants, an energy decomposition analysis was performed on selected systems using Ds–Px as reference. Specifically, Ps–Px and Do–Px—displaying the strongest and weakest stacking interactions with A–T, respectively—and Dp–Px and Dss–Px—showing the strongest and weakest stacking interactions with G–C (Table 2)—were examined in detail.

**Table 2:** Energy Decomposition Analysis (EDA) of the π–π Stacking Interaction Energies for the Unnatural Base Pair Ds–Px and Selected Variants, Stacked with A–T and G–C in the Gas Phase^a^,^b^

system	Δ*E*_int_	Δ*V*_elstat_	Δ*E*_Pauli_	Δ*E*_oi_	Δ*E*_disp_
Ds−Px//A−T	–16.5	–8.5	23.2	–3.9	–27.3
Ps−Px//A−T	–16.9	–8.5	23.4	–4.2	–27.6
Do−Px//A−T	–14.9	–7.8	21.6	–3.7	–25.0
Ds−Px//G−C	–16.8	–6.9	19.6	–3.5	–26.0
Dp−Px//G−C	–19.0	–8.5	17.7	–3.4	–24.8
Dss−Px//G−C	–15.6	–5.5	17.8	–3.3	–24.6

a Computed at ZORA-BLYP-D3­(BJ)/TZ2P level in vacuo. Δ*E*_int_ = Δ*E*_Pauli_ + Δ*E*_elstat_ + Δ*E*_oi_ + Δ*E*_disp_. For comparison, stacking interaction between Watson–Crick base pairs amount to –14.1 (A–T//A–T), –12.5 (G–C//G–C), and –13.3 kcal mol^–1^ (A–T//G–C).

b All values are given in kcal mol^–1^.

For the reference Ds–Px system stacked with A–T (−16.5 kcal mol^–1^), π–π stabilization is dominated by dispersion (−27.3 kcal mol^–1^), followed by electrostatic (−8.5 kcal mol^–1^) and orbital (−3.9 kcal mol^–1^) contributions (Table 2), which collectively counterbalance Pauli repulsion (23.2 kcal mol^–1^). When stacked with G–C (−16.8 kcal mol^–1^), all attractive terms are slightly reduced—dispersion (−26.0 kcal mol^–1^), electrostatics (−6.9 kcal mol^–1^), and orbital interactions (−3.5 kcal mol^–1^)—along with a lower Pauli repulsion (19.6 kcal mol^–1^), resulting in a comparable overall interaction energy. Ps–Px, which shows the strongest stacking with A–T, follows a similar EDA profile to the reference system. In contrast, Do–Px exhibits reduced Pauli repulsion due to the less diffuse orbitals of the furan oxygen, decreasing steric overlap with the opposing base; however, this is offset by weaker dispersion (−25.0 kcal mol^–1^) and electrostatic (−7.8 kcal mol^–1^) contributions. Yo–Px displays nearly identical stacking behavior, consistent with sharing the same furan side chain and differing only by a single N → C substitution in the main module.

For G–C stacking, Dp–Px—showing the strongest interaction—is characterized by reduced Pauli repulsion (17.7 kcal mol^–1^) and enhanced electrostatic stabilization (−8.5 kcal mol^–1^), reflecting improved steric and electrostatic complementarity of the pyridazine ring with G–C (Table 2). Conversely, Dss–Px, despite its enlarged aromatic surface, presents a weaker electrostatic term (−5.5 kcal mol^–1^), leading to the weakest stacking interaction with G–C; its closely related analogue Yss–Px exhibits nearly identical behavior. The remaining variants, involving minor N ↔ C substitutions or methylation, show negligible differences in stacking energies. Overall, the 13 Ds–Px analogues display remarkably similar π–π stacking interactions, indicating that structural modifications in either the main core or side chain have limited impact on helical stability. Dispersion remains the dominant stabilizing factor, with electrostatic and orbital interactions providing secondary contributions.

Finally, as previously done for base-pair binding energies, the effect of solvation on π–π stacking interactions was examined. As expected, increasing solvent polarity leads to a systematic reduction of stacking energies (Figure 7 and Tables S11–S13), consistent with solvent screening of intermolecular interactions.

**Figure 7:**
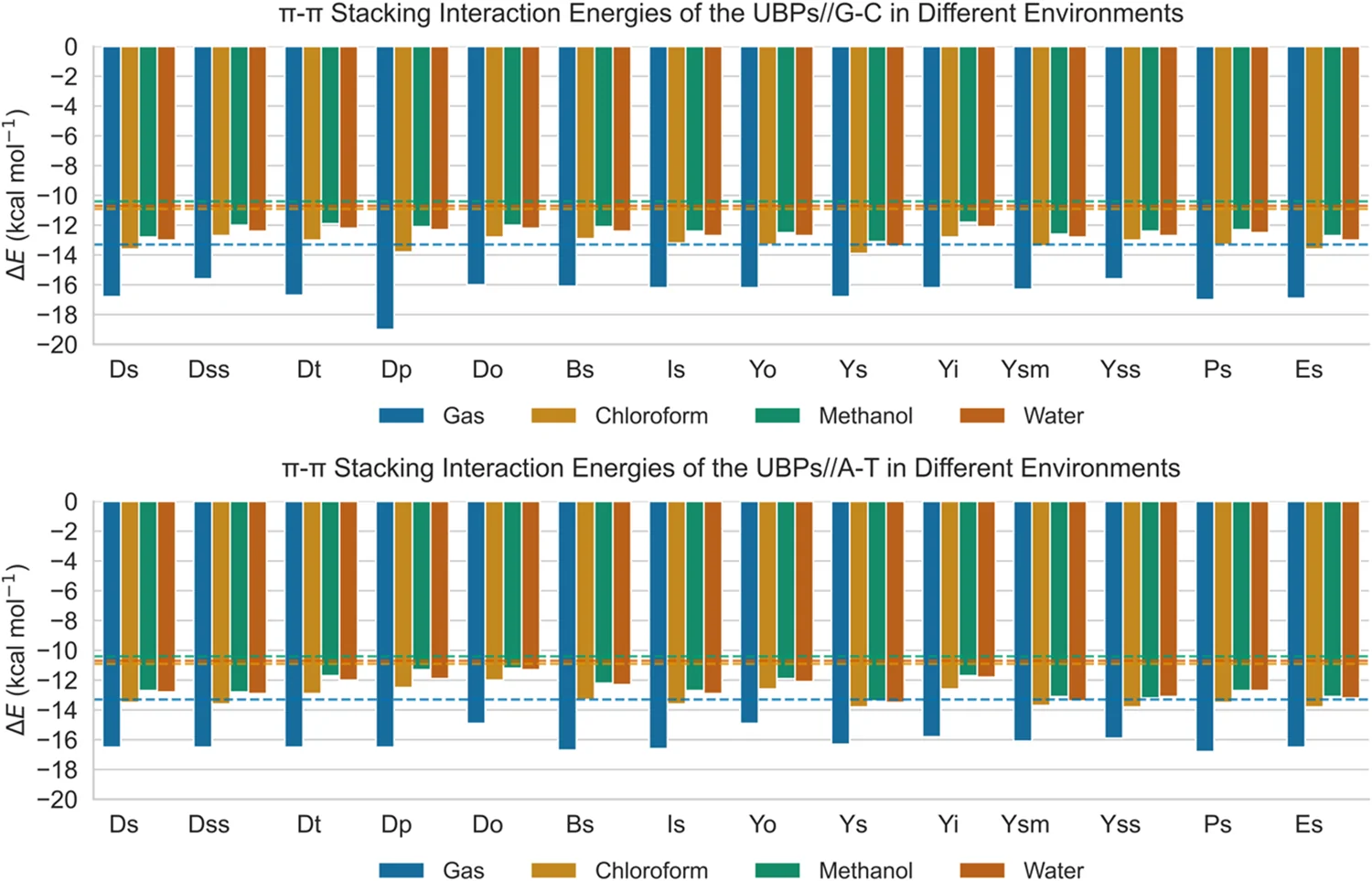
π–π Stacking interaction energies (Δ*E*, kcal mol^–1^) of the unnatural DNA base pair Ds–Px and its variants, stacked with A–T (top) and G–C (bottom), computed in four different environments: gas phase, chloroform (ε = 4.8), methanol (ε = 32.6), and water (ε = 78.4). Dashed horizontal lines indicate the stacking energy of A–T//G–C system in each environment.

In chloroform, π–π stacking interaction energies range from −12.0 to −13.8 kcal mol^–1^ for systems stacked with A–T and from −12.7 to −13.9 kcal mol^–1^ for those stacked with G–C (Figure 7). For the reference Ds–Px pair, stacking energies decrease by approximately 3 kcal mol^–1^ relative to the gas phase, yielding −13.5 kcal mol^–1^ (A–T) and −13.6 kcal mol^–1^ (G–C). Among the A–T-stacked systems, Ys–Px, Es–Px, and Yss–Px exhibit the strongest interactions (−13.8 kcal mol^–1^), closely followed by Ps–Px (−13.5 kcal mol^–1^), which was the strongest in the gas phase. The slightly reduced performance of Ps–Px in solution reflects enhanced stabilization of the isolated Ps–Px dimer, likely due to its higher nitrogen content and increased polarity. Consistent with gas-phase results, Do–Px remains the weakest interactor (−12.0 kcal mol^–1^). For the G–C-stacked systems, Ys–Px shows the strongest interaction (−13.9 kcal mol^–1^), followed by Dp–Px (−13.8 kcal mol^–1^), whereas Dss–Px again exhibits the weakest stacking (−12.7 kcal mol^–1^).

In methanol, stacking energies further decrease, spanning −11.2 to −13.4 kcal mol^–1^ (A–T) and −11.8 to −13.1 kcal mol^–1^ (G–C), while preserving the relative trends observed in chloroform (Figure 7). In water, variations relative to methanol are minimal (<0.5 kcal mol^–1^ for most systems), confirming that once a sufficiently high dielectric constant is reached, further increases have only a minor effect on interaction energies.

Importantly, for all solvents examined, the π–π stacking interactions of the modified Ds–Px systems remain stronger than those of the natural A–T//G–C stacked pair. These results demonstrate that incorporation of Ds–Px-type unnatural base pairs enhances stacking stabilization within the DNA helix, reinforcing the dominant structural role of dispersion-driven π–π interactions in these systems.

## Conclusions

In this work, we carried out a comprehensive quantum chemical investigation of the unnatural DNA base pair Ds–Px and its 13 structural variants within a B-DNA environment. By separately analyzing base-pairing and π–π stacking interactions, we elucidate the molecular factors governing the stability and selectivity of these expanded genetic alphabet systems. Our results demonstrate that π–π stacking is the primary stabilizing force in Ds–Px-type systems. These interactions are strongly dispersion-driven and exceed those of canonical Watson–Crick stacks under comparable conditions. Energy decomposition analyses reveal dispersion as the dominant attractive contribution, while electrostatic and orbital interactions play secondary roles. Moreover, the optimal stacking geometry is found within a twist-angle range of 25–40°, closely matching the ∼36° twist of natural B-DNA and confirming the excellent geometric compatibility of Ds–Px-type pairs with the native double helix.

In contrast, intrinsic base-pairing interactions are relatively weak and fundamentally different from the hydrogen-bonding patterns of natural nucleobases. Although strategic incorporation of heteroatoms or alternative side chains can modestly improve electrostatic complementarity and base-pair stability, these effects remain secondary to stacking. Nevertheless, base-pairing interactions contribute to orientation, selectivity, and replication fidelity. Solvation weakens both stacking and pairing interactions, but the attenuation rapidly reaches a plateau, and Ds–Px variants retain stronger stacking interactions than natural base pairs even in polar media.

Overall, our findings establish that Ds–Px and its derivatives can be accommodated within the DNA double helix without disrupting canonical helical parameters. The robustness of their stacking interactions toward chemical modification suggests promising opportunities for the design of improved unnatural base pairs. More broadly, this work provides a theoretical framework for the rational development of expanded genetic alphabets, high-affinity aptamers, and other functional nucleic acid systems.

## Experimental Section

### Quantum Chemical Computations

All calculations were carried out using the Amsterdam Density Functional (ADF) program using the dispersion-corrected density functional theory at the ZORA-BLYP-D3­(BJ)/TZ2P level of theory.^35,36^ This level of theory has been previously proven to correctly address π–π stacking interactions based on benchmark analyses.^21,37^ The chosen dispersion correction in this level of theory has also shown to correctly describe noncovalent interactions in nucleobase systems.^38,39^ Solvent effects were modeled using the Conductor-like Screening Model (COSMO) as implemented in the ADF program. With the aim to further assess the robustness of the computational methodology, representative benchmark calculations were additionally performed for the Ds–Px and Es–Px systems using B3LYP-D3­(BJ), M06–2X, and ωB97X-D functionals with the TZ2P basis set, as well as BLYP-D3­(BJ)/QZ4P. Although the absolute interaction energies vary slightly among methods, all of them reproduce the same qualitative energetic trends (Table S14), confirming that the conclusions of the present work are not sensitive to the choice of density functional or basis set.

Base-pair and π–π stacking interactions were analyzed by means of quantitative Kohn–Sham molecular orbital theory together with a Morokuma-Ziegler-type energy decomposition analysis (EDA) in the gas phase. The interaction energy Δ*E*_int_ was decomposed into the classical electrostatic interaction Δ*V*_elstat_, Pauli repulsion between occupied orbitals Δ*E*_Pauli_, stabilizing orbital interactions Δ*E*_oi_, and dispersion Δ*E*_disp_.^40,41^ The electron density distribution was analyzed by using the Voronoi Deformation Density (VDD) method, which monitors if charge flows away or toward the space around a certain nucleus upon the formation of the molecule from its atoms.^42^ To visualize regions of positive and negative potential, molecular electrostatic potential (MEP) isosurfaces were calculated.^43–45^ NCI plots were computed by means of the Multiwfn software using the NCI analysis based on promolecular density, where the electron density is approximately constructed by superposing the electron densities of the atoms in their free states.^46^

Stacked dimers were generated from the C_s_-optimized base pairs. To focus exclusively on π–π stacking interactions, the sugar–phosphate backbone was removed and replaced with hydrogen atoms at the glycosidic positions, allowing direct evaluation of nucleobase–nucleobase interactions.^28,47,48^ The stacked geometry was constructed by fixing the interbase-pair distance at 3.4 Å and applying a 36° twist angle to reproduce the structural constraints imposed by the B-DNA backbone while preventing relaxation of the assembly (Figure 4). For the rotational setup, each base pair was first centered in the xy plane with the glycosidic bonds aligned along the *x*-axis. A 36° rotation was then applied about the *z*-axis through this geometric center. The twist angle was defined between the planes formed by the straight lines connecting the added hydrogen atoms that represent the glycosidic bonds of each base. This backbone-truncated stacking model has been employed previously in studies of modified and ring-expanded nucleobases. Prior work has shown that, although explicit inclusion of the phosphodiester backbone can slightly affect certain stationary points, capped models provide a reliable compromise between computational efficiency and accuracy.^33,49,50^ The stacking energy (Δ*E* ^stacking^) was calculated as Δ*E* ^stacking^ = *E*(X-Y//X′-Y′) – *E*(X-Y) – *E*(X′-Y′), where both individual base pairs and the stacked system were fully optimized under C_s_ symmetry constraints.

For completeness, with the aim to further support the suitability of our model system, a trimer including the sugar–phosphate backbone has been analyzed. In particular, we have taken the experimental crystal structure of a B-DNA dodecamer from the PDB (1BNA) and built a trimer in which we have kept the G–C base pair above and below and substituted Ds–Px in the middle (Figure 8). This latter has been allowed to fully relax, whereas only the coordinate along the stacking direction of the G–C pairs has been constrained. Noticeably, Ds–Px adopts an almost planar conformation. Computed stacking interactions of Ds-Px with the G-C above and below amount to −17.5 and −16.0 kcal mol^–1^, respectively, with a difference smaller than 1 kcal mol^–1^ compared to that computed with our model system, thus reinforcing the suitability of the chosen procedure above. Finally, to further assess the effect of the imposed structural constraints, vibrational frequency calculations were performed for the isolated bases and base pairs (Tables S15 and S16). Although some constrained structures exhibit low-magnitude imaginary frequencies associated with shallow out-of-plane distortions, relaxation of these modes changes the relative energies by less than 0.5 kcal mol^–1^.

**Figure 8:**
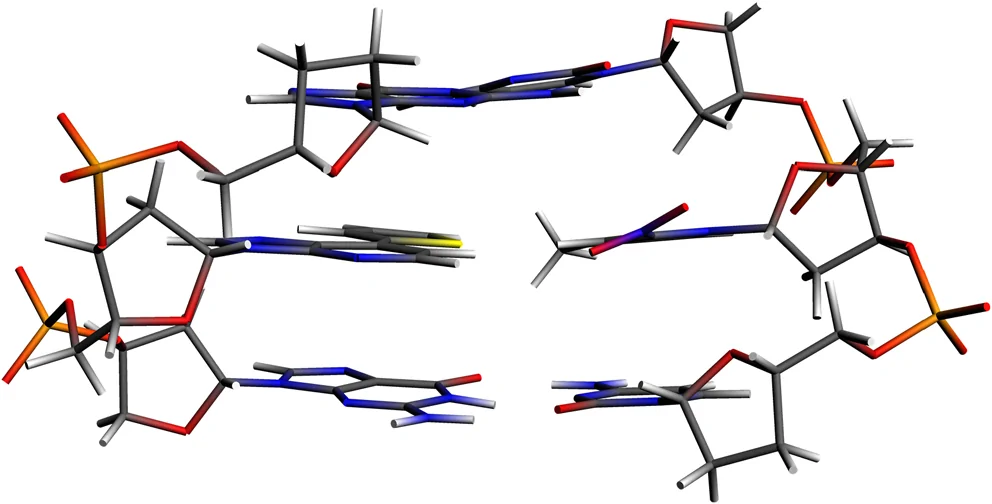
Equilibrium geometry of the G–C//Ds–Px//G–C trimer including the sugar–phosphate backbone and four sodium atoms as counterions, mimicking a B-DNA environment.

## Supplementary Material



## Data Availability

The data underlying this study are available in the published article and its Supporting Information.

## Notes

Base-pair binding energies and d_H–H_ values in different environments, MEP isosurfaces and NCI plots for selected cases, solvation energies, analysis of the influence of the twist angle on π–π stacking in Ds-Px//W–C systems, π–π stacking interaction energies in different environments, and Cartesian coordinates of all systems under analysis together with their ADF electronic energies (PDF)
