# Supplementary material for: Toward Expanded Genetic Alphabets: Insights into Functionalized Ds–Px Unnatural Base Pairs
Source: ACS Phys Chem Au. 2026 Aug 6;6(5):1264–73. doi: 10.1021/acsphyschemau.6c00082 (PMC13614050; doi:10.1021/acsphyschemau.6c00082)
Supplement: Supplementary file 1 [file pg6c00082_si_001.pdf]

SUPPORTING INFORMATION

Toward Expanded Genetic Alphabets: Insights into Functionalized Ds–Px Unnatural Base Pairs

Andreu Bermejo, Josep M. Bofill,* and Jordi Poater*

AUTHOR INFORMATION

Corresponding Authors

Jordi Poater - *Departament de Química Inorgànica i Orgànica & IQTCUB, Universitat de Barcelona, Martí i Franquès 1-11, 08028 Barcelona, Spain; ICREA, Passeig Lluís Companys 23, 08010 Barcelona, Spain; orcid.org/0000-0002-0814-5074;*

E-mail: jordi.poater@ub.edu

Josep Maria Bofill - *Departament de Química Inorgànica i Orgànica & IQTCUB, Universitat de Barcelona, Martí i Franquès 1-11, 08028 Barcelona, Spain; orcid.org/0000-0002-0974-4618;*

E-mail: jmbofill@ub.edu

Authors

Andreu Bermejo - *Departament de Química Inorgànica i Orgànica & IQTCUB, Universitat de Barcelona, Martí i Franquès 1-11, 08028 Barcelona, Spain; orcid.org/0009-0008-5340-7695*

CONTENTS

S1. Base-Pair Binding Energies and $d_{H–H}$ of Ds–Px Variants in Different Environments S3

S2. EDA of Ds–Px and Watson–Crick Base Pairs S5

S3. MEP Isosurfaces of Ds–Px and Watson–Crick Base Pairs S6

S4. NCI Plots of Ds–Px and Watson–Crick Base Pairs S7

S5. Solvation Energies of Ds–Px Variants and Watson–Crick Base Pairs S8

S6. MEP Isosurfaces of Ds Variants S9

S7. COSMO Surface and Solvent Effects on the Ds–Px Base Pair S10

S8. Influence of Twist Angle on π-π Stacking in Ds–Px//W–C Systems S11

S9. NCI Plots of Ds–Px//A–T and Ds–Px//G–C at Selected Twist Angles S12

S10. π-π Stacking Interaction Energies of Ds–Px//W–C Variants in Different Environments S13

S11. Cartesian Coordinates and Total Bonding Energies S16

S11.1. Gas Phase S16

S11.2. Chloroform S79

S11.3. Methanol S142

S11.4. Water S205

S12. Benchmark analysis of Ds–Px and Es–Px Base Pairs S268

S13. Imaginary frequencies of UBPs and their bases constrained in C_s symmetry S281

S1. Base-Pair Binding Energies and $d_{H–H}$ of Ds–Px Variants in Different Environments

UBP	ΔE (kcal mol^{-1})	$d_{H–H}$ (Å)	UBP	ΔE (kcal mol^{-1})	$d_{H–H}$ (Å)
Ds–Px	-3.9	11.43	Dss–Px	-4.1	11.24
Bs–Px	-4.2	11.54	Dt–Px	-3.8	11.41
Is–Px	-4.0	11.46	Yo–Px	-4.6	11.47
Ys–Px	-3.6	11.46	Ps–Px	-5.7	10.75
Yi–Px	-3.7	10.74	Es–Px	-5.6	10.67
Do–Px	-4.9	11.54	Ysm–Px	-4.4	11.62
Dp–Px	-5.2	11.43	Yss–Px	-3.8	11.26

TABLE S1: Base-pair binding energies (ΔE, kcal mol^{-1}) and the separation between the hydrogens placed at the glycosidic positions ($d_{H–H}$, Å) obtained for the Ds–Px variants in the gas phase.

UBP	ΔE (kcal mol^{-1})	$d_{H–H}$ (Å)	UBP	ΔE (kcal mol^{-1})	$d_{H–H}$ (Å)
Ds–Px	-2.9	11.46	Dss–Px	-2.8	11.44
Bs–Px	-3.1	11.61	Dt–Px	-2.8	11.46
Is–Px	-3.1	11.53	Yo–Px	-3.3	11.56
Ys–Px	-2.8	11.59	Ps–Px	-3.9	10.79
Yi–Px	-2.1	10.73	Es–Px	-3.9	10.72
Do–Px	-3.5	11.61	Ysm–Px	-3.6	11.49
Dp–Px	-2.1	11.58	Yss–Px	-2.7	11.33

TABLE S2: Base-pair binding energies (ΔE, kcal mol^{-1}) and the separation between the hydrogens placed at the glycosidic positions ($d_{H–H}$, Å) obtained for the Ds–Px variants in chloroform.

UBP	ΔE (kcal mol^{-1})	$d_{H–H}$ (Å)	UBP	ΔE (kcal mol^{-1})	$d_{H–H}$ (Å)
Ds–Px	-2.6	11.47	Dss–Px	-2.5	11.44
Bs–Px	-2.8	11.63	Dt–Px	-2.4	11.48
Is–Px	-2.8	11.56	Yo–Px	-3.0	11.59
Ys–Px	-2.7	11.41	Ps–Px	-3.4	10.81
Yi–Px	-1.8	10.73	Es–Px	-3.4	10.75
Do–Px	-3.1	11.62	Ysm–Px	-3.5	11.51
Dp–Px	-1.3	11.64	Yss–Px	-2.4	11.34

TABLE S3: Base-pair binding energies (ΔE, kcal mol^{-1}) and the separation between the hydrogens placed at the glycosidic positions ($d_{H\text{-}H}$, Å) obtained for the Ds-Px variants in methanol.

UBP	ΔE (kcal mol^{-1})	$d_{H–H}$ (Å)	UBP	ΔE (kcal mol^{-1})	$d_{H–H}$ (Å)
Ds–Px	-2.6	11.48	Dss–Px	-2.5	11.44
Bs–Px	-2.8	11.63	Dt–Px	-2.4	11.48
Is–Px	-2.8	11.56	Yo–Px	-3.0	11.60
Ys–Px	-2.7	11.43	Ps–Px	-3.2	10.82
Yi–Px	-1.8	10.74	Es–Px	-3.2	10.76
Do–Px	-3.0	11.63	Ysm–Px	-3.4	11.53
Dp–Px	-1.2	11.65	Yss–Px	-2.4	11.47

TABLE S4: Base-pair binding energies (ΔE, kcal mol^{-1}) and the separation between the hydrogens placed at the glycosidic positions ($d_{H–H}$, Å) obtained for the Ds–Px variants in water.

S2. EDA of Ds–Px and Watson–Crick Base Pairs

Base Pair	ΔE	ΔE_{strain}	ΔE_{int}	ΔV_{elstat}	ΔE_{Pauli}	ΔE_{oi}	ΔE_{disp}
A–T	-16.5	1.8	-18.3	-31.0	38.0	-20.1	-5.3
G–C	-30.3	3.4	-33.7	-46.6	49.4	-30.3	-6.2
Ds–Px	-3.9	0.1	-4.0	-1.9	6.3	-2.2	-6.2

Table S5: Energy decomposition analysis (EDA) for the Watson–Crick base pairs (A–T and G–C) and the unnatural base pair Ds–Px. All values are given in kcal mol^{-1}.

S3. MEP isosurfaces of Ds–Px and Watson–Crick base pairs

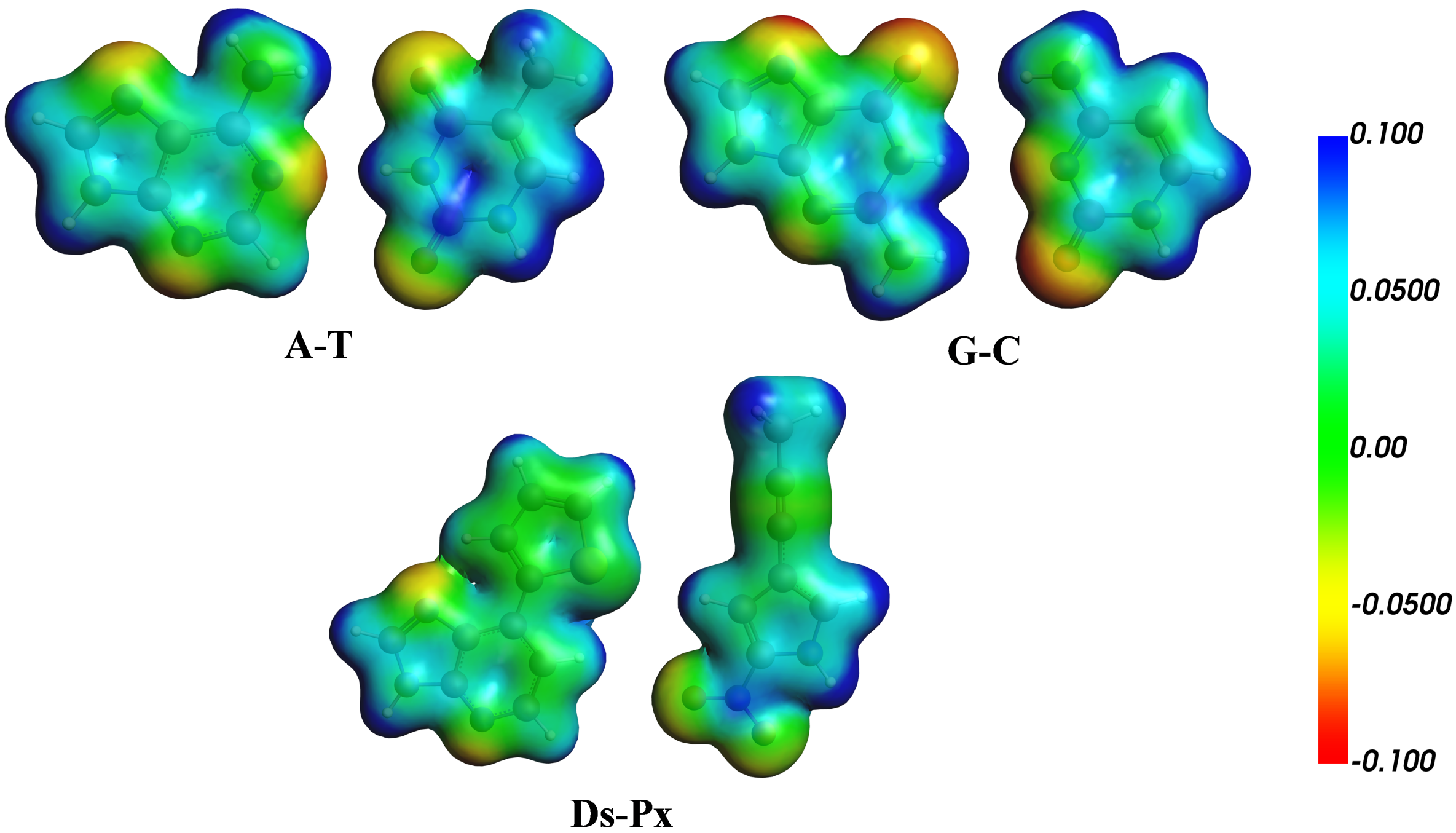

Figure S1: Molecular electrostatic potential isosurfaces (a.u., electronic density isovalue = 0.01) of Watson-Crick base pairs (A–T and G–C) and the unnatural base pair Ds–Px.

S4. NCI Plots of Ds–Px and Watson–Crick base pairs

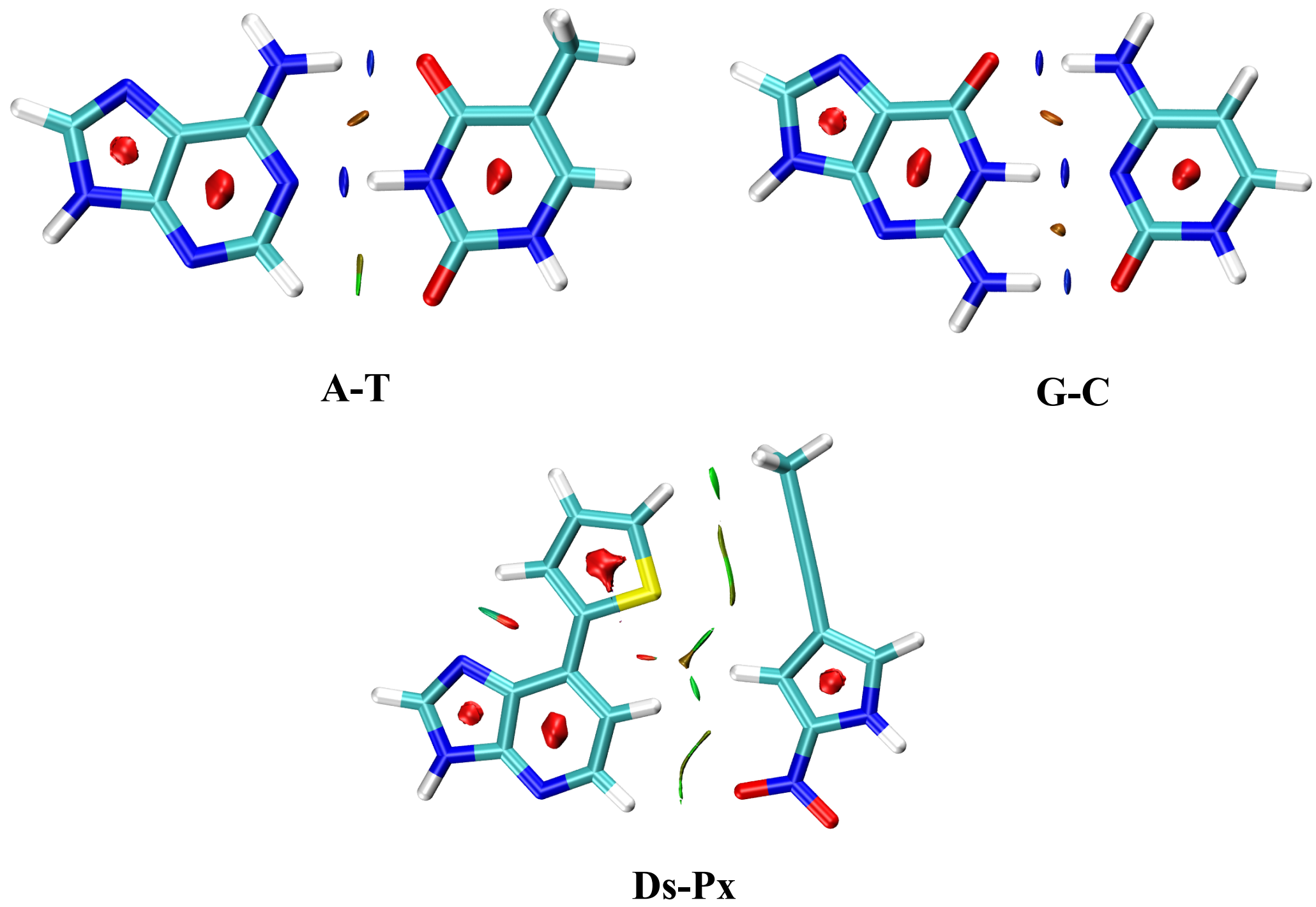

Figure S2: NCI plots (promolecular density value = 0.3 a.u.) of Watson–Crick base pairs (A–T and G–C) and the unnatural base pair Ds–Px.

S5. Solvation Energies of Ds–Px Variants and Watson–Crick Base Pairs

UBP	$\Delta E_{solv}^{chloroform}$	$\Delta E_{solv}^{methanol}$	ΔE_{solv}^{water}	UBP	$\Delta E_{solv}^{chloroform}$	$\Delta E_{solv}^{methanol}$	ΔE_{solv}^{water}
A	-9.6	-12.8	-13.6	G	-16.9	-22.7	-24.0
T	-10.5	-14.1	-15.0	C	-14.1	-18.9	-19.9
Ds	-6.1	-8.2	-8.8	Dss	-6.3	-8.5	-9.2
Bs	-5.1	-7.0	-7.6	Dt	-9.5	-13.0	-14.0
Is	-3.5	-4.8	-5.1	Yo	-4.8	-6.5	-6.9
Ys	-4.6	-6.2	-6.6	Ps	-7.5	-10.2	-10.9
Yi	-7.7	-10.5	-11.3	Es	-5.9	-8.0	-8.6
Do	-6.3	-8.5	-9.2	Ysm	-4.5	-6.0	-6.5
Dp	-9.3	-12.5	-13.4	Yss	-4.8	-6.5	-7.1
Px	-7.1	-9.6	-10.3	-	-	-	-

Table S6: Solvation energies (ΔE_{solv}, kcal mol^{-1}) of the Watson–Crick bases, the unnatural base Ds and its variants, as well as Px, computed in four different environments: gas phase, chloroform (ε = 4.8), methanol (ε = 32.6), and water (ε = 78.4).

UBP	$\Delta E_{solv}^{chloroform}$	$\Delta E_{solv}^{methanol}$	ΔE_{solv}^{water}	UBP	$\Delta E_{solv}^{chloroform}$	$\Delta E_{solv}^{methanol}$	ΔE_{solv}^{water}
A–T	-13.8	-18.6	-20.0	G–C	-17.0	-22.7	-24.2
Ds–Px	-12.2	-16.6	-17.9	Dss–Px	-12.2	-16.6	-17.9
Bs–Px	-11.2	-15.3	-16.5	Dt–Px	-15.7	-21.3	-23.0
Is–Px	-9.7	-13.2	-14.2	Yo–Px	-10.8	-14.6	-15.6
Ys–Px	-10.9	-14.8	-15.9	Ps–Px	-12.9	-17.4	-18.7
Yi–Px	-13.3	-18.2	-19.7	Es–Px	-11.3	-15.4	-16.6
Do–Px	-12.1	-16.4	-17.6	Ysm–Px	-10.8	-14.7	-15.8
Dp–Px	-13.3	-18.3	-19.8	Yss–Px	-10.8	-14.8	-16.0

Table S7: Solvation energies (ΔE_{solv}, kcal mol^{-1}) of the Watson–Crick base pairs, the unnatural base pair Ds–Px and its variants, computed in four different environments: gas phase, chloroform (ε = 4.8), methanol (ε = 32.6), and water (ε = 78.4).

S6. MEP Isosurfaces of Ds Variants

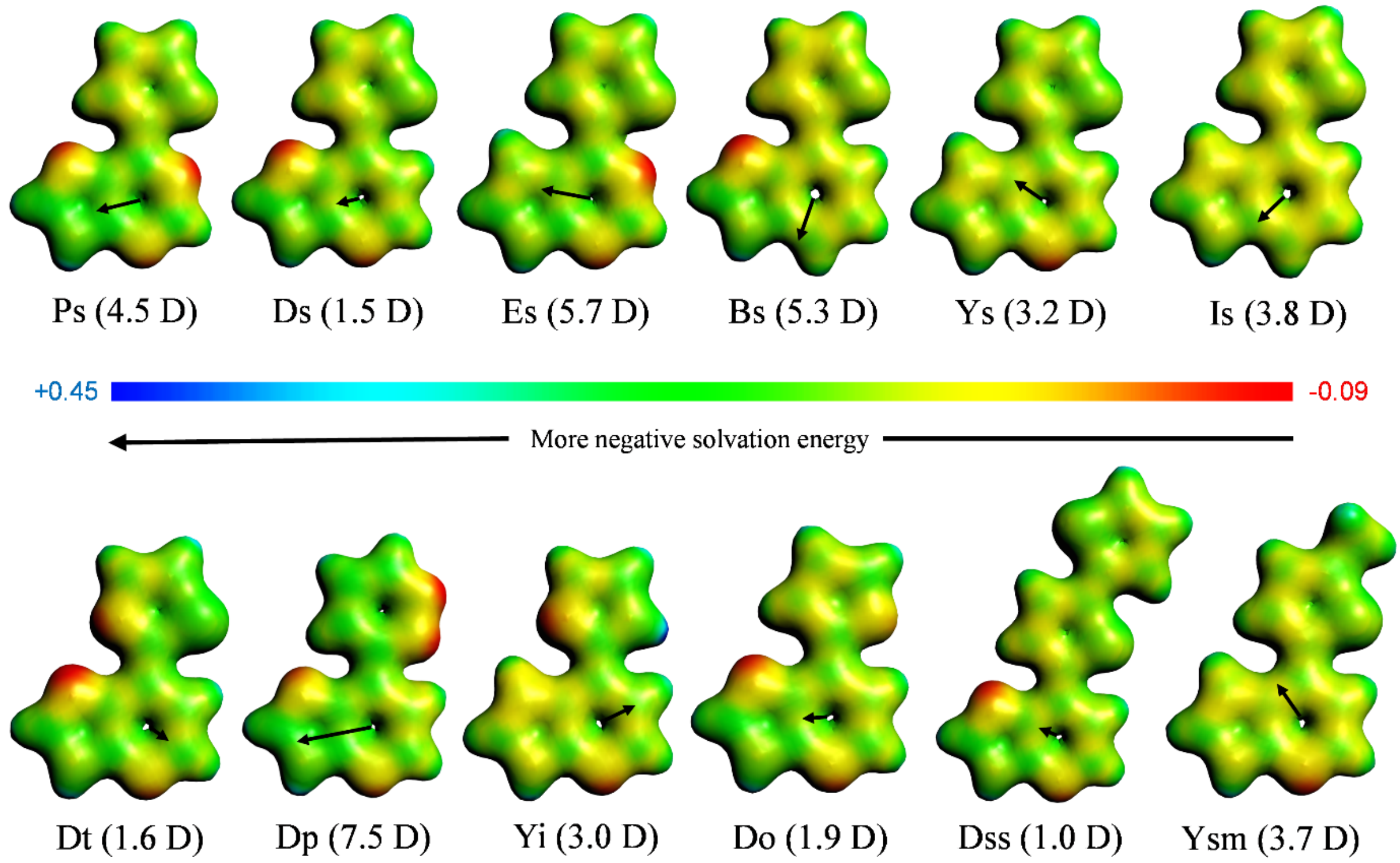

Figure S3: Molecular electrostatic potential isosurfaces (a.u., electronic density isovalue = 0.03) and dipole moment vectors (black arrows) of the unnatural DNA base pair Ds and its variants in water. Structures in the top and bottom rows allow comparison of modifications in the main module and in the side-chain module, respectively. The structures are ordered according to their solvation energies, from more negative (left) to less negative (right) values. Dipole moments are indicated in Debye below each structure.

S7. COSMO Surface and Solvent Effects on the Ds–Px Base Pair

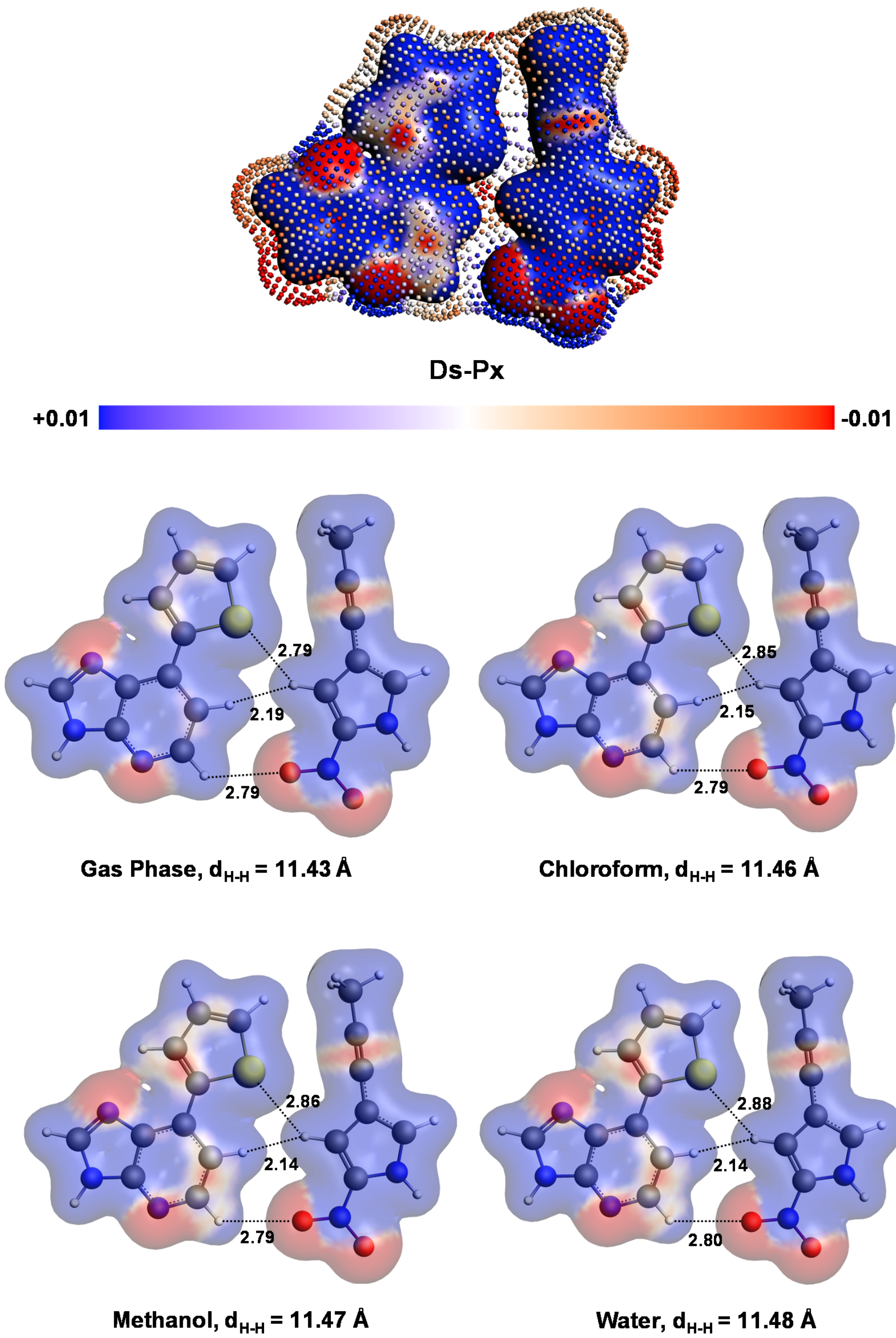

Figure S4: Top: COSMO surface representation of the unnatural DNA base pair Ds–Px. The surrounding points represent the charge density resulting from solvent polarization in response to the solute's electric field (a.u., electronic density isovalue = 0.01). Bottom: Evolution of selected distances between the bases (Å) in solvents of increasing polarity: gas phase, chloroform, methanol, and water.

S8. Influence of Twist Angle on π-π Stacking in Ds–Px//W–C Systems

Ds–Px//A–T system					
Twist-angle (°)	ΔE_{int}	ΔV_{elstat}	ΔE_{Pauli}	ΔE_{oi}	ΔE_{disp}
0	-12.0	-6.2	28.1	-4.2	-29.8
5	-12.6	-6.3	27.6	-4.2	-29.8
10	-14.1	-6.6	26.3	-4.3	-29.6
15	-15.6	-6.9	24.9	-4.4	-29.3
20	-16.5	-7.4	24.0	-4.3	-28.8
25	-16.7	-7.8	23.6	-4.1	-28.4
30	-16.7	-8.2	23.4	-4.0	-27.9
36	-16.5	-8.5	23.2	-3.9	-27.3
40	-16.4	-8.5	22.8	-3.9	-26.9
45	-16.3	-8.1	22.2	-3.9	-26.4
50	-16.0	-7.8	21.6	-3.8	-26.0
55	-15.5	-7.4	21.2	-3.7	-25.7
60	-15.1	-7.3	21.1	-3.6	-25.3
65	-15.1	-7.3	20.9	-3.6	-25.1
70	-15.2	-7.4	20.6	-3.7	-24.8
75	-15.6	-7.6	20.3	-3.7	-24.6

Table S8: Energy Decomposition Analysis (EDA) of the π-π stacking interactions in the Ds–Px//A–T system as a function of the twist-angle (0-75°) in the gas phase. All values are given in kcal mol^{-1} .

Ds–Px//G–C system					
Twist-angle (°)	ΔE_{int}	ΔV_{elstat}	ΔE_{Pauli}	ΔE_{oi}	ΔE_{disp}
0	-12.5	-4.0	23.8	-4.1	-28.2
5	-12.8	-4.1	23.4	-4.0	-28.2
10	-13.9	-4.5	22.6	-3.9	-28.1
15	-15.0	-5.0	21.8	-4.0	-27.8
20	-15.9	-5.7	21.2	-3.9	-27.4
25	-16.3	-6.2	20.6	-3.8	-27.0
30	-16.7	-6.6	20.1	-3.6	-26.5
36	-16.8	-6.9	19.6	-3.5	-26.0
40	-16.8	-7.0	19.3	-3.5	-25.6
45	-16.6	-6.9	19.1	-3.5	-25.3
50	-16.4	-6.8	18.8	-3.5	-25.0
55	-16.2	-6.7	18.6	-3.5	-24.7
60	-16.0	-6.6	18.4	-3.4	-24.4
65	-16.0	-6.8	18.3	-3.3	-24.2
70	-16.0	-7.0	18.3	-3.3	-24.0
75	-16.3	-7.4	18.2	-3.3	-23.9

Table S9: Energy Decomposition Analysis (EDA) of the π-π stacking interactions in the Ds–Px//G–C system as a function of the twist-angle (0-75°) in the gas phase. All values are given in kcal mol^{-1} .

S9. NCI Plots of Ds–Px//A–T and Ds–Px//G–C at Selected Twist Angles

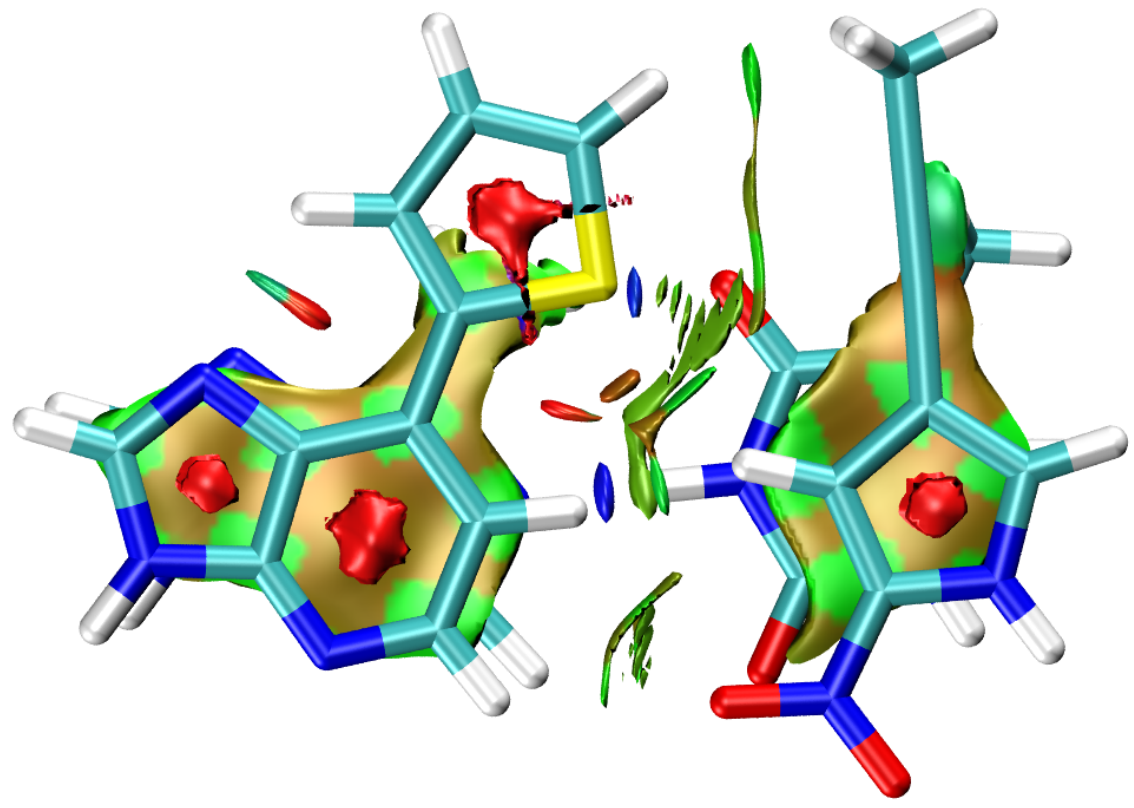

Ds–Px//A–T: 0°

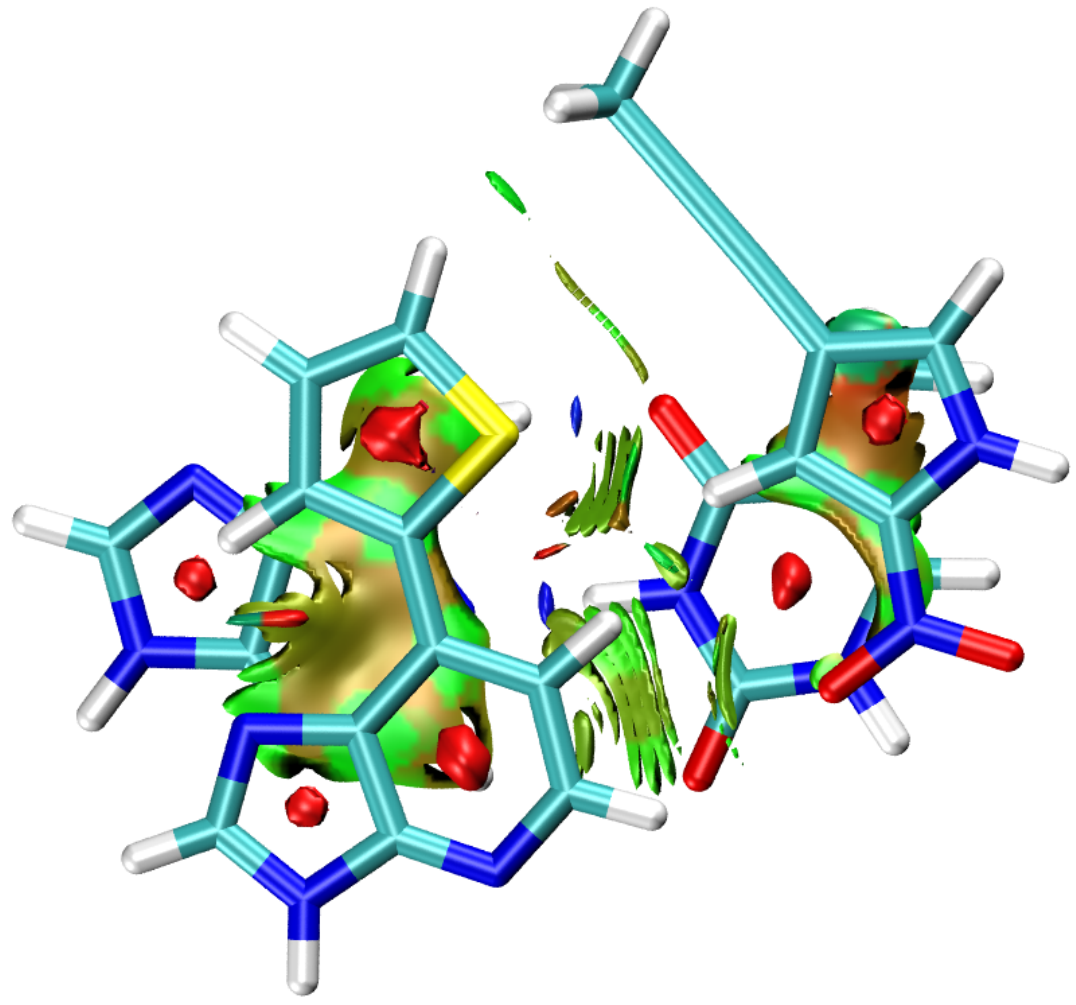

Ds–Px//A–T: 36°

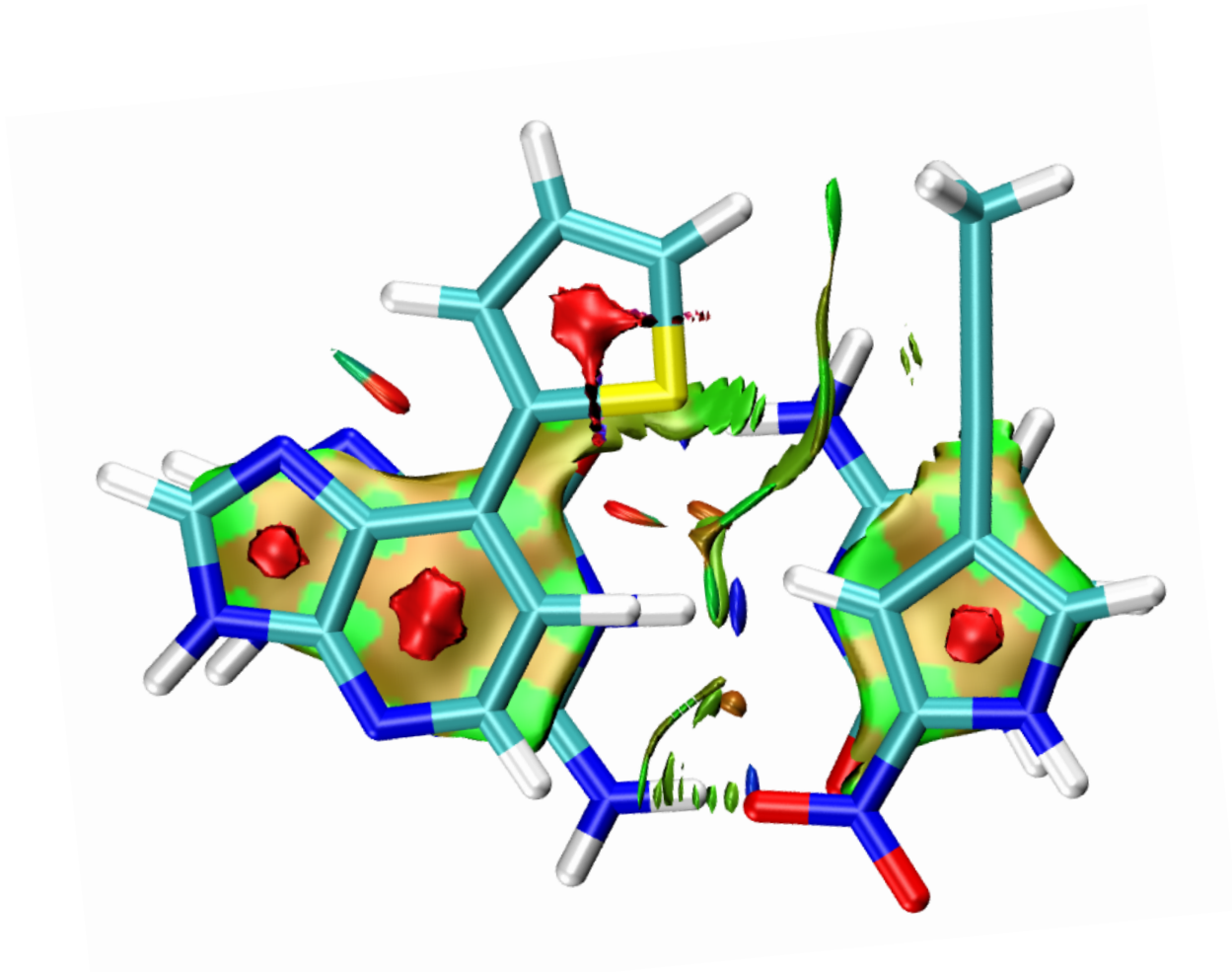

Ds–Px//G–C: 0°

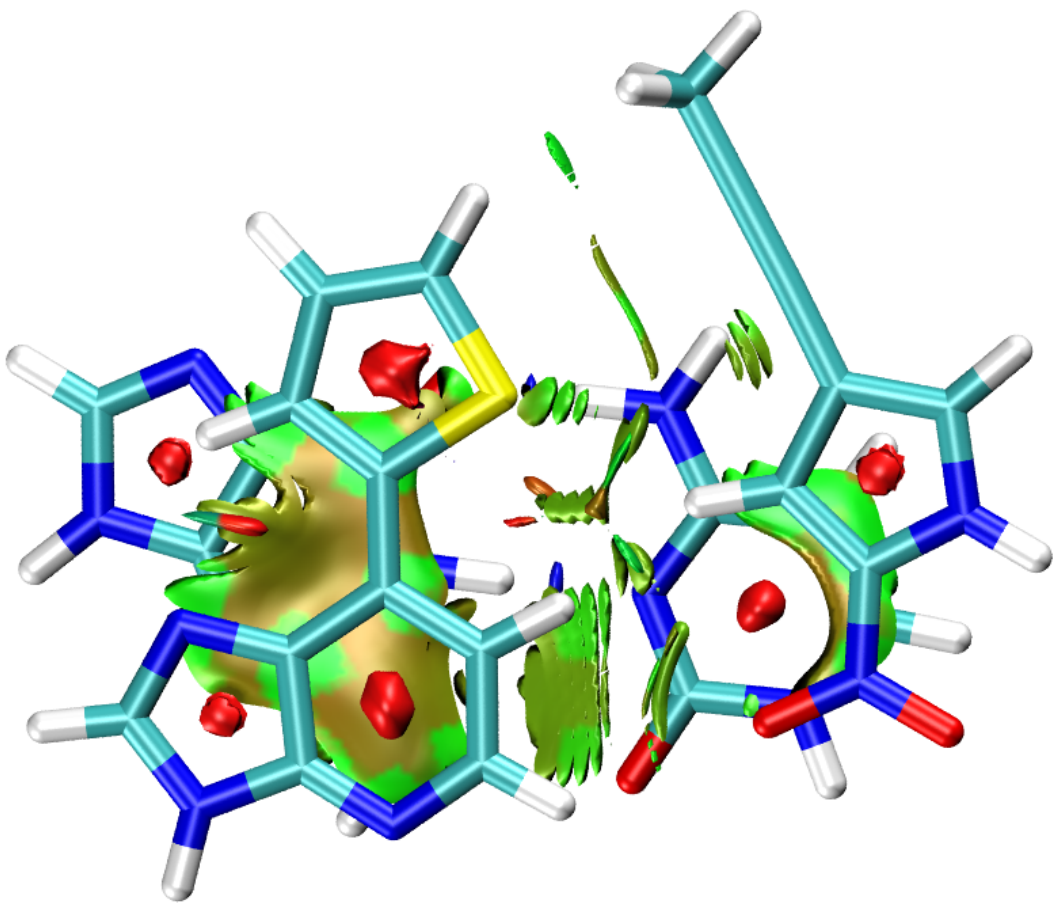

Ds–Px//G–C: 36°

Figure S5: NCI plot of the Ds–Px//A–T and Ds–Px//G–C dimers at twist-angles of 0° (left) and 36° (right). Promolecular density isovalue = 0.35 a.u.

S10. π-π Stacking Interaction Energies of Ds–Px//W–C Variants in Different Environments

UBP//A–T	ΔE (kcal mol^{-1})	UBP//A–T	ΔE (kcal mol^{-1})
Ds–Px//A–T	-16.5	Dss–Px//A–T	-16.5
Bs–Px//A–T	-16.7	Dt–Px//A–T	-16.5
Is–Px//A–T	-16.6	Yo–Px//A–T	-14.9
Ys–Px//A–T	-16.3	Ps–Px//A–T	-16.9
Yi–Px//A–T	-15.8	Es–Px//A–T	-16.5
Do–Px//A–T	-14.9	Ysm–Px//A–T	-16.1
Dp–Px//A–T	-16.5	Yss–Px//A–T	-15.9
UBP//G–C	ΔE (kcal mol^{-1})	UBP//G–C	ΔE (kcal mol^{-1})
Ds–Px//G–C	-16.8	Dss–Px//G–C	-15.6
Bs–Px//G–C	-16.1	Dt–Px//G–C	-16.7
Is–Px//G–C	-16.2	Yo–Px//G–C	-16.2
Ys–Px//G–C	-16.8	Ps–Px//G–C	-17.0
Yi–Px//G–C	-16.2	Es–Px//G–C	-16.9
Do–Px//G–C	-16.0	Ysm–Px//G–C	-16.3
Dp–Px//G–C	-19.0	Yss–Px//G–C	-15.6

TABLE S10: π-π stacking interaction energies (ΔE, kcal mol^{-1}) of Ds–Px//W–C variants in the gas phase.

UBP//A–T	ΔE (kcal mol^{-1})	UBP//A–T	ΔE (kcal mol^{-1})
Ds–Px//A–T	-13.5	Dss–Px//A–T	-13.6
Bs–Px//A–T	-13.3	Dt–Px//A–T	-12.9
Is–Px//A–T	-13.6	Yo–Px//A–T	-12.6
Ys–Px//A–T	-13.8	Ps–Px//A–T	-13.5
Yi–Px//A–T	-12.6	Es–Px//A–T	-13.8
Do–Px//A–T	-12.0	Ysm–Px//A–T	-13.7
Dp–Px//A–T	-12.5	Yss–Px//A–T	-13.8
UBP//G–C	ΔE (kcal mol^{-1})	UBP//G–C	ΔE (kcal mol^{-1})
Ds–Px//G–C	-13.6	Dss–Px//G–C	-12.7
Bs–Px//G–C	-12.9	Dt–Px//G–C	-13.0
Is–Px//G–C	-13.2	Yo–Px//G–C	-13.3

Ys–Px//G–C	-13.9	Ps–Px//G–C	-13.3
Yi–Px//G–C	-12.8	Es–Px//G–C	-13.6
Do–Px//G–C	-12.8	Ysm–Px//G–C	-13.4
Dp–Px//G–C	-13.8	Yss–Px//G–C	-13.0

TABLE S11: π-π stacking interaction energies (Δ*E*, kcal mol^{-1}) of Ds–Px//W–C variants in chloroform.

UBP//A–T	Δ*E* (kcal mol^{-1})	UBP//A–T	Δ*E* (kcal mol^{-1})
Ds–Px//A–T	-12.7	Dss–Px//A–T	-12.8
Bs–Px//A–T	-12.2	Dt–Px//A–T	-11.7
Is–Px//A–T	-12.7	Yo–Px//A–T	-11.9
Ys–Px//A–T	-13.4	Ps–Px//A–T	-12.7
Yi–Px//A–T	-11.7	Es–Px//A–T	-13.1
Do–Px//A–T	-11.2	Ysm–Px//A–T	-13.1
Dp–Px//A–T	-11.3	Yss–Px//A–T	-13.2
UBP//G–C	Δ*E* (kcal mol^{-1})	UBP//G–C	Δ*E* (kcal mol^{-1})
Ds–Px//G–C	-12.8	Dss–Px//G–C	-12.0
Bs–Px//G–C	-12.1	Dt–Px//G–C	-11.9
Is–Px//G–C	-12.4	Yo–Px//G–C	-12.5
Ys–Px//G–C	-13.1	Ps–Px//G–C	-12.3
Yi–Px//G–C	-11.8	Es–Px//G–C	-12.7
Do–Px//G–C	-12.0	Ysm–Px//G–C	-12.6
Dp–Px//G–C	-12.1	Yss–Px//G–C	-12.4

TABLE S12: π-π stacking interaction energies (Δ*E*, kcal mol^{-1}) of Ds–Px//W–C variants in methanol.

UBP//A–T	Δ*E* (kcal mol^{-1})	UBP//A–T	Δ*E* (kcal mol^{-1})
Ds–Px//A–T	-12.8	Dss–Px//A–T	-12.9
Bs–Px//A–T	-12.3	Dt–Px//A–T	-12.0
Is–Px//A–T	-12.9	Yo–Px//A–T	-12.1
Ys–Px//A–T	-13.5	Ps–Px//A–T	-12.7
Yi–Px//A–T	-11.8	Es–Px//A–T	-13.2
Do–Px//A–T	-11.3	Ysm–Px//A–T	-13.4
Dp–Px//A–T	-11.9	Yss–Px//A–T	-13.1
UBP//G–C	Δ*E* (kcal mol^{-1})	UBP//G–C	Δ*E* (kcal mol^{-1})
Ds–Px//G–C	-13.0	Dss–Px//G–C	-12.4

Bs–Px//G–C	-12.4	Dt–Px//G–C	-12.2
Is–Px//G–C	-12.7	Yo–Px//G–C	-12.7
Ys–Px//G–C	-13.4	Ps–Px//G–C	-12.5
Yi–Px//G–C	-12.1	Es–Px//G–C	-13.0
Do–Px//G–C	-12.2	Ysm–Px//G–C	-12.8
Dp–Px//G–C	-12.3	Yss–Px//G–C	-12.7

TABLE S13: π-π stacking interaction energies (Δ*E*, kcal mol^{-1}) of Ds–Px//W–C variants in water.

S11. Cartesian Coordinates and Total Bonding Energies

S11.1. Gas Phase

Cartesian Coordinates and Total Bonding Energies of W–C base pairs in the Gas Phase

Cartesian coordinates (Å) and total bonding energies (kcal mol^{-1}) of Watson–Crick base pairs optimized at ZORA-BLYP-D3(BJ)/TZ2P level of theory in the gas phase.

A–T

Total bonding energy = -4490.74 kcal mol^{-1}

N	1.26540244	-0.60369110	0.00000000
C	1.87747865	-1.80938847	0.00000000
N	3.19446042	-2.06250893	0.00000000
C	3.91468343	-0.92521780	0.00000000
C	3.41808014	0.38955004	0.00000000
C	2.00897920	0.53634184	0.00000000
N	5.29249635	-0.77739483	0.00000000
C	5.55308337	0.58827683	0.00000000
N	4.45470666	1.31994791	0.00000000
N	1.38621436	1.73060424	0.00000000
H	1.20479263	-2.66608739	0.00000000
H	5.96706248	-1.53396343	0.00000000
H	6.56573712	0.97264305	0.00000000
H	1.94806965	2.57111986	0.00000000
H	0.35882981	1.78506477	0.00000000
N	-1.55140060	-0.57632800	0.00000000
C	-2.16491838	-1.81879674	0.00000000
N	-3.56609366	-1.74274521	0.00000000
C	-4.26713612	-0.55449963	0.00000000
C	-3.64671256	0.65337345	0.00000000
C	-2.18044560	0.67118057	0.00000000
O	-1.50883563	1.71850837	0.00000000
O	-1.56567873	-2.88983820	0.00000000
C	-4.38201218	1.96552153	0.00000000
H	-0.49056154	-0.58818551	0.00000000
H	-4.04759803	-2.63502361	0.00000000
H	-5.34961352	-0.65278849	0.00000000
H	-4.11168277	2.56296861	0.87918469
H	-5.46570459	1.80838775	0.00000000
H	-4.11168277	2.56296861	-0.87918469

G–C

Total bonding energy = -4396.41 kcal mol^{-1}

N	-2.60282567	-0.57255291	0.00000000
O	1.37038223	1.81601623	0.00000000
N	-0.65936985	0.71373547	0.00000000
C	-2.03833665	0.65911156	0.00000000
N	-2.81072630	1.74686973	0.00000000
C	-2.09695489	2.89362281	0.00000000
C	-0.69898094	3.06727310	0.00000000
C	0.12410834	1.89594403	0.00000000

N	-2.61030653	4.17545039	0.00000000
C	-1.52148444	5.05128895	0.00000000
N	-0.36778176	4.42109767	0.00000000
H	-2.05090016	-1.43921256	0.00000000
H	-3.61217782	-0.61255400	0.00000000
H	-0.12336385	-0.17952228	0.00000000
H	-3.59585525	4.41145736	0.00000000
H	-1.65900447	6.12563197	0.00000000
O	-1.03453993	-3.01368714	0.00000000
N	2.83910019	-0.52094187	0.00000000
N	0.89111378	-1.74396255	0.00000000
C	3.01639010	-2.93710152	0.00000000
C	0.20418673	-2.91955428	0.00000000
N	0.97089898	-4.11681893	0.00000000
C	2.33602433	-4.11473023	0.00000000
C	2.23805893	-1.71973794	0.00000000
H	4.10060744	-2.91620005	0.00000000
H	0.44068280	-4.98219229	0.00000000
H	2.82567651	-5.08412273	0.00000000
H	2.27690625	0.36264852	0.00000000
H	3.84847184	-0.46725665	0.00000000

A–T//G–C

Total bonding energy = -8900.44 kcal mol^{-1}

N	5.23345609	-0.87899211	-3.40000000
C	5.59160726	0.42487566	-3.40000000
N	4.78739603	1.49807427	-3.40000000
C	3.48944633	1.14103243	-3.40000000
C	2.97248336	-0.16586411	-3.40000000
C	3.91638179	-1.22235313	-3.40000000
N	2.37051779	1.95847906	-3.40000000
C	1.25904175	1.12326311	-3.40000000
N	1.57958859	-0.15698050	-3.40000000
N	3.57383157	-2.52494979	-3.40000000
H	6.66563420	0.60627482	-3.40000000
H	2.38031100	2.97205669	-3.40000000
H	0.25118933	1.52004832	-3.40000000
H	2.59269826	-2.76892788	-3.40000000
H	4.29739424	-3.25634710	-3.40000000
N	7.29929887	-2.79404347	-3.40000000
C	8.58904448	-2.28739873	-3.40000000
N	9.57466394	-3.28621316	-3.40000000
C	9.29408141	-4.63701349	-3.40000000
C	8.02249775	-5.11341434	-3.40000000
C	6.92557797	-4.14026334	-3.40000000
O	5.72411518	-4.46343920	-3.40000000
O	8.86611365	-1.09180238	-3.40000000
C	7.68391665	-6.57893847	-3.40000000
H	6.52232389	-2.07166546	-3.40000000
H	10.53116258	-2.94988363	-3.40000000
H	10.16116050	-5.29244609	-3.40000000
H	7.08200048	-6.83916515	-2.52081531

H	8.59147851	-7.19164699	-3.40000000
H	7.08200048	-6.83916515	-4.27918469
N	4.97119005	1.05533672	-0.00000000
O	4.34528524	-3.53812458	-0.00000000
N	4.55667268	-1.23807420	-0.00000000
C	4.06244004	0.05043940	-0.00000000
N	2.75810350	0.33063542	-0.00000000
C	1.98630422	-0.77789430	-0.00000000
C	2.37868353	-2.13086291	-0.00000000
C	3.77981851	-2.42464605	-0.00000000
N	0.60593209	-0.81231267	-0.00000000
C	0.23111076	-2.15846856	-0.00000000
N	1.26559360	-2.96965052	-0.00000000
H	5.98534901	0.89040548	-0.00000000
H	4.60944716	1.99848779	-0.00000000
H	5.58898402	-1.37787815	-0.00000000
H	0.00000000	-0.00000000	-0.00000000
H	-0.81025242	-2.45627290	-0.00000000
O	7.83318423	0.57821431	-0.00000000
N	7.07225634	-3.96489646	-0.00000000
N	7.42685680	-1.69230122	-0.00000000
C	9.36213639	-3.17387907	-0.00000000
C	8.23575027	-0.59704963	-0.00000000
N	9.63845619	-0.82879603	-0.00000000
C	10.17549177	-2.08385156	-0.00000000
C	7.93637707	-2.93939270	-0.00000000
H	9.77098431	-4.17827316	-0.00000000
H	10.22420135	0.00000000	-0.00000000
H	11.25945255	-2.15100901	-0.00000000
H	6.03848799	-3.79721523	-0.00000000
H	7.42143441	-4.91346797	-0.00000000

Cartesian Coordinates and Total Bonding Energies of Ds variants and Px in the Gas Phase

Cartesian coordinates (Å) and total bonding energies (kcal mol^{-1}) of Ds variants and Px optimized at ZORA-BLYP-D3(BJ)/TZ2P level of theory in the gas phase.

Ds

Total bonding energy = -3166.82 kcal mol^{-1}

H	6.86767685	-0.63534468	0.00000000
H	1.83433668	3.44398619	0.00000000
H	6.42707676	1.90021853	0.00000000
C	2.14036974	-2.51358940	0.00000000
C	1.14437762	-3.52776929	0.00000000
C	-0.13562419	-3.02179749	0.00000000
S	-0.14392463	-1.28919858	0.00000000
C	1.61927889	-1.22789781	0.00000000
H	0.59046440	1.33061612	0.00000000
H	3.20924615	-2.69167457	0.00000000
H	1.36313491	-4.59112852	0.00000000
H	-1.07414100	-3.56174146	0.00000000
C	1.67661442	1.29129123	0.00000000
C	2.38254377	2.50322907	0.00000000
N	3.72890361	2.60600303	0.00000000
C	4.33833602	1.42043945	0.00000000
C	3.74718143	0.12580671	0.00000000
C	2.33278630	0.03973212	0.00000000
N	5.70908062	1.18505429	0.00000000
C	5.88188352	-0.18577753	0.00000000
N	4.74178510	-0.85271231	0.00000000

Bs

Total bonding energy = -3269.03 kcal mol^{-1}

H	6.85516367	-0.72153809	0.00000000
H	1.71098527	3.45097255	0.00000000
H	6.51607953	1.81184566	0.00000000
C	2.15567303	-2.49554918	0.00000000
C	1.16080512	-3.51303747	0.00000000
C	-0.11995296	-3.01300472	0.00000000
S	-0.12916297	-1.27825708	0.00000000
C	1.63598159	-1.21020372	0.00000000
H	0.56614104	1.29405322	0.00000000
H	3.22392661	-2.67400659	0.00000000
H	1.38413675	-4.57574576	0.00000000
H	-1.05769293	-3.55414822	0.00000000
C	1.65324348	1.29973532	0.00000000
C	2.30831573	2.54261582	0.00000000
C	3.70152930	2.63831767	0.00000000
C	4.39802179	1.42740482	0.00000000
C	3.75870249	0.15554191	0.00000000
C	2.33967132	0.06864953	0.00000000
N	5.76017341	1.13857557	0.00000000
C	5.88514895	-0.23846198	0.00000000

N	4.72333773	-0.85791303	0.00000000
H	4.20719483	3.60052148	0.00000000

Is

Total bonding energy = -3366.22 kcal mol^{-1}

H	7.07175780	-0.50947223	0.00000000
H	1.69103801	3.40312826	0.00000000
H	6.46653511	1.94616224	0.00000000
C	2.07781571	-2.55034575	0.00000000
C	1.04252958	-3.52776842	0.00000000
C	-0.21420521	-2.97428761	0.00000000
S	-0.14519250	-1.24174731	0.00000000
C	1.62467326	-1.23960017	0.00000000
H	0.57280336	1.23311925	0.00000000
H	3.12427918	-2.81766228	0.00000000
H	1.22421945	-4.59833296	0.00000000
H	-1.17418511	-3.47468009	0.00000000
C	1.65960041	1.24928577	0.00000000
C	2.29652722	2.50036049	0.00000000
C	3.68633185	2.59923344	0.00000000
C	4.40367661	1.40052158	0.00000000
C	3.78670499	0.10480220	0.00000000
C	2.35974728	0.02774996	0.00000000
N	5.77715191	1.20723618	0.00000000
C	6.05159604	-0.15003008	0.00000000
C	4.86658396	-0.85173038	0.00000000
H	4.18890227	3.56385928	0.00000000
H	4.80676945	-1.92785728	0.00000000

Ys

Total bonding energy = -3265.02 kcal mol^{-1}

H	7.06963780	-0.42782150	0.00000000
H	1.81468202	3.39559213	0.00000000
H	6.36970733	2.02313846	0.00000000
C	2.06149391	-2.56796818	0.00000000
C	1.02545445	-3.54227520	0.00000000
C	-0.23059990	-2.98373409	0.00000000
S	-0.16095479	-1.25307741	0.00000000
C	1.60603434	-1.25789148	0.00000000
H	0.59315818	1.26914438	0.00000000
H	3.10994748	-2.83020916	0.00000000
H	1.20283623	-4.61336204	0.00000000
H	-1.19122496	-3.48306123	0.00000000
C	1.67945467	1.23973240	0.00000000
C	2.37037229	2.45880101	0.00000000
N	3.71224018	2.56501264	0.00000000
C	4.34522625	1.39070554	0.00000000
C	3.77138474	0.07297556	0.00000000
C	2.35029474	-0.00120076	0.00000000
N	5.72139109	1.24610597	0.00000000
C	6.03850745	-0.10153335	0.00000000
C	4.87800186	-0.84742365	0.00000000

H	4.85408395	-1.92589758	0.00000000

Yi

Total bonding energy = -3351.85 kcal mol^{-1}

H	6.99729835	-0.50115805	0.00000000
H	1.74620564	3.33513876	0.00000000
H	6.31859747	1.95466002	0.00000000
N	2.17180628	-2.54612262	0.00000000
C	1.13999230	-3.45854036	0.00000000
C	-0.07975691	-2.80979149	0.00000000
N	0.22585334	-1.46223024	0.00000000
C	1.60573602	-1.33392062	0.00000000
H	0.52612488	1.21316370	0.00000000
H	-0.44721323	-0.70637907	0.00000000
H	1.32579531	-4.52490274	0.00000000
H	-1.09935815	-3.16821467	0.00000000
C	1.61305064	1.17737557	0.00000000
C	2.30422670	2.39962657	0.00000000
N	3.64596167	2.51050450	0.00000000
C	4.28807021	1.33983219	0.00000000
C	3.71385023	0.02419689	0.00000000
C	2.29986420	-0.05305551	0.00000000
N	5.66157857	1.18517393	0.00000000
C	5.96684249	-0.17192934	0.00000000
C	4.80226217	-0.91043326	0.00000000
H	4.71519459	-1.98621335	0.00000000

Do

Total bonding energy = -3224.78 kcal mol^{-1}

H	0.19867003	4.12968049	0.00000000
H	-3.72400944	-1.02918112	0.00000000
H	-2.32222482	3.61610523	0.00000000
C	2.23909474	-0.60788253	0.00000000
C	3.09359484	-1.75424189	0.00000000
C	2.27463899	-2.85114020	0.00000000
O	0.95559176	-2.46665233	0.00000000
C	0.94124368	-1.07245196	0.00000000
H	-1.55795706	-2.20890878	0.00000000
H	2.51775847	0.43615320	0.00000000
H	4.17569486	-1.76380369	0.00000000
H	2.45160063	-3.91705743	0.00000000
C	-1.56786616	-1.12374735	0.00000000
C	-2.80026728	-0.45214893	0.00000000
N	-2.94675618	0.89055918	0.00000000
C	-1.78233990	1.54075594	0.00000000
C	-0.47221356	0.99142107	0.00000000
C	-0.34497867	-0.41733709	0.00000000
N	-1.58734026	2.91839103	0.00000000
C	-0.22134220	3.13078480	0.00000000
N	0.47940749	2.01070240	0.00000000

Dp

Total bonding energy = -3424.47 kcal mol^{-1}

H	-0.04490510	4.17231165	0.00000000
H	-3.91860632	-1.01986244	0.00000000
H	-2.55826845	3.62820317	0.00000000
N	1.79477531	-3.19976167	0.00000000
C	2.99353874	-2.58926920	0.00000000
C	3.14472812	-1.19959236	0.00000000
C	1.99482848	-0.42622123	0.00000000
H	4.13362581	-0.74652651	0.00000000
H	-1.72615973	-2.18200647	0.00000000
H	2.01981981	0.65831304	0.00000000
H	3.85094872	-3.26046364	0.00000000
C	0.75768861	-1.10304360	0.00000000
C	-1.75948474	-1.09802847	0.00000000
C	-2.99808310	-0.43838647	0.00000000
N	-3.14976902	0.90357648	0.00000000
C	-1.99316288	1.56031114	0.00000000
C	-0.67397571	1.01897287	0.00000000
C	-0.54267908	-0.38844247	0.00000000
N	-1.81440925	2.93984314	0.00000000
C	-0.45405307	3.16893404	0.00000000
N	0.26096480	2.05809101	0.00000000
N	0.68263803	-2.45695202	0.00000000

Dss

Total bonding energy = -4198.77 kcal mol^{-1}

H	-0.55468121	6.86630432	0.00000000
H	3.42201826	1.75051939	0.00000000
H	1.97143214	6.37379753	0.00000000
C	-2.52742147	2.17493054	0.00000000
C	-3.55589439	1.20694538	0.00000000
C	-3.09565974	-0.10409650	0.00000000
S	-1.33965855	-0.13142558	0.00000000
C	-1.24922498	1.63159435	0.00000000
H	1.28578700	0.54938141	0.00000000
H	-2.68565571	3.24707245	0.00000000
H	-4.61300499	1.45503954	0.00000000
C	-3.85081823	-1.33317981	0.00000000
C	1.26758184	1.63618133	0.00000000
C	2.49251390	2.31798229	0.00000000
N	2.62282809	3.66226356	0.00000000
C	1.44909792	4.29522875	0.00000000
C	0.14350610	3.73096604	0.00000000
C	0.02776021	2.31753874	0.00000000
N	1.24170656	5.67066027	0.00000000
C	-0.12515140	5.87156764	0.00000000
N	-0.81486724	4.74493541	0.00000000
C	-3.39361173	-2.64302331	0.00000000
C	-4.43576087	-3.61078069	0.00000000
C	-5.69001464	-3.04650218	0.00000000
S	-5.61331793	-1.31053590	0.00000000

H	-6.65313398	-3.54051568	0.00000000
H	-2.33757852	-2.89462866	0.00000000
H	-4.26572858	-4.68296678	0.00000000

Dt

Total bonding energy = -3061.04 kcal mol^{-1}

H	-0.61442745	6.85050292	0.00000000
H	3.43908484	1.79832544	0.00000000
H	1.92104036	6.39234322	0.00000000
N	-2.42508318	2.10374023	0.00000000
C	-3.40679003	1.14677917	0.00000000
C	-2.97247038	-0.15744839	0.00000000
S	-1.24374446	-0.21550300	0.00000000
C	-1.22204358	1.57732693	0.00000000
H	1.31964938	0.56418295	0.00000000
N	-0.85091327	4.72932291	0.00000000
H	-4.44837224	1.45295926	0.00000000
H	-3.56197579	-1.06541754	0.00000000
C	1.28512538	1.65039394	0.00000000
C	2.49992093	2.34912098	0.00000000
N	2.60251099	3.69490108	0.00000000
C	1.42206122	4.31059683	0.00000000
C	0.11637368	3.72809054	0.00000000
C	0.03961585	2.31484175	0.00000000
N	1.19947828	5.68069819	0.00000000
C	-0.17293436	5.86059342	0.00000000

Yo

Total bonding energy = -3323.49 kcal mol^{-1}

H	0.00483637	4.31310015	0.00000000
H	-3.67931826	-1.04418696	0.00000000
H	-2.42767472	3.55519535	0.00000000
C	2.28799775	-0.69271954	0.00000000
C	3.09679258	-1.87295252	0.00000000
C	2.23346432	-2.93288418	0.00000000
O	0.93302717	-2.48987086	0.00000000
C	0.96875930	-1.09582342	0.00000000
H	-1.50235911	-2.21056196	0.00000000
H	2.64500835	0.32546778	0.00000000
H	4.17760943	-1.92503142	0.00000000
H	2.36408681	-4.00536096	0.00000000
C	-1.52076571	-1.12594200	0.00000000
C	-2.75743873	-0.46394592	0.00000000
N	-2.90248310	0.87493362	0.00000000
C	-1.74786337	1.54454218	0.00000000
C	-0.41803509	1.00727474	0.00000000
C	-0.30728278	-0.40849914	0.00000000
N	-1.63628844	2.92455477	0.00000000
C	-0.29525674	3.27400788	0.00000000
C	0.47833415	2.13093761	0.00000000
H	1.55791308	2.12335955	0.00000000

Ps

Total bonding energy = -3063.98 kcal mol^{-1}

H	6.94117621	-0.55756278	0.00000000
H	1.71326057	3.24917890	0.00000000
H	6.42004461	1.96551977	0.00000000
C	2.13519037	-2.52731572	0.00000000
C	1.09862879	-3.49630790	0.00000000
C	-0.15832532	-2.92713875	0.00000000
S	-0.10222949	-1.19695731	0.00000000
C	1.65615758	-1.22435028	0.00000000
N	4.82447112	-0.84478557	0.00000000
H	3.19659269	-2.74854957	0.00000000
H	1.26952521	-4.56844444	0.00000000
H	-1.11690018	-3.43179069	0.00000000
N	1.68620009	1.18256110	0.00000000
C	2.33798953	2.35685455	0.00000000
N	3.67162144	2.56540485	0.00000000
C	4.35068594	1.41493581	0.00000000
C	3.80375387	0.10751912	0.00000000
C	2.39266651	0.02030925	0.00000000
N	5.72388200	1.22864570	0.00000000
C	5.94058837	-0.14187798	0.00000000

Es

Total bonding energy = -3163.85 kcal mol^{-1}

H	-0.34061327	7.08636152	0.00000000
H	3.18837061	1.63698476	0.00000000
H	2.08624674	6.30527702	0.00000000
C	-2.58863690	2.02367565	0.00000000
C	-3.51865617	0.95016866	0.00000000
C	-2.90002314	-0.28150121	0.00000000
S	-1.17366568	-0.15189986	0.00000000
C	-1.26615596	1.60430053	0.00000000
C	-0.83683159	4.91072632	0.00000000
H	-2.88419308	3.06496611	0.00000000
H	-4.59641205	1.08110665	0.00000000
H	-3.36646988	-1.25913805	0.00000000
N	1.11909354	1.63729979	0.00000000
C	2.30289845	2.27143998	0.00000000
N	2.51903860	3.59958160	0.00000000
C	1.38429452	4.30573215	0.00000000
C	0.05280739	3.77964838	0.00000000
C	-0.02744309	2.36422855	0.00000000
N	1.28976065	5.68040468	0.00000000
C	-0.05288673	6.04390893	0.00000000
H	-1.91637028	4.91262992	0.00000000

Ysm

Total bonding energy = -3633.84 kcal mol^{-1}

H	3.02847156	2.64830989	0.00000000
H	1.44489612	-3.65553262	0.00000000
H	4.06213548	0.31788220	0.00000000

C	-2.19208859	1.07761926	0.00000000
C	-3.61064390	1.15760751	0.00000000
C	-4.23489085	-0.07077663	0.00000000
S	-3.05135033	-1.35134191	0.00000000
C	-1.69654086	-0.21754470	0.00000000
H	-0.85527329	-2.80588892	0.00000000
H	-1.55842521	1.95353897	0.00000000
H	-4.15821900	2.09636731	0.00000000
H	0.36700384	2.37645298	0.00000000
C	-0.04003256	-2.08726106	0.00000000
C	1.27148920	-2.58013437	0.00000000
N	2.37019567	-1.80212950	0.00000000
C	2.10283035	-0.49450075	0.00000000
C	0.81830126	0.14873388	0.00000000
C	-0.32142873	-0.70421317	0.00000000
N	3.06655943	0.49887580	0.00000000
C	2.44628037	1.73684847	0.00000000
C	1.07758807	1.56477972	0.00000000
C	-5.70731166	-0.37833116	0.00000000
H	-6.19537648	0.04683230	0.88617387
H	-5.89504352	-1.45666646	0.00000000
H	-6.19537648	0.04683230	-0.88617387

Yss

Total bonding energy = -4296.89 kcal mol^{-1}

H	-0.27297661	7.08048539	0.00000000
H	3.33646130	1.67570393	0.00000000
H	2.14776692	6.28185893	0.00000000
C	-2.61169168	2.15691053	0.00000000
C	-3.62090982	1.16666694	0.00000000
C	-3.13086235	-0.13122760	0.00000000
S	-1.37566561	-0.11323165	0.00000000
C	-1.31764140	1.65412019	0.00000000
H	1.16400864	0.53982274	0.00000000
H	-2.83483786	3.21453652	0.00000000
H	-4.68270399	1.39357973	0.00000000
C	-3.85499824	-1.37942394	0.00000000
C	1.17775010	1.62646299	0.00000000
C	2.42266015	2.26837583	0.00000000
N	2.58262366	3.60517944	0.00000000
C	1.43427433	4.28480128	0.00000000
C	0.09472340	3.76459030	0.00000000
C	-0.03701128	2.34639301	0.00000000
N	1.34499377	5.66573440	0.00000000
C	0.01098662	6.03692188	0.00000000
C	-0.78046779	4.90642563	0.00000000
C	-3.36356064	-2.67662191	0.00000000
C	-4.38033424	-3.67152465	0.00000000
C	-5.64889701	-3.14060859	0.00000000
S	-5.61699015	-1.40245273	0.00000000
H	-2.30132570	-2.90047254	0.00000000
H	-4.18238831	-4.73895675	0.00000000

H	-6.59881619	-3.65941784	0.00000000
H	-1.85914654	4.92633260	0.00000000

Px

Total bonding energy = -2437.64 kcal mol^{-1}

C	-1.36246376	-3.84192611	0.00000000
H	-0.98430387	-4.36752048	0.88714804
H	-2.45680568	-3.92885541	0.00000000
H	-0.98430387	-4.36752048	-0.88714804
C	-1.02591620	1.20061041	0.00000000
C	-0.18376035	0.07087448	0.00000000
C	1.15996308	0.55441220	0.00000000
C	1.08333018	1.94008247	0.00000000
N	-0.24393274	2.31899863	0.00000000
O	3.29134438	2.55540606	0.00000000
H	2.06686689	-0.03143393	0.00000000
H	-0.53379494	3.29099666	0.00000000
H	-2.10496633	1.25875418	0.00000000
N	2.10311551	2.93499052	0.00000000
O	1.72729933	4.13709540	0.00000000
C	-0.60320877	-1.28119052	0.00000000
C	-0.94846290	-2.44377408	0.00000000

Cartesian Coordinates and Total Bonding Energies of Ds–Px variants in the Gas Phase

Cartesian coordinates (Å) and total bonding energies (kcal mol^{-1}) of Ds–Px variants optimized at ZORA-BLYP-D3(BJ)/TZ2P level of theory in the gas phase.

Ds–Px

Total bonding energy = -5608.35 kcal mol^{-1}

H	6.90638807	-0.97307677	0.00000000
H	2.01377987	3.28012526	0.00000000
H	6.55467338	1.57666221	0.00000000
C	2.11506878	-2.68420890	0.00000000
C	1.08182494	-3.66122023	0.00000000
C	-0.17931296	-3.11021505	0.00000000
S	-0.12235030	-1.37899179	0.00000000
C	1.64112669	-1.38072837	0.00000000
H	0.70703463	1.22775115	0.00000000
H	3.17671832	-2.90169390	0.00000000
H	1.26241203	-4.73188473	0.00000000
H	-1.13624383	-3.61406202	0.00000000
C	1.78846610	1.13675159	0.00000000
C	2.53458667	2.32505005	0.00000000
N	3.88420294	2.37713992	0.00000000
C	4.45032226	1.17050783	0.00000000
C	3.81433617	-0.10212812	0.00000000
C	2.39802213	-0.13716537	0.00000000
N	5.81242829	0.88678285	0.00000000
C	5.93692100	-0.48904297	0.00000000
N	4.77417494	-1.11579134	0.00000000
C	-4.74260005	1.72328403	0.00000000
C	-3.70966001	0.76417803	0.00000000
C	-2.47784353	1.48287382	0.00000000
C	-2.80084423	2.83119703	0.00000000
N	-4.17535181	2.96525146	0.00000000
O	-0.73174607	3.81304148	0.00000000
H	-1.48611927	1.06379631	0.00000000
H	-4.63897235	3.86733127	0.00000000
H	-5.81454319	1.58639067	0.00000000
N	-1.96903973	3.98553732	0.00000000
O	-2.54183393	5.10589051	0.00000000
C	-3.86564108	-0.64277235	0.00000000
C	-3.97109639	-1.85077123	0.00000000
C	-4.09575034	-3.30314314	0.00000000
H	-3.62183163	-3.74184455	0.88853572
H	-5.14987486	-3.60895743	0.00000000
H	-3.62183163	-3.74184455	-0.88853572

Bs–Px

Total bonding energy = -5710.86 kcal mol^{-1}

H	-1.06693962	6.78940845	0.00000000
H	3.21656812	1.73160120	0.00000000
H	1.47399221	6.50551110	0.00000000
C	-2.73675943	2.04816717	0.00000000

C	-3.72919732	1.02767127	0.00000000
C	-3.19869117	-0.24084571	0.00000000
S	-1.46478570	-0.20547872	0.00000000
C	-1.43914565	1.55998519	0.00000000
H	1.10056809	0.55091437	0.00000000
H	-2.94152179	3.11172810	0.00000000
H	-4.79715788	1.22486379	0.00000000
H	-3.71593505	-1.19050282	0.00000000
C	1.07119422	1.63610610	0.00000000
C	2.30063170	2.31534981	0.00000000
C	2.36251937	3.71037389	0.00000000
C	1.13624243	4.37970900	0.00000000
C	-0.12144534	3.71293778	0.00000000
C	-0.17565935	2.29248448	0.00000000
N	0.81678743	5.73565879	0.00000000
C	-0.56221378	5.83046565	0.00000000
N	-1.15620530	4.65505187	0.00000000
C	1.58142646	-4.87863263	0.00000000
C	0.64251109	-3.82724377	0.00000000
C	1.38467193	-2.60930320	0.00000000
C	2.72691817	-2.95900471	0.00000000
N	2.83407057	-4.33618683	0.00000000
O	3.75503873	-0.91203820	0.00000000
H	0.98550803	-1.60872991	0.00000000
H	3.72724624	-4.81657369	0.00000000
H	1.42313652	-5.94764836	0.00000000
N	3.89849727	-2.15299238	0.00000000
O	5.00676032	-2.75080323	0.00000000
C	-0.76697634	-3.95729518	0.00000000
C	-1.97653413	-4.04083833	0.00000000
C	-3.43082687	-4.13543330	0.00000000
H	-3.85930516	-3.65164299	0.88817792
H	3.31297464	4.23790292	0.00000000
H	-3.85930516	-3.65164299	-0.88817792
H	-3.75865868	-5.18305395	0.00000000

Is–Px

Total bonding energy = -5807.87 kcal mol^{-1}

H	-0.76862418	6.90762080	0.00000000
H	3.20398996	1.56470787	0.00000000
H	1.69296343	6.33092320	0.00000000
C	-2.75151195	1.88424249	0.00000000
C	-3.71316196	0.83310869	0.00000000
C	-3.14213516	-0.41544165	0.00000000
S	-1.41072139	-0.31916564	0.00000000
C	-1.43431550	1.45068462	0.00000000
H	1.05979651	0.43304171	0.00000000
H	-3.03504529	2.92651053	0.00000000
H	-4.78628943	0.99973879	0.00000000
H	-3.62595718	-1.38237806	0.00000000
C	1.05472327	1.51843469	0.00000000
C	2.29967456	2.16611367	0.00000000

C	2.37949780	3.55708317	0.00000000
C	1.17216172	4.26083473	0.00000000
C	-0.11652584	3.62952242	0.00000000
C	-0.17593068	2.20161313	0.00000000
N	0.96253827	5.63261719	0.00000000
C	-0.39797611	5.89137645	0.00000000
C	-1.08577297	4.69785395	0.00000000
C	1.64096265	-4.98899139	0.00000000
C	0.70662868	-3.93323173	0.00000000
C	1.45400318	-2.71841763	0.00000000
C	2.79488094	-3.07462833	0.00000000
N	2.89582448	-4.45199492	0.00000000
O	3.83447088	-1.03323787	0.00000000
H	1.06329535	-1.71423187	0.00000000
H	3.78676905	-4.93656795	0.00000000
H	1.47773067	-6.05723824	0.00000000
N	3.97112680	-2.27458481	0.00000000
O	5.07573626	-2.87900717	0.00000000
C	-0.70308478	-4.06310748	0.00000000
C	-1.91188629	-4.15868095	0.00000000
C	-3.36503001	-4.27128420	0.00000000
H	3.33785538	4.07156395	0.00000000
H	-3.80000515	-3.79342595	-0.88833290
H	-3.80000515	-3.79342595	0.88833290
H	-2.16115362	4.62434874	0.00000000
H	-3.67949722	-5.32289909	0.00000000

Ys–Px

Total bonding energy = -5706.30 kcal mol^{-1}

H	-0.67446320	7.02973034	0.00000000
H	3.31535786	1.89498247	0.00000000
H	1.79758805	6.40786926	0.00000000
C	-2.65202845	1.95349666	0.00000000
C	-3.59130241	0.88457789	0.00000000
C	-2.99247840	-0.35235304	0.00000000
S	-1.26571435	-0.22467441	0.00000000
C	-1.32793735	1.54121698	0.00000000
H	1.24013250	0.61461030	0.00000000
H	-2.94953711	2.99247568	0.00000000
H	-4.66763495	1.02719341	0.00000000
H	-3.45747297	-1.32813988	0.00000000
C	1.16674808	1.69738338	0.00000000
C	2.36493381	2.42483968	0.00000000
N	2.42459953	3.76990583	0.00000000
C	1.23036175	4.36413244	0.00000000
C	-0.06828054	3.74872000	0.00000000
C	-0.09518718	2.32573920	0.00000000
N	1.04137102	5.73542579	0.00000000
C	-0.31578450	6.00934350	0.00000000
C	-1.02383660	4.82486116	0.00000000
C	1.69542796	-4.90353076	0.00000000
C	0.77200410	-3.83865219	0.00000000

C	1.53161446	-2.63081129	0.00000000
C	2.86807253	-3.00090080	0.00000000
N	2.95549884	-4.37868608	0.00000000
O	3.92970527	-0.97063517	0.00000000
H	1.14752239	-1.62432001	0.00000000
H	3.84257532	-4.87049303	0.00000000
H	1.52185319	-5.97012338	0.00000000
N	4.05387999	-2.21268463	0.00000000
O	5.15125894	-2.82926965	0.00000000
C	-0.63877138	-3.95941135	0.00000000
C	-1.84709471	-4.06110810	0.00000000
C	-3.29937423	-4.18698621	0.00000000
H	-3.73939024	-3.71447136	0.88875216
H	-2.10113062	4.76670999	0.00000000
H	-3.73939024	-3.71447136	-0.88875216
H	-3.60369601	-5.24149137	0.00000000

Yi–Px

Total bonding energy = -5793.14 kcal mol^{-1}

H	-6.72831093	-0.81738589	0.00000000
H	-0.51815037	-2.74129357	0.00000000
H	-5.28471567	-2.91701685	0.00000000
N	-2.83004831	2.68532339	0.00000000
C	-2.15481400	3.88474553	0.00000000
C	-0.79061270	3.67069540	0.00000000
N	-0.63334464	2.29683773	0.00000000
C	-1.89702604	1.72612332	0.00000000
H	-0.05902862	-0.31258464	0.00000000
H	-5.05576546	1.33175355	0.00000000
H	-2.67965072	4.83157539	0.00000000
H	0.05070800	4.34657902	0.00000000
C	-1.09192020	-0.64942916	0.00000000
C	-1.34428346	-2.03123272	0.00000000
N	-2.57849880	-2.57020567	0.00000000
C	-3.56633622	-1.67289232	0.00000000
C	-3.45307994	-0.24169073	0.00000000
C	-2.14100917	0.28996123	0.00000000
N	-4.91528027	-1.97502525	0.00000000
C	-5.64679626	-0.79196380	0.00000000
C	-4.78711775	0.28642624	0.00000000
C	4.79885135	-1.13502951	0.00000000
C	3.49377582	-0.60145137	0.00000000
C	2.59350874	-1.70821458	0.00000000
C	3.37293017	-2.85489278	0.00000000
N	4.70508591	-2.49562863	0.00000000
O	1.79094052	-4.51221914	0.00000000
H	1.51669439	-1.68332984	0.00000000
H	5.45570242	-3.17829220	0.00000000
H	5.75276291	-0.62746183	0.00000000
N	3.00581030	-4.23282702	0.00000000
O	3.94372741	-5.07137181	0.00000000
C	3.18127454	0.78074345	0.00000000

C	2.99711415	1.98055820	0.00000000
C	2.83726058	3.43069057	0.00000000
H	2.29313672	3.77489155	-0.88925334
H	0.25526015	1.80636945	0.00000000
H	2.29313672	3.77489155	0.88925334
H	3.81810876	3.92327370	0.00000000

Do–Px

Total bonding energy = -5667.27 kcal mol^{-1}

H	1.11462301	6.90390370	0.00000000
H	-3.50744710	2.35656994	0.00000000
H	-1.45402341	6.75701345	0.00000000
C	2.45133825	1.92123183	0.00000000
C	3.13410546	0.66371279	0.00000000
C	2.17065742	-0.30739329	0.00000000
O	0.91797894	0.26475032	0.00000000
C	1.10097862	1.64884375	0.00000000
H	-1.55203284	0.88738794	0.00000000
H	2.87497178	2.91545220	0.00000000
H	4.20425479	0.50238728	0.00000000
H	2.18505424	-1.38776992	0.00000000
C	-1.38957025	1.95845275	0.00000000
C	-2.51365032	2.79987547	0.00000000
N	-2.46382698	4.15015300	0.00000000
C	-1.21779461	4.62551777	0.00000000
C	-0.00033658	3.89400078	0.00000000
C	-0.07927195	2.48142815	0.00000000
N	-0.82652249	5.96142547	0.00000000
C	0.55553228	5.97570027	0.00000000
N	1.08825411	4.76624960	0.00000000
C	-1.41069857	-4.72232128	0.00000000
C	-0.43911391	-3.70157198	0.00000000
C	-1.13869249	-2.45644891	0.00000000
C	-2.49212177	-2.76662339	0.00000000
N	-2.64340851	-4.13965076	0.00000000
O	-3.48020958	-0.69686662	0.00000000
H	-0.70664591	-1.46662386	0.00000000
H	-3.55311305	-4.58843792	0.00000000
H	-1.28587800	-5.79572675	0.00000000
N	-3.64701523	-1.93422790	0.00000000
O	-4.76685031	-2.50927552	0.00000000
C	0.96394327	-3.89339327	0.00000000
C	2.16705358	-4.04839159	0.00000000
C	3.61544731	-4.21818422	0.00000000
H	4.06898113	-3.75713868	-0.88766262
H	4.06898113	-3.75713868	0.88766262
H	3.88704576	-5.28158936	0.00000000

Dp–Px

Total bonding energy = -5867.34 kcal mol^{-1}

H	0.84339528	6.95065995	0.00000000
H	-3.62470549	2.25433342	0.00000000

H	-1.71727520	6.71159747	0.00000000
C	1.00244499	1.61789069	0.00000000
N	0.77489943	0.28094144	0.00000000
H	3.93980872	-4.05576182	0.88741770
H	3.93980872	-4.05576182	-0.88741770
N	1.79367337	-0.58561318	0.00000000
H	-1.61352635	0.84829513	0.00000000
C	3.05425073	-0.12163591	0.00000000
C	3.36482640	1.24159527	0.00000000
C	2.31244648	2.14259293	0.00000000
C	-1.49381325	1.92386974	0.00000000
C	-2.64529107	2.72773638	0.00000000
N	-2.63216233	4.07841412	0.00000000
C	-1.40450945	4.59076976	0.00000000
C	-0.16041438	3.89555816	0.00000000
C	-0.20216527	2.48169577	0.00000000
N	-1.06090146	5.93940712	0.00000000
C	0.31654567	6.00382224	0.00000000
N	0.89314429	4.81453989	0.00000000
C	-1.57365507	-4.79476024	0.00000000
C	-0.54875415	-3.82660685	0.00000000
C	-1.17902485	-2.54424505	0.00000000
C	-2.54911576	-2.78470777	0.00000000
N	-2.77411680	-4.14934630	0.00000000
O	-3.45150723	-0.67272697	0.00000000
H	-0.68730618	-1.57952307	0.00000000
H	-3.70638925	-4.54805533	0.00000000
H	-1.50583368	-5.87345731	0.00000000
N	-3.66444074	-1.90495566	0.00000000
O	-4.81118278	-2.42936560	0.00000000
C	0.84025811	-4.09727221	0.00000000
C	2.03398250	-4.30415261	0.00000000
C	3.47666773	-4.50902378	0.00000000
H	4.39928206	1.57803866	0.00000000
H	2.46269447	3.21707651	0.00000000
H	3.82689963	-0.88889001	0.00000000
H	3.73029169	-5.57733401	0.00000000

Dss–Px

Total bonding energy = -6640.53 kcal mol^{-1}

H	-7.19001861	2.96633289	0.00000000
H	-0.91797416	4.60938202	0.00000000
H	-5.74372990	5.09575034	0.00000000
C	-3.65327070	-0.68218955	0.00000000
C	-3.17637000	-2.01118048	0.00000000
C	-1.79160905	-2.11519549	0.00000000
S	-1.06444104	-0.51419918	0.00000000
C	-2.64700588	0.27330612	0.00000000
H	-0.65461349	2.18760909	0.00000000
H	-4.69938029	-0.39891909	0.00000000
H	-3.82766066	-2.88007551	0.00000000
H	-0.17570964	-6.78584408	0.00000000

C	-1.66299244	2.58856032	0.00000000
C	-1.80745365	3.98417436	0.00000000
N	-2.99504543	4.62927697	0.00000000
C	-4.03753123	3.79845222	0.00000000
C	-4.03179434	2.37654399	0.00000000
C	-2.77528926	1.71847966	0.00000000
N	-5.38472280	4.14821650	0.00000000
C	-6.10646936	2.97078920	0.00000000
N	-5.34142522	1.89349592	0.00000000
C	5.25886111	1.00957542	0.00000000
C	4.15242496	0.13553736	0.00000000
C	2.97908994	0.94836631	0.00000000
C	3.41372285	2.26827100	0.00000000
N	4.79395007	2.29061303	0.00000000
O	1.44715680	3.44298232	0.00000000
H	1.95143511	0.61638742	0.00000000
H	5.32537545	3.15473234	0.00000000
H	6.31578650	0.78461434	0.00000000
N	2.69499255	3.49517638	0.00000000
O	3.37051896	4.55679741	0.00000000
C	4.23177605	-1.27762370	0.00000000
C	4.34755434	-2.48472401	0.00000000
C	4.50769630	-3.93329439	0.00000000
C	-0.98187765	-3.30914375	0.00000000
C	0.39826619	-3.45034899	0.00000000
C	0.82836810	-4.80729914	0.00000000
C	-0.21313297	-5.70406620	0.00000000
S	-1.75054164	-4.89730223	0.00000000
H	4.04541866	-4.38356325	-0.88822616
H	4.04541866	-4.38356325	0.88822616
H	1.08097232	-2.60609263	0.00000000
H	1.86909862	-5.11027190	0.00000000
H	5.57032182	-4.20820101	0.00000000

Dt–Px

Total bonding energy = -5502.43 kcal mol^{-1}

H	-0.96951820	7.01840757	0.00000000
H	3.17330950	2.03394183	0.00000000
H	1.57392296	6.60461300	0.00000000
N	-2.69580190	2.23467459	0.00000000
C	-3.65648679	1.25573955	0.00000000
C	-3.19501153	-0.03906415	0.00000000
S	-1.46696289	-0.05950220	0.00000000
C	-1.48171275	1.73361639	0.00000000
H	1.09091812	0.77168919	0.00000000
H	-3.83510833	-3.50660230	-0.88868877
H	-4.70454902	1.53909092	0.00000000
H	-3.76503292	-0.95819628	0.00000000
C	1.02481826	1.85494913	0.00000000
C	2.22879116	2.57310737	0.00000000
N	2.30360259	3.92123078	0.00000000
C	1.11179480	4.51439106	0.00000000

C	-0.18327943	3.90920127	0.00000000
C	-0.23328946	2.49424534	0.00000000
N	0.86459199	5.88081307	0.00000000
C	-0.51041973	6.03651139	0.00000000
N	-1.16848008	4.89303791	0.00000000
C	1.62145188	-4.68484292	0.00000000
C	0.66734565	-3.64737026	0.00000000
C	1.39242492	-2.41918947	0.00000000
C	2.73896898	-2.74855384	0.00000000
N	2.86617636	-4.12361369	0.00000000
O	3.72771727	-0.68309508	0.00000000
H	0.97880480	-1.42514108	0.00000000
H	3.76622226	-4.59133073	0.00000000
H	1.47943289	-5.75610822	0.00000000
N	3.89639289	-1.92109677	0.00000000
O	5.01445327	-2.49767171	0.00000000
C	-0.74063647	-3.79385612	0.00000000
C	-1.94981702	-3.88413993	0.00000000
C	-3.40382147	-3.98706840	0.00000000
H	-3.83510833	-3.50660230	0.88868877
H	-3.72610408	-5.03621473	0.00000000

Yo–Px

Total bonding energy = -5765.68 kcal mol^{-1}

H	0.84785293	7.02208891	0.00000000
H	-3.52220659	2.20390089	0.00000000
H	-1.66496183	6.59695244	0.00000000
C	2.43605204	1.75906831	0.00000000
C	3.08429541	0.48262225	0.00000000
C	2.09340295	-0.45792478	0.00000000
O	0.86001975	0.15413984	0.00000000
C	1.07574779	1.53372338	0.00000000
H	-1.54231324	0.76360654	0.00000000
H	2.92250469	2.72206692	0.00000000
H	4.14959028	0.29243069	0.00000000
H	2.07599980	-1.53830518	0.00000000
C	-1.39676938	1.83673445	0.00000000
C	-2.53378547	2.65935521	0.00000000
N	-2.49547602	4.00586817	0.00000000
C	-1.26098813	4.51328491	0.00000000
C	-0.01598919	3.80221894	0.00000000
C	-0.09885815	2.38381685	0.00000000
N	-0.96482237	5.86638727	0.00000000
C	0.41079158	6.03272712	0.00000000
C	1.02340842	4.79545032	0.00000000
C	-1.46563925	-4.85503382	0.00000000
C	-0.48694711	-3.84120032	0.00000000
C	-1.17783889	-2.59101470	0.00000000
C	-2.53300365	-2.89173578	0.00000000
N	-2.69428402	-4.26345267	0.00000000
O	-3.50219039	-0.81409584	0.00000000
H	-0.74168375	-1.60301838	0.00000000

H	-3.60721613	-4.70566490	0.00000000
H	-1.34854197	-5.92930839	0.00000000
N	-3.68092362	-2.04922769	0.00000000
O	-4.80554150	-2.61498024	0.00000000
C	0.91486637	-4.04223938	0.00000000
C	2.11707483	-4.20312323	0.00000000
C	3.56453534	-4.38048701	0.00000000
H	4.02065704	-3.92183271	0.88763111
H	4.02065704	-3.92183271	-0.88763111
H	2.09234043	4.64347976	0.00000000
H	3.83018382	-5.44544558	0.00000000

Ps–Px

Total bonding energy = -5507.36 kcal mol^{-1}

H	-0.53736498	6.81214264	0.00000000
H	2.87937066	1.31852087	0.00000000
H	1.93988259	6.10854139	0.00000000
C	-2.85438729	2.15919304	0.00000000
C	-3.88999936	1.18825544	0.00000000
C	-3.40613213	-0.10393598	0.00000000
S	-1.67524131	-0.16154417	0.00000000
C	-1.58797228	1.59109674	0.00000000
H	-3.61071056	-3.40628697	-0.88867146
H	-3.00536304	3.23273064	0.00000000
H	-4.94844489	1.43034514	0.00000000
H	-3.97489757	-1.02431331	0.00000000
N	0.81567787	1.44364870	0.00000000
C	2.03870954	2.00678398	0.00000000
N	2.33715336	3.32394325	0.00000000
C	1.24052652	4.08435088	0.00000000
C	-0.10326820	3.63432753	0.00000000
C	-0.29189234	2.23466873	0.00000000
N	1.15459989	5.46747013	0.00000000
C	-0.19684796	5.78349705	0.00000000
N	-0.97945303	4.72187416	0.00000000
C	1.81140612	-4.44700128	0.00000000
C	0.90325294	-3.36882162	0.00000000
C	1.67607604	-2.16524632	0.00000000
C	3.00950018	-2.56279810	0.00000000
N	3.07623670	-3.94265293	0.00000000
O	4.15926659	-0.57393817	0.00000000
H	1.31267445	-1.14458097	0.00000000
H	3.95725971	-4.44512121	0.00000000
H	1.61966696	-5.51054708	0.00000000
N	4.22392392	-1.82062792	0.00000000
O	5.29716813	-2.48061753	0.00000000
C	-0.50566402	-3.51143416	0.00000000
C	-1.70664456	-3.67706506	0.00000000
C	-3.15254887	-3.86136590	0.00000000
H	-3.61071056	-3.40628697	0.88867146
H	-3.41480905	-4.92720475	0.00000000

Es–Px

Total bonding energy = -5607.06 kcal mol^{-1}

H	-0.28648996	6.87622008	0.00000000
H	2.91503396	1.22508965	0.00000000
H	2.09139125	5.95206181	0.00000000
C	-2.83053134	1.95732353	0.00000000
C	-3.81825663	0.93590369	0.00000000
C	-3.26934124	-0.32835528	0.00000000
S	-1.53841180	-0.29453898	0.00000000
C	-1.53478996	1.46267656	0.00000000
H	-1.98755284	4.80017067	0.00000000
H	-3.06857356	3.01323762	0.00000000
H	-4.88729782	1.12589123	0.00000000
H	-3.79097049	-1.27572256	0.00000000
N	0.84989530	1.35027465	0.00000000
C	2.07362972	1.91223623	0.00000000
N	2.36136582	3.22690021	0.00000000
C	1.27166286	3.99846464	0.00000000
C	-0.08832009	3.55230622	0.00000000
C	-0.25162276	2.14536988	0.00000000
N	1.25840958	5.37661209	0.00000000
C	-0.06050382	5.81867248	0.00000000
C	-0.91003169	4.73363864	0.00000000
C	1.83569078	-4.55576264	0.00000000
C	0.92644660	-3.47810990	0.00000000
C	1.69792709	-2.27389971	0.00000000
C	3.03188907	-2.67087816	0.00000000
N	3.09963545	-4.05071314	0.00000000
O	4.18381465	-0.68343160	0.00000000
H	1.33716682	-1.25228649	0.00000000
H	3.98218522	-4.55064574	0.00000000
H	1.64323066	-5.61915041	0.00000000
N	4.24696735	-1.92949141	0.00000000
O	5.31957064	-2.59144812	0.00000000
C	-0.48176080	-3.63137201	0.00000000
C	-1.67847714	-3.82868581	0.00000000
C	-3.11765964	-4.06206363	0.00000000
H	-3.59218900	-3.62479181	0.88882449
H	-3.59218900	-3.62479181	-0.88882449
H	-3.34094336	-5.13691068	0.00000000

Ysm–Px

Total bonding energy = -6075.91 kcal mol^{-1}

H	0.27078571	7.45673777	0.00000000
H	-3.72716728	2.32739514	0.00000000
H	-2.20306863	6.83983924	0.00000000
C	2.23818089	2.36654796	0.00000000
C	3.16838934	1.28833381	0.00000000
C	2.57902432	0.04491607	0.00000000
S	0.84013180	0.20238819	0.00000000
C	0.91188747	1.97006075	0.00000000
H	-1.65643833	1.04117181	0.00000000

H	2.54591765	3.40306090	0.00000000
H	4.24650337	1.42455772	0.00000000
C	3.22972516	-1.30690045	0.00000000
C	-1.58018028	2.12425605	0.00000000
C	-2.77579964	2.85603773	0.00000000
N	-2.83558483	4.20149816	0.00000000
C	-1.64035129	4.79471099	0.00000000
C	-0.34361759	4.17722203	0.00000000
C	-0.31847706	2.75419323	0.00000000
N	-1.44819089	6.16601407	0.00000000
C	-0.08992360	6.43705823	0.00000000
C	0.61494034	5.25048881	0.00000000
C	-1.95789318	-4.99320158	0.00000000
C	-0.88278375	-4.08235120	0.00000000
C	-1.44743459	-2.77151116	0.00000000
C	-2.82517460	-2.93294223	0.00000000
N	-3.12260983	-4.28160601	0.00000000
O	-3.56709672	-0.76442610	0.00000000
H	-0.91308553	-1.83462947	0.00000000
H	-4.07458507	-4.63173910	0.00000000
H	-1.95002134	-6.07384030	0.00000000
N	-3.87712997	-1.97390913	0.00000000
O	-5.05533378	-2.41674083	0.00000000
C	0.49455644	-4.41043511	0.00000000
C	1.67846561	-4.67340322	0.00000000
C	3.10328140	-4.98318978	0.00000000
H	3.59958686	-4.56734609	-0.88677215
H	2.94426638	-1.89404023	0.88172128
H	4.31891515	-1.19320926	0.00000000
H	2.94426638	-1.89404023	-0.88172128
H	1.69219048	5.18835064	0.00000000
H	3.59958686	-4.56734609	0.88677215
H	3.27134600	-6.06803173	0.00000000

Yss–Px
Total bonding energy = -6738.35 kcal mol^{-1}

H	-7.20834961	3.43760316	0.00000000
H	-0.81360477	4.62551678	0.00000000
H	-5.52091504	5.34902135	0.00000000
C	-3.59626658	-0.64446528	0.00000000
C	-3.08539254	-1.96249728	0.00000000
C	-1.69999064	-2.02679324	0.00000000
S	-1.02239431	-0.40579796	0.00000000
C	-2.62466055	0.34611031	0.00000000
H	-0.62600314	2.19572762	0.00000000
H	-4.65569109	-0.43046233	0.00000000
H	-3.71398237	-2.84792368	0.00000000
C	-0.84989891	-3.19202983	0.00000000
C	-1.62124431	2.62760094	0.00000000
C	-1.72102840	4.02543992	0.00000000
N	-2.88947557	4.69599855	0.00000000
C	-3.96539001	3.90702736	0.00000000

C	-4.01439779	2.47137146	0.00000000
C	-2.75977284	1.79600970	0.00000000
N	-5.27106505	4.36845676	0.00000000
C	-6.13710597	3.28851790	0.00000000
C	-5.40857682	2.11590144	0.00000000
C	5.42422921	0.74565417	0.00000000
C	4.32923077	-0.14291562	0.00000000
C	3.14551148	0.65510711	0.00000000
C	3.56315739	1.98030416	0.00000000
N	4.94270741	2.02063512	0.00000000
O	1.58152733	3.13033575	0.00000000
H	2.12037617	0.31539452	0.00000000
H	5.46305591	2.89151966	0.00000000
H	6.48395500	0.53442658	0.00000000
N	2.82812702	3.19832111	0.00000000
O	3.49062438	4.26821845	0.00000000
C	4.43240264	-1.55496700	0.00000000
C	4.57950259	-2.75873469	0.00000000
C	4.78187676	-4.20245738	0.00000000
C	0.53473572	-3.27812296	0.00000000
C	1.01968104	-4.61555605	0.00000000
C	0.01513827	-5.55388479	0.00000000
S	-1.55498614	-4.80963692	0.00000000
H	1.18405226	-2.40858274	0.00000000
H	4.33507463	-4.66762355	-0.88861707
H	4.33507463	-4.66762355	0.88861707
H	2.07159299	-4.87448543	0.00000000
H	0.09748042	-6.63316155	0.00000000
H	-5.85153141	1.13200129	0.00000000
H	5.85260986	-4.44449928	0.00000000

Cartesian Coordinates and Total Bonding Energies of Ds–Px//A–T variants in the Gas Phase

Cartesian coordinates (Å) and total bonding energies (kcal mol^{-1}) of Ds–Px//A–T variants optimized at ZORA-BLYP-D3(BJ)-TZ2P level of theory in the gas phase.

Ds–Px//A–T

Total bonding energy = -10115.57 kcal mol^{-1}

H	0.72723927	-1.58189205	0.00000000
H	7.16758596	-0.84075983	0.00000000
H	2.84638629	-3.04274385	0.00000000
C	2.69728086	3.10891277	0.00000000
C	2.67338058	4.53073300	0.00000000
C	3.93100000	5.08972217	0.00000000
S	5.17028909	3.87953984	0.00000000
C	3.97910305	2.57918223	0.00000000
H	6.53456193	1.50851733	0.00000000
H	1.82041966	2.47211302	0.00000000
H	1.76132861	5.11988100	0.00000000
H	4.20482529	6.13595262	0.00000000
C	5.73770513	0.77177165	0.00000000
C	6.11128144	-0.58070381	0.00000000
N	5.23907398	-1.61193199	0.00000000
C	3.96653401	-1.21558100	0.00000000
C	3.45639952	0.11251665	0.00000000
C	4.38619688	1.18146471	0.00000000
N	2.83805186	-2.02943647	0.00000000
C	1.73862604	-1.19298086	0.00000000
N	2.06061788	0.08807854	0.00000000
C	10.57742426	5.19624498	0.00000000
C	9.17258342	5.08104223	0.00000000
C	8.87184792	3.68696417	0.00000000
C	10.08491858	3.01557419	0.00000000
N	11.11130473	3.93957267	0.00000000
O	9.41344424	0.82598338	0.00000000
H	7.89338394	3.23779693	0.00000000
H	12.08990976	3.67306907	0.00000000
H	11.19968306	6.07975834	0.00000000
N	10.37561701	1.62277319	0.00000000
O	11.58898294	1.28956516	0.00000000
C	8.23943561	6.14550202	0.00000000
C	7.41903122	7.03842908	0.00000000
C	6.43122222	8.11041610	0.00000000
H	5.78766367	8.05665504	0.88853572
H	6.91678631	9.09475771	0.00000000
H	5.78766367	8.05665504	-0.88853572
N	4.57183255	1.43852731	-3.40000000
C	4.09518869	0.17316015	-3.40000000
N	2.81375783	-0.22237226	-3.40000000
C	1.97355839	0.82939634	-3.40000000
C	2.32350107	2.19056107	-3.40000000
C	3.70811962	2.49046906	-3.40000000

N	0.58784311	0.82575783	-3.40000000
C	0.17956772	2.15477095	-3.40000000
N	1.19140388	3.00209708	-3.40000000
N	4.19663776	3.74563787	-3.40000000
H	4.85747100	-0.60489205	-3.40000000
H	-0.00000000	-0.00000000	-3.40000000
H	-0.86902651	2.42616576	-3.40000000
H	3.54629097	4.51971598	-3.40000000
H	5.21191681	3.91205125	-3.40000000
N	7.36877342	1.77356449	-3.40000000
C	8.11440123	0.60558684	-3.40000000
N	9.49887252	0.83431210	-3.40000000
C	10.06585699	2.09205480	-3.40000000
C	9.31714560	3.22488931	-3.40000000
C	7.85771504	3.08234688	-3.40000000
O	7.07566923	4.05000375	-3.40000000
O	7.63580099	-0.52452810	-3.40000000
C	9.90464120	4.60953621	-3.40000000
H	6.31558431	1.64584283	-3.40000000
H	10.07500663	0.00000000	-3.40000000
H	11.15259232	2.11265461	-3.40000000
H	9.57063820	5.17386146	-4.27918469
H	10.99901520	4.57177634	-3.40000000
H	9.57063820	5.17386146	-2.52081531

Bs–Px//A–T

Total bonding energy = -10218.28 kcal mol^{-1}

H	0.70096590	1.54647536	0.00000000
H	7.27960647	0.73941839	0.00000000
H	2.75968738	3.06258820	0.00000000
C	2.69268629	-3.06879461	0.00000000
C	2.65649099	-4.49183177	0.00000000
C	3.90706797	-5.06334670	0.00000000
S	5.15642431	-3.86052180	0.00000000
C	3.97698268	-2.54658206	0.00000000
H	6.52700582	-1.56385532	0.00000000
H	1.82044153	-2.42670540	0.00000000
H	1.73835234	-5.07187148	0.00000000
H	4.17176954	-6.11183272	0.00000000
C	5.76888296	-0.78683945	0.00000000
C	6.21074586	0.54644436	0.00000000
C	5.30935230	1.61293951	0.00000000
C	3.95449104	1.27218217	0.00000000
C	3.48341600	-0.07111626	0.00000000
C	4.40770456	-1.15107801	0.00000000
N	2.79956450	2.05115332	0.00000000
C	1.72249316	1.18481043	0.00000000
N	2.08405969	-0.08155962	0.00000000
C	10.56533339	-5.22488192	0.00000000
C	9.16219635	-5.09002261	0.00000000
C	8.88057955	-3.69185455	0.00000000
C	10.10366283	-3.03765013	0.00000000

N	11.11708768	-3.97630997	0.00000000
O	9.46936780	-0.83656548	0.00000000
H	7.90831999	-3.22796871	0.00000000
H	12.09907844	-3.72287660	0.00000000
H	11.17465891	-6.11739117	0.00000000
N	10.41699364	-1.65053901	0.00000000
O	11.63664124	-1.33735449	0.00000000
C	8.21535407	-6.14219079	0.00000000
C	7.38377140	-7.02450580	0.00000000
C	6.37995929	-8.08104583	0.00000000
H	5.73692712	-8.01657619	0.88817792
H	5.64930690	2.64545246	0.00000000
H	5.73692712	-8.01657619	-0.88817792
H	6.85025367	-9.07291574	0.00000000
N	4.57183255	-1.43852731	3.40000000
C	4.09518869	-0.17316015	3.40000000
N	2.81375783	0.22237226	3.40000000
C	1.97355839	-0.82939634	3.40000000
C	2.32350107	-2.19056107	3.40000000
C	3.70811962	-2.49046906	3.40000000
N	0.58784311	-0.82575783	3.40000000
C	0.17956772	-2.15477095	3.40000000
N	1.19140388	-3.00209708	3.40000000
N	4.19663776	-3.74563787	3.40000000
H	4.85747100	0.60489205	3.40000000
H	-0.00000000	0.00000000	3.40000000
H	-0.86902651	-2.42616576	3.40000000
H	3.54629097	-4.51971598	3.40000000
H	5.21191681	-3.91205125	3.40000000
N	7.36877342	-1.77356449	3.40000000
C	8.11440123	-0.60558684	3.40000000
N	9.49887252	-0.83431210	3.40000000
C	10.06585699	-2.09205480	3.40000000
C	9.31714560	-3.22488931	3.40000000
C	7.85771504	-3.08234688	3.40000000
O	7.07566923	-4.05000375	3.40000000
O	7.63580099	0.52452810	3.40000000
C	9.90464120	-4.60953621	3.40000000
H	6.31558431	-1.64584283	3.40000000
H	10.07500663	-0.00000000	3.40000000
H	11.15259232	-2.11265461	3.40000000
H	9.57063820	-5.17386146	4.27918469
H	10.99901520	-4.57177634	3.40000000
H	9.57063820	-5.17386146	2.52081531

Is–Px//A–T

Total bonding energy = -10315.20 kcal mol^{-1}

H	0.60474525	1.66290625	0.00000000
H	7.16365718	0.51876637	0.00000000
H	2.78789214	2.93799973	0.00000000
C	2.62191116	-3.34680584	0.00000000
C	2.64723555	-4.77123966	0.00000000

C	3.92064709	-5.28443806	0.00000000
S	5.11089341	-4.02333679	0.00000000
C	3.87609255	-2.75519111	0.00000000
H	6.38621812	-1.77771406	0.00000000
H	1.69905812	-2.78550729	0.00000000
H	1.75382439	-5.38864118	0.00000000
H	4.23480198	-6.31901841	0.00000000
C	5.63577283	-0.99353582	0.00000000
C	6.09362177	0.33302595	0.00000000
C	5.19454539	1.39737061	0.00000000
C	3.83419302	1.07742047	0.00000000
C	3.33334423	-0.26735511	0.00000000
C	4.27265326	-1.34445883	0.00000000
N	2.73826635	1.92869824	0.00000000
C	1.57291277	1.18042906	0.00000000
C	1.89493731	-0.15892178	0.00000000
C	10.53839498	-5.31262909	0.00000000
C	9.13397411	-5.18929849	0.00000000
C	8.84053613	-3.79350621	0.00000000
C	10.05868722	-3.12946768	0.00000000
N	11.07958692	-4.05957095	0.00000000
O	9.40861506	-0.93278189	0.00000000
H	7.86610772	-3.33358129	0.00000000
H	12.05953675	-3.79824439	0.00000000
H	11.15490459	-6.20016046	0.00000000
N	10.36184803	-1.73960528	0.00000000
O	11.57931208	-1.41824921	0.00000000
C	8.20030245	-6.25344931	0.00000000
C	7.38883201	-7.15447712	0.00000000
C	6.41175937	-8.23597386	0.00000000
H	5.53605553	2.43009057	0.00000000
H	5.76732598	-8.18846079	-0.88833290
H	5.76732598	-8.18846079	0.88833290
H	1.16510704	-0.95213950	0.00000000
H	6.90707471	-9.21548711	0.00000000
N	4.57183255	-1.43852731	3.40000000
C	4.09518869	-0.17316015	3.40000000
N	2.81375783	0.22237226	3.40000000
C	1.97355839	-0.82939634	3.40000000
C	2.32350107	-2.19056107	3.40000000
C	3.70811962	-2.49046906	3.40000000
N	0.58784311	-0.82575783	3.40000000
C	0.17956772	-2.15477095	3.40000000
N	1.19140388	-3.00209708	3.40000000
N	4.19663776	-3.74563787	3.40000000
H	4.85747100	0.60489205	3.40000000
H	-0.00000000	0.00000000	3.40000000
H	-0.86902651	-2.42616576	3.40000000
H	3.54629097	-4.51971598	3.40000000
H	5.21191681	-3.91205125	3.40000000
N	7.36877342	-1.77356449	3.40000000
C	8.11440123	-0.60558684	3.40000000

N	9.49887252	-0.83431210	3.40000000
C	10.06585699	-2.09205480	3.40000000
C	9.31714560	-3.22488931	3.40000000
C	7.85771504	-3.08234688	3.40000000
O	7.07566923	-4.05000375	3.40000000
O	7.63580099	0.52452810	3.40000000
C	9.90464120	-4.60953621	3.40000000
H	6.31558431	-1.64584283	3.40000000
H	10.07500663	-0.00000000	3.40000000
H	11.15259232	-2.11265461	3.40000000
H	9.57063820	-5.17386146	4.27918469
H	10.99901520	-4.57177634	3.40000000
H	9.57063820	-5.17386146	2.52081531

Ys–Px//A–T

Total bonding energy = -10213.30 kcal mol^{-1}

H	0.63818449	1.70218235	0.00000000
H	7.07058106	0.74898656	0.00000000
H	2.85452803	2.96137650	0.00000000
C	2.71755539	-3.33320535	0.00000000
C	2.77755442	-4.75490259	0.00000000
C	4.06524956	-5.23493337	0.00000000
S	5.22492047	-3.94917317	0.00000000
C	3.95942088	-2.71598426	0.00000000
H	6.45578738	-1.61066212	0.00000000
H	1.78443692	-2.78797264	0.00000000
H	1.90112590	-5.39576448	0.00000000
H	4.40363650	-6.26151712	0.00000000
C	5.65437255	-0.87887180	0.00000000
C	6.01749567	0.47500534	0.00000000
N	5.13094596	1.48831306	0.00000000
C	3.85716813	1.09233431	0.00000000
C	3.34400447	-0.25000221	0.00000000
C	4.30808119	-1.29697612	0.00000000
N	2.77278892	1.95273229	0.00000000
C	1.60265971	1.21266497	0.00000000
C	1.90963503	-0.13273547	0.00000000
C	10.59880049	-5.28389191	0.00000000
C	9.19543710	-5.15255575	0.00000000
C	8.90957779	-3.75463872	0.00000000
C	10.13122020	-3.09837946	0.00000000
N	11.14668664	-4.03366741	0.00000000
O	9.49519457	-0.89735379	0.00000000
H	7.93634073	-3.29272852	0.00000000
H	12.12769203	-3.77597151	0.00000000
H	11.21055644	-6.17467959	0.00000000
N	10.44340224	-1.70914713	0.00000000
O	11.66263444	-1.39627256	0.00000000
C	8.25934607	-6.21491341	0.00000000
C	7.45639021	-7.12356593	0.00000000
C	6.49384262	-8.21830938	0.00000000
H	5.84925838	-8.18095263	0.88875216

H	1.17127561	-0.91935440	0.00000000
H	5.84925838	-8.18095263	-0.88875216
H	7.00275426	-9.19072997	0.00000000
N	4.57183255	-1.43852731	3.40000000
C	4.09518869	-0.17316015	3.40000000
N	2.81375783	0.22237226	3.40000000
C	1.97355839	-0.82939634	3.40000000
C	2.32350107	-2.19056107	3.40000000
C	3.70811962	-2.49046906	3.40000000
N	0.58784311	-0.82575783	3.40000000
C	0.17956772	-2.15477095	3.40000000
N	1.19140388	-3.00209708	3.40000000
N	4.19663776	-3.74563787	3.40000000
H	4.85747100	0.60489205	3.40000000
H	-0.00000000	0.00000000	3.40000000
H	-0.86902651	-2.42616576	3.40000000
H	3.54629097	-4.51971598	3.40000000
H	5.21191681	-3.91205125	3.40000000
N	7.36877342	-1.77356449	3.40000000
C	8.11440123	-0.60558684	3.40000000
N	9.49887252	-0.83431210	3.40000000
C	10.06585699	-2.09205480	3.40000000
C	9.31714560	-3.22488931	3.40000000
C	7.85771504	-3.08234688	3.40000000
O	7.07566923	-4.05000375	3.40000000
O	7.63580099	0.52452810	3.40000000
C	9.90464120	-4.60953621	3.40000000
H	6.31558431	-1.64584283	3.40000000
H	10.07500663	-0.00000000	3.40000000
H	11.15259232	-2.11265461	3.40000000
H	9.57063820	-5.17386146	4.27918469
H	10.99901520	-4.57177634	3.40000000
H	9.57063820	-5.17386146	2.52081531

Yi–Px//A–T
Total bonding energy = -10299.68 kcal mol^{-1}

H	0.36306980	-1.69073465	0.00000000
H	6.46530076	0.55209666	0.00000000
H	2.78505865	-2.48218108	0.00000000
N	1.33302781	3.45946973	0.00000000
C	1.14109787	4.82245093	0.00000000
C	2.35492263	5.48085208	0.00000000
N	3.31419405	4.48485184	0.00000000
C	2.65680721	3.26401272	0.00000000
H	5.35514429	2.76048351	0.00000000
H	0.38673683	1.03243420	0.00000000
H	0.14912731	5.25596540	0.00000000
H	2.61288317	6.52875262	0.00000000
C	4.73909019	1.86560554	0.00000000
C	5.37774180	0.61452904	0.00000000
N	4.72448910	-0.56319769	0.00000000
C	3.39474292	-0.45019711	0.00000000

C	2.61557441	0.75564757	0.00000000
C	3.33512787	1.97483841	0.00000000
N	2.50651055	-1.50943156	0.00000000
C	1.20687664	-1.01374846	0.00000000
C	1.23498562	0.36508514	0.00000000
C	9.71410795	5.05718749	0.00000000
C	8.35321272	4.68855225	0.00000000
C	8.31008743	3.26252777	0.00000000
C	9.62568637	2.82484287	0.00000000
N	10.46588573	3.91927181	0.00000000
O	9.37529321	0.54740458	0.00000000
H	7.43946428	2.62836396	0.00000000
H	11.47681004	3.83274594	0.00000000
H	10.16373597	6.03973869	0.00000000
N	10.17081600	1.50715040	0.00000000
O	11.42521199	1.41052455	0.00000000
C	7.26554807	5.59690525	0.00000000
C	6.39060498	6.43829997	0.00000000
C	5.38295886	7.49332904	0.00000000
H	4.74163310	7.43635214	-0.88925334
H	4.31803023	4.63482027	0.00000000
H	4.74163310	7.43635214	0.88925334
H	5.86308746	8.48033350	0.00000000
N	4.57183255	1.43852731	-3.40000000
C	4.09518869	0.17316015	-3.40000000
N	2.81375783	-0.22237226	-3.40000000
C	1.97355839	0.82939634	-3.40000000
C	2.32350107	2.19056107	-3.40000000
C	3.70811962	2.49046906	-3.40000000
N	0.58784311	0.82575783	-3.40000000
C	0.17956772	2.15477095	-3.40000000
N	1.19140388	3.00209708	-3.40000000
N	4.19663776	3.74563787	-3.40000000
H	4.85747100	-0.60489205	-3.40000000
H	-0.00000000	-0.00000000	-3.40000000
H	-0.86902651	2.42616576	-3.40000000
H	3.54629097	4.51971598	-3.40000000
H	5.21191681	3.91205125	-3.40000000
N	7.36877342	1.77356449	-3.40000000
C	8.11440123	0.60558684	-3.40000000
N	9.49887252	0.83431210	-3.40000000
C	10.06585699	2.09205480	-3.40000000
C	9.31714560	3.22488931	-3.40000000
C	7.85771504	3.08234688	-3.40000000
O	7.07566923	4.05000375	-3.40000000
O	7.63580099	-0.52452810	-3.40000000
C	9.90464120	4.60953621	-3.40000000
H	6.31558431	1.64584283	-3.40000000
H	10.07500663	0.00000000	-3.40000000
H	11.15259232	2.11265461	-3.40000000
H	9.57063820	5.17386146	-4.27918469
H	10.99901520	4.57177634	-3.40000000

H	9.57063820	5.17386146	-2.52081531

Do–Px//A–T

Total bonding energy = -10172.87 kcal mol^{-1}

H	0.81751879	-1.57978776	0.00000000
H	7.30046418	-1.46491657	0.00000000
H	2.78134252	-3.24199037	0.00000000
C	3.27916109	2.95388005	0.00000000
C	3.64994995	4.33592231	0.00000000
C	5.01728860	4.37671679	0.00000000
O	5.53171277	3.09925077	0.00000000
C	4.44594823	2.22157265	0.00000000
H	6.89412831	0.94693581	0.00000000
H	2.28735790	2.52461794	0.00000000
H	2.98500691	5.18979363	0.00000000
H	5.75077557	5.17007535	0.00000000
C	6.03880204	0.28211185	0.00000000
C	6.27455137	-1.10207400	0.00000000
N	5.30864589	-2.04693291	0.00000000
C	4.07774670	-1.53365373	0.00000000
C	3.69860446	-0.16486874	0.00000000
C	4.72851603	0.80511804	0.00000000
N	2.87413125	-2.23297705	0.00000000
C	1.86209200	-1.29167172	0.00000000
N	2.30858870	-0.04780511	0.00000000
C	10.65437834	5.11218882	0.00000000
C	9.24695597	5.04099781	0.00000000
C	8.89689365	3.65636861	0.00000000
C	10.09192579	2.94934866	0.00000000
N	11.14707352	3.84083906	0.00000000
O	9.38325143	0.76806665	0.00000000
H	7.90201620	3.23608687	0.00000000
H	12.11578060	3.53987587	0.00000000
H	11.30299029	5.97652788	0.00000000
N	10.35623572	1.55049148	0.00000000
O	11.56426150	1.19639532	0.00000000
C	8.36160050	6.14621749	0.00000000
C	7.59588277	7.08705502	0.00000000
C	6.66248257	8.20751831	0.00000000
H	6.01613104	8.18548168	-0.88766262
H	6.01613104	8.18548168	0.88766262
H	7.19777113	9.16567402	0.00000000
N	4.57183255	1.43852731	-3.40000000
C	4.09518869	0.17316015	-3.40000000
N	2.81375783	-0.22237226	-3.40000000
C	1.97355839	0.82939634	-3.40000000
C	2.32350107	2.19056107	-3.40000000
C	3.70811962	2.49046906	-3.40000000
N	0.58784311	0.82575783	-3.40000000
C	0.17956772	2.15477095	-3.40000000
N	1.19140388	3.00209708	-3.40000000
N	4.19663776	3.74563787	-3.40000000

H	4.85747100	-0.60489205	-3.40000000
H	-0.00000000	-0.00000000	-3.40000000
H	-0.86902651	2.42616576	-3.40000000
H	3.54629097	4.51971598	-3.40000000
H	5.21191681	3.91205125	-3.40000000
N	7.36877342	1.77356449	-3.40000000
C	8.11440123	0.60558684	-3.40000000
N	9.49887252	0.83431210	-3.40000000
C	10.06585699	2.09205480	-3.40000000
C	9.31714560	3.22488931	-3.40000000
C	7.85771504	3.08234688	-3.40000000
O	7.07566923	4.05000375	-3.40000000
O	7.63580099	-0.52452810	-3.40000000
C	9.90464120	4.60953621	-3.40000000
H	6.31558431	1.64584283	-3.40000000
H	10.07500663	0.00000000	-3.40000000
H	11.15259232	2.11265461	-3.40000000
H	9.57063820	5.17386146	-4.27918469
H	10.99901520	4.57177634	-3.40000000
H	9.57063820	5.17386146	-2.52081531

Dp–Px//A–T

Total bonding energy = -10374.55 kcal mol^{-1}

H	0.87693204	-1.55715699	0.00000000
H	7.35326871	-1.28062344	0.00000000
H	2.88550758	-3.16333522	0.00000000
C	4.46570481	2.39055562	0.00000000
N	5.55785189	3.19456123	0.00000000
H	6.29202375	8.51289157	0.88741770
H	6.29202375	8.51289157	-0.88741770
N	5.42653866	4.52556614	0.00000000
H	6.88248931	1.12772702	0.00000000
C	4.19727530	5.06708418	0.00000000
C	3.02713193	4.30182542	0.00000000
C	3.15870861	2.92269965	0.00000000
C	6.04944010	0.43691520	0.00000000
C	6.31977438	-0.94113492	0.00000000
N	5.37238030	-1.90391588	0.00000000
C	4.13321294	-1.42007279	0.00000000
C	3.72078170	-0.05589091	0.00000000
C	4.73264835	0.93247941	0.00000000
N	2.94943507	-2.15189184	0.00000000
C	1.91354329	-1.24170608	0.00000000
N	2.32451397	0.01446332	0.00000000
C	10.77248954	5.21594936	0.00000000
C	9.36269675	5.23102076	0.00000000
C	8.92570962	3.87060348	0.00000000
C	10.07856034	3.09220343	0.00000000
N	11.18810467	3.91790022	0.00000000
O	9.26127145	0.94585487	0.00000000
H	7.90195798	3.51788783	0.00000000
H	12.13580788	3.55740135	0.00000000

H	11.47276416	6.03923701	0.00000000
N	10.27018345	1.68465444	0.00000000
O	11.45950118	1.26567184	0.00000000
C	8.55117206	6.39034922	0.00000000
C	7.83587533	7.36816721	0.00000000
C	6.94004029	8.51742456	0.00000000
H	2.04914038	4.77808712	0.00000000
H	2.30444526	2.25387715	0.00000000
H	4.17410762	6.15572023	0.00000000
H	7.49940533	9.46226455	0.00000000
N	4.54678998	1.48057199	-3.40000000
C	4.02627579	0.23261029	-3.40000000
N	2.73182167	-0.11795988	-3.40000000
C	1.92884025	0.96249055	-3.40000000
C	2.32607372	2.31061328	-3.40000000
C	3.72031543	2.56201607	-3.40000000
N	0.54384213	1.00721502	-3.40000000
C	0.18219734	2.34966715	-3.40000000
N	1.22298837	3.16116454	-3.40000000
N	4.25234075	3.79937123	-3.40000000
H	4.76094011	-0.57157120	-3.40000000
H	-0.07246141	0.20247565	-3.40000000
H	-0.85628657	2.65749204	-3.40000000
H	3.62940507	4.59567457	-3.40000000
H	5.27280905	3.93025051	-3.40000000
N	7.35371965	1.71779325	-3.40000000
C	8.05813142	0.52450507	-3.40000000
N	9.44974172	0.70477364	-3.40000000
C	10.06027539	1.94196269	-3.40000000
C	9.35155545	3.10023675	-3.40000000
C	7.88803927	3.00871455	-3.40000000
O	7.14024061	4.00307496	-3.40000000
O	7.54038229	-0.58821853	-3.40000000
C	9.98701664	4.46353687	-3.40000000
H	6.29671470	1.62690516	-3.40000000
H	9.99640779	-0.14913701	-3.40000000
H	11.14706763	1.92462343	-3.40000000
H	9.67291177	5.03917488	-4.27918469
H	11.07940618	4.38760689	-3.40000000
H	9.67291177	5.03917488	-2.52081531

Dss–Px//A–T
Total bonding energy = -11147.73 kcal mol^{-1}

H	1.38103284	1.79574636	0.00000000
H	7.73737104	0.51702955	0.00000000
H	3.61562998	3.07353560	0.00000000
C	2.95587748	-3.03541977	0.00000000
C	2.80059786	-4.43882256	0.00000000
C	3.99895426	-5.14048056	0.00000000
S	5.35541516	-4.02156309	0.00000000
C	4.27955743	-2.61899181	0.00000000
H	6.91028096	-1.77431612	0.00000000

H	2.14049108	-2.32146426	0.00000000
H	1.83385628	-4.93336154	0.00000000
H	3.39920977	-10.04623184	0.00000000
C	6.18047863	-0.97121055	0.00000000
C	6.66367740	0.34603147	0.00000000
N	5.88010969	1.44719142	0.00000000
C	4.57864733	1.15867427	0.00000000
C	3.95927248	-0.12125961	0.00000000
C	4.79905750	-1.26433212	0.00000000
N	3.52198020	2.06460736	0.00000000
C	2.35642927	1.32383467	0.00000000
N	2.57056233	0.01999405	0.00000000
C	11.70541466	-5.42994712	0.00000000
C	10.32752048	-5.72919805	0.00000000
C	9.63042931	-4.48361791	0.00000000
C	10.60062177	-3.48874067	0.00000000
N	11.85040355	-4.07489072	0.00000000
O	9.34985312	-1.56965066	0.00000000
H	8.56139326	-4.33049589	0.00000000
H	12.70736297	-3.53199507	0.00000000
H	12.55612832	-6.09626913	0.00000000
N	10.49380860	-2.07083341	0.00000000
O	11.56697197	-1.41379803	0.00000000
C	9.77812109	-7.03360638	0.00000000
C	9.35195291	-8.16889332	0.00000000
C	8.85958200	-9.54059755	0.00000000
C	4.20199642	-6.56874919	0.00000000
C	5.37987025	-7.30179023	0.00000000
C	5.17026732	-8.70975613	0.00000000
C	3.84072652	-9.05794705	0.00000000
S	2.81389496	-7.65790760	0.00000000
H	8.24651442	-9.74206914	-0.88822616
H	8.24651442	-9.74206914	0.88822616
H	6.36401311	-6.84318441	0.00000000
H	5.97216763	-9.43904919	0.00000000
H	9.69347953	-10.25429326	0.00000000
N	4.57183255	-1.43852731	3.40000000
C	4.09518869	-0.17316015	3.40000000
N	2.81375783	0.22237226	3.40000000
C	1.97355839	-0.82939634	3.40000000
C	2.32350107	-2.19056107	3.40000000
C	3.70811962	-2.49046906	3.40000000
N	0.58784311	-0.82575783	3.40000000
C	0.17956772	-2.15477095	3.40000000
N	1.19140388	-3.00209708	3.40000000
N	4.19663776	-3.74563787	3.40000000
H	4.85747100	0.60489205	3.40000000
H	-0.00000000	0.00000000	3.40000000
H	-0.86902651	-2.42616576	3.40000000
H	3.54629097	-4.51971598	3.40000000
H	5.21191681	-3.91205125	3.40000000
N	7.36877342	-1.77356449	3.40000000

C	8.11440123	-0.60558684	3.40000000
N	9.49887252	-0.83431210	3.40000000
C	10.06585699	-2.09205480	3.40000000
C	9.31714560	-3.22488931	3.40000000
C	7.85771504	-3.08234688	3.40000000
O	7.07566923	-4.05000375	3.40000000
O	7.63580099	0.52452810	3.40000000
C	9.90464120	-4.60953621	3.40000000
H	6.31558431	-1.64584283	3.40000000
H	10.07500663	-0.00000000	3.40000000
H	11.15259232	-2.11265461	3.40000000
H	9.57063820	-5.17386146	4.27918469
H	10.99901520	-4.57177634	3.40000000
H	9.57063820	-5.17386146	2.52081531

Dt–Px//A–T

Total bonding energy = -10009.70 kcal mol^{-1}

H	0.68964348	1.66555388	0.00000000
H	7.11777631	0.83668213	0.00000000
H	2.83397866	3.09459045	0.00000000
N	2.68316697	-3.01312336	0.00000000
C	2.64630769	-4.38420711	0.00000000
C	3.86599024	-5.01812392	0.00000000
S	5.14534723	-3.85630831	0.00000000
C	3.91345181	-2.55326359	0.00000000
H	6.45243477	-1.50574315	0.00000000
H	5.75860710	-7.99327872	-0.88868877
H	1.68585927	-4.89042967	0.00000000
H	4.07448619	-6.07937681	0.00000000
C	5.66634207	-0.75749215	0.00000000
C	6.05894576	0.58830291	0.00000000
N	5.19567236	1.62647077	0.00000000
C	3.91898351	1.24923014	0.00000000
C	3.38273662	-0.07587787	0.00000000
C	4.30968415	-1.14609727	0.00000000
N	2.80744000	2.08150976	0.00000000
C	1.69449891	1.25915867	0.00000000
N	1.99129851	-0.02633152	0.00000000
C	10.55678003	-5.14023881	0.00000000
C	9.15158663	-5.03024018	0.00000000
C	8.84617980	-3.63708023	0.00000000
C	10.05653889	-2.96128983	0.00000000
N	11.08609278	-3.88161047	0.00000000
O	9.37403803	-0.77544087	0.00000000
H	7.86635234	-3.19081577	0.00000000
H	12.06370081	-3.61119522	0.00000000
H	11.18230042	-6.02143288	0.00000000
N	10.34062374	-1.56715529	0.00000000
O	11.55201340	-1.22799049	0.00000000
C	8.22028565	-6.09633130	0.00000000
C	7.39629280	-6.98588430	0.00000000
C	6.40162787	-8.05141932	0.00000000

H	5.75860710	-7.99327872	0.88868877
H	6.88007958	-9.03917337	0.00000000
N	4.57183255	-1.43852731	3.40000000
C	4.09518869	-0.17316015	3.40000000
N	2.81375783	0.22237226	3.40000000
C	1.97355839	-0.82939634	3.40000000
C	2.32350107	-2.19056107	3.40000000
C	3.70811962	-2.49046906	3.40000000
N	0.58784311	-0.82575783	3.40000000
C	0.17956772	-2.15477095	3.40000000
N	1.19140388	-3.00209708	3.40000000
N	4.19663776	-3.74563787	3.40000000
H	4.85747100	0.60489205	3.40000000
H	-0.00000000	0.00000000	3.40000000
H	-0.86902651	-2.42616576	3.40000000
H	3.54629097	-4.51971598	3.40000000
H	5.21191681	-3.91205125	3.40000000
N	7.36877342	-1.77356449	3.40000000
C	8.11440123	-0.60558684	3.40000000
N	9.49887252	-0.83431210	3.40000000
C	10.06585699	-2.09205480	3.40000000
C	9.31714560	-3.22488931	3.40000000
C	7.85771504	-3.08234688	3.40000000
O	7.07566923	-4.05000375	3.40000000
O	7.63580099	0.52452810	3.40000000
C	9.90464120	-4.60953621	3.40000000
H	6.31558431	-1.64584283	3.40000000
H	10.07500663	-0.00000000	3.40000000
H	11.15259232	-2.11265461	3.40000000
H	9.57063820	-5.17386146	4.27918469
H	10.99901520	-4.57177634	3.40000000
H	9.57063820	-5.17386146	2.52081531

Yo–Px//A–T

Total bonding energy = -10271.31 kcal mol^{-1}

H	0.71245644	-1.68020171	0.00000000
H	7.20482042	-1.27824905	0.00000000
H	2.80906033	-3.12907408	0.00000000
C	3.24726602	3.19796420	0.00000000
C	3.67360706	4.56462704	0.00000000
C	5.03969017	4.54692025	0.00000000
O	5.49608431	3.24785844	0.00000000
C	4.37890555	2.41018253	0.00000000
H	6.79161992	1.13498340	0.00000000
H	2.22685109	2.84759884	0.00000000
H	3.04323986	5.44420830	0.00000000
H	5.80602805	5.30866229	0.00000000
C	5.93855444	0.46785088	0.00000000
C	6.17899735	-0.91479068	0.00000000
N	5.21198114	-1.85257766	0.00000000
C	3.97363982	-1.35463903	0.00000000
C	3.57800675	0.02344357	0.00000000

C	4.62710438	0.98163244	0.00000000
N	2.81732097	-2.11721811	0.00000000
C	1.71588775	-1.27648774	0.00000000
C	2.14041671	0.03725822	0.00000000
C	10.65729348	5.21316515	0.00000000
C	9.24881147	5.16986303	0.00000000
C	8.87134395	3.79225140	0.00000000
C	10.05189827	3.06204855	0.00000000
N	11.12458649	3.93208221	0.00000000
O	9.29639164	0.89753466	0.00000000
H	7.86951456	3.38888318	0.00000000
H	12.08709602	3.61181343	0.00000000
H	11.32302702	6.06438573	0.00000000
N	10.28627468	1.65755297	0.00000000
O	11.48662095	1.27806628	0.00000000
C	8.38496233	6.29203164	0.00000000
C	7.63607232	7.24615561	0.00000000
C	6.72300561	8.38321718	0.00000000
H	6.07624024	8.37295350	0.88763111
H	6.07624024	8.37295350	-0.88763111
H	1.48077421	0.89199938	0.00000000
H	7.27583009	9.33142125	0.00000000
N	4.57183255	1.43852731	-3.40000000
C	4.09518869	0.17316015	-3.40000000
N	2.81375783	-0.22237226	-3.40000000
C	1.97355839	0.82939634	-3.40000000
C	2.32350107	2.19056107	-3.40000000
C	3.70811962	2.49046906	-3.40000000
N	0.58784311	0.82575783	-3.40000000
C	0.17956772	2.15477095	-3.40000000
N	1.19140388	3.00209708	-3.40000000
N	4.19663776	3.74563787	-3.40000000
H	4.85747100	-0.60489205	-3.40000000
H	-0.00000000	-0.00000000	-3.40000000
H	-0.86902651	2.42616576	-3.40000000
H	3.54629097	4.51971598	-3.40000000
H	5.21191681	3.91205125	-3.40000000
N	7.36877342	1.77356449	-3.40000000
C	8.11440123	0.60558684	-3.40000000
N	9.49887252	0.83431210	-3.40000000
C	10.06585699	2.09205480	-3.40000000
C	9.31714560	3.22488931	-3.40000000
C	7.85771504	3.08234688	-3.40000000
O	7.07566923	4.05000375	-3.40000000
O	7.63580099	-0.52452810	-3.40000000
C	9.90464120	4.60953621	-3.40000000
H	6.31558431	1.64584283	-3.40000000
H	10.07500663	0.00000000	-3.40000000
H	11.15259232	2.11265461	-3.40000000
H	9.57063820	5.17386146	-4.27918469
H	10.99901520	4.57177634	-3.40000000
H	9.57063820	5.17386146	-2.52081531

Ps–Px//A–T

Total bonding energy = -10014.94 kcal mol^{-1}

H	0.58247821	1.54761686	0.00000000
H	6.83318049	-0.12052091	0.00000000
H	2.87041168	2.72963660	0.00000000
C	2.07671232	-3.43091673	0.00000000
C	1.98589757	-4.84759061	0.00000000
C	3.22295524	-5.45879802	0.00000000
S	4.52459024	-4.31641102	0.00000000
C	3.38894984	-2.97861921	0.00000000
H	5.33347025	-8.00695437	-0.88867146
H	1.23202695	-2.75137036	0.00000000
H	1.04839390	-5.39531428	0.00000000
H	3.43798164	-6.51915277	0.00000000
N	5.24265504	-1.44139703	0.00000000
C	5.74918842	-0.19385876	0.00000000
N	5.06552977	0.97086786	0.00000000
C	3.74551380	0.77499023	0.00000000
C	3.07351743	-0.47269809	0.00000000
C	3.89371171	-1.62243943	0.00000000
N	2.73642670	1.72480362	0.00000000
C	1.53466428	1.03050442	0.00000000
N	1.69040275	-0.27917470	0.00000000
C	9.99956345	-5.05568179	0.00000000
C	8.59954606	-4.89086403	0.00000000
C	8.33954054	-3.48436264	0.00000000
C	9.58394456	-2.86184680	0.00000000
N	10.57680394	-3.82241090	0.00000000
O	9.06147030	-0.62476226	0.00000000
H	7.37612702	-2.98872885	0.00000000
H	11.56309523	-3.58596768	0.00000000
H	10.58749415	-5.96245136	0.00000000
N	9.96169260	-1.48964127	0.00000000
O	11.19594424	-1.23653595	0.00000000
C	7.66970870	-5.95894072	0.00000000
C	6.90725534	-6.90151843	0.00000000
C	5.97897178	-8.02530476	0.00000000
H	5.33347025	-8.00695437	0.88867146
H	6.51704559	-8.98200191	0.00000000
N	4.57183255	-1.43852731	3.40000000
C	4.09518869	-0.17316015	3.40000000
N	2.81375783	0.22237226	3.40000000
C	1.97355839	-0.82939634	3.40000000
C	2.32350107	-2.19056107	3.40000000
C	3.70811962	-2.49046906	3.40000000
N	0.58784311	-0.82575783	3.40000000
C	0.17956772	-2.15477095	3.40000000
N	1.19140388	-3.00209708	3.40000000
N	4.19663776	-3.74563787	3.40000000
H	4.85747100	0.60489205	3.40000000
H	-0.00000000	0.00000000	3.40000000
H	-0.86902651	-2.42616576	3.40000000

H	3.54629097	-4.51971598	3.40000000
H	5.21191681	-3.91205125	3.40000000
N	7.36877342	-1.77356449	3.40000000
C	8.11440123	-0.60558684	3.40000000
N	9.49887252	-0.83431210	3.40000000
C	10.06585699	-2.09205480	3.40000000
C	9.31714560	-3.22488931	3.40000000
C	7.85771504	-3.08234688	3.40000000
O	7.07566923	-4.05000375	3.40000000
O	7.63580099	0.52452810	3.40000000
C	9.90464120	-4.60953621	3.40000000
H	6.31558431	-1.64584283	3.40000000
H	10.07500663	-0.00000000	3.40000000
H	11.15259232	-2.11265461	3.40000000
H	9.57063820	-5.17386146	4.27918469
H	10.99901520	-4.57177634	3.40000000
H	9.57063820	-5.17386146	2.52081531

Es–Px//A–T

Total bonding energy = -10114.33 kcal mol^{-1}

H	0.52169509	1.65228178	0.00000000
H	6.74363717	-0.21117396	0.00000000
H	2.87770056	2.63086532	0.00000000
C	2.08956934	-3.65897610	0.00000000
C	2.08349855	-5.07984305	0.00000000
C	3.35468005	-5.61253096	0.00000000
S	4.58070970	-4.39019239	0.00000000
C	3.36720314	-3.11927879	0.00000000
H	0.73059774	-1.02352759	0.00000000
H	1.18697452	-3.06153047	0.00000000
H	1.18034971	-5.68255898	0.00000000
H	3.63380060	-6.65737236	0.00000000
N	5.16632555	-1.55003804	0.00000000
C	5.66073281	-0.29748536	0.00000000
N	4.95858567	0.85060995	0.00000000
C	3.63802909	0.65339293	0.00000000
C	2.96512966	-0.60986369	0.00000000
C	3.82095548	-1.73844678	0.00000000
N	2.67468428	1.63900584	0.00000000
C	1.41671800	1.04531336	0.00000000
C	1.55442746	-0.32582929	0.00000000
C	9.96530733	-5.13093780	0.00000000
C	8.56317556	-4.98232117	0.00000000
C	8.28665062	-3.57916819	0.00000000
C	9.52427941	-2.94251960	0.00000000
N	10.52812676	-3.89163742	0.00000000
O	8.98031381	-0.71070916	0.00000000
H	7.31921321	-3.09141171	0.00000000
H	11.51116542	-3.64171406	0.00000000
H	10.56232591	-6.03171705	0.00000000
N	9.88826316	-1.56644351	0.00000000
O	11.12062020	-1.30194282	0.00000000

C	7.65276083	-6.06753193	0.00000000
C	6.92549321	-7.03817322	0.00000000
C	6.04816569	-8.20265074	0.00000000
H	5.40301332	-8.21542461	0.88882449
H	5.40301332	-8.21542461	-0.88882449
H	6.63086560	-9.13303380	0.00000000
N	4.57183255	-1.43852731	3.40000000
C	4.09518869	-0.17316015	3.40000000
N	2.81375783	0.22237226	3.40000000
C	1.97355839	-0.82939634	3.40000000
C	2.32350107	-2.19056107	3.40000000
C	3.70811962	-2.49046906	3.40000000
N	0.58784311	-0.82575783	3.40000000
C	0.17956772	-2.15477095	3.40000000
N	1.19140388	-3.00209708	3.40000000
N	4.19663776	-3.74563787	3.40000000
H	4.85747100	0.60489205	3.40000000
H	-0.00000000	0.00000000	3.40000000
H	-0.86902651	-2.42616576	3.40000000
H	3.54629097	-4.51971598	3.40000000
H	5.21191681	-3.91205125	3.40000000
N	7.36877342	-1.77356449	3.40000000
C	8.11440123	-0.60558684	3.40000000
N	9.49887252	-0.83431210	3.40000000
C	10.06585699	-2.09205480	3.40000000
C	9.31714560	-3.22488931	3.40000000
C	7.85771504	-3.08234688	3.40000000
O	7.07566923	-4.05000375	3.40000000
O	7.63580099	0.52452810	3.40000000
C	9.90464120	-4.60953621	3.40000000
H	6.31558431	-1.64584283	3.40000000
H	10.07500663	-0.00000000	3.40000000
H	11.15259232	-2.11265461	3.40000000
H	9.57063820	-5.17386146	4.27918469
H	10.99901520	-4.57177634	3.40000000
H	9.57063820	-5.17386146	2.52081531

Ysm–Px//A–T
Total bonding energy = -10582.73 kcal mol^{-1}

H	0.95531385	-1.83089127	0.00000000
H	7.40550545	-1.00094410	0.00000000
H	3.14687447	-3.13380383	0.00000000
C	3.14028662	3.16976569	0.00000000
C	3.23835261	4.59040519	0.00000000
C	4.53216613	5.05889785	0.00000000
S	5.65660829	3.72316791	0.00000000
C	4.36151365	2.51795215	0.00000000
H	6.83976954	1.37018048	0.00000000
H	2.19215956	2.65003879	0.00000000
H	2.37660535	5.25243123	0.00000000
C	5.02135510	6.47717626	0.00000000
C	6.02330251	0.65445124	0.00000000

C	6.35760466	-0.70689080	0.00000000
N	5.45311474	-1.70475653	0.00000000
C	4.18653084	-1.28493526	0.00000000
C	3.69995776	0.06638170	0.00000000
C	4.68365261	1.09496687	0.00000000
N	3.08487535	-2.12383509	0.00000000
C	1.92922388	-1.36040555	0.00000000
C	2.26363250	-0.02139433	0.00000000
C	11.30102134	5.44470739	0.00000000
C	9.89621195	5.55433980	0.00000000
C	9.37473481	4.22573385	0.00000000
C	10.46707157	3.37073212	0.00000000
N	11.62757821	4.11945506	0.00000000
O	9.46787595	1.30808365	0.00000000
H	8.33574929	3.93627692	0.00000000
H	12.55027230	3.69816460	0.00000000
H	12.05600301	6.21791157	0.00000000
N	10.53937396	1.94906868	0.00000000
O	11.68801925	1.43440554	0.00000000
C	9.14868898	6.75680164	0.00000000
C	8.49274578	7.77686617	0.00000000
C	7.69861909	8.99974415	0.00000000
H	7.05337523	9.05364626	-0.88677215
H	5.63737914	6.69335725	0.88172128
H	4.16759865	7.16300455	0.00000000
H	5.63737914	6.69335725	-0.88172128
H	1.54210985	0.78093609	0.00000000
H	7.05337523	9.05364626	0.88677215
H	8.34276138	9.88868087	0.00000000
N	4.57183255	1.43852731	-3.40000000
C	4.09518869	0.17316015	-3.40000000
N	2.81375783	-0.22237226	-3.40000000
C	1.97355839	0.82939634	-3.40000000
C	2.32350107	2.19056107	-3.40000000
C	3.70811962	2.49046906	-3.40000000
N	0.58784311	0.82575783	-3.40000000
C	0.17956772	2.15477095	-3.40000000
N	1.19140388	3.00209708	-3.40000000
N	4.19663776	3.74563787	-3.40000000
H	4.85747100	-0.60489205	-3.40000000
H	-0.00000000	-0.00000000	-3.40000000
H	-0.86902651	2.42616576	-3.40000000
H	3.54629097	4.51971598	-3.40000000
H	5.21191681	3.91205125	-3.40000000
N	7.36877342	1.77356449	-3.40000000
C	8.11440123	0.60558684	-3.40000000
N	9.49887252	0.83431210	-3.40000000
C	10.06585699	2.09205480	-3.40000000
C	9.31714560	3.22488931	-3.40000000
C	7.85771504	3.08234688	-3.40000000
O	7.07566923	4.05000375	-3.40000000
O	7.63580099	-0.52452810	-3.40000000

C	9.90464120	4.60953621	-3.40000000
H	6.31558431	1.64584283	-3.40000000
H	10.07500663	0.00000000	-3.40000000
H	11.15259232	2.11265461	-3.40000000
H	9.57063820	5.17386146	-4.27918469
H	10.99901520	4.57177634	-3.40000000
H	9.57063820	5.17386146	-2.52081531

Yss–Px//A–T

Total bonding energy = -11245.03 kcal mol^{-1}

H	1.30414896	2.00206711	0.00000000
H	7.64502110	0.55388171	0.00000000
H	3.61169995	3.08657777	0.00000000
C	2.99900014	-3.17846434	0.00000000
C	2.94468439	-4.59099767	0.00000000
C	4.19072950	-5.19996728	0.00000000
S	5.45612920	-3.98114568	0.00000000
C	4.28399701	-2.65497477	0.00000000
H	6.85266595	-1.75073172	0.00000000
H	2.11157652	-2.56149037	0.00000000
H	2.01625990	-5.15414334	0.00000000
C	4.50841363	-6.60691755	0.00000000
C	6.11063895	-0.95926759	0.00000000
C	6.57394746	0.36332679	0.00000000
N	5.76769599	1.44262111	0.00000000
C	4.46699134	1.14557766	0.00000000
C	3.85210464	-0.15266113	0.00000000
C	4.73554629	-1.27057353	0.00000000
N	3.45177045	2.08740086	0.00000000
C	2.22819065	1.43998500	0.00000000
C	2.43137102	0.07451737	0.00000000
C	11.83014337	-5.48339071	0.00000000
C	10.47238558	-5.86427340	0.00000000
C	9.70271568	-4.66192575	0.00000000
C	10.61210249	-3.61140638	0.00000000
N	11.89430981	-4.12202289	0.00000000
O	9.24981863	-1.76923021	0.00000000
H	8.62695793	-4.56678213	0.00000000
H	12.71761416	-3.52925616	0.00000000
H	12.71894524	-6.09793695	0.00000000
N	10.42097752	-2.20168791	0.00000000
O	11.45375206	-1.48268872	0.00000000
C	10.00654465	-7.20125724	0.00000000
C	9.66370381	-8.36450923	0.00000000
C	9.27634167	-9.76994194	0.00000000
C	5.74510179	-7.23558874	0.00000000
C	5.65928624	-8.65563625	0.00000000
C	4.36479911	-9.11809716	0.00000000
S	3.21912686	-7.81171642	0.00000000
H	6.68624369	-6.69525173	0.00000000
H	8.68159680	-10.01952208	-0.88861707
H	8.68159680	-10.01952208	0.88861707

H	6.52198034	-9.31086298	0.00000000
H	4.01193998	-10.14138059	0.00000000
H	1.63423810	-0.65270158	0.00000000
H	10.16301420	-10.41714011	0.00000000
N	4.57183255	-1.43852731	3.40000000
C	4.09518869	-0.17316015	3.40000000
N	2.81375783	0.22237226	3.40000000
C	1.97355839	-0.82939634	3.40000000
C	2.32350107	-2.19056107	3.40000000
C	3.70811962	-2.49046906	3.40000000
N	0.58784311	-0.82575783	3.40000000
C	0.17956772	-2.15477095	3.40000000
N	1.19140388	-3.00209708	3.40000000
N	4.19663776	-3.74563787	3.40000000
H	4.85747100	0.60489205	3.40000000
H	-0.00000000	0.00000000	3.40000000
H	-0.86902651	-2.42616576	3.40000000
H	3.54629097	-4.51971598	3.40000000
H	5.21191681	-3.91205125	3.40000000
N	7.36877342	-1.77356449	3.40000000
C	8.11440123	-0.60558684	3.40000000
N	9.49887252	-0.83431210	3.40000000
C	10.06585699	-2.09205480	3.40000000
C	9.31714560	-3.22488931	3.40000000
C	7.85771504	-3.08234688	3.40000000
O	7.07566923	-4.05000375	3.40000000
O	7.63580099	0.52452810	3.40000000
C	9.90464120	-4.60953621	3.40000000
H	6.31558431	-1.64584283	3.40000000
H	10.07500663	-0.00000000	3.40000000
H	11.15259232	-2.11265461	3.40000000
H	9.57063820	-5.17386146	4.27918469
H	10.99901520	-4.57177634	3.40000000
H	9.57063820	-5.17386146	2.52081531

Cartesian Coordinates and Total Bonding Energies of Ds–Px//G–C variants in the Gas Phase

Cartesian coordinates (Å) and total bonding energies (kcal mol^{-1}) of Ds–Px//G–C variants optimized at ZORA-BLYP-D3(BJ)-TZ2P level of theory in the gas phase.

Ds–Px//G–C
Total bonding energy = -10021.52 kcal mol^{-1}

H	0.72723927	-1.58189205	0.00000000
H	7.16758596	-0.84075983	0.00000000
H	2.84638629	-3.04274385	0.00000000
C	2.69728086	3.10891277	0.00000000
C	2.67338058	4.53073300	0.00000000
C	3.93100000	5.08972217	0.00000000
S	5.17028909	3.87953984	0.00000000
C	3.97910305	2.57918223	0.00000000
H	6.53456193	1.50851733	0.00000000
H	1.82041966	2.47211302	0.00000000
H	1.76132861	5.11988100	0.00000000
H	4.20482529	6.13595262	0.00000000
C	5.73770513	0.77177165	0.00000000
C	6.11128144	-0.58070381	0.00000000
N	5.23907398	-1.61193199	0.00000000
C	3.96653401	-1.21558100	0.00000000
C	3.45639952	0.11251665	0.00000000
C	4.38619688	1.18146471	0.00000000
N	2.83805186	-2.02943647	0.00000000
C	1.73862604	-1.19298086	0.00000000
N	2.06061788	0.08807854	0.00000000
C	10.57742426	5.19624498	0.00000000
C	9.17258342	5.08104223	0.00000000
C	8.87184792	3.68696417	0.00000000
C	10.08491858	3.01557419	0.00000000
N	11.11130473	3.93957267	0.00000000
O	9.41344424	0.82598338	0.00000000
H	7.89338394	3.23779693	0.00000000
H	12.08990976	3.67306907	0.00000000
H	11.19968306	6.07975834	0.00000000
N	10.37561701	1.62277319	0.00000000
O	11.58898294	1.28956516	0.00000000
C	8.23943561	6.14550202	0.00000000
C	7.41903122	7.03842908	0.00000000
C	6.43122222	8.11041610	0.00000000
H	5.78766367	8.05665504	0.88853572
H	6.91678631	9.09475771	0.00000000
H	5.78766367	8.05665504	-0.88853572
N	4.97119005	-1.05533672	-3.40000000
O	4.34528524	3.53812458	-3.40000000
N	4.55667268	1.23807420	-3.40000000
C	4.06244004	-0.05043940	-3.40000000
N	2.75810350	-0.33063542	-3.40000000
C	1.98630422	0.77789430	-3.40000000

C	2.37868353	2.13086291	-3.40000000
C	3.77981851	2.42464605	-3.40000000
N	0.60593208	0.81231267	-3.40000000
C	0.23111076	2.15846856	-3.40000000
N	1.26559359	2.96965052	-3.40000000
H	5.98534901	-0.89040548	-3.40000000
H	4.60944716	-1.99848779	-3.40000000
H	5.58898402	1.37787815	-3.40000000
H	-0.00000000	0.00000000	-3.40000000
H	-0.81025242	2.45627290	-3.40000000
O	7.83318423	-0.57821431	-3.40000000
N	7.07225635	3.96489646	-3.40000000
N	7.42685680	1.69230122	-3.40000000
C	9.36213639	3.17387907	-3.40000000
C	8.23575027	0.59704963	-3.40000000
N	9.63845619	0.82879603	-3.40000000
C	10.17549178	2.08385156	-3.40000000
C	7.93637707	2.93939270	-3.40000000
H	9.77098431	4.17827316	-3.40000000
H	10.22420135	-0.00000000	-3.40000000
H	11.25945255	2.15100901	-3.40000000
H	6.03848799	3.79721523	-3.40000000
H	7.42143441	4.91346797	-3.40000000

Bs–Px//G–C

Total bonding energy = -10123.34 kcal mol^{-1}

H	0.70096590	1.54647536	0.00000000
H	7.27960647	0.73941839	0.00000000
H	2.75968738	3.06258820	0.00000000
C	2.69268629	-3.06879461	0.00000000
C	2.65649099	-4.49183177	0.00000000
C	3.90706797	-5.06334670	0.00000000
S	5.15642431	-3.86052180	0.00000000
C	3.97698268	-2.54658206	0.00000000
H	6.52700582	-1.56385532	0.00000000
H	1.82044153	-2.42670540	0.00000000
H	1.73835234	-5.07187148	0.00000000
H	4.17176954	-6.11183272	0.00000000
C	5.76888296	-0.78683945	0.00000000
C	6.21074586	0.54644436	0.00000000
C	5.30935230	1.61293951	0.00000000
C	3.95449104	1.27218217	0.00000000
C	3.48341600	-0.07111626	0.00000000
C	4.40770456	-1.15107801	0.00000000
N	2.79956450	2.05115332	0.00000000
C	1.72249316	1.18481043	0.00000000
N	2.08405969	-0.08155962	0.00000000
C	10.56533339	-5.22488192	0.00000000
C	9.16219635	-5.09002261	0.00000000
C	8.88057955	-3.69185455	0.00000000
C	10.10366283	-3.03765013	0.00000000
N	11.11708768	-3.97630997	0.00000000

O	9.46936780	-0.83656548	0.00000000
H	7.90831999	-3.22796871	0.00000000
H	12.09907844	-3.72287660	0.00000000
H	11.17465891	-6.11739117	0.00000000
N	10.41699364	-1.65053901	0.00000000
O	11.63664124	-1.33735449	0.00000000
C	8.21535407	-6.14219079	0.00000000
C	7.38377140	-7.02450580	0.00000000
C	6.37995929	-8.08104583	0.00000000
H	5.73692712	-8.01657619	0.88817792
H	5.64930690	2.64545246	0.00000000
H	5.73692712	-8.01657619	-0.88817792
H	6.85025367	-9.07291574	0.00000000
N	4.97119005	1.05533672	3.40000000
O	4.34528524	-3.53812458	3.40000000
N	4.55667268	-1.23807420	3.40000000
C	4.06244004	0.05043940	3.40000000
N	2.75810350	0.33063542	3.40000000
C	1.98630422	-0.77789430	3.40000000
C	2.37868353	-2.13086291	3.40000000
C	3.77981851	-2.42464605	3.40000000
N	0.60593208	-0.81231267	3.40000000
C	0.23111076	-2.15846856	3.40000000
N	1.26559359	-2.96965052	3.40000000
H	5.98534901	0.89040548	3.40000000
H	4.60944716	1.99848779	3.40000000
H	5.58898402	-1.37787815	3.40000000
H	-0.00000000	-0.00000000	3.40000000
H	-0.81025242	-2.45627290	3.40000000
O	7.83318423	0.57821431	3.40000000
N	7.07225635	-3.96489646	3.40000000
N	7.42685680	-1.69230122	3.40000000
C	9.36213639	-3.17387907	3.40000000
C	8.23575027	-0.59704963	3.40000000
N	9.63845619	-0.82879603	3.40000000
C	10.17549178	-2.08385156	3.40000000
C	7.93637707	-2.93939270	3.40000000
H	9.77098431	-4.17827316	3.40000000
H	10.22420135	0.00000000	3.40000000
H	11.25945255	-2.15100901	3.40000000
H	6.03848799	-3.79721523	3.40000000
H	7.42143441	-4.91346797	3.40000000

Is–Px//G–C

Total bonding energy = -10220.45 kcal mol^{-1}

H	0.60474525	1.66290625	0.00000000
H	7.16365718	0.51876637	0.00000000
H	2.78789214	2.93799973	0.00000000
C	2.62191116	-3.34680584	0.00000000
C	2.64723555	-4.77123966	0.00000000
C	3.92064709	-5.28443806	0.00000000
S	5.11089341	-4.02333679	0.00000000

C	3.87609255	-2.75519111	0.00000000
H	6.38621812	-1.77771406	0.00000000
H	1.69905812	-2.78550729	0.00000000
H	1.75382439	-5.38864118	0.00000000
H	4.23480198	-6.31901841	0.00000000
C	5.63577283	-0.99353582	0.00000000
C	6.09362177	0.33302595	0.00000000
C	5.19454539	1.39737061	0.00000000
C	3.83419302	1.07742047	0.00000000
C	3.33334423	-0.26735511	0.00000000
C	4.27265326	-1.34445883	0.00000000
N	2.73826635	1.92869824	0.00000000
C	1.57291277	1.18042906	0.00000000
C	1.89493731	-0.15892178	0.00000000
C	10.53839498	-5.31262909	0.00000000
C	9.13397411	-5.18929849	0.00000000
C	8.84053613	-3.79350621	0.00000000
C	10.05868722	-3.12946768	0.00000000
N	11.07958692	-4.05957095	0.00000000
O	9.40861506	-0.93278189	0.00000000
H	7.86610772	-3.33358129	0.00000000
H	12.05953675	-3.79824439	0.00000000
H	11.15490459	-6.20016046	0.00000000
N	10.36184803	-1.73960528	0.00000000
O	11.57931208	-1.41824921	0.00000000
C	8.20030245	-6.25344931	0.00000000
C	7.38883201	-7.15447712	0.00000000
C	6.41175937	-8.23597386	0.00000000
H	5.53605553	2.43009057	0.00000000
H	5.76732598	-8.18846079	-0.88833290
H	5.76732598	-8.18846079	0.88833290
H	1.16510704	-0.95213950	0.00000000
H	6.90707471	-9.21548711	0.00000000
N	4.97119005	1.05533672	3.40000000
O	4.34528524	-3.53812458	3.40000000
N	4.55667268	-1.23807420	3.40000000
C	4.06244004	0.05043940	3.40000000
N	2.75810350	0.33063542	3.40000000
C	1.98630422	-0.77789430	3.40000000
C	2.37868353	-2.13086291	3.40000000
C	3.77981851	-2.42464605	3.40000000
N	0.60593208	-0.81231267	3.40000000
C	0.23111076	-2.15846856	3.40000000
N	1.26559359	-2.96965052	3.40000000
H	5.98534901	0.89040548	3.40000000
H	4.60944716	1.99848779	3.40000000
H	5.58898402	-1.37787815	3.40000000
H	-0.00000000	-0.00000000	3.40000000
H	-0.81025242	-2.45627290	3.40000000
O	7.83318423	0.57821431	3.40000000
N	7.07225635	-3.96489646	3.40000000
N	7.42685680	-1.69230122	3.40000000

C	9.36213639	-3.17387907	3.40000000
C	8.23575027	-0.59704963	3.40000000
N	9.63845619	-0.82879603	3.40000000
C	10.17549178	-2.08385156	3.40000000
C	7.93637707	-2.93939270	3.40000000
H	9.77098431	-4.17827316	3.40000000
H	10.22420135	0.00000000	3.40000000
H	11.25945255	-2.15100901	3.40000000
H	6.03848799	-3.79721523	3.40000000
H	7.42143441	-4.91346797	3.40000000

Ys–Px//G–C

Total bonding energy = -10119.53 kcal mol^{-1}

H	0.63818449	1.70218235	0.00000000
H	7.07058106	0.74898656	0.00000000
H	2.85452803	2.96137650	0.00000000
C	2.71755539	-3.33320535	0.00000000
C	2.77755442	-4.75490259	0.00000000
C	4.06524956	-5.23493337	0.00000000
S	5.22492047	-3.94917317	0.00000000
C	3.95942088	-2.71598426	0.00000000
H	6.45578738	-1.61066212	0.00000000
H	1.78443692	-2.78797264	0.00000000
H	1.90112590	-5.39576448	0.00000000
H	4.40363650	-6.26151712	0.00000000
C	5.65437255	-0.87887180	0.00000000
C	6.01749567	0.47500534	0.00000000
N	5.13094596	1.48831306	0.00000000
C	3.85716813	1.09233431	0.00000000
C	3.34400447	-0.25000221	0.00000000
C	4.30808119	-1.29697612	0.00000000
N	2.77278892	1.95273229	0.00000000
C	1.60265971	1.21266497	0.00000000
C	1.90963503	-0.13273547	0.00000000
C	10.59880049	-5.28389191	0.00000000
C	9.19543710	-5.15255575	0.00000000
C	8.90957779	-3.75463872	0.00000000
C	10.13122020	-3.09837946	0.00000000
N	11.14668664	-4.03366741	0.00000000
O	9.49519457	-0.89735379	0.00000000
H	7.93634073	-3.29272852	0.00000000
H	12.12769203	-3.77597151	0.00000000
H	11.21055644	-6.17467959	0.00000000
N	10.44340224	-1.70914713	0.00000000
O	11.66263444	-1.39627256	0.00000000
C	8.25934607	-6.21491341	0.00000000
C	7.45639021	-7.12356593	0.00000000
C	6.49384262	-8.21830938	0.00000000
H	5.84925838	-8.18095263	0.88875216
H	1.17127561	-0.91935440	0.00000000
H	5.84925838	-8.18095263	-0.88875216
H	7.00275426	-9.19072997	0.00000000

N	4.97119005	1.05533672	3.40000000
O	4.34528524	-3.53812458	3.40000000
N	4.55667268	-1.23807420	3.40000000
C	4.06244004	0.05043940	3.40000000
N	2.75810350	0.33063542	3.40000000
C	1.98630422	-0.77789430	3.40000000
C	2.37868353	-2.13086291	3.40000000
C	3.77981851	-2.42464605	3.40000000
N	0.60593208	-0.81231267	3.40000000
C	0.23111076	-2.15846856	3.40000000
N	1.26559359	-2.96965052	3.40000000
H	5.98534901	0.89040548	3.40000000
H	4.60944716	1.99848779	3.40000000
H	5.58898402	-1.37787815	3.40000000
H	-0.00000000	-0.00000000	3.40000000
H	-0.81025242	-2.45627290	3.40000000
O	7.83318423	0.57821431	3.40000000
N	7.07225635	-3.96489646	3.40000000
N	7.42685680	-1.69230122	3.40000000
C	9.36213639	-3.17387907	3.40000000
C	8.23575027	-0.59704963	3.40000000
N	9.63845619	-0.82879603	3.40000000
C	10.17549178	-2.08385156	3.40000000
C	7.93637707	-2.93939270	3.40000000
H	9.77098431	-4.17827316	3.40000000
H	10.22420135	0.00000000	3.40000000
H	11.25945255	-2.15100901	3.40000000
H	6.03848799	-3.79721523	3.40000000
H	7.42143441	-4.91346797	3.40000000

Yi–Px//G–C

Total bonding energy = -10205.79 kcal mol^{-1}

H	0.36306980	-1.69073465	0.00000000
H	6.46530076	0.55209666	0.00000000
H	2.78505865	-2.48218108	0.00000000
N	1.33302781	3.45946973	0.00000000
C	1.14109787	4.82245093	0.00000000
C	2.35492263	5.48085208	0.00000000
N	3.31419405	4.48485184	0.00000000
C	2.65680721	3.26401272	0.00000000
H	5.35514429	2.76048351	0.00000000
H	0.38673683	1.03243420	0.00000000
H	0.14912731	5.25596540	0.00000000
H	2.61288317	6.52875262	0.00000000
C	4.73909019	1.86560554	0.00000000
C	5.37774180	0.61452904	0.00000000
N	4.72448910	-0.56319769	0.00000000
C	3.39474292	-0.45019711	0.00000000
C	2.61557441	0.75564757	0.00000000
C	3.33512787	1.97483841	0.00000000
N	2.50651055	-1.50943156	0.00000000
C	1.20687664	-1.01374846	0.00000000

C	1.23498562	0.36508514	0.00000000
C	9.71410795	5.05718749	0.00000000
C	8.35321272	4.68855225	0.00000000
C	8.31008743	3.26252777	0.00000000
C	9.62568637	2.82484287	0.00000000
N	10.46588573	3.91927181	0.00000000
O	9.37529321	0.54740458	0.00000000
H	7.43946428	2.62836396	0.00000000
H	11.47681004	3.83274594	0.00000000
H	10.16373597	6.03973869	0.00000000
N	10.17081600	1.50715040	0.00000000
O	11.42521199	1.41052455	0.00000000
C	7.26554807	5.59690525	0.00000000
C	6.39060498	6.43829997	0.00000000
C	5.38295886	7.49332904	0.00000000
H	4.74163310	7.43635214	-0.88925334
H	4.31803023	4.63482027	0.00000000
H	4.74163310	7.43635214	0.88925334
H	5.86308746	8.48033350	0.00000000
N	4.97119005	-1.05533672	-3.40000000
O	4.34528524	3.53812458	-3.40000000
N	4.55667268	1.23807420	-3.40000000
C	4.06244004	-0.05043940	-3.40000000
N	2.75810350	-0.33063542	-3.40000000
C	1.98630422	0.77789430	-3.40000000
C	2.37868353	2.13086291	-3.40000000
C	3.77981851	2.42464605	-3.40000000
N	0.60593208	0.81231267	-3.40000000
C	0.23111076	2.15846856	-3.40000000
N	1.26559359	2.96965052	-3.40000000
H	5.98534901	-0.89040548	-3.40000000
H	4.60944716	-1.99848779	-3.40000000
H	5.58898402	1.37787815	-3.40000000
H	-0.00000000	0.00000000	-3.40000000
H	-0.81025242	2.45627290	-3.40000000
O	7.83318423	-0.57821431	-3.40000000
N	7.07225635	3.96489646	-3.40000000
N	7.42685680	1.69230122	-3.40000000
C	9.36213639	3.17387907	-3.40000000
C	8.23575027	0.59704963	-3.40000000
N	9.63845619	0.82879603	-3.40000000
C	10.17549178	2.08385156	-3.40000000
C	7.93637707	2.93939270	-3.40000000
H	9.77098431	4.17827316	-3.40000000
H	10.22420135	-0.00000000	-3.40000000
H	11.25945255	2.15100901	-3.40000000
H	6.03848799	3.79721523	-3.40000000
H	7.42143441	4.91346797	-3.40000000

Do–Px//G–C

Total bonding energy = -10079.65 kcal mol^{-1}

H	0.81751879	-1.57978776	0.00000000

H	7.30046418	-1.46491657	0.00000000
H	2.78134252	-3.24199037	0.00000000
C	3.27916109	2.95388005	0.00000000
C	3.64994995	4.33592231	0.00000000
C	5.01728860	4.37671679	0.00000000
O	5.53171277	3.09925077	0.00000000
C	4.44594823	2.22157265	0.00000000
H	6.89412831	0.94693581	0.00000000
H	2.28735790	2.52461794	0.00000000
H	2.98500691	5.18979363	0.00000000
H	5.75077557	5.17007535	0.00000000
C	6.03880204	0.28211185	0.00000000
C	6.27455137	-1.10207400	0.00000000
N	5.30864589	-2.04693291	0.00000000
C	4.07774670	-1.53365373	0.00000000
C	3.69860446	-0.16486874	0.00000000
C	4.72851603	0.80511804	0.00000000
N	2.87413125	-2.23297705	0.00000000
C	1.86209200	-1.29167172	0.00000000
N	2.30858870	-0.04780511	0.00000000
C	10.65437834	5.11218882	0.00000000
C	9.24695597	5.04099781	0.00000000
C	8.89689365	3.65636861	0.00000000
C	10.09192579	2.94934866	0.00000000
N	11.14707352	3.84083906	0.00000000
O	9.38325143	0.76806665	0.00000000
H	7.90201620	3.23608687	0.00000000
H	12.11578060	3.53987587	0.00000000
H	11.30299029	5.97652788	0.00000000
N	10.35623572	1.55049148	0.00000000
O	11.56426150	1.19639532	0.00000000
C	8.36160050	6.14621749	0.00000000
C	7.59588277	7.08705502	0.00000000
C	6.66248257	8.20751831	0.00000000
H	6.01613104	8.18548168	-0.88766262
H	6.01613104	8.18548168	0.88766262
H	7.19777113	9.16567402	0.00000000
N	4.97119005	-1.05533672	-3.40000000
O	4.34528524	3.53812458	-3.40000000
N	4.55667268	1.23807420	-3.40000000
C	4.06244004	-0.05043940	-3.40000000
N	2.75810350	-0.33063542	-3.40000000
C	1.98630422	0.77789430	-3.40000000
C	2.37868353	2.13086291	-3.40000000
C	3.77981851	2.42464605	-3.40000000
N	0.60593208	0.81231267	-3.40000000
C	0.23111076	2.15846856	-3.40000000
N	1.26559359	2.96965052	-3.40000000
H	5.98534901	-0.89040548	-3.40000000
H	4.60944716	-1.99848779	-3.40000000
H	5.58898402	1.37787815	-3.40000000
H	-0.00000000	0.00000000	-3.40000000

H	-0.81025242	2.45627290	-3.40000000
O	7.83318423	-0.57821431	-3.40000000
N	7.07225635	3.96489646	-3.40000000
N	7.42685680	1.69230122	-3.40000000
C	9.36213639	3.17387907	-3.40000000
C	8.23575027	0.59704963	-3.40000000
N	9.63845619	0.82879603	-3.40000000
C	10.17549178	2.08385156	-3.40000000
C	7.93637707	2.93939270	-3.40000000
H	9.77098431	4.17827316	-3.40000000
H	10.22420135	-0.00000000	-3.40000000
H	11.25945255	2.15100901	-3.40000000
H	6.03848799	3.79721523	-3.40000000
H	7.42143441	4.91346797	-3.40000000

Dp–Px//G–C

Total bonding energy = -10282.75 kcal mol^{-1}

H	0.87693204	-1.55715699	0.00000000
H	7.35326871	-1.28062344	0.00000000
H	2.88550758	-3.16333522	0.00000000
C	4.46570481	2.39055562	0.00000000
N	5.55785189	3.19456123	0.00000000
H	6.29202375	8.51289157	0.88741770
H	6.29202375	8.51289157	-0.88741770
N	5.42653866	4.52556614	0.00000000
H	6.88248931	1.12772702	0.00000000
C	4.19727530	5.06708418	0.00000000
C	3.02713193	4.30182542	0.00000000
C	3.15870861	2.92269965	0.00000000
C	6.04944010	0.43691520	0.00000000
C	6.31977438	-0.94113492	0.00000000
N	5.37238030	-1.90391588	0.00000000
C	4.13321294	-1.42007279	0.00000000
C	3.72078170	-0.05589091	0.00000000
C	4.73264835	0.93247941	0.00000000
N	2.94943507	-2.15189184	0.00000000
C	1.91354329	-1.24170608	0.00000000
N	2.32451397	0.01446332	0.00000000
C	10.77248954	5.21594936	0.00000000
C	9.36269675	5.23102076	0.00000000
C	8.92570962	3.87060348	0.00000000
C	10.07856034	3.09220343	0.00000000
N	11.18810467	3.91790022	0.00000000
O	9.26127145	0.94585487	0.00000000
H	7.90195798	3.51788783	0.00000000
H	12.13580788	3.55740135	0.00000000
H	11.47276416	6.03923701	0.00000000
N	10.27018345	1.68465444	0.00000000
O	11.45950118	1.26567184	0.00000000
C	8.55117206	6.39034922	0.00000000
C	7.83587533	7.36816721	0.00000000
C	6.94004029	8.51742456	0.00000000

H	2.04914038	4.77808712	0.00000000
H	2.30444526	2.25387715	0.00000000
H	4.17410762	6.15572023	0.00000000
H	7.49940533	9.46226455	0.00000000
N	4.97119005	-1.05533672	-3.40000000
O	4.34528524	3.53812458	-3.40000000
N	4.55667268	1.23807420	-3.40000000
C	4.06244004	-0.05043940	-3.40000000
N	2.75810350	-0.33063542	-3.40000000
C	1.98630422	0.77789430	-3.40000000
C	2.37868353	2.13086291	-3.40000000
C	3.77981851	2.42464605	-3.40000000
N	0.60593208	0.81231267	-3.40000000
C	0.23111076	2.15846856	-3.40000000
N	1.26559359	2.96965052	-3.40000000
H	5.98534901	-0.89040548	-3.40000000
H	4.60944716	-1.99848779	-3.40000000
H	5.58898402	1.37787815	-3.40000000
H	-0.00000000	0.00000000	-3.40000000
H	-0.81025242	2.45627290	-3.40000000
O	7.83318423	-0.57821431	-3.40000000
N	7.07225635	3.96489646	-3.40000000
N	7.42685680	1.69230122	-3.40000000
C	9.36213639	3.17387907	-3.40000000
C	8.23575027	0.59704963	-3.40000000
N	9.63845619	0.82879603	-3.40000000
C	10.17549178	2.08385156	-3.40000000
C	7.93637707	2.93939270	-3.40000000
H	9.77098431	4.17827316	-3.40000000
H	10.22420135	-0.00000000	-3.40000000
H	11.25945255	2.15100901	-3.40000000
H	6.03848799	3.79721523	-3.40000000
H	7.42143441	4.91346797	-3.40000000

Dss–Px//G–C
Total bonding energy = -11052.53 kcal mol^{-1}

H	1.38103284	1.79574636	0.00000000
H	7.73737104	0.51702955	0.00000000
H	3.61562998	3.07353560	0.00000000
C	2.95587748	-3.03541977	0.00000000
C	2.80059786	-4.43882256	0.00000000
C	3.99895426	-5.14048056	0.00000000
S	5.35541516	-4.02156309	0.00000000
C	4.27955743	-2.61899181	0.00000000
H	6.91028096	-1.77431612	0.00000000
H	2.14049108	-2.32146426	0.00000000
H	1.83385628	-4.93336154	0.00000000
H	3.39920977	-10.04623184	0.00000000
C	6.18047863	-0.97121055	0.00000000
C	6.66367740	0.34603147	0.00000000
N	5.88010969	1.44719142	0.00000000
C	4.57864733	1.15867427	0.00000000

C	3.95927248	-0.12125961	0.00000000
C	4.79905750	-1.26433212	0.00000000
N	3.52198020	2.06460736	0.00000000
C	2.35642927	1.32383467	0.00000000
N	2.57056233	0.01999405	0.00000000
C	11.70541466	-5.42994712	0.00000000
C	10.32752048	-5.72919805	0.00000000
C	9.63042931	-4.48361791	0.00000000
C	10.60062177	-3.48874067	0.00000000
N	11.85040355	-4.07489072	0.00000000
O	9.34985312	-1.56965066	0.00000000
H	8.56139326	-4.33049589	0.00000000
H	12.70736297	-3.53199507	0.00000000
H	12.55612832	-6.09626913	0.00000000
N	10.49380860	-2.07083341	0.00000000
O	11.56697197	-1.41379803	0.00000000
C	9.77812109	-7.03360638	0.00000000
C	9.35195291	-8.16889332	0.00000000
C	8.85958200	-9.54059755	0.00000000
C	4.20199642	-6.56874919	0.00000000
C	5.37987025	-7.30179023	0.00000000
C	5.17026732	-8.70975613	0.00000000
C	3.84072652	-9.05794705	0.00000000
S	2.81389496	-7.65790760	0.00000000
H	8.24651442	-9.74206914	-0.88822616
H	8.24651442	-9.74206914	0.88822616
H	6.36401311	-6.84318441	0.00000000
H	5.97216763	-9.43904919	0.00000000
H	9.69347953	-10.25429326	0.00000000
N	4.97119005	1.05533672	3.40000000
O	4.34528524	-3.53812458	3.40000000
N	4.55667268	-1.23807420	3.40000000
C	4.06244004	0.05043940	3.40000000
N	2.75810350	0.33063542	3.40000000
C	1.98630422	-0.77789430	3.40000000
C	2.37868353	-2.13086291	3.40000000
C	3.77981851	-2.42464605	3.40000000
N	0.60593208	-0.81231267	3.40000000
C	0.23111076	-2.15846856	3.40000000
N	1.26559359	-2.96965052	3.40000000
H	5.98534901	0.89040548	3.40000000
H	4.60944716	1.99848779	3.40000000
H	5.58898402	-1.37787815	3.40000000
H	-0.00000000	-0.00000000	3.40000000
H	-0.81025242	-2.45627290	3.40000000
O	7.83318423	0.57821431	3.40000000
N	7.07225635	-3.96489646	3.40000000
N	7.42685680	-1.69230122	3.40000000
C	9.36213639	-3.17387907	3.40000000
C	8.23575027	-0.59704963	3.40000000
N	9.63845619	-0.82879603	3.40000000
C	10.17549178	-2.08385156	3.40000000

C	7.93637707	-2.93939270	3.40000000
H	9.77098431	-4.17827316	3.40000000
H	10.22420135	0.00000000	3.40000000
H	11.25945255	-2.15100901	3.40000000
H	6.03848799	-3.79721523	3.40000000
H	7.42143441	-4.91346797	3.40000000

Dt–Px//G–C

Total bonding energy = -9915.57 kcal mol^{-1}

H	0.68964348	1.66555388	0.00000000
H	7.11777631	0.83668213	0.00000000
H	2.83397866	3.09459045	0.00000000
N	2.68316697	-3.01312336	0.00000000
C	2.64630769	-4.38420711	0.00000000
C	3.86599024	-5.01812392	0.00000000
S	5.14534723	-3.85630831	0.00000000
C	3.91345181	-2.55326359	0.00000000
H	6.45243477	-1.50574315	0.00000000
H	5.75860710	-7.99327872	-0.88868877
H	1.68585927	-4.89042967	0.00000000
H	4.07448619	-6.07937681	0.00000000
C	5.66634207	-0.75749215	0.00000000
C	6.05894576	0.58830291	0.00000000
N	5.19567236	1.62647077	0.00000000
C	3.91898351	1.24923014	0.00000000
C	3.38273662	-0.07587787	0.00000000
C	4.30968415	-1.14609727	0.00000000
N	2.80744000	2.08150976	0.00000000
C	1.69449891	1.25915867	0.00000000
N	1.99129851	-0.02633152	0.00000000
C	10.55678003	-5.14023881	0.00000000
C	9.15158663	-5.03024018	0.00000000
C	8.84617980	-3.63708023	0.00000000
C	10.05653889	-2.96128983	0.00000000
N	11.08609278	-3.88161047	0.00000000
O	9.37403803	-0.77544087	0.00000000
H	7.86635234	-3.19081577	0.00000000
H	12.06370081	-3.61119522	0.00000000
H	11.18230042	-6.02143288	0.00000000
N	10.34062374	-1.56715529	0.00000000
O	11.55201340	-1.22799049	0.00000000
C	8.22028565	-6.09633130	0.00000000
C	7.39629280	-6.98588430	0.00000000
C	6.40162787	-8.05141932	0.00000000
H	5.75860710	-7.99327872	0.88868877
H	6.88007958	-9.03917337	0.00000000
N	4.97119005	1.05533672	3.40000000
O	4.34528524	-3.53812458	3.40000000
N	4.55667268	-1.23807420	3.40000000
C	4.06244004	0.05043940	3.40000000
N	2.75810350	0.33063542	3.40000000
C	1.98630422	-0.77789430	3.40000000

C	2.37868353	-2.13086291	3.40000000
C	3.77981851	-2.42464605	3.40000000
N	0.60593208	-0.81231267	3.40000000
C	0.23111076	-2.15846856	3.40000000
N	1.26559359	-2.96965052	3.40000000
H	5.98534901	0.89040548	3.40000000
H	4.60944716	1.99848779	3.40000000
H	5.58898402	-1.37787815	3.40000000
H	-0.00000000	-0.00000000	3.40000000
H	-0.81025242	-2.45627290	3.40000000
O	7.83318423	0.57821431	3.40000000
N	7.07225635	-3.96489646	3.40000000
N	7.42685680	-1.69230122	3.40000000
C	9.36213639	-3.17387907	3.40000000
C	8.23575027	-0.59704963	3.40000000
N	9.63845619	-0.82879603	3.40000000
C	10.17549178	-2.08385156	3.40000000
C	7.93637707	-2.93939270	3.40000000
H	9.77098431	-4.17827316	3.40000000
H	10.22420135	0.00000000	3.40000000
H	11.25945255	-2.15100901	3.40000000
H	6.03848799	-3.79721523	3.40000000
H	7.42143441	-4.91346797	3.40000000

Yo–Px//G–C

Total bonding energy = -10178.29 kcal mol^{-1}

H	0.71245644	-1.68020171	0.00000000
H	7.20482042	-1.27824905	0.00000000
H	2.80906033	-3.12907408	0.00000000
C	3.24726602	3.19796420	0.00000000
C	3.67360706	4.56462704	0.00000000
C	5.03969017	4.54692025	0.00000000
O	5.49608431	3.24785844	0.00000000
C	4.37890555	2.41018253	0.00000000
H	6.79161992	1.13498340	0.00000000
H	2.22685109	2.84759884	0.00000000
H	3.04323986	5.44420830	0.00000000
H	5.80602805	5.30866229	0.00000000
C	5.93855444	0.46785088	0.00000000
C	6.17899735	-0.91479068	0.00000000
N	5.21198114	-1.85257766	0.00000000
C	3.97363982	-1.35463903	0.00000000
C	3.57800675	0.02344357	0.00000000
C	4.62710438	0.98163244	0.00000000
N	2.81732097	-2.11721811	0.00000000
C	1.71588775	-1.27648774	0.00000000
C	2.14041671	0.03725822	0.00000000
C	10.65729348	5.21316515	0.00000000
C	9.24881147	5.16986303	0.00000000
C	8.87134395	3.79225140	0.00000000
C	10.05189827	3.06204855	0.00000000
N	11.12458649	3.93208221	0.00000000

O	9.29639164	0.89753466	0.00000000
H	7.86951456	3.38888318	0.00000000
H	12.08709602	3.61181343	0.00000000
H	11.32302702	6.06438573	0.00000000
N	10.28627468	1.65755297	0.00000000
O	11.48662095	1.27806628	0.00000000
C	8.38496233	6.29203164	0.00000000
C	7.63607232	7.24615561	0.00000000
C	6.72300561	8.38321718	0.00000000
H	6.07624024	8.37295350	0.88763111
H	6.07624024	8.37295350	-0.88763111
H	1.48077421	0.89199938	0.00000000
H	7.27583009	9.33142125	0.00000000
N	4.97119005	-1.05533672	-3.40000000
O	4.34528524	3.53812458	-3.40000000
N	4.55667268	1.23807420	-3.40000000
C	4.06244004	-0.05043940	-3.40000000
N	2.75810350	-0.33063542	-3.40000000
C	1.98630422	0.77789430	-3.40000000
C	2.37868353	2.13086291	-3.40000000
C	3.77981851	2.42464605	-3.40000000
N	0.60593208	0.81231267	-3.40000000
C	0.23111076	2.15846856	-3.40000000
N	1.26559359	2.96965052	-3.40000000
H	5.98534901	-0.89040548	-3.40000000
H	4.60944716	-1.99848779	-3.40000000
H	5.58898402	1.37787815	-3.40000000
H	-0.00000000	0.00000000	-3.40000000
H	-0.81025242	2.45627290	-3.40000000
O	7.83318423	-0.57821431	-3.40000000
N	7.07225635	3.96489646	-3.40000000
N	7.42685680	1.69230122	-3.40000000
C	9.36213639	3.17387907	-3.40000000
C	8.23575027	0.59704963	-3.40000000
N	9.63845619	0.82879603	-3.40000000
C	10.17549178	2.08385156	-3.40000000
C	7.93637707	2.93939270	-3.40000000
H	9.77098431	4.17827316	-3.40000000
H	10.22420135	-0.00000000	-3.40000000
H	11.25945255	2.15100901	-3.40000000
H	6.03848799	3.79721523	-3.40000000
H	7.42143441	4.91346797	-3.40000000

Ps–Px//G–C

Total bonding energy = -9920.74 kcal mol^{-1}

H	0.58247821	1.54761686	0.00000000
H	6.83318049	-0.12052091	0.00000000
H	2.87041168	2.72963660	0.00000000
C	2.07671232	-3.43091673	0.00000000
C	1.98589757	-4.84759061	0.00000000
C	3.22295524	-5.45879802	0.00000000
S	4.52459024	-4.31641102	0.00000000

C	3.38894984	-2.97861921	0.00000000
H	5.33347025	-8.00695437	-0.88867146
H	1.23202695	-2.75137036	0.00000000
H	1.04839390	-5.39531428	0.00000000
H	3.43798164	-6.51915277	0.00000000
N	5.24265504	-1.44139703	0.00000000
C	5.74918842	-0.19385876	0.00000000
N	5.06552977	0.97086786	0.00000000
C	3.74551380	0.77499023	0.00000000
C	3.07351743	-0.47269809	0.00000000
C	3.89371171	-1.62243943	0.00000000
N	2.73642670	1.72480362	0.00000000
C	1.53466428	1.03050442	0.00000000
N	1.69040275	-0.27917470	0.00000000
C	9.99956345	-5.05568179	0.00000000
C	8.59954606	-4.89086403	0.00000000
C	8.33954054	-3.48436264	0.00000000
C	9.58394456	-2.86184680	0.00000000
N	10.57680394	-3.82241090	0.00000000
O	9.06147030	-0.62476226	0.00000000
H	7.37612702	-2.98872885	0.00000000
H	11.56309523	-3.58596768	0.00000000
H	10.58749415	-5.96245136	0.00000000
N	9.96169260	-1.48964127	0.00000000
O	11.19594424	-1.23653595	0.00000000
C	7.66970870	-5.95894072	0.00000000
C	6.90725534	-6.90151843	0.00000000
C	5.97897178	-8.02530476	0.00000000
H	5.33347025	-8.00695437	0.88867146
H	6.51704559	-8.98200191	0.00000000
N	4.97119005	1.05533672	3.40000000
O	4.34528524	-3.53812458	3.40000000
N	4.55667268	-1.23807420	3.40000000
C	4.06244004	0.05043940	3.40000000
N	2.75810350	0.33063542	3.40000000
C	1.98630422	-0.77789430	3.40000000
C	2.37868353	-2.13086291	3.40000000
C	3.77981851	-2.42464605	3.40000000
N	0.60593208	-0.81231267	3.40000000
C	0.23111076	-2.15846856	3.40000000
N	1.26559359	-2.96965052	3.40000000
H	5.98534901	0.89040548	3.40000000
H	4.60944716	1.99848779	3.40000000
H	5.58898402	-1.37787815	3.40000000
H	-0.00000000	-0.00000000	3.40000000
H	-0.81025242	-2.45627290	3.40000000
O	7.83318423	0.57821431	3.40000000
N	7.07225635	-3.96489646	3.40000000
N	7.42685680	-1.69230122	3.40000000
C	9.36213639	-3.17387907	3.40000000
C	8.23575027	-0.59704963	3.40000000
N	9.63845619	-0.82879603	3.40000000

C	10.17549178	-2.08385156	3.40000000
C	7.93637707	-2.93939270	3.40000000
H	9.77098431	-4.17827316	3.40000000
H	10.22420135	0.00000000	3.40000000
H	11.25945255	-2.15100901	3.40000000
H	6.03848799	-3.79721523	3.40000000
H	7.42143441	-4.91346797	3.40000000

Es–Px//G–C

Total bonding energy = -10020.38 kcal mol^{-1}

H	0.52169509	1.65228178	0.00000000
H	6.74363717	-0.21117396	0.00000000
H	2.87770056	2.63086532	0.00000000
C	2.08956934	-3.65897610	0.00000000
C	2.08349855	-5.07984305	0.00000000
C	3.35468005	-5.61253096	0.00000000
S	4.58070970	-4.39019239	0.00000000
C	3.36720314	-3.11927879	0.00000000
H	0.73059774	-1.02352759	0.00000000
H	1.18697452	-3.06153047	0.00000000
H	1.18034971	-5.68255898	0.00000000
H	3.63380060	-6.65737236	0.00000000
N	5.16632555	-1.55003804	0.00000000
C	5.66073281	-0.29748536	0.00000000
N	4.95858567	0.85060995	0.00000000
C	3.63802909	0.65339293	0.00000000
C	2.96512966	-0.60986369	0.00000000
C	3.82095548	-1.73844678	0.00000000
N	2.67468428	1.63900584	0.00000000
C	1.41671800	1.04531336	0.00000000
C	1.55442746	-0.32582929	0.00000000
C	9.96530733	-5.13093780	0.00000000
C	8.56317556	-4.98232117	0.00000000
C	8.28665062	-3.57916819	0.00000000
C	9.52427941	-2.94251960	0.00000000
N	10.52812676	-3.89163742	0.00000000
O	8.98031381	-0.71070916	0.00000000
H	7.31921321	-3.09141171	0.00000000
H	11.51116542	-3.64171406	0.00000000
H	10.56232591	-6.03171705	0.00000000
N	9.88826316	-1.56644351	0.00000000
O	11.12062020	-1.30194282	0.00000000
C	7.65276083	-6.06753193	0.00000000
C	6.92549321	-7.03817322	0.00000000
C	6.04816569	-8.20265074	0.00000000
H	5.40301332	-8.21542461	0.88882449
H	5.40301332	-8.21542461	-0.88882449
H	6.63086560	-9.13303380	0.00000000
N	4.97119005	1.05533672	3.40000000
O	4.34528524	-3.53812458	3.40000000
N	4.55667268	-1.23807420	3.40000000
C	4.06244004	0.05043940	3.40000000

N	2.75810350	0.33063542	3.40000000
C	1.98630422	-0.77789430	3.40000000
C	2.37868353	-2.13086291	3.40000000
C	3.77981851	-2.42464605	3.40000000
N	0.60593208	-0.81231267	3.40000000
C	0.23111076	-2.15846856	3.40000000
N	1.26559359	-2.96965052	3.40000000
H	5.98534901	0.89040548	3.40000000
H	4.60944716	1.99848779	3.40000000
H	5.58898402	-1.37787815	3.40000000
H	-0.00000000	-0.00000000	3.40000000
H	-0.81025242	-2.45627290	3.40000000
O	7.83318423	0.57821431	3.40000000
N	7.07225635	-3.96489646	3.40000000
N	7.42685680	-1.69230122	3.40000000
C	9.36213639	-3.17387907	3.40000000
C	8.23575027	-0.59704963	3.40000000
N	9.63845619	-0.82879603	3.40000000
C	10.17549178	-2.08385156	3.40000000
C	7.93637707	-2.93939270	3.40000000
H	9.77098431	-4.17827316	3.40000000
H	10.22420135	0.00000000	3.40000000
H	11.25945255	-2.15100901	3.40000000
H	6.03848799	-3.79721523	3.40000000
H	7.42143441	-4.91346797	3.40000000

Ysm–Px//G–C
Total bonding energy = -10488.59 kcal mol^{-1}

H	0.95531385	-1.83089127	0.00000000
H	7.40550545	-1.00094410	0.00000000
H	3.14687447	-3.13380383	0.00000000
C	3.14028662	3.16976569	0.00000000
C	3.23835261	4.59040519	0.00000000
C	4.53216613	5.05889785	0.00000000
S	5.65660829	3.72316791	0.00000000
C	4.36151365	2.51795215	0.00000000
H	6.83976954	1.37018048	0.00000000
H	2.19215956	2.65003879	0.00000000
H	2.37660535	5.25243123	0.00000000
C	5.02135510	6.47717626	0.00000000
C	6.02330251	0.65445124	0.00000000
C	6.35760466	-0.70689080	0.00000000
N	5.45311474	-1.70475653	0.00000000
C	4.18653084	-1.28493526	0.00000000
C	3.69995776	0.06638170	0.00000000
C	4.68365261	1.09496687	0.00000000
N	3.08487535	-2.12383509	0.00000000
C	1.92922388	-1.36040555	0.00000000
C	2.26363250	-0.02139433	0.00000000
C	11.30102134	5.44470739	0.00000000
C	9.89621195	5.55433980	0.00000000
C	9.37473481	4.22573385	0.00000000

C	10.46707157	3.37073212	0.00000000
N	11.62757821	4.11945506	0.00000000
O	9.46787595	1.30808365	0.00000000
H	8.33574929	3.93627692	0.00000000
H	12.55027230	3.69816460	0.00000000
H	12.05600301	6.21791157	0.00000000
N	10.53937396	1.94906868	0.00000000
O	11.68801925	1.43440554	0.00000000
C	9.14868898	6.75680164	0.00000000
C	8.49274578	7.77686617	0.00000000
C	7.69861909	8.99974415	0.00000000
H	7.05337523	9.05364626	-0.88677215
H	5.63737914	6.69335725	0.88172128
H	4.16759865	7.16300455	0.00000000
H	5.63737914	6.69335725	-0.88172128
H	1.54210985	0.78093609	0.00000000
H	7.05337523	9.05364626	0.88677215
H	8.34276138	9.88868087	0.00000000
N	4.97119005	-1.05533672	-3.40000000
O	4.34528524	3.53812458	-3.40000000
N	4.55667268	1.23807420	-3.40000000
C	4.06244004	-0.05043940	-3.40000000
N	2.75810350	-0.33063542	-3.40000000
C	1.98630422	0.77789430	-3.40000000
C	2.37868353	2.13086291	-3.40000000
C	3.77981851	2.42464605	-3.40000000
N	0.60593208	0.81231267	-3.40000000
C	0.23111076	2.15846856	-3.40000000
N	1.26559359	2.96965052	-3.40000000
H	5.98534901	-0.89040548	-3.40000000
H	4.60944716	-1.99848779	-3.40000000
H	5.58898402	1.37787815	-3.40000000
H	-0.00000000	0.00000000	-3.40000000
H	-0.81025242	2.45627290	-3.40000000
O	7.83318423	-0.57821431	-3.40000000
N	7.07225635	3.96489646	-3.40000000
N	7.42685680	1.69230122	-3.40000000
C	9.36213639	3.17387907	-3.40000000
C	8.23575027	0.59704963	-3.40000000
N	9.63845619	0.82879603	-3.40000000
C	10.17549178	2.08385156	-3.40000000
C	7.93637707	2.93939270	-3.40000000
H	9.77098431	4.17827316	-3.40000000
H	10.22420135	-0.00000000	-3.40000000
H	11.25945255	2.15100901	-3.40000000
H	6.03848799	3.79721523	-3.40000000
H	7.42143441	4.91346797	-3.40000000

Yss–Px//G–C

Total bonding energy = -11150.38 kcal mol^{-1}

H	1.30414896	2.00206711	0.00000000
H	7.64502110	0.55388171	0.00000000

H	3.61169995	3.08657777	0.00000000
C	2.99900014	-3.17846434	0.00000000
C	2.94468439	-4.59099767	0.00000000
C	4.19072950	-5.19996728	0.00000000
S	5.45612920	-3.98114568	0.00000000
C	4.28399701	-2.65497477	0.00000000
H	6.85266595	-1.75073172	0.00000000
H	2.11157652	-2.56149037	0.00000000
H	2.01625990	-5.15414334	0.00000000
C	4.50841363	-6.60691755	0.00000000
C	6.11063895	-0.95926759	0.00000000
C	6.57394746	0.36332679	0.00000000
N	5.76769599	1.44262111	0.00000000
C	4.46699134	1.14557766	0.00000000
C	3.85210464	-0.15266113	0.00000000
C	4.73554629	-1.27057353	0.00000000
N	3.45177045	2.08740086	0.00000000
C	2.22819065	1.43998500	0.00000000
C	2.43137102	0.07451737	0.00000000
C	11.83014337	-5.48339071	0.00000000
C	10.47238558	-5.86427340	0.00000000
C	9.70271568	-4.66192575	0.00000000
C	10.61210249	-3.61140638	0.00000000
N	11.89430981	-4.12202289	0.00000000
O	9.24981863	-1.76923021	0.00000000
H	8.62695793	-4.56678213	0.00000000
H	12.71761416	-3.52925616	0.00000000
H	12.71894524	-6.09793695	0.00000000
N	10.42097752	-2.20168791	0.00000000
O	11.45375206	-1.48268872	0.00000000
C	10.00654465	-7.20125724	0.00000000
C	9.66370381	-8.36450923	0.00000000
C	9.27634167	-9.76994194	0.00000000
C	5.74510179	-7.23558874	0.00000000
C	5.65928624	-8.65563625	0.00000000
C	4.36479911	-9.11809716	0.00000000
S	3.21912686	-7.81171642	0.00000000
H	6.68624369	-6.69525173	0.00000000
H	8.68159680	-10.01952208	-0.88861707
H	8.68159680	-10.01952208	0.88861707
H	6.52198034	-9.31086298	0.00000000
H	4.01193998	-10.14138059	0.00000000
H	1.63423810	-0.65270158	0.00000000
H	10.16301420	-10.41714011	0.00000000
N	4.97119005	1.05533672	3.40000000
O	4.34528524	-3.53812458	3.40000000
N	4.55667268	-1.23807420	3.40000000
C	4.06244004	0.05043940	3.40000000
N	2.75810350	0.33063542	3.40000000
C	1.98630422	-0.77789430	3.40000000
C	2.37868353	-2.13086291	3.40000000
C	3.77981851	-2.42464605	3.40000000

N	0.60593208	-0.81231267	3.40000000
C	0.23111076	-2.15846856	3.40000000
N	1.26559359	-2.96965052	3.40000000
H	5.98534901	0.89040548	3.40000000
H	4.60944716	1.99848779	3.40000000
H	5.58898402	-1.37787815	3.40000000
H	-0.00000000	-0.00000000	3.40000000
H	-0.81025242	-2.45627290	3.40000000
O	7.83318423	0.57821431	3.40000000
N	7.07225635	-3.96489646	3.40000000
N	7.42685680	-1.69230122	3.40000000
C	9.36213639	-3.17387907	3.40000000
C	8.23575027	-0.59704963	3.40000000
N	9.63845619	-0.82879603	3.40000000
C	10.17549178	-2.08385156	3.40000000
C	7.93637707	-2.93939270	3.40000000
H	9.77098431	-4.17827316	3.40000000
H	10.22420135	0.00000000	3.40000000
H	11.25945255	-2.15100901	3.40000000
H	6.03848799	-3.79721523	3.40000000
H	7.42143441	-4.91346797	3.40000000

S11.2. Chloroform

Cartesian Coordinates and Total Bonding Energies of W–C base pairs in Chloroform

Cartesian coordinates (Å) and total bonding energies (kcal mol^{-1}) of Watson–Crick base pairs optimized at ZORA-BLYP-D3(BJ)/TZ2P level of theory in chloroform.

A–T

Total bonding energy = -4504.94 kcal mol^{-1}

N	1.28041513	-0.60138331	0.00000000
C	1.89057292	-1.80443917	0.00000000
N	3.20869643	-2.06080188	0.00000000
C	3.93654147	-0.92376425	0.00000000
C	3.43650847	0.39016727	0.00000000
C	2.02812464	0.53849025	0.00000000
N	5.31078369	-0.77949580	0.00000000
C	5.57744183	0.57744063	0.00000000
N	4.47824891	1.31675968	0.00000000
N	1.39933249	1.72885722	0.00000000
H	1.21966012	-2.66232789	0.00000000
H	5.99396102	-1.52940202	0.00000000
H	6.59146160	0.95592322	0.00000000
H	1.94400228	2.58095917	0.00000000
H	0.37264757	1.77121483	0.00000000
N	-1.56992049	-0.57403089	0.00000000
C	-2.18891950	-1.80980370	0.00000000
N	-3.57705223	-1.73908606	0.00000000
C	-4.27801127	-0.55385689	0.00000000
C	-3.65747576	0.65616860	0.00000000
C	-2.19902120	0.67206671	0.00000000
O	-1.51421848	1.71818233	0.00000000
O	-1.57460682	-2.88357982	0.00000000
C	-4.40171236	1.96369717	0.00000000
H	-0.51496344	-0.58559961	0.00000000
H	-4.07113232	-2.62547596	0.00000000
H	-5.35861106	-0.65698719	0.00000000
H	-4.13982506	2.56233046	0.88121399
H	-5.48310354	1.79544653	0.00000000
H	-4.13982506	2.56233046	-0.88121399

G–C

Total bonding energy = -4414.01 kcal mol^{-1}

N	-2.56875722	-0.57287819	0.00000000
O	1.37084221	1.86842475	0.00000000
N	-0.64103706	0.73271571	0.00000000
C	-2.02049938	0.66164301	0.00000000
N	-2.80704292	1.74453528	0.00000000
C	-2.11066639	2.90279251	0.00000000
C	-0.71546692	3.08229281	0.00000000
C	0.11674744	1.92209772	0.00000000
N	-2.63213213	4.17952351	0.00000000
C	-1.55986425	5.06242264	0.00000000

N	-0.39470213	4.44106812	0.00000000
H	-2.00097174	-1.43275661	0.00000000
H	-3.57814444	-0.63541680	0.00000000
H	-0.09629357	-0.15901347	0.00000000
H	-3.61763123	4.42003412	0.00000000
H	-1.70835805	6.13428954	0.00000000
O	-1.04126495	-2.99434465	0.00000000
N	2.86440435	-0.54676198	0.00000000
N	0.89604239	-1.74586976	0.00000000
C	3.01090319	-2.95870092	0.00000000
C	0.21037294	-2.92012139	0.00000000
N	0.95381995	-4.11489580	0.00000000
C	2.31808203	-4.12978724	0.00000000
C	2.24791262	-1.73912635	0.00000000
H	4.09465261	-2.94530694	0.00000000
H	0.42404119	-4.98117811	0.00000000
H	2.79174688	-5.10546266	0.00000000
H	2.31838712	0.33232641	0.00000000
H	3.87487738	-0.50254538	0.00000000

A–T//G–C

Total bonding energy = -8929.82 kcal mol^{-1}

N	5.00124589	-1.00838958	0.00000000
O	4.31439792	3.57512911	0.00000000
N	4.56316571	1.27825998	0.00000000
C	4.08363083	-0.01712178	0.00000000
N	2.77812701	-0.31202208	0.00000000
C	1.98907622	0.78520120	0.00000000
C	2.37521426	2.13786499	0.00000000
C	3.76977416	2.44419170	0.00000000
N	0.61018743	0.81038579	0.00000000
C	0.22256913	2.14418538	0.00000000
N	1.25359689	2.96921021	0.00000000
H	6.01546686	-0.82638110	0.00000000
H	4.66003477	-1.96041300	0.00000000
H	5.59754734	1.42652009	0.00000000
H	0.00000000	0.00000000	0.00000000
H	-0.82080301	2.43110636	0.00000000
O	7.82914031	-0.56146045	0.00000000
N	7.12312219	3.99336689	0.00000000
N	7.44732185	1.71143725	0.00000000
C	9.39682962	3.17534130	0.00000000
C	8.25529553	0.61773344	0.00000000
N	9.64656408	0.82885228	0.00000000
C	10.19907071	2.07631739	0.00000000
C	7.97505830	2.95606283	0.00000000
H	9.81255981	4.17627118	0.00000000
H	10.23317680	0.00000000	0.00000000
H	11.28250062	2.12612238	0.00000000
H	6.09985058	3.83894401	0.00000000
H	7.48159458	4.93915143	0.00000000
N	5.24892689	0.86358006	3.40000000

C	5.60537575	-0.43741192	3.40000000
N	4.80138826	-1.51294692	3.40000000
C	3.49833363	-1.15987240	3.40000000
C	2.98582423	0.14924301	3.40000000
C	3.92925455	1.20540774	3.40000000
N	2.38359341	-1.97639509	3.40000000
C	1.27435438	-1.15057925	3.40000000
N	1.59169045	0.13554499	3.40000000
N	3.59513268	2.50952265	3.40000000
H	6.67870071	-0.62199409	3.40000000
H	2.38148441	-2.99083393	3.40000000
H	0.26903383	-1.55159765	3.40000000
H	2.61919522	2.77464337	3.40000000
H	4.32706512	3.23073759	3.40000000
N	7.34159095	2.79901758	3.40000000
C	8.63037253	2.29968173	3.40000000
N	9.61094836	3.28476268	3.40000000
C	9.33372758	4.63356230	3.40000000
C	8.06110802	5.11279652	3.40000000
C	6.97025144	4.14461555	3.40000000
O	5.76016697	4.45927128	3.40000000
O	8.89687932	1.09164641	3.40000000
C	7.73376522	6.58125346	3.40000000
H	6.56803186	2.08160936	3.40000000
H	10.57245614	2.96025538	3.40000000
H	10.20334629	5.28325075	3.40000000
H	7.13757388	6.84865325	2.51878601
H	8.64772530	7.18324361	3.40000000
H	7.13757388	6.84865325	4.28121399

Cartesian Coordinates and Total Bonding Energies of Ds variants and Px in Chloroform

Cartesian coordinates (Å) and total bonding energies (kcal mol^{-1}) of Ds variants and Px optimized at ZORA-BLYP-D3(BJ)/TZ2P level of theory in chloroform.

Ds

Total bonding energy = -3172.99 kcal mol^{-1}

H	6.88228745	-0.61305674	0.00000000
H	1.82392131	3.43558856	0.00000000
H	6.43562848	1.90213298	0.00000000
C	2.13594357	-2.52094733	0.00000000
C	1.13466447	-3.53071217	0.00000000
C	-0.14317780	-3.01690091	0.00000000
S	-0.14178043	-1.28464673	0.00000000
C	1.62198841	-1.23125198	0.00000000
H	0.59139887	1.32221434	0.00000000
H	3.20270872	-2.70960615	0.00000000
H	1.34797388	-4.59532618	0.00000000
H	-1.08559329	-3.55007182	0.00000000
C	1.67734615	1.28479389	0.00000000
C	2.37706722	2.49811304	0.00000000
N	3.72571666	2.60519891	0.00000000
C	4.34422951	1.42096269	0.00000000
C	3.75283885	0.12561475	0.00000000
C	2.33914633	0.03524534	0.00000000
N	5.71232317	1.19107820	0.00000000
C	5.89345603	-0.17196823	0.00000000
N	4.75329940	-0.84870934	0.00000000

Bs

Total bonding energy = -3274.25 kcal mol^{-1}

H	6.87167184	-0.69635935	0.00000000
H	1.70918003	3.44690121	0.00000000
H	6.51620514	1.82148110	0.00000000
C	2.14975780	-2.50293133	0.00000000
C	1.14997881	-3.51782928	0.00000000
C	-0.12884957	-3.01169854	0.00000000
S	-0.12961179	-1.27615641	0.00000000
C	1.63641928	-1.21422892	0.00000000
H	0.56792860	1.28806230	0.00000000
H	3.21631020	-2.69020972	0.00000000
H	1.36915547	-4.58153169	0.00000000
H	-1.07014475	-3.54675242	0.00000000
C	1.65509685	1.29463415	0.00000000
C	2.30816072	2.53966229	0.00000000
C	3.70175077	2.63624131	0.00000000
C	4.40076181	1.42651683	0.00000000
C	3.76391771	0.15217344	0.00000000
C	2.34456354	0.06430645	0.00000000
N	5.76152944	1.14515449	0.00000000
C	5.89818769	-0.22203056	0.00000000
N	4.73600733	-0.85596993	0.00000000

H	4.20944585	3.59693228	0.00000000

Is

Total bonding energy = -3369.78 kcal mol^{-1}

H	7.07300038	-0.50300617	0.00000000
H	1.69216976	3.40536258	0.00000000
H	6.46818276	1.94598928	0.00000000
C	2.07802319	-2.55088489	0.00000000
C	1.04193058	-3.52944320	0.00000000
C	-0.21595582	-2.97667386	0.00000000
S	-0.14838462	-1.24327529	0.00000000
C	1.62245210	-1.24010573	0.00000000
H	0.57102902	1.23609638	0.00000000
H	3.12449617	-2.81926865	0.00000000
H	1.22475267	-4.59993615	0.00000000
H	-1.17661325	-3.47603938	0.00000000
C	1.65801901	1.25046946	0.00000000
C	2.29703065	2.50205327	0.00000000
C	3.68795108	2.59972213	0.00000000
C	4.40534294	1.40015097	0.00000000
C	3.78613458	0.10305522	0.00000000
C	2.35828895	0.02787160	0.00000000
N	5.77664602	1.20715596	0.00000000
C	6.05187548	-0.14653911	0.00000000
C	4.86643191	-0.85329752	0.00000000
H	4.19320657	3.56252023	0.00000000
H	4.80965054	-1.93003303	0.00000000

Ys

Total bonding energy = -3269.66 kcal mol^{-1}

H	7.06920817	-0.42575641	0.00000000
H	1.80728902	3.39405735	0.00000000
H	6.38259226	2.01410499	0.00000000
C	2.06389517	-2.56780943	0.00000000
C	1.02711734	-3.54216708	0.00000000
C	-0.22989606	-2.98299601	0.00000000
S	-0.16140375	-1.25270943	0.00000000
C	1.60612276	-1.25743358	0.00000000
H	0.59062311	1.27026676	0.00000000
H	3.11192551	-2.83234220	0.00000000
H	1.20476023	-4.61324963	0.00000000
H	-1.19136657	-3.48087285	0.00000000
C	1.67664975	1.24022953	0.00000000
C	2.36509453	2.45904342	0.00000000
N	3.71019777	2.56704439	0.00000000
C	4.34959807	1.39216201	0.00000000
C	3.77102764	0.07481309	0.00000000
C	2.35013588	-0.00051741	0.00000000
N	5.72281107	1.24541815	0.00000000
C	6.03770700	-0.10135906	0.00000000
C	4.87605048	-0.84777915	0.00000000
H	4.85098991	-1.92639499	0.00000000

Yi
Total bonding energy = -3359.67 kcal mol^{-1}

H	7.00745459	-0.48506470	0.00000000
H	1.73349286	3.32451785	0.00000000
H	6.32831837	1.95548743	0.00000000
N	2.16840877	-2.55630037	0.00000000
C	1.12736095	-3.46289565	0.00000000
C	-0.08651519	-2.80150541	0.00000000
N	0.22933321	-1.46010800	0.00000000
C	1.60551352	-1.33750072	0.00000000
H	0.52529219	1.19951346	0.00000000
H	-0.44302152	-0.70226554	0.00000000
H	1.30302957	-4.53107385	0.00000000
H	-1.10904477	-3.15053466	0.00000000
C	1.61119273	1.16907357	0.00000000
C	2.29636818	2.39259446	0.00000000
N	3.64104256	2.50938735	0.00000000
C	4.29233513	1.34030323	0.00000000
C	3.71823932	0.02325825	0.00000000
C	2.30383105	-0.05870410	0.00000000
N	5.66452008	1.19037267	0.00000000
C	5.97525176	-0.16221737	0.00000000
C	4.81217115	-0.90642121	0.00000000
H	4.73740825	-1.98313588	0.00000000

Do
Total bonding energy = -3231.21 kcal mol^{-1}

H	0.17783966	4.14228188	0.00000000
H	-3.71681068	-1.03824272	0.00000000
H	-2.32225886	3.62587064	0.00000000
C	2.24552660	-0.61336493	0.00000000
C	3.09536516	-1.76421159	0.00000000
C	2.27203697	-2.85748278	0.00000000
O	0.95333793	-2.46449787	0.00000000
C	0.94433408	-1.06918814	0.00000000
H	-1.55298215	-2.20774508	0.00000000
H	2.53701993	0.42689971	0.00000000
H	4.17743974	-1.77831660	0.00000000
H	2.44313439	-3.92425792	0.00000000
C	-1.56254836	-1.12279512	0.00000000
C	-2.79618727	-0.45726512	0.00000000
N	-2.94597364	0.88768240	0.00000000
C	-1.78314666	1.54669336	0.00000000
C	-0.47224197	0.99635179	0.00000000
C	-0.34133234	-0.41164469	0.00000000
N	-1.59270019	2.92129545	0.00000000
C	-0.23483318	3.14128036	0.00000000
N	0.47498082	2.02065700	0.00000000

Dp
Total bonding energy = -3433.84 kcal mol^{-1}

H	-0.05241239	4.17441305	0.00000000

H	-3.92095506	-1.02069643	0.00000000
H	-2.54804282	3.63820404	0.00000000
N	1.80406846	-3.20006379	0.00000000
C	3.00185174	-2.58998147	0.00000000
C	3.14577844	-1.19937627	0.00000000
C	1.99290839	-0.43296761	0.00000000
H	4.13195892	-0.74301077	0.00000000
H	-1.74135733	-2.18134940	0.00000000
H	2.01892498	0.65049163	0.00000000
H	3.86218747	-3.25589111	0.00000000
C	0.75453386	-1.10797125	0.00000000
C	-1.76385602	-1.09764245	0.00000000
C	-3.00109833	-0.43907100	0.00000000
N	-3.14945704	0.90511240	0.00000000
C	-1.99188547	1.56531797	0.00000000
C	-0.67311458	1.01740680	0.00000000
C	-0.54533649	-0.39006575	0.00000000
N	-1.81196011	2.94029496	0.00000000
C	-0.45831550	3.17066988	0.00000000
N	0.26107324	2.05690106	0.00000000
N	0.68450561	-2.46072450	0.00000000

Dss

Total bonding energy = -4205.15 kcal mol^{-1}

H	-0.52705756	6.88135608	0.00000000
H	3.41006336	1.73425502	0.00000000
H	1.97681513	6.38044320	0.00000000
C	-2.53723716	2.17592328	0.00000000
C	-3.56409562	1.20559109	0.00000000
C	-3.09829935	-0.10412751	0.00000000
S	-1.34197939	-0.12653100	0.00000000
C	-1.25633926	1.63736474	0.00000000
H	1.27111721	0.54805133	0.00000000
H	-2.70412727	3.24632629	0.00000000
H	-4.62154557	1.45172311	0.00000000
C	-3.85065872	-1.33559857	0.00000000
C	1.25731955	1.63459280	0.00000000
C	2.48488131	2.30770365	0.00000000
N	2.62166498	3.65417543	0.00000000
C	1.45086998	4.29874114	0.00000000
C	0.14321482	3.73653857	0.00000000
C	0.02100788	2.32398447	0.00000000
N	1.25121383	5.67179287	0.00000000
C	-0.10737628	5.88332065	0.00000000
N	-0.80877454	4.75783307	0.00000000
C	-3.38768237	-2.64415928	0.00000000
C	-4.42806965	-3.61492127	0.00000000
C	-5.68462791	-3.05301517	0.00000000
S	-5.61351300	-1.31723480	0.00000000
H	-6.64739732	-3.54793911	0.00000000
H	-2.33115813	-2.89420496	0.00000000
H	-4.25518113	-4.68673125	0.00000000

Dt

Total bonding energy = -3070.72 kcal mol^{-1}

H	-0.59777361	6.86057720	0.00000000
H	3.43297380	1.79002676	0.00000000
H	1.91471546	6.40226229	0.00000000
N	-2.43183866	2.10434432	0.00000000
C	-3.41149631	1.14029821	0.00000000
C	-2.96890537	-0.16064738	0.00000000
S	-1.24209728	-0.20574987	0.00000000
C	-1.22304071	1.57559668	0.00000000
H	1.31530980	0.56301336	0.00000000
N	-0.84753840	4.73491034	0.00000000
H	-4.45490985	1.43926554	0.00000000
H	-3.55094440	-1.07317611	0.00000000
C	1.28110320	1.64875277	0.00000000
C	2.49618251	2.34362226	0.00000000
N	2.60067432	3.69187362	0.00000000
C	1.42118456	4.31448393	0.00000000
C	0.11711933	3.72890185	0.00000000
C	0.03690014	2.31659095	0.00000000
N	1.20048539	5.68208765	0.00000000
C	-0.16199774	5.86931643	0.00000000

Yo

Total bonding energy = -3328.36 kcal mol^{-1}

H	0.00549607	4.31088021	0.00000000
H	-3.68011345	-1.04957796	0.00000000
H	-2.41687382	3.56908725	0.00000000
C	2.28568760	-0.69034040	0.00000000
C	3.09676471	-1.86969987	0.00000000
C	2.23683390	-2.93260185	0.00000000
O	0.93350649	-2.49143833	0.00000000
C	0.96704113	-1.09551898	0.00000000
H	-1.50911898	-2.21314203	0.00000000
H	2.64337288	0.32781076	0.00000000
H	4.17779546	-1.91873305	0.00000000
H	2.36989469	-4.00476408	0.00000000
C	-1.52361593	-1.12850097	0.00000000
C	-2.75954813	-0.46807014	0.00000000
N	-2.90508942	0.87400399	0.00000000
C	-1.74939190	1.54896104	0.00000000
C	-0.42033848	1.00571950	0.00000000
C	-0.30941479	-0.40992780	0.00000000
N	-1.63433949	2.92584293	0.00000000
C	-0.29374523	3.27176378	0.00000000
C	0.47928509	2.12686400	0.00000000
H	1.55897488	2.11697673	0.00000000

Ps

Total bonding energy = -3071.60 kcal mol^{-1}

H	6.94678607	-0.54290395	0.00000000
H	1.71277118	3.25067656	0.00000000

H	6.42797141	1.96085499	0.00000000
C	2.13231689	-2.53059080	0.00000000
C	1.09432870	-3.49941737	0.00000000
C	-0.16272846	-2.93002886	0.00000000
S	-0.10265465	-1.20012193	0.00000000
C	1.65660785	-1.22595399	0.00000000
N	4.82694732	-0.84200791	0.00000000
H	3.19194873	-2.75833474	0.00000000
H	1.26463312	-4.57163996	0.00000000
H	-1.12303976	-3.43097542	0.00000000
N	1.68667454	1.18314891	0.00000000
C	2.33719401	2.35849976	0.00000000
N	3.67086897	2.56517810	0.00000000
C	4.35588735	1.41499486	0.00000000
C	3.80415995	0.10806490	0.00000000
C	2.39519256	0.01880573	0.00000000
N	5.72408362	1.23015339	0.00000000
C	5.94503053	-0.13255419	0.00000000

Es

Total bonding energy = -3169.84 kcal mol^{-1}

H	-0.34751928	7.07798924	0.00000000
H	3.19972192	1.64103665	0.00000000
H	2.07129684	6.31829436	0.00000000
C	-2.58229702	2.02767265	0.00000000
C	-3.51854876	0.95855087	0.00000000
C	-2.90904670	-0.27807893	0.00000000
S	-1.18206499	-0.15741030	0.00000000
C	-1.26152292	1.60098424	0.00000000
C	-0.83923149	4.89973462	0.00000000
H	-2.87468701	3.06965402	0.00000000
H	-4.59540835	1.09659720	0.00000000
H	-3.37861169	-1.25409445	0.00000000
N	1.12958662	1.63413104	0.00000000
C	2.31203303	2.27208672	0.00000000
N	2.52310606	3.60149459	0.00000000
C	1.38574952	4.30987147	0.00000000
C	0.05601406	3.77360480	0.00000000
C	-0.02201603	2.35975716	0.00000000
N	1.28455065	5.67957331	0.00000000
C	-0.05821606	6.03627563	0.00000000
H	-1.91873570	4.89817718	0.00000000

Ysm

Total bonding energy = -3638.37 kcal mol^{-1}

H	3.02802659	2.64708547	0.00000000
H	1.43930915	-3.65927707	0.00000000
H	4.06579873	0.33403521	0.00000000
C	-2.19062252	1.07795181	0.00000000
C	-3.60933798	1.15804575	0.00000000
C	-4.23461934	-0.07133715	0.00000000
S	-3.05193143	-1.35309162	0.00000000

C	-1.69615645	-0.21892611	0.00000000
H	-0.85578597	-2.80957630	0.00000000
H	-1.55854137	1.95522546	0.00000000
H	-4.15678687	2.09676541	0.00000000
H	0.36310996	2.37437890	0.00000000
C	-0.04144652	-2.09027299	0.00000000
C	1.26819317	-2.58395708	0.00000000
N	2.37032866	-1.80484498	0.00000000
C	2.10691520	-0.49263497	0.00000000
C	0.81886840	0.14669108	0.00000000
C	-0.32143225	-0.70561305	0.00000000
N	3.06668157	0.50087475	0.00000000
C	2.44476045	1.73656058	0.00000000
C	1.07494332	1.56355877	0.00000000
C	-5.70674113	-0.37834168	0.00000000
H	-6.19245483	0.04963480	0.88575940
H	-5.89487385	-1.45621041	0.00000000
H	-6.19245483	0.04963480	-0.88575940

Yss

Total bonding energy = -4301.78 kcal mol^{-1}

H	-0.27504289	7.07871695	0.00000000
H	3.33688593	1.67120252	0.00000000
H	2.13565882	6.29726878	0.00000000
C	-2.60936512	2.15688872	0.00000000
C	-3.61868515	1.16660959	0.00000000
C	-3.12766278	-0.13189242	0.00000000
S	-1.37209608	-0.11566501	0.00000000
C	-1.31500205	1.65264116	0.00000000
H	1.16857617	0.53741581	0.00000000
H	-2.83500182	3.21403638	0.00000000
H	-4.68008789	1.39484570	0.00000000
C	-3.85395253	-1.37946848	0.00000000
C	1.18044839	1.62385942	0.00000000
C	2.42423165	2.26482618	0.00000000
N	2.58461963	3.60501643	0.00000000
C	1.43515077	4.28981092	0.00000000
C	0.09645198	3.76335230	0.00000000
C	-0.03475820	2.34518230	0.00000000
N	1.34198467	5.66783043	0.00000000
C	0.00820788	6.03517434	0.00000000
C	-0.78238223	4.90264029	0.00000000
C	-3.36275620	-2.67757855	0.00000000
C	-4.38221389	-3.67085089	0.00000000
C	-5.65064321	-3.13657736	0.00000000
S	-5.61640744	-1.39888217	0.00000000
H	-2.30106773	-2.90456855	0.00000000
H	-4.18632889	-4.73875964	0.00000000
H	-6.60249638	-3.65206983	0.00000000
H	-1.86124190	4.91995874	0.00000000

Px

Total bonding energy = -2445.01 kcal mol^{-1}

C	-1.36553127	-3.84091555	0.00000000
H	-0.98979924	-4.36691712	0.88724034
H	-2.45999086	-3.91866620	0.00000000
H	-0.98979924	-4.36691712	-0.88724034
C	-1.02121471	1.20112247	0.00000000
C	-0.17140255	0.07055919	0.00000000
C	1.16529221	0.55733223	0.00000000
C	1.08080176	1.94795620	0.00000000
N	-0.25027629	2.31808251	0.00000000
O	3.29148634	2.54677676	0.00000000
H	2.07400724	-0.02585562	0.00000000
H	-0.56762821	3.28185908	0.00000000
H	-2.10016635	1.25602603	0.00000000
N	2.09394808	2.92641840	0.00000000
O	1.74349599	4.14033760	0.00000000
C	-0.59032398	-1.28269774	0.00000000
C	-0.94289900	-2.44450115	0.00000000

Cartesian Coordinates and Total Bonding Energies of Ds–Px variants in Chloroform

Cartesian coordinates (Å) and total bonding energies (kcal mol^{-1}) of Ds–Px variants optimized at ZORA-BLYP-D3(BJ)/TZ2P level of theory in chloroform.

Ds–Px

Total bonding energy = -5620.91 kcal mol^{-1}

H	6.91054344	-0.95093337	0.00000000
H	1.97896375	3.25448603	0.00000000
H	6.54424753	1.57678398	0.00000000
C	2.10764389	-2.70841080	0.00000000
C	1.07446416	-3.68569927	0.00000000
C	-0.18639397	-3.13235330	0.00000000
S	-0.12890374	-1.40165616	0.00000000
C	1.63494384	-1.40336538	0.00000000
H	0.68845159	1.19201102	0.00000000
H	3.16770759	-2.93141477	0.00000000
H	1.25351269	-4.75650577	0.00000000
H	-1.14278571	-3.63740561	0.00000000
C	1.77037877	1.11124166	0.00000000
C	2.50597258	2.30301079	0.00000000
N	3.85751610	2.36587541	0.00000000
C	4.43797664	1.16275441	0.00000000
C	3.80603837	-0.11346757	0.00000000
C	2.39043756	-0.15884061	0.00000000
N	5.79829328	0.88957503	0.00000000
C	5.93639480	-0.47834974	0.00000000
N	4.77540203	-1.11894669	0.00000000
C	-4.73016354	1.73032848	0.00000000
C	-3.68072514	0.78206698	0.00000000
C	-2.46330287	1.51559971	0.00000000
C	-2.80825235	2.86366817	0.00000000
N	-4.18542460	2.97449944	0.00000000
O	-0.74370132	3.85121318	0.00000000
H	-1.46366841	1.11554869	0.00000000
H	-4.68144802	3.85961701	0.00000000
H	-5.79994752	1.57955381	0.00000000
N	-1.99019130	4.01027127	0.00000000
O	-2.55434101	5.14033101	0.00000000
C	-3.82414854	-0.62715059	0.00000000
C	-3.92867383	-1.83657259	0.00000000
C	-4.05448070	-3.28966420	0.00000000
H	-3.58170004	-3.72772400	0.88873742
H	-5.10893596	-3.59225167	0.00000000
H	-3.58170004	-3.72772400	-0.88873742

Bs–Px

Total bonding energy = -5722.38 kcal mol^{-1}

H	-1.06134022	6.80239332	0.00000000
H	3.20770742	1.74032225	0.00000000
H	1.46511932	6.50943749	0.00000000
C	-2.74970010	2.03625404	0.00000000

C	-3.73843146	1.01037546	0.00000000
C	-3.20042094	-0.25508802	0.00000000
S	-1.46574234	-0.21061139	0.00000000
C	-1.44831864	1.55616949	0.00000000
H	1.08825547	0.55442477	0.00000000
H	-2.96456074	3.09759821	0.00000000
H	-4.80743794	1.20172339	0.00000000
H	-3.71312391	-1.20714990	0.00000000
C	1.06052119	1.63968951	0.00000000
C	2.28955385	2.32105165	0.00000000
C	2.35006957	3.71664065	0.00000000
C	1.12295034	4.38494632	0.00000000
C	-0.13534529	3.71683306	0.00000000
C	-0.18751093	2.29575099	0.00000000
N	0.80736935	5.73854085	0.00000000
C	-0.56263086	5.84117390	0.00000000
N	-1.16736405	4.66339749	0.00000000
C	1.57641375	-4.87834451	0.00000000
C	0.65277554	-3.80700991	0.00000000
C	1.41434656	-2.60671253	0.00000000
C	2.75417435	-2.98324512	0.00000000
N	2.83270884	-4.36294251	0.00000000
O	3.79450644	-0.94442119	0.00000000
H	1.03551577	-1.59868457	0.00000000
H	3.70651955	-4.87859150	0.00000000
H	1.40031181	-5.94427912	0.00000000
N	3.92079033	-2.19462805	0.00000000
O	5.03656366	-2.78775437	0.00000000
C	-0.75912835	-3.92043299	0.00000000
C	-1.97034206	-4.00276673	0.00000000
C	-3.42538641	-4.10153985	0.00000000
H	-3.85466035	-3.62039496	0.88853706
H	3.29752979	4.24848107	0.00000000
H	-3.85466035	-3.62039496	-0.88853706
H	-3.74759814	-5.15021172	0.00000000

Is–Px

Total bonding energy = -5817.85 kcal mol^{-1}

H	-0.77980146	6.91185744	0.00000000
H	3.20630976	1.58437694	0.00000000
H	1.67792590	6.34406653	0.00000000
C	-2.75324006	1.88288697	0.00000000
C	-3.71363740	0.82952762	0.00000000
C	-3.14030729	-0.41859243	0.00000000
S	-1.40814780	-0.32042361	0.00000000
C	-1.43489263	1.45020919	0.00000000
H	1.06303909	0.44011823	0.00000000
H	-3.03997007	2.92450890	0.00000000
H	-4.78706763	0.99387208	0.00000000
H	-3.62431092	-1.38568318	0.00000000
C	1.05548504	1.52569997	0.00000000
C	2.29799214	2.18038416	0.00000000

C	2.37399570	3.57277780	0.00000000
C	1.16360525	4.27251557	0.00000000
C	-0.12436541	3.63427418	0.00000000
C	-0.17831818	2.20540649	0.00000000
N	0.94981867	5.64129811	0.00000000
C	-0.40793754	5.89616507	0.00000000
C	-1.09684398	4.70004867	0.00000000
C	1.63672421	-4.99656011	0.00000000
C	0.71234334	-3.92548389	0.00000000
C	1.47318015	-2.72480702	0.00000000
C	2.81368563	-3.10129878	0.00000000
N	2.89251332	-4.48081776	0.00000000
O	3.85469085	-1.06302706	0.00000000
H	1.09895493	-1.71430473	0.00000000
H	3.76591756	-4.99718133	0.00000000
H	1.46082300	-6.06251278	0.00000000
N	3.98032370	-2.31314662	0.00000000
O	5.09589654	-2.90688084	0.00000000
C	-0.69928522	-4.04296245	0.00000000
C	-1.90943354	-4.13730013	0.00000000
C	-3.36304090	-4.25381818	0.00000000
H	3.32914725	4.09226919	0.00000000
H	-3.79889859	-3.77840125	-0.88846846
H	-3.79889859	-3.77840125	0.88846846
H	-2.17252999	4.62604725	0.00000000
H	-3.67144490	-5.30670704	0.00000000

Ys–Px

Total bonding energy = -5717.45 kcal mol^{-1}

H	-0.71040800	7.03910938	0.00000000
H	3.31248942	1.92919059	0.00000000
H	1.75381853	6.44927623	0.00000000
C	-2.65388617	1.95440684	0.00000000
C	-3.58689411	0.87969311	0.00000000
C	-2.97994855	-0.35411742	0.00000000
S	-1.25380770	-0.21823833	0.00000000
C	-1.32710140	1.54816974	0.00000000
H	1.24404273	0.63523546	0.00000000
H	-2.96022771	2.99093329	0.00000000
H	-4.66394283	1.01555492	0.00000000
H	-3.44143823	-1.33151154	0.00000000
C	1.16662497	1.71751981	0.00000000
C	2.35822587	2.45196225	0.00000000
N	2.41207641	3.80017860	0.00000000
C	1.21319157	4.39293056	0.00000000
C	-0.08063064	3.76302850	0.00000000
C	-0.09959295	2.34026690	0.00000000
N	1.01210532	5.75934802	0.00000000
C	-0.34648414	6.02083691	0.00000000
C	-1.04602101	4.83013958	0.00000000
C	1.68764596	-4.92205154	0.00000000
C	0.78211021	-3.83526649	0.00000000

C	1.56281425	-2.64765283	0.00000000
C	2.89632367	-3.04720741	0.00000000
N	2.95210830	-4.42752444	0.00000000
O	3.97456883	-1.02808107	0.00000000
H	1.20255543	-1.63293294	0.00000000
H	3.81726456	-4.95763922	0.00000000
H	1.49335245	-5.98478063	0.00000000
N	4.07777564	-2.27961448	0.00000000
O	5.18185338	-2.89392605	0.00000000
C	-0.63093021	-3.93607697	0.00000000
C	-1.84078798	-4.03444124	0.00000000
C	-3.29420807	-4.15960242	0.00000000
H	-3.73272112	-3.68785038	0.88894520
H	-2.12285020	4.76272952	0.00000000
H	-3.73272112	-3.68785038	-0.88894520
H	-3.59634522	-5.21414457	0.00000000

Yi–Px

Total bonding energy = -5806.81 mol^{-1}

H	-6.71624870	-0.85276307	0.00000000
H	-0.48296288	-2.70845271	0.00000000
H	-5.26691126	-2.93012515	0.00000000
N	-2.83552772	2.69939939	0.00000000
C	-2.15817384	3.90135077	0.00000000
C	-0.79424688	3.68398600	0.00000000
N	-0.63970904	2.31305399	0.00000000
C	-1.89831580	1.73920797	0.00000000
H	-0.04865759	-0.28310424	0.00000000
H	-5.07094593	1.31326968	0.00000000
H	-2.68057679	4.84945479	0.00000000
H	0.04696317	4.35952727	0.00000000
C	-1.07830659	-0.62689031	0.00000000
C	-1.31754915	-2.00879494	0.00000000
N	-2.54836198	-2.56220581	0.00000000
C	-3.54983418	-1.67505624	0.00000000
C	-3.44463198	-0.24202530	0.00000000
C	-2.13674875	0.30229128	0.00000000
N	-4.89407915	-1.98828758	0.00000000
C	-5.63538263	-0.81498359	0.00000000
C	-4.78461285	0.27267000	0.00000000
C	4.80218740	-1.15897793	0.00000000
C	3.49747308	-0.61196315	0.00000000
C	2.59223277	-1.70749611	0.00000000
C	3.36499662	-2.86433600	0.00000000
N	4.70032769	-2.51258337	0.00000000
O	1.74611762	-4.48209066	0.00000000
H	1.51628641	-1.67082225	0.00000000
H	5.46155162	-3.18366596	0.00000000
H	5.76126867	-0.66179611	0.00000000
N	2.97385041	-4.21867664	0.00000000
O	3.88206097	-5.09548506	0.00000000
C	3.19254780	0.77217092	0.00000000

C	3.00313145	1.97217896	0.00000000
C	2.82764719	3.42125966	0.00000000
H	2.28059816	3.75657061	-0.89017486
H	0.24970688	1.82316461	0.00000000
H	2.28059816	3.75657061	0.89017486
H	3.80223764	3.92445560	0.00000000

Do–Px

Total bonding energy = -5679.68 kcal mol^{-1}

H	1.10181453	6.92620736	0.00000000
H	-3.49711533	2.35792672	0.00000000
H	-1.44638751	6.77484467	0.00000000
C	2.46212582	1.91759332	0.00000000
C	3.13544703	0.65454446	0.00000000
C	2.16327163	-0.30790257	0.00000000
O	0.91513466	0.27496183	0.00000000
C	1.10858208	1.65755830	0.00000000
H	-1.53710479	0.89404791	0.00000000
H	2.90169244	2.90455579	0.00000000
H	4.20425917	0.48474864	0.00000000
H	2.16906988	-1.38851319	0.00000000
C	-1.37898845	1.96606312	0.00000000
C	-2.50395164	2.80246591	0.00000000
N	-2.45810327	4.15517661	0.00000000
C	-1.21237289	4.63946635	0.00000000
C	0.00559529	3.90618032	0.00000000
C	-0.06901364	2.49398712	0.00000000
N	-0.82582239	5.97263894	0.00000000
C	0.54950909	5.99495280	0.00000000
N	1.09057635	4.78360535	0.00000000
C	-1.41745445	-4.73629622	0.00000000
C	-0.44668004	-3.70787798	0.00000000
C	-1.15023443	-2.47117945	0.00000000
C	-2.50658846	-2.78939398	0.00000000
N	-2.64774249	-4.16423431	0.00000000
O	-3.47295131	-0.71243588	0.00000000
H	-0.72532380	-1.47880025	0.00000000
H	-3.54481740	-4.63837756	0.00000000
H	-1.28918961	-5.80901097	0.00000000
N	-3.64299796	-1.95704165	0.00000000
O	-4.77979908	-2.50880922	0.00000000
C	0.95770169	-3.89436079	0.00000000
C	2.16282818	-4.04304894	0.00000000
C	3.61272967	-4.20495705	0.00000000
H	4.06189081	-3.74128365	-0.88789533
H	4.06189081	-3.74128365	0.88789533
H	3.88851969	-5.26671822	0.00000000

Dp–Px

Total bonding energy = -5880.99 kcal mol^{-1}

H	0.87887244	6.96927681	0.00000000
H	-3.62958625	2.31661131	0.00000000

H	-1.66490622	6.76093267	0.00000000
C	0.98872242	1.62366717	0.00000000
N	0.73859622	0.29185426	0.00000000
H	3.94912062	-4.00708410	0.88722551
H	3.94912062	-4.00708410	-0.88722551
N	1.74525791	-0.59294485	0.00000000
H	-1.62904325	0.88744188	0.00000000
C	3.01359114	-0.15088281	0.00000000
C	3.34591058	1.20748356	0.00000000
C	2.30786836	2.12371697	0.00000000
C	-1.50215299	1.96251246	0.00000000
C	-2.64343792	2.77586934	0.00000000
N	-2.61607236	4.12842370	0.00000000
C	-1.38245617	4.63284232	0.00000000
C	-0.14619308	3.91895133	0.00000000
C	-0.20296244	2.50681771	0.00000000
N	-1.02570765	5.97337648	0.00000000
C	0.34635333	6.02660232	0.00000000
N	0.91547308	4.82877429	0.00000000
C	-1.55882166	-4.82593678	0.00000000
C	-0.54741670	-3.83683007	0.00000000
C	-1.19915115	-2.57043452	0.00000000
C	-2.56873994	-2.83714732	0.00000000
N	-2.76524973	-4.20661549	0.00000000
O	-3.47850956	-0.73122235	0.00000000
H	-0.72806140	-1.59572187	0.00000000
H	-3.68118915	-4.64273714	0.00000000
H	-1.47334123	-5.90308372	0.00000000
N	-3.67855524	-1.97198666	0.00000000
O	-4.83299997	-2.49108048	0.00000000
C	0.84693789	-4.08606883	0.00000000
C	2.04560835	-4.27447698	0.00000000
C	3.49169344	-4.46468264	0.00000000
H	4.38499018	1.52592166	0.00000000
H	2.47950565	3.19390705	0.00000000
H	3.77485985	-0.92828989	0.00000000
H	3.75207187	-5.53067279	0.00000000

Dss–Px

Total bonding energy = -6652.98 kcal mol^{-1}

H	-7.28929770	2.97992192	0.00000000
H	-1.03790870	4.69490162	0.00000000
H	-5.88538222	5.11327914	0.00000000
C	-3.67942439	-0.64418370	0.00000000
C	-3.16818391	-1.96073648	0.00000000
C	-1.77978990	-2.02483934	0.00000000
S	-1.09606483	-0.40591474	0.00000000
C	-2.69865916	0.33909664	0.00000000
H	-0.74240268	2.27758076	0.00000000
H	-4.73363396	-0.39476435	0.00000000
H	-3.79542119	-2.84692675	0.00000000
H	-0.00210150	-6.63578742	0.00000000

C	-1.75558394	2.66630422	0.00000000
C	-1.91890569	4.05702865	0.00000000
N	-3.11641515	4.68838480	0.00000000
C	-4.15291985	3.84472467	0.00000000
C	-4.12241773	2.42181116	0.00000000
C	-2.85760904	1.78072585	0.00000000
N	-5.50105045	4.17470079	0.00000000
C	-6.20672624	2.99476461	0.00000000
N	-5.42636892	1.92232490	0.00000000
C	5.27547964	0.88254465	0.00000000
C	4.16866283	0.00088273	0.00000000
C	2.99664296	0.80700330	0.00000000
C	3.42921659	2.13246222	0.00000000
N	4.81016285	2.15614901	0.00000000
O	1.44703410	3.27904654	0.00000000
H	1.96899694	0.47542938	0.00000000
H	5.35851299	3.01001894	0.00000000
H	6.33319644	0.66284080	0.00000000
N	2.70092611	3.33807721	0.00000000
O	3.35178463	4.42129641	0.00000000
C	4.26293752	-1.41251721	0.00000000
C	4.40942083	-2.61768016	0.00000000
C	4.61442789	-4.06161695	0.00000000
C	-0.93327274	-3.19297047	0.00000000
C	0.45226964	-3.28226347	0.00000000
C	0.93156950	-4.62198577	0.00000000
C	-0.07887681	-5.55601769	0.00000000
S	-1.64502949	-4.80709171	0.00000000
H	4.17043363	-4.52706342	-0.88914528
H	4.17043363	-4.52706342	0.88914528
H	1.10430700	-2.41423080	0.00000000
H	1.98124684	-4.88889229	0.00000000
H	5.68578364	-4.29875480	0.00000000

Dt–Px

Total bonding energy = -5518.48 kcal mol^{-1}

H	-0.97231639	7.01056708	0.00000000
H	3.15236966	2.01375631	0.00000000
H	1.54896762	6.59874471	0.00000000
N	-2.71703555	2.21726087	0.00000000
C	-3.67617673	1.23266613	0.00000000
C	-3.20699021	-0.05879468	0.00000000
S	-1.48086699	-0.06758948	0.00000000
C	-1.49753800	1.71422917	0.00000000
H	1.06800777	0.75457548	0.00000000
H	-3.82130227	-3.43975501	-0.88881576
H	-4.72582834	1.50850158	0.00000000
H	-3.77294142	-0.98009739	0.00000000
C	1.00554566	1.83741819	0.00000000
C	2.20825173	2.55318609	0.00000000
N	2.28559284	3.90324385	0.00000000
C	1.09419100	4.50268146	0.00000000

C	-0.19882879	3.89289461	0.00000000
C	-0.25191018	2.47893275	0.00000000
N	0.84762448	5.86600354	0.00000000
C	-0.51795396	6.02768263	0.00000000
N	-1.18216886	4.88035213	0.00000000
C	1.62117596	-4.67062139	0.00000000
C	0.68438597	-3.61081585	0.00000000
C	1.43150551	-2.40181780	0.00000000
C	2.77554086	-2.76109311	0.00000000
N	2.87116411	-4.13951049	0.00000000
O	3.78356462	-0.70660632	0.00000000
H	1.04235587	-1.39827363	0.00000000
H	3.75123679	-4.64452402	0.00000000
H	1.45835595	-5.73864425	0.00000000
N	3.93031972	-1.95471665	0.00000000
O	5.05448561	-2.53011967	0.00000000
C	-0.72629875	-3.73783624	0.00000000
C	-1.93723371	-3.82242605	0.00000000
C	-3.39236748	-3.92068096	0.00000000
H	-3.82130227	-3.43975501	0.88881576
H	-3.71558167	-4.96901841	0.00000000

Yo–Px

Total bonding energy = -5776.70 kcal mol^{-1}

H	0.86649428	7.02542820	0.00000000
H	-3.52316152	2.22305030	0.00000000
H	-1.63574334	6.62459037	0.00000000
C	2.43403509	1.75854030	0.00000000
C	3.07601927	0.47863503	0.00000000
C	2.07982282	-0.45716066	0.00000000
O	0.84894567	0.15999135	0.00000000
C	1.07229471	1.53903124	0.00000000
H	-1.54729319	0.77479971	0.00000000
H	2.92818033	2.71786662	0.00000000
H	4.14041536	0.28350792	0.00000000
H	2.05882130	-1.53765960	0.00000000
C	-1.39972505	1.84811216	0.00000000
C	-2.53237240	2.67355825	0.00000000
N	-2.49090842	4.02296435	0.00000000
C	-1.25301037	4.53167103	0.00000000
C	-0.01212670	3.81019097	0.00000000
C	-0.09882211	2.39251836	0.00000000
N	-0.94904648	5.87991867	0.00000000
C	0.42635220	6.03765572	0.00000000
C	1.03383897	4.79666835	0.00000000
C	-1.46740297	-4.87203391	0.00000000
C	-0.49168846	-3.84823824	0.00000000
C	-1.18948768	-2.60808705	0.00000000
C	-2.54702742	-2.91993609	0.00000000
N	-2.69492466	-4.29411834	0.00000000
O	-3.50094568	-0.83779924	0.00000000
H	-0.76217102	-1.61672830	0.00000000

H	-3.59458758	-4.76340720	0.00000000
H	-1.34404927	-5.94534302	0.00000000
N	-3.67864436	-2.08102687	0.00000000
O	-4.81856854	-2.62657546	0.00000000
C	0.91182058	-4.04114415	0.00000000
C	2.11632036	-4.19318265	0.00000000
C	3.56559356	-4.35906675	0.00000000
H	4.01650965	-3.89660081	0.88775433
H	4.01650965	-3.89660081	-0.88775433
H	2.10184527	4.63783967	0.00000000
H	3.83788799	-5.42182956	0.00000000

Ps–Px

Total bonding energy = -5520.53 kcal mol^{-1}

H	-0.49967676	6.82289288	0.00000000
H	2.83269219	1.28114487	0.00000000
H	1.94970708	6.09442593	0.00000000
C	-2.89073040	2.18180278	0.00000000
C	-3.93400909	1.21795771	0.00000000
C	-3.45943171	-0.07764385	0.00000000
S	-1.72852112	-0.14621418	0.00000000
C	-1.62679482	1.60799568	0.00000000
H	-3.57778785	-3.36565108	-0.88870847
H	-3.03931425	3.25536295	0.00000000
H	-4.99077874	1.46670072	0.00000000
H	-4.03493954	-0.99407357	0.00000000
N	0.77350068	1.43385055	0.00000000
C	2.00138515	1.98100168	0.00000000
N	2.31787435	3.29428974	0.00000000
C	1.23135690	4.07477394	0.00000000
C	-0.11796674	3.63633702	0.00000000
C	-0.32514861	2.24093797	0.00000000
N	1.16290651	5.45388790	0.00000000
C	-0.17644172	5.78968446	0.00000000
N	-0.97840065	4.73603733	0.00000000
C	1.84462941	-4.46837225	0.00000000
C	0.93662444	-3.38344600	0.00000000
C	1.71148618	-2.18813930	0.00000000
C	3.04809547	-2.59163163	0.00000000
N	3.10571058	-3.97266015	0.00000000
O	4.17254504	-0.59226669	0.00000000
H	1.34984204	-1.16766840	0.00000000
H	3.97325373	-4.49879110	0.00000000
H	1.65071007	-5.53122255	0.00000000
N	4.24297420	-1.84643229	0.00000000
O	5.33557912	-2.48274540	0.00000000
C	-0.47370843	-3.51674980	0.00000000
C	-1.67852121	-3.66385848	0.00000000
C	-3.12768053	-3.82699630	0.00000000
H	-3.57778785	-3.36565108	0.88870847
H	-3.40323295	-4.88887008	0.00000000

Es–Px

Total bonding energy = -5618.71 kcal mol^{-1}

H	-0.26058850	6.87149071	0.00000000
H	2.86185453	1.17715105	0.00000000
H	2.09178026	5.93294415	0.00000000
C	-2.87254909	1.99576389	0.00000000
C	-3.87804510	0.99065611	0.00000000
C	-3.35244996	-0.28357097	0.00000000
S	-1.61967277	-0.27898745	0.00000000
C	-1.58480876	1.47953839	0.00000000
H	-1.99340184	4.81340549	0.00000000
H	-3.09551734	3.05491009	0.00000000
H	-4.94327485	1.20047470	0.00000000
H	-3.89045131	-1.22253019	0.00000000
N	0.80046681	1.33035567	0.00000000
C	2.02935324	1.87583404	0.00000000
N	2.33790643	3.18729035	0.00000000
C	1.25846050	3.98004824	0.00000000
C	-0.10785207	3.54593280	0.00000000
C	-0.29194025	2.14309837	0.00000000
N	1.26027840	5.35368251	0.00000000
C	-0.05257853	5.81052823	0.00000000
C	-0.91659535	4.73596041	0.00000000
C	1.88585069	-4.58725932	0.00000000
C	0.97510283	-3.50447332	0.00000000
C	1.74723860	-2.30748256	0.00000000
C	3.08487040	-2.70808914	0.00000000
N	3.14579031	-4.08876665	0.00000000
O	4.20458529	-0.70623603	0.00000000
H	1.38749673	-1.28632809	0.00000000
H	4.01359869	-4.61438138	0.00000000
H	1.69452055	-5.65059431	0.00000000
N	4.27814447	-1.96004992	0.00000000
O	5.37211461	-2.59420158	0.00000000
C	-0.43464779	-3.64701176	0.00000000
C	-1.63664410	-3.81642654	0.00000000
C	-3.08160294	-4.01365847	0.00000000
H	-3.54305145	-3.56349007	0.88866381
H	-3.54305145	-3.56349007	-0.88866381
H	-3.33069002	-5.08203739	0.00000000

Ysm–Px

Total bonding energy = -6087.00 kcal mol^{-1}

H	0.17292139	7.39826986	0.00000000
H	-3.66038781	2.14272605	0.00000000
H	-2.26849742	6.71912695	0.00000000
C	2.29971884	2.37782977	0.00000000
C	3.26587977	1.33196594	0.00000000
C	2.71876088	0.06798259	0.00000000
S	0.97480314	0.16496805	0.00000000
C	0.98689503	1.93479849	0.00000000
H	-1.55097899	0.92472363	0.00000000

H	2.57479768	3.42355398	0.00000000
H	4.33863747	1.50444637	0.00000000
C	3.41577009	-1.25984160	0.00000000
C	-1.50959364	2.00939156	0.00000000
C	-2.72655145	2.70132017	0.00000000
N	-2.83087715	4.04712426	0.00000000
C	-1.65375390	4.68335971	0.00000000
C	-0.33878029	4.10122944	0.00000000
C	-0.26711606	2.67975309	0.00000000
N	-1.50223662	6.05663089	0.00000000
C	-0.15378503	6.36742333	0.00000000
C	0.58801156	5.20241020	0.00000000
C	-2.02928039	-4.92052314	0.00000000
C	-0.96103904	-3.99330326	0.00000000
C	-1.54076899	-2.69487767	0.00000000
C	-2.92132446	-2.87558805	0.00000000
N	-3.19785013	-4.22939217	0.00000000
O	-3.66503038	-0.70976119	0.00000000
H	-1.02130054	-1.75013276	0.00000000
H	-4.13700248	-4.61343581	0.00000000
H	-2.00878845	-6.00069264	0.00000000
N	-3.96505999	-1.92956062	0.00000000
O	-5.15305196	-2.36006751	0.00000000
C	0.41881396	-4.31432375	0.00000000
C	1.60170365	-4.58787254	0.00000000
C	3.02193291	-4.92073695	0.00000000
H	3.52391740	-4.51379182	-0.88703420
H	3.15064617	-1.85385241	0.88307911
H	4.49986897	-1.10838896	0.00000000
H	3.15064617	-1.85385241	-0.88307911
H	1.66669072	5.17273153	0.00000000
H	3.52391740	-4.51379182	0.88703420
H	3.16872180	-6.00797875	0.00000000

Yss–Px
Total bonding energy = -6749.44 kcal mol^{-1}

H	-7.21776171	3.43597115	0.00000000
H	-0.82345943	4.63468745	0.00000000
H	-5.55336779	5.34738485	0.00000000
C	-3.60011696	-0.63814418	0.00000000
C	-3.08580406	-1.95491572	0.00000000
C	-1.69902290	-2.01464699	0.00000000
S	-1.02398456	-0.39270666	0.00000000
C	-2.62925411	0.35441973	0.00000000
H	-0.63229989	2.20588048	0.00000000
H	-4.66082079	-0.43014090	0.00000000
H	-3.71320149	-2.84096052	0.00000000
C	-0.84594571	-3.17832771	0.00000000
C	-1.62795907	2.63705189	0.00000000
C	-1.72967996	4.03274639	0.00000000
N	-2.90060965	4.70497298	0.00000000
C	-3.98094345	3.91567747	0.00000000

C	-4.02177671	2.47802630	0.00000000
C	-2.76708855	1.80348021	0.00000000
N	-5.28575253	4.37040342	0.00000000
C	-6.14662951	3.28778852	0.00000000
C	-5.41448922	2.11626125	0.00000000
C	5.42706202	0.72876905	0.00000000
C	4.32360669	-0.15688185	0.00000000
C	3.14862619	0.64525558	0.00000000
C	3.57638882	1.97218474	0.00000000
N	4.95716605	2.00075959	0.00000000
O	1.58994005	3.11127498	0.00000000
H	2.12169543	0.31161117	0.00000000
H	5.50229019	2.85666121	0.00000000
H	6.48559392	0.51301061	0.00000000
N	2.84326488	3.17512726	0.00000000
O	3.49048274	4.26058211	0.00000000
C	4.42354226	-1.57000966	0.00000000
C	4.57501843	-2.77458830	0.00000000
C	4.78413654	-4.21817341	0.00000000
C	0.53971075	-3.25817996	0.00000000
C	1.02923633	-4.59440144	0.00000000
C	0.02609391	-5.53579484	0.00000000
S	-1.54586436	-4.79736375	0.00000000
H	1.18626934	-2.38626169	0.00000000
H	4.34079930	-4.68485525	-0.88897279
H	4.34079930	-4.68485525	0.88897279
H	2.08151257	-4.85115362	0.00000000
H	0.10979727	-6.61503436	0.00000000
H	-5.85314031	1.13037982	0.00000000
H	5.85593978	-4.45297212	0.00000000

Cartesian Coordinates and Total Bonding Energies of Ds–Px//A–T variants in Chloroform

Cartesian coordinates (Å) and total bonding energies (kcal mol^{-1}) of Ds–Px//A–T variants optimized at ZORA-BLYP-D3(BJ)-TZ2P level of theory in chloroform.

Ds–Px//A–T
Total bonding energy = -10139.39 kcal mol^{-1}

H	0.72856716	-1.60276921	3.40000000
H	7.16092660	-0.80855098	3.40000000
H	2.83953137	-3.04060014	3.40000000
C	2.67803974	3.12545749	3.40000000
C	2.65564222	4.54744604	3.40000000
C	3.91548548	5.10309876	3.40000000
S	5.15253950	3.89135824	3.40000000
C	3.95952331	2.59217636	3.40000000
H	6.51238063	1.53636116	3.40000000
H	1.79739808	2.49463573	3.40000000
H	1.74525108	5.13894660	3.40000000
H	4.18934567	6.14940827	3.40000000
C	5.72182439	0.79331947	3.40000000
C	6.10340654	-0.55419935	3.40000000
N	5.23657138	-1.59305384	3.40000000
C	3.95741900	-1.20808143	3.40000000
C	3.44354125	0.12008203	3.40000000
C	4.36655224	1.19434228	3.40000000
N	2.83691919	-2.02635310	3.40000000
C	1.73515383	-1.20391568	3.40000000
N	2.04732848	0.08481069	3.40000000
C	10.57036263	5.16733316	3.40000000
C	9.16222855	5.03436994	3.40000000
C	8.88044065	3.64125012	3.40000000
C	10.10732591	2.98472039	3.40000000
N	11.11952870	3.92510994	3.40000000
O	9.44043139	0.79545846	3.40000000
H	7.91010651	3.17460086	3.40000000
H	12.10719331	3.69275040	3.40000000
H	11.18201626	6.05786704	3.40000000
N	10.39989136	1.60692215	3.40000000
O	11.61416129	1.25928949	3.40000000
C	8.22023578	6.09225181	3.40000000
C	7.39925373	6.98646408	3.40000000
C	6.41301359	8.06100258	3.40000000
H	5.77063127	8.00843930	4.28873742
H	6.90239143	9.04280928	3.40000000
H	5.77063127	8.00843930	2.51126258
N	4.58537740	1.43284602	-0.00000000
C	4.10904668	0.17080601	-0.00000000
N	2.82642353	-0.22674809	-0.00000000
C	1.97976219	0.82481149	-0.00000000
C	2.33461211	2.18515357	-0.00000000
C	3.71866132	2.48507436	-0.00000000

N	0.59797841	0.81945862	-0.00000000
C	0.18598755	2.13955201	-0.00000000
N	1.19868269	2.99352291	-0.00000000
N	4.21489056	3.73651739	-0.00000000
H	4.86889014	-0.60940868	-0.00000000
H	0.00000000	-0.00000000	-0.00000000
H	-0.86304656	2.40603390	-0.00000000
H	3.58117462	4.52464620	-0.00000000
H	5.23095585	3.88977344	-0.00000000
N	7.41599982	1.76861079	-0.00000000
C	8.16514377	0.60711280	-0.00000000
N	9.53746234	0.82769201	-0.00000000
C	10.10599054	2.08184011	-0.00000000
C	9.35810649	3.21757575	-0.00000000
C	7.90650249	3.07549030	-0.00000000
O	7.11247358	4.04132194	-0.00000000
O	7.67068695	-0.52685707	-0.00000000
C	9.95641794	4.59798964	-0.00000000
H	6.36849540	1.64290197	-0.00000000
H	10.12459787	0.00000000	-0.00000000
H	11.19140414	2.09630006	-0.00000000
H	9.63126266	5.16475309	-0.88121399
H	11.04966811	4.54779765	-0.00000000
H	9.63126266	5.16475309	0.88121399

Bs–Px//A–T

Total bonding energy = -10240.57 kcal mol^{-1}

H	0.69853704	1.56922549	-3.40000000
H	7.27116835	0.76309341	-3.40000000
H	2.74960871	3.07319555	-3.40000000
C	2.70334271	-3.07269189	-3.40000000
C	2.67652945	-4.49722606	-3.40000000
C	3.93173304	-5.05875707	-3.40000000
S	5.17291745	-3.84609500	-3.40000000
C	3.98378707	-2.53927168	-3.40000000
H	6.52444535	-1.54793107	-3.40000000
H	1.82385110	-2.44093952	-3.40000000
H	1.76282643	-5.08419768	-3.40000000
H	4.20361120	-6.10535557	-3.40000000
C	5.76583627	-0.77134817	-3.40000000
C	6.20314626	0.56414261	-3.40000000
C	5.29811384	1.62821363	-3.40000000
C	3.94405775	1.28326990	-3.40000000
C	3.47628641	-0.06241699	-3.40000000
C	4.40478142	-1.13949290	-3.40000000
N	2.79193136	2.06071076	-3.40000000
C	1.71796430	1.20395591	-3.40000000
N	2.07593697	-0.07068720	-3.40000000
C	10.57803361	-5.19782186	-3.40000000
C	9.17224388	-5.04091235	-3.40000000
C	8.91390572	-3.64306914	-3.40000000
C	10.15208544	-3.00759587	-3.40000000

N	11.14822467	-3.96542633	-3.40000000
O	9.52763584	-0.80551581	-3.40000000
H	7.95050124	-3.16194034	-3.40000000
H	12.13947272	-3.74893998	-3.40000000
H	11.17409012	-6.09890183	-3.40000000
N	10.47068373	-1.63595274	-3.40000000
O	11.69198550	-1.31165206	-3.40000000
C	8.21454143	-6.08453268	-3.40000000
C	7.38278641	-6.96884094	-3.40000000
C	6.38349792	-8.03107100	-3.40000000
H	5.74154755	-7.97043896	-2.51146294
H	5.63076024	2.66256438	-3.40000000
H	5.74154755	-7.97043896	-4.28853706
H	6.86071913	-9.01889336	-3.40000000
N	4.58537740	-1.43284602	0.00000000
C	4.10904668	-0.17080601	0.00000000
N	2.82642353	0.22674809	0.00000000
C	1.97976219	-0.82481149	0.00000000
C	2.33461211	-2.18515357	0.00000000
C	3.71866132	-2.48507436	0.00000000
N	0.59797841	-0.81945862	0.00000000
C	0.18598755	-2.13955201	0.00000000
N	1.19868269	-2.99352291	0.00000000
N	4.21489056	-3.73651739	0.00000000
H	4.86889014	0.60940868	0.00000000
H	0.00000000	0.00000000	0.00000000
H	-0.86304656	-2.40603390	0.00000000
H	3.58117462	-4.52464620	0.00000000
H	5.23095585	-3.88977344	0.00000000
N	7.41599982	-1.76861079	0.00000000
C	8.16514377	-0.60711280	0.00000000
N	9.53746234	-0.82769201	0.00000000
C	10.10599054	-2.08184011	0.00000000
C	9.35810649	-3.21757575	0.00000000
C	7.90650249	-3.07549030	0.00000000
O	7.11247358	-4.04132194	0.00000000
O	7.67068695	0.52685707	0.00000000
C	9.95641794	-4.59798964	0.00000000
H	6.36849540	-1.64290197	0.00000000
H	10.12459787	-0.00000000	0.00000000
H	11.19140414	-2.09630006	0.00000000
H	9.63126266	-5.16475309	0.88121399
H	11.04966811	-4.54779765	0.00000000
H	9.63126266	-5.16475309	-0.88121399

Is–Px//A–T

Total bonding energy = -10336.37 kcal mol^{-1}

H	0.60660732	1.67168565	-3.40000000
H	7.16655728	0.55894682	-3.40000000
H	2.77869354	2.95420339	-3.40000000
C	2.64280830	-3.33220178	-3.40000000
C	2.67294472	-4.75734064	-3.40000000

C	3.94858087	-5.26651987	-3.40000000
S	5.13596255	-4.00155411	-3.40000000
C	3.89623074	-2.73706970	-3.40000000
H	6.40228875	-1.74731222	-3.40000000
H	1.71715156	-2.77511117	-3.40000000
H	1.78191821	-5.37809287	-3.40000000
H	4.26443187	-6.30081295	-3.40000000
C	5.64861188	-0.96595702	-3.40000000
C	6.09765344	0.36475593	-3.40000000
C	5.19305791	1.42600213	-3.40000000
C	3.83379321	1.09877519	-3.40000000
C	3.34047819	-0.25135842	-3.40000000
C	4.28618930	-1.32383419	-3.40000000
N	2.73550255	1.94318503	-3.40000000
C	1.57607695	1.19205830	-3.40000000
C	1.90131381	-0.14939894	-3.40000000
C	10.56501608	-5.29107959	-3.40000000
C	9.15704699	-5.15212887	-3.40000000
C	8.88078433	-3.75779156	-3.40000000
C	10.11153699	-3.10667798	-3.40000000
N	11.11944252	-4.05188317	-3.40000000
O	9.46098493	-0.91236134	-3.40000000
H	7.91318092	-3.28355214	-3.40000000
H	12.10815784	-3.82404919	-3.40000000
H	11.17224115	-6.18465334	-3.40000000
N	10.41361875	-1.73155078	-3.40000000
O	11.63112340	-1.39286874	-3.40000000
C	8.21521562	-6.21016955	-3.40000000
C	7.40341824	-7.11257942	-3.40000000
C	6.43050900	-8.19885674	-3.40000000
H	5.52707257	2.46070982	-3.40000000
H	5.78704059	-8.15479334	-4.28846846
H	5.78704059	-8.15479334	-2.51153154
H	1.17292538	-0.94440013	-3.40000000
H	6.93272494	-9.17428851	-3.40000000
N	4.58537740	-1.43284602	0.00000000
C	4.10904668	-0.17080601	0.00000000
N	2.82642353	0.22674809	0.00000000
C	1.97976219	-0.82481149	0.00000000
C	2.33461211	-2.18515357	0.00000000
C	3.71866132	-2.48507436	0.00000000
N	0.59797841	-0.81945862	0.00000000
C	0.18598755	-2.13955201	0.00000000
N	1.19868269	-2.99352291	0.00000000
N	4.21489056	-3.73651739	0.00000000
H	4.86889014	0.60940868	0.00000000
H	0.00000000	0.00000000	0.00000000
H	-0.86304656	-2.40603390	0.00000000
H	3.58117462	-4.52464620	0.00000000
H	5.23095585	-3.88977344	0.00000000
N	7.41599982	-1.76861079	0.00000000
C	8.16514377	-0.60711280	0.00000000

N	9.53746234	-0.82769201	0.00000000
C	10.10599054	-2.08184011	0.00000000
C	9.35810649	-3.21757575	0.00000000
C	7.90650249	-3.07549030	0.00000000
O	7.11247358	-4.04132194	0.00000000
O	7.67068695	0.52685707	0.00000000
C	9.95641794	-4.59798964	0.00000000
H	6.36849540	-1.64290197	0.00000000
H	10.12459787	-0.00000000	0.00000000
H	11.19140414	-2.09630006	0.00000000
H	9.63126266	-5.16475309	0.88121399
H	11.04966811	-4.54779765	0.00000000
H	9.63126266	-5.16475309	-0.88121399

Ys–Px//A–T

Total bonding energy = -10236.15 kcal mol^{-1}

H	0.65356843	1.72006561	-3.40000000
H	7.09308299	0.81032616	-3.40000000
H	2.84159443	2.99789883	-3.40000000
C	2.76549300	-3.29701341	-3.40000000
C	2.83461100	-4.71853939	-3.40000000
C	4.12621308	-5.19016998	-3.40000000
S	5.27923397	-3.89843929	-3.40000000
C	4.00487194	-2.67305328	-3.40000000
H	6.49355000	-1.55470064	-3.40000000
H	1.82746505	-2.76004433	-3.40000000
H	1.96259900	-5.36513621	-3.40000000
H	4.46866598	-6.21535191	-3.40000000
C	5.68925818	-0.82638232	-3.40000000
C	6.04223624	0.52813785	-3.40000000
N	5.14889022	1.53933521	-3.40000000
C	3.87293813	1.13855346	-3.40000000
C	3.37382834	-0.21112870	-3.40000000
C	4.34392351	-1.25205401	-3.40000000
N	2.78283840	1.98661888	-3.40000000
C	1.62057223	1.23609966	-3.40000000
C	1.93855037	-0.10777603	-3.40000000
C	10.65669669	-5.26258849	-3.40000000
C	9.25105422	-5.10363567	-3.40000000
C	8.99384410	-3.70586317	-3.40000000
C	10.23346024	-3.07242566	-3.40000000
N	11.22820410	-4.03100360	-3.40000000
O	9.61623186	-0.86822219	-3.40000000
H	8.03194445	-3.22192936	-3.40000000
H	12.21975662	-3.81573483	-3.40000000
H	11.25118045	-6.16465952	-3.40000000
N	10.55618415	-1.70097499	-3.40000000
O	11.77855466	-1.38132411	-3.40000000
C	8.29997058	-6.15353370	-3.40000000
C	7.49397611	-7.06117034	-3.40000000
C	6.53055962	-8.15658074	-3.40000000
H	5.88757326	-8.11900120	-2.51105480

H	1.20725289	-0.90106616	-3.40000000
H	5.88757326	-8.11900120	-4.28894520
H	7.04147534	-9.12730786	-3.40000000
N	4.58537740	-1.43284602	0.00000000
C	4.10904668	-0.17080601	0.00000000
N	2.82642353	0.22674809	0.00000000
C	1.97976219	-0.82481149	0.00000000
C	2.33461211	-2.18515357	0.00000000
C	3.71866132	-2.48507436	0.00000000
N	0.59797841	-0.81945862	0.00000000
C	0.18598755	-2.13955201	0.00000000
N	1.19868269	-2.99352291	0.00000000
N	4.21489056	-3.73651739	0.00000000
H	4.86889014	0.60940868	0.00000000
H	0.00000000	0.00000000	0.00000000
H	-0.86304656	-2.40603390	0.00000000
H	3.58117462	-4.52464620	0.00000000
H	5.23095585	-3.88977344	0.00000000
N	7.41599982	-1.76861079	0.00000000
C	8.16514377	-0.60711280	0.00000000
N	9.53746234	-0.82769201	0.00000000
C	10.10599054	-2.08184011	0.00000000
C	9.35810649	-3.21757575	0.00000000
C	7.90650249	-3.07549030	0.00000000
O	7.11247358	-4.04132194	0.00000000
O	7.67068695	0.52685707	0.00000000
C	9.95641794	-4.59798964	0.00000000
H	6.36849540	-1.64290197	0.00000000
H	10.12459787	-0.00000000	0.00000000
H	11.19140414	-2.09630006	0.00000000
H	9.63126266	-5.16475309	0.88121399
H	11.04966811	-4.54779765	0.00000000
H	9.63126266	-5.16475309	-0.88121399

Yi–Px//A–T

Total bonding energy = -10324.32 kcal mol^{-1}

H	0.37484106	-1.69910490	3.40000000
H	6.45561789	0.60775212	3.40000000
H	2.78733369	-2.47104502	3.40000000
N	1.30442125	3.47909306	3.40000000
C	1.11358675	4.84550354	3.40000000
C	2.32966082	5.50026155	3.40000000
N	3.28429599	4.50426546	3.40000000
C	2.63199665	3.28447212	3.40000000
H	5.32930525	2.79917999	3.40000000
H	0.36849131	1.02094634	3.40000000
H	0.12307859	5.28219526	3.40000000
H	2.58846770	6.54764370	3.40000000
C	4.71941998	1.90117894	3.40000000
C	5.36769493	0.65754033	3.40000000
N	4.72509038	-0.52914547	3.40000000
C	3.39075126	-0.43157477	3.40000000

C	2.60490694	0.77137739	3.40000000
C	3.31429290	1.99759541	3.40000000
N	2.51225371	-1.49616411	3.40000000
C	1.21110390	-1.01327459	3.40000000
C	1.22746690	0.36749758	3.40000000
C	9.71668547	5.04612162	3.40000000
C	8.34766959	4.68933015	3.40000000
C	8.29278843	3.26924546	3.40000000
C	9.60895687	2.81853143	3.40000000
N	10.45699652	3.90833313	3.40000000
O	9.30365150	0.55034097	3.40000000
H	7.41526285	2.64558338	3.40000000
H	11.46926589	3.83674794	3.40000000
H	10.17740559	6.02324168	3.40000000
N	10.11975894	1.50463829	3.40000000
O	11.37368965	1.35870271	3.40000000
C	7.26548066	5.60457530	3.40000000
C	6.38682597	6.44354119	3.40000000
C	5.36812486	7.48894908	3.40000000
H	4.72982841	7.42357538	2.50982514
H	4.28852945	4.65449009	3.40000000
H	4.72982841	7.42357538	4.29017486
H	5.83752432	8.48025975	3.40000000
N	4.58537740	1.43284602	-0.00000000
C	4.10904668	0.17080601	-0.00000000
N	2.82642353	-0.22674809	-0.00000000
C	1.97976219	0.82481149	-0.00000000
C	2.33461211	2.18515357	-0.00000000
C	3.71866132	2.48507436	-0.00000000
N	0.59797841	0.81945862	-0.00000000
C	0.18598755	2.13955201	-0.00000000
N	1.19868269	2.99352291	-0.00000000
N	4.21489056	3.73651739	-0.00000000
H	4.86889014	-0.60940868	-0.00000000
H	0.00000000	-0.00000000	-0.00000000
H	-0.86304656	2.40603390	-0.00000000
H	3.58117462	4.52464620	-0.00000000
H	5.23095585	3.88977344	-0.00000000
N	7.41599982	1.76861079	-0.00000000
C	8.16514377	0.60711280	-0.00000000
N	9.53746234	0.82769201	-0.00000000
C	10.10599054	2.08184011	-0.00000000
C	9.35810649	3.21757575	-0.00000000
C	7.90650249	3.07549030	-0.00000000
O	7.11247358	4.04132194	-0.00000000
O	7.67068695	-0.52685707	-0.00000000
C	9.95641794	4.59798964	-0.00000000
H	6.36849540	1.64290197	-0.00000000
H	10.12459787	0.00000000	-0.00000000
H	11.19140414	2.09630006	-0.00000000
H	9.63126266	5.16475309	-0.88121399
H	11.04966811	4.54779765	-0.00000000

H	9.63126266	5.16475309	0.88121399

Do–Px//A–T

Total bonding energy = -10196.64 kcal mol^{-1}

H	0.82859881	-1.61005640	3.40000000
H	7.30934669	-1.47130924	3.40000000
H	2.77883593	-3.25712028	3.40000000
C	3.29610150	2.95591468	3.40000000
C	3.67909056	4.33503514	3.40000000
C	5.04682650	4.36201132	3.40000000
O	5.54914103	3.07933563	3.40000000
C	4.45585937	2.21115932	3.40000000
H	6.89872295	0.94032312	3.40000000
H	2.29728064	2.54400373	3.40000000
H	3.02191088	5.19486354	3.40000000
H	5.78759029	5.14879079	3.40000000
C	6.04514687	0.27277143	3.40000000
C	6.28344844	-1.10864964	3.40000000
N	5.31768887	-2.05693059	3.40000000
C	4.08143045	-1.54894847	3.40000000
C	3.70466927	-0.17810716	3.40000000
C	4.73226940	0.79343497	3.40000000
N	2.88233950	-2.24819902	3.40000000
C	1.87068182	-1.31622083	3.40000000
N	2.31383165	-0.06572707	3.40000000
C	10.69356206	5.10136714	3.40000000
C	9.28136099	5.02563633	3.40000000
C	8.93843819	3.64476125	3.40000000
C	10.14034028	2.94021308	3.40000000
N	11.19039417	3.83882156	3.40000000
O	9.40852501	0.76948543	3.40000000
H	7.94649973	3.21882276	3.40000000
H	12.16709559	3.56384962	3.40000000
H	11.34016909	5.96685438	3.40000000
N	10.38972578	1.55383513	3.40000000
O	11.59359572	1.16982811	3.40000000
C	8.39260233	6.12889148	3.40000000
C	7.62212661	7.06740370	3.40000000
C	6.68345235	8.18423816	3.40000000
H	6.03843248	8.15800632	2.51210467
H	6.03843248	8.15800632	4.28789533
H	7.21564249	9.14349338	3.40000000
N	4.58537740	1.43284602	-0.00000000
C	4.10904668	0.17080601	-0.00000000
N	2.82642353	-0.22674809	-0.00000000
C	1.97976219	0.82481149	-0.00000000
C	2.33461211	2.18515357	-0.00000000
C	3.71866132	2.48507436	-0.00000000
N	0.59797841	0.81945862	-0.00000000
C	0.18598755	2.13955201	-0.00000000
N	1.19868269	2.99352291	-0.00000000
N	4.21489056	3.73651739	-0.00000000

H	4.86889014	-0.60940868	-0.00000000
H	0.00000000	-0.00000000	-0.00000000
H	-0.86304656	2.40603390	-0.00000000
H	3.58117462	4.52464620	-0.00000000
H	5.23095585	3.88977344	-0.00000000
N	7.41599982	1.76861079	-0.00000000
C	8.16514377	0.60711280	-0.00000000
N	9.53746234	0.82769201	-0.00000000
C	10.10599054	2.08184011	-0.00000000
C	9.35810649	3.21757575	-0.00000000
C	7.90650249	3.07549030	-0.00000000
O	7.11247358	4.04132194	-0.00000000
O	7.67068695	-0.52685707	-0.00000000
C	9.95641794	4.59798964	-0.00000000
H	6.36849540	1.64290197	-0.00000000
H	10.12459787	0.00000000	-0.00000000
H	11.19140414	2.09630006	-0.00000000
H	9.63126266	5.16475309	-0.88121399
H	11.04966811	4.54779765	-0.00000000
H	9.63126266	5.16475309	0.88121399

Dp–Px//A–T
Total bonding energy = -10398.39 kcal mol^{-1}

H	0.89638086	-1.58870619	3.40000000
H	7.37140625	-1.37066422	3.40000000
H	2.87170741	-3.20496799	3.40000000
C	4.52889651	2.33461377	3.40000000
N	5.63360974	3.11940510	3.40000000
H	6.30795197	8.44233667	4.28722551
H	6.30795197	8.44233667	2.51277449
N	5.52348534	4.45511084	3.40000000
H	6.92398807	1.04687886	3.40000000
C	4.30377705	5.01760504	3.40000000
C	3.12147559	4.27077263	3.40000000
C	3.23235795	2.89065655	3.40000000
C	6.08622513	0.36129068	3.40000000
C	6.34283705	-1.01647237	3.40000000
N	5.38403565	-1.97085807	3.40000000
C	4.14601650	-1.47734451	3.40000000
C	3.75199083	-0.10521822	3.40000000
C	4.77332027	0.87162890	3.40000000
N	2.95851556	-2.19438207	3.40000000
C	1.93413531	-1.28003563	3.40000000
N	2.35623724	-0.02284860	3.40000000
C	10.84037277	5.20735451	3.40000000
C	9.42574201	5.19776897	3.40000000
C	9.01548710	3.83387513	3.40000000
C	10.18631676	3.07488430	3.40000000
N	11.27858898	3.92400183	3.40000000
O	9.37885757	0.92765111	3.40000000
H	7.99969645	3.45949611	3.40000000
H	12.24056719	3.60190708	3.40000000

H	11.52674159	6.04189047	3.40000000
N	10.38431261	1.68168991	3.40000000
O	11.57554413	1.25370801	3.40000000
C	8.59532544	6.34526755	3.40000000
C	7.86349993	7.31312081	3.40000000
C	6.95486679	8.45405292	3.40000000
H	2.15258788	4.76305898	3.40000000
H	2.36578077	2.23964951	3.40000000
H	4.29568885	6.10564291	3.40000000
H	7.50762122	9.40199514	3.40000000
N	4.58537740	1.43284602	-0.00000000
C	4.10904668	0.17080601	-0.00000000
N	2.82642353	-0.22674809	-0.00000000
C	1.97976219	0.82481149	-0.00000000
C	2.33461211	2.18515357	-0.00000000
C	3.71866132	2.48507436	-0.00000000
N	0.59797841	0.81945862	-0.00000000
C	0.18598755	2.13955201	-0.00000000
N	1.19868269	2.99352291	-0.00000000
N	4.21489056	3.73651739	-0.00000000
H	4.86889014	-0.60940868	-0.00000000
H	0.00000000	-0.00000000	-0.00000000
H	-0.86304656	2.40603390	-0.00000000
H	3.58117462	4.52464620	-0.00000000
H	5.23095585	3.88977344	-0.00000000
N	7.41599982	1.76861079	-0.00000000
C	8.16514377	0.60711280	-0.00000000
N	9.53746234	0.82769201	-0.00000000
C	10.10599054	2.08184011	-0.00000000
C	9.35810649	3.21757575	-0.00000000
C	7.90650249	3.07549030	-0.00000000
O	7.11247358	4.04132194	-0.00000000
O	7.67068695	-0.52685707	-0.00000000
C	9.95641794	4.59798964	-0.00000000
H	6.36849540	1.64290197	-0.00000000
H	10.12459787	0.00000000	-0.00000000
H	11.19140414	2.09630006	-0.00000000
H	9.63126266	5.16475309	-0.88121399
H	11.04966811	4.54779765	-0.00000000
H	9.63126266	5.16475309	0.88121399

Dss–Px//A–T

Total bonding energy = -11171.51 kcal mol^{-1}

H	1.41921568	1.87498650	-3.40000000
H	7.80183590	0.74221165	-3.40000000
H	3.60260523	3.19975129	-3.40000000
C	3.12523032	-2.94734083	-3.40000000
C	3.02221783	-4.35590978	-3.40000000
C	4.24885227	-5.00945017	-3.40000000
S	5.56099711	-3.84040236	-3.40000000
C	4.43299293	-2.47990352	-3.40000000
H	7.03170899	-1.56812783	-3.40000000

H	2.27996725	-2.26977300	-3.40000000
H	2.07544947	-4.88731309	-3.40000000
H	3.87648937	-9.93716405	-3.40000000
C	6.28326976	-0.78232829	-3.40000000
C	6.73237744	0.54397888	-3.40000000
N	5.92152678	1.62802717	-3.40000000
C	4.62331337	1.31062154	-3.40000000
C	4.04042034	0.01221907	-3.40000000
C	4.90788843	-1.10948967	-3.40000000
N	3.54711139	2.18705160	-3.40000000
C	2.40346935	1.42395881	-3.40000000
N	2.64827709	0.12044293	-3.40000000
C	11.86917177	-5.41000071	-3.40000000
C	10.49114229	-5.73156867	-3.40000000
C	9.77829221	-4.50059112	-3.40000000
C	10.73767213	-3.48888392	-3.40000000
N	11.99524176	-4.05992881	-3.40000000
O	9.43906546	-1.60279703	-3.40000000
H	8.70777146	-4.35923014	-3.40000000
H	12.85688563	-3.52387699	-3.40000000
H	12.73035119	-6.06223120	-3.40000000
N	10.59702999	-2.08740815	-3.40000000
O	11.64966298	-1.38816253	-3.40000000
C	9.96993640	-7.04873667	-3.40000000
C	9.58522649	-8.20020242	-3.40000000
C	9.15094442	-9.59245973	-3.40000000
C	4.51236947	-6.42778733	-3.40000000
C	5.72562119	-7.10285858	-3.40000000
C	5.58381678	-8.51865366	-3.40000000
C	4.27037139	-8.92887140	-3.40000000
S	3.17696434	-7.58047037	-3.40000000
H	8.55020308	-9.82241876	-4.28914528
H	8.55020308	-9.82241876	-2.51085472
H	6.68700163	-6.59849652	-3.40000000
H	6.41748370	-9.21007326	-3.40000000
H	10.01696453	-10.26628960	-3.40000000
N	4.58537740	-1.43284602	0.00000000
C	4.10904668	-0.17080601	0.00000000
N	2.82642353	0.22674809	0.00000000
C	1.97976219	-0.82481149	0.00000000
C	2.33461211	-2.18515357	0.00000000
C	3.71866132	-2.48507436	0.00000000
N	0.59797841	-0.81945862	0.00000000
C	0.18598755	-2.13955201	0.00000000
N	1.19868269	-2.99352291	0.00000000
N	4.21489056	-3.73651739	0.00000000
H	4.86889014	0.60940868	0.00000000
H	0.00000000	0.00000000	0.00000000
H	-0.86304656	-2.40603390	0.00000000
H	3.58117462	-4.52464620	0.00000000
H	5.23095585	-3.88977344	0.00000000
N	7.41599982	-1.76861079	0.00000000

C	8.16514377	-0.60711280	0.00000000
N	9.53746234	-0.82769201	0.00000000
C	10.10599054	-2.08184011	0.00000000
C	9.35810649	-3.21757575	0.00000000
C	7.90650249	-3.07549030	0.00000000
O	7.11247358	-4.04132194	0.00000000
O	7.67068695	0.52685707	0.00000000
C	9.95641794	-4.59798964	0.00000000
H	6.36849540	-1.64290197	0.00000000
H	10.12459787	-0.00000000	0.00000000
H	11.19140414	-2.09630006	0.00000000
H	9.63126266	-5.16475309	0.88121399
H	11.04966811	-4.54779765	0.00000000
H	9.63126266	-5.16475309	-0.88121399

Dt–Px//A–T

Total bonding energy = -10036.27 kcal mol^{-1}

H	0.69207161	1.68348304	-3.40000000
H	7.11527504	0.83283002	-3.40000000
H	2.81892344	3.09874698	-3.40000000
N	2.67833126	-3.01487858	-3.40000000
C	2.64637386	-4.38905373	-3.40000000
C	3.86938913	-5.01534493	-3.40000000
S	5.13947762	-3.84639072	-3.40000000
C	3.91394840	-2.55285535	-3.40000000
H	6.44625763	-1.50864741	-3.40000000
H	5.72175514	-7.90965179	-4.28881576
H	1.68984893	-4.90180489	-3.40000000
H	4.08227995	-6.07542839	-3.40000000
C	5.66315816	-0.75817751	-3.40000000
C	6.05654284	0.58498073	-3.40000000
N	5.19386735	1.62633975	-3.40000000
C	3.91317856	1.25404953	-3.40000000
C	3.38148675	-0.07299380	-3.40000000
C	4.30544365	-1.14463247	-3.40000000
N	2.80426204	2.08455919	-3.40000000
C	1.69410717	1.27307859	-3.40000000
N	1.98894858	-0.01944500	-3.40000000
C	10.54562241	-5.10502955	-3.40000000
C	9.13790945	-4.96679976	-3.40000000
C	8.86178976	-3.57266138	-3.40000000
C	10.09072004	-2.92055698	-3.40000000
N	11.09937465	-3.86490552	-3.40000000
O	9.42993822	-0.72957655	-3.40000000
H	7.89344366	-3.10272078	-3.40000000
H	12.08776873	-3.63546331	-3.40000000
H	11.15364850	-5.99805109	-3.40000000
N	10.38730652	-1.54367875	-3.40000000
O	11.60238987	-1.19957115	-3.40000000
C	8.19131015	-6.02041986	-3.40000000
C	7.36210162	-6.90694799	-3.40000000
C	6.36336304	-7.96976950	-3.40000000

H	5.72175514	-7.90965179	-2.51118424
H	6.84052224	-8.95759434	-3.40000000
N	4.58537740	-1.43284602	0.00000000
C	4.10904668	-0.17080601	0.00000000
N	2.82642353	0.22674809	0.00000000
C	1.97976219	-0.82481149	0.00000000
C	2.33461211	-2.18515357	0.00000000
C	3.71866132	-2.48507436	0.00000000
N	0.59797841	-0.81945862	0.00000000
C	0.18598755	-2.13955201	0.00000000
N	1.19868269	-2.99352291	0.00000000
N	4.21489056	-3.73651739	0.00000000
H	4.86889014	0.60940868	0.00000000
H	0.00000000	0.00000000	0.00000000
H	-0.86304656	-2.40603390	0.00000000
H	3.58117462	-4.52464620	0.00000000
H	5.23095585	-3.88977344	0.00000000
N	7.41599982	-1.76861079	0.00000000
C	8.16514377	-0.60711280	0.00000000
N	9.53746234	-0.82769201	0.00000000
C	10.10599054	-2.08184011	0.00000000
C	9.35810649	-3.21757575	0.00000000
C	7.90650249	-3.07549030	0.00000000
O	7.11247358	-4.04132194	0.00000000
O	7.67068695	0.52685707	0.00000000
C	9.95641794	-4.59798964	0.00000000
H	6.36849540	-1.64290197	0.00000000
H	10.12459787	-0.00000000	0.00000000
H	11.19140414	-2.09630006	0.00000000
H	9.63126266	-5.16475309	0.88121399
H	11.04966811	-4.54779765	0.00000000
H	9.63126266	-5.16475309	-0.88121399

Yo–Px/A–T

Total bonding energy = -10294.21 kcal mol^{-1}

H	0.72923755	-1.69358245	3.40000000
H	7.22454471	-1.31556606	3.40000000
H	2.80154839	-3.15214058	3.40000000
C	3.28074720	3.17335610	3.40000000
C	3.71376226	4.53820010	3.40000000
C	5.08033329	4.51361241	3.40000000
O	5.53159526	3.21272894	3.40000000
C	4.40947298	2.38057668	3.40000000
H	6.81938384	1.10049066	3.40000000
H	2.25744049	2.83082159	3.40000000
H	3.08733844	5.42058711	3.40000000
H	5.84920720	5.27305427	3.40000000
C	5.96484851	0.43449866	3.40000000
C	6.20041662	-0.94707976	3.40000000
N	5.22927542	-1.88489682	3.40000000
C	3.98750959	-1.38570578	3.40000000
C	3.60186491	-0.00309812	3.40000000

C	4.65303820	0.95207017	3.40000000
N	2.82911573	-2.13955549	3.40000000
C	1.73370110	-1.29299227	3.40000000
C	2.16427917	0.01990238	3.40000000
C	10.70175549	5.20174691	3.40000000
C	9.28846354	5.14899986	3.40000000
C	8.92317073	3.77369629	3.40000000
C	10.11330959	3.05000197	3.40000000
N	11.17798748	3.93131431	3.40000000
O	9.34408105	0.89279513	3.40000000
H	7.92539358	3.36158896	3.40000000
H	12.14993351	3.63985877	3.40000000
H	11.36219394	6.05674962	3.40000000
N	10.33874671	1.65949714	3.40000000
O	11.53602521	1.25505356	3.40000000
C	8.41754187	6.26638330	3.40000000
C	7.66068312	7.21564630	3.40000000
C	6.73812275	8.34559883	3.40000000
H	6.09242924	8.32886682	4.28775433
H	6.09242924	8.32886682	2.51224567
H	1.50994471	0.87880309	3.40000000
H	7.28449889	9.29695689	3.40000000
N	4.58537740	1.43284602	-0.00000000
C	4.10904668	0.17080601	-0.00000000
N	2.82642353	-0.22674809	-0.00000000
C	1.97976219	0.82481149	-0.00000000
C	2.33461211	2.18515357	-0.00000000
C	3.71866132	2.48507436	-0.00000000
N	0.59797841	0.81945862	-0.00000000
C	0.18598755	2.13955201	-0.00000000
N	1.19868269	2.99352291	-0.00000000
N	4.21489056	3.73651739	-0.00000000
H	4.86889014	-0.60940868	-0.00000000
H	0.00000000	-0.00000000	-0.00000000
H	-0.86304656	2.40603390	-0.00000000
H	3.58117462	4.52464620	-0.00000000
H	5.23095585	3.88977344	-0.00000000
N	7.41599982	1.76861079	-0.00000000
C	8.16514377	0.60711280	-0.00000000
N	9.53746234	0.82769201	-0.00000000
C	10.10599054	2.08184011	-0.00000000
C	9.35810649	3.21757575	-0.00000000
C	7.90650249	3.07549030	-0.00000000
O	7.11247358	4.04132194	-0.00000000
O	7.67068695	-0.52685707	-0.00000000
C	9.95641794	4.59798964	-0.00000000
H	6.36849540	1.64290197	-0.00000000
H	10.12459787	0.00000000	-0.00000000
H	11.19140414	2.09630006	-0.00000000
H	9.63126266	5.16475309	-0.88121399
H	11.04966811	4.54779765	-0.00000000
H	9.63126266	5.16475309	0.88121399

Ps–Px//A–T

Total bonding energy = -10039.01 kcal mol^{-1}

H	0.58669695	1.57432000	-3.40000000
H	6.80903106	-0.18585388	-3.40000000
H	2.87118114	2.71943015	-3.40000000
C	2.01946641	-3.44604120	-3.40000000
C	1.91838728	-4.86280185	-3.40000000
C	3.15108254	-5.48269659	-3.40000000
S	4.46008925	-4.34812407	-3.40000000
C	3.33374701	-2.99943819	-3.40000000
H	5.31497243	-7.96111952	-4.28870847
H	1.17642352	-2.76494849	-3.40000000
H	0.97762170	-5.40464621	-3.40000000
H	3.35862555	-6.54475995	-3.40000000
N	5.20308185	-1.48374287	-3.40000000
C	5.72393446	-0.24447406	-3.40000000
N	5.05593516	0.92969282	-3.40000000
C	3.72957556	0.75520507	-3.40000000
C	3.04577663	-0.48790403	-3.40000000
C	3.84967017	-1.64713567	-3.40000000
N	2.73585184	1.71393023	-3.40000000
C	1.52947016	1.04217414	-3.40000000
N	1.66580457	-0.27491482	-3.40000000
C	10.02334591	-5.05426175	-3.40000000
C	8.61879860	-4.88460077	-3.40000000
C	8.36576001	-3.48276615	-3.40000000
C	9.61647306	-2.86224433	-3.40000000
N	10.60360657	-3.82978073	-3.40000000
O	9.06806649	-0.63489226	-3.40000000
H	7.40369819	-2.98619351	-3.40000000
H	11.59623301	-3.61969109	-3.40000000
H	10.60932588	-5.96194173	-3.40000000
N	9.97772306	-1.50115812	-3.40000000
O	11.20985602	-1.21738312	-3.40000000
C	7.68169365	-5.94697689	-3.40000000
C	6.90388959	-6.87876852	-3.40000000
C	5.95889192	-7.98946924	-3.40000000
H	5.31497243	-7.96111952	-2.51129153
H	6.48468148	-8.95230380	-3.40000000
N	4.58537740	-1.43284602	0.00000000
C	4.10904668	-0.17080601	0.00000000
N	2.82642353	0.22674809	0.00000000
C	1.97976219	-0.82481149	0.00000000
C	2.33461211	-2.18515357	0.00000000
C	3.71866132	-2.48507436	0.00000000
N	0.59797841	-0.81945862	0.00000000
C	0.18598755	-2.13955201	0.00000000
N	1.19868269	-2.99352291	0.00000000
N	4.21489056	-3.73651739	0.00000000
H	4.86889014	0.60940868	0.00000000
H	0.00000000	0.00000000	0.00000000
H	-0.86304656	-2.40603390	0.00000000

H	3.58117462	-4.52464620	0.00000000
H	5.23095585	-3.88977344	0.00000000
N	7.41599982	-1.76861079	0.00000000
C	8.16514377	-0.60711280	0.00000000
N	9.53746234	-0.82769201	0.00000000
C	10.10599054	-2.08184011	0.00000000
C	9.35810649	-3.21757575	0.00000000
C	7.90650249	-3.07549030	0.00000000
O	7.11247358	-4.04132194	0.00000000
O	7.67068695	0.52685707	0.00000000
C	9.95641794	-4.59798964	0.00000000
H	6.36849540	-1.64290197	0.00000000
H	10.12459787	-0.00000000	0.00000000
H	11.19140414	-2.09630006	0.00000000
H	9.63126266	-5.16475309	0.88121399
H	11.04966811	-4.54779765	0.00000000
H	9.63126266	-5.16475309	-0.88121399

Es–Px//A–T

Total bonding energy = -10137.45 kcal mol^{-1}

H	0.52830361	1.66868676	-3.40000000
H	6.71893861	-0.29375890	-3.40000000
H	2.87785226	2.61427102	-3.40000000
C	2.00602837	-3.66154585	-3.40000000
C	1.97284108	-5.08287005	-3.40000000
C	3.23291167	-5.64155333	-3.40000000
S	4.48304159	-4.44167293	-3.40000000
C	3.29391240	-3.14567895	-3.40000000
H	0.69618830	-1.01648950	-3.40000000
H	1.11336744	-3.04944947	-3.40000000
H	1.05748461	-5.66670290	-3.40000000
H	3.49217841	-6.69220581	-3.40000000
N	5.12216877	-1.60643443	-3.40000000
C	5.63432743	-0.36329244	-3.40000000
N	4.95187685	0.79833742	-3.40000000
C	3.62369065	0.62631998	-3.40000000
C	2.93523205	-0.63117396	-3.40000000
C	3.77080749	-1.77294740	-3.40000000
N	2.67644561	1.62110701	-3.40000000
C	1.41139964	1.04494728	-3.40000000
C	1.52850786	-0.32891833	-3.40000000
C	9.99360780	-5.13705861	-3.40000000
C	8.58716048	-4.98280846	-3.40000000
C	8.31905702	-3.58384418	-3.40000000
C	9.56318652	-2.94989918	-3.40000000
N	10.56067270	-3.90645705	-3.40000000
O	8.99068670	-0.72876856	-3.40000000
H	7.35370463	-3.09367542	-3.40000000
H	11.55130915	-3.68736428	-3.40000000
H	10.58950469	-6.03827747	-3.40000000
N	9.90970932	-1.58483961	-3.40000000
O	11.13887553	-1.28807471	-3.40000000

C	7.66593589	-6.05940461	-3.40000000
C	6.91353667	-7.01197582	-3.40000000
C	6.00461490	-8.15244402	-3.40000000
H	5.35999251	-8.14549529	-2.51133619
H	5.35999251	-8.14549529	-4.28866381
H	6.56222002	-9.09719500	-3.40000000
N	4.58537740	-1.43284602	0.00000000
C	4.10904668	-0.17080601	0.00000000
N	2.82642353	0.22674809	0.00000000
C	1.97976219	-0.82481149	0.00000000
C	2.33461211	-2.18515357	0.00000000
C	3.71866132	-2.48507436	0.00000000
N	0.59797841	-0.81945862	0.00000000
C	0.18598755	-2.13955201	0.00000000
N	1.19868269	-2.99352291	0.00000000
N	4.21489056	-3.73651739	0.00000000
H	4.86889014	0.60940868	0.00000000
H	0.00000000	0.00000000	0.00000000
H	-0.86304656	-2.40603390	0.00000000
H	3.58117462	-4.52464620	0.00000000
H	5.23095585	-3.88977344	0.00000000
N	7.41599982	-1.76861079	0.00000000
C	8.16514377	-0.60711280	0.00000000
N	9.53746234	-0.82769201	0.00000000
C	10.10599054	-2.08184011	0.00000000
C	9.35810649	-3.21757575	0.00000000
C	7.90650249	-3.07549030	0.00000000
O	7.11247358	-4.04132194	0.00000000
O	7.67068695	0.52685707	0.00000000
C	9.95641794	-4.59798964	0.00000000
H	6.36849540	-1.64290197	0.00000000
H	10.12459787	-0.00000000	0.00000000
H	11.19140414	-2.09630006	0.00000000
H	9.63126266	-5.16475309	0.88121399
H	11.04966811	-4.54779765	0.00000000
H	9.63126266	-5.16475309	-0.88121399

Ysm–Px//A–T

Total bonding energy = -10605.68 kcal mol^{-1}

H	0.93937617	-1.81151198	3.40000000
H	7.35967169	-0.76518238	3.40000000
H	3.15378955	-3.04363789	3.40000000
C	2.95346139	3.25519769	3.40000000
C	3.00081372	4.67824226	3.40000000
C	4.27822235	5.19323758	3.40000000
S	5.45102360	3.89889142	3.40000000
C	4.19890640	2.64803230	3.40000000
H	6.71447267	1.58361597	3.40000000
H	2.02298040	2.70436866	3.40000000
H	2.11628576	5.30924849	3.40000000
C	4.71520463	6.62780560	3.40000000
C	5.92291801	0.84087881	3.40000000

C	6.30270568	-0.50653087	3.40000000
N	5.43135567	-1.53746329	3.40000000
C	4.14672233	-1.16312138	3.40000000
C	3.62004355	0.17502686	3.40000000
C	4.56780304	1.23685675	3.40000000
N	3.07402374	-2.03383887	3.40000000
C	1.89614039	-1.30754385	3.40000000
C	2.18685958	0.04264137	3.40000000
C	11.16178663	5.40684505	3.40000000
C	9.75017957	5.49762942	3.40000000
C	9.25040105	4.16638291	3.40000000
C	10.35973062	3.32496890	3.40000000
N	11.50770332	4.09400123	3.40000000
O	9.36718129	1.26129492	3.40000000
H	8.21696996	3.85911984	3.40000000
H	12.44580988	3.70741005	3.40000000
H	11.90615069	6.18985731	3.40000000
N	10.43772467	1.91845950	3.40000000
O	11.58554346	1.39009114	3.40000000
C	8.99387450	6.69556560	3.40000000
C	8.34436784	7.72133266	3.40000000
C	7.56765405	8.95606565	3.40000000
H	6.92453127	9.01919928	2.51296580
H	5.32122049	6.86420423	4.28307911
H	3.83738113	7.28174305	3.40000000
H	5.32122049	6.86420423	2.51692089
H	1.44015852	0.82165811	3.40000000
H	6.92453127	9.01919928	4.28703420
H	8.22711834	9.83284872	3.40000000
N	4.58537740	1.43284602	-0.00000000
C	4.10904668	0.17080601	-0.00000000
N	2.82642353	-0.22674809	-0.00000000
C	1.97976219	0.82481149	-0.00000000
C	2.33461211	2.18515357	-0.00000000
C	3.71866132	2.48507436	-0.00000000
N	0.59797841	0.81945862	-0.00000000
C	0.18598755	2.13955201	-0.00000000
N	1.19868269	2.99352291	-0.00000000
N	4.21489056	3.73651739	-0.00000000
H	4.86889014	-0.60940868	-0.00000000
H	0.00000000	-0.00000000	-0.00000000
H	-0.86304656	2.40603390	-0.00000000
H	3.58117462	4.52464620	-0.00000000
H	5.23095585	3.88977344	-0.00000000
N	7.41599982	1.76861079	-0.00000000
C	8.16514377	0.60711280	-0.00000000
N	9.53746234	0.82769201	-0.00000000
C	10.10599054	2.08184011	-0.00000000
C	9.35810649	3.21757575	-0.00000000
C	7.90650249	3.07549030	-0.00000000
O	7.11247358	4.04132194	-0.00000000
O	7.67068695	-0.52685707	-0.00000000

C	9.95641794	4.59798964	-0.00000000
H	6.36849540	1.64290197	-0.00000000
H	10.12459787	0.00000000	-0.00000000
H	11.19140414	2.09630006	-0.00000000
H	9.63126266	5.16475309	-0.88121399
H	11.04966811	4.54779765	-0.00000000
H	9.63126266	5.16475309	0.88121399

Yss–Px//A–T

Total bonding energy = -11268.15 kcal mol^{-1}

H	1.32319487	2.01494820	-3.40000000
H	7.67007002	0.58624311	-3.40000000
H	3.60797587	3.11198295	-3.40000000
C	3.03396385	-3.15796575	-3.40000000
C	2.98539517	-4.57078072	-3.40000000
C	4.23541254	-5.17425929	-3.40000000
S	5.49703199	-3.95168677	-3.40000000
C	4.31828877	-2.63045530	-3.40000000
H	6.88478198	-1.72004568	-3.40000000
H	2.14207983	-2.54730965	-3.40000000
H	2.05865543	-5.13633918	-3.40000000
C	4.55853553	-6.58049009	-3.40000000
C	6.14092312	-0.93015962	-3.40000000
C	6.59964698	0.39191552	-3.40000000
N	5.79017928	1.47252968	-3.40000000
C	4.48572843	1.17501244	-3.40000000
C	3.87948164	-0.12920114	-3.40000000
C	4.76495719	-1.24508100	-3.40000000
N	3.46725847	2.10883329	-3.40000000
C	2.24832197	1.45510704	-3.40000000
C	2.45726995	0.08951329	-3.40000000
C	11.86546532	-5.47377960	-3.40000000
C	10.50166035	-5.85064451	-3.40000000
C	9.73986592	-4.64911512	-3.40000000
C	10.65767397	-3.59966276	-3.40000000
N	11.93711172	-4.11966404	-3.40000000
O	9.28391176	-1.76764675	-3.40000000
H	8.66472020	-4.54927991	-3.40000000
H	12.77636518	-3.54924179	-3.40000000
H	12.75228660	-6.09069897	-3.40000000
N	10.46025067	-2.20482748	-3.40000000
O	11.48408188	-1.46396795	-3.40000000
C	10.03440007	-7.18802447	-3.40000000
C	9.69697919	-8.35425853	-3.40000000
C	9.31794521	-9.76280445	-3.40000000
C	5.79955996	-7.20200323	-3.40000000
C	5.72053158	-8.62287537	-3.40000000
C	4.42680386	-9.09061979	-3.40000000
S	3.27521447	-7.79055243	-3.40000000
H	6.73830913	-6.65699914	-3.40000000
H	8.72615290	-10.01602709	-4.28897279
H	8.72615290	-10.01602709	-2.51102721

H	6.58539013	-9.27496943	-3.40000000
H	4.07672411	-10.11492851	-3.40000000
H	1.66438256	-0.64240648	-3.40000000
H	10.20942269	-10.40246102	-3.40000000
N	4.58537740	-1.43284602	0.00000000
C	4.10904668	-0.17080601	0.00000000
N	2.82642353	0.22674809	0.00000000
C	1.97976219	-0.82481149	0.00000000
C	2.33461211	-2.18515357	0.00000000
C	3.71866132	-2.48507436	0.00000000
N	0.59797841	-0.81945862	0.00000000
C	0.18598755	-2.13955201	0.00000000
N	1.19868269	-2.99352291	0.00000000
N	4.21489056	-3.73651739	0.00000000
H	4.86889014	0.60940868	0.00000000
H	0.00000000	0.00000000	0.00000000
H	-0.86304656	-2.40603390	0.00000000
H	3.58117462	-4.52464620	0.00000000
H	5.23095585	-3.88977344	0.00000000
N	7.41599982	-1.76861079	0.00000000
C	8.16514377	-0.60711280	0.00000000
N	9.53746234	-0.82769201	0.00000000
C	10.10599054	-2.08184011	0.00000000
C	9.35810649	-3.21757575	0.00000000
C	7.90650249	-3.07549030	0.00000000
O	7.11247358	-4.04132194	0.00000000
O	7.67068695	0.52685707	0.00000000
C	9.95641794	-4.59798964	0.00000000
H	6.36849540	-1.64290197	0.00000000
H	10.12459787	-0.00000000	0.00000000
H	11.19140414	-2.09630006	0.00000000
H	9.63126266	-5.16475309	0.88121399
H	11.04966811	-4.54779765	0.00000000
H	9.63126266	-5.16475309	-0.88121399

Cartesian Coordinates and Total Bonding Energies of Ds–Px//G–C variants in Chloroform

Cartesian coordinates (Å) and total bonding energies (kcal mol^{-1}) of Ds–Px//G–C variants optimized at ZORA-BLYP-D3(BJ)-TZ2P level of theory in chloroform.

Ds–Px//G–C
Total bonding energy = -10048.52 kcal mol^{-1}

H	0.72856716	-1.60276921	3.40000000
H	7.16092660	-0.80855098	3.40000000
H	2.83953137	-3.04060014	3.40000000
C	2.67803974	3.12545749	3.40000000
C	2.65564222	4.54744604	3.40000000
C	3.91548548	5.10309876	3.40000000
S	5.15253950	3.89135824	3.40000000
C	3.95952331	2.59217636	3.40000000
H	6.51238063	1.53636116	3.40000000
H	1.79739808	2.49463573	3.40000000
H	1.74525108	5.13894660	3.40000000
H	4.18934567	6.14940827	3.40000000
C	5.72182439	0.79331947	3.40000000
C	6.10340654	-0.55419935	3.40000000
N	5.23657138	-1.59305384	3.40000000
C	3.95741900	-1.20808143	3.40000000
C	3.44354125	0.12008203	3.40000000
C	4.36655224	1.19434228	3.40000000
N	2.83691919	-2.02635310	3.40000000
C	1.73515383	-1.20391568	3.40000000
N	2.04732848	0.08481069	3.40000000
C	10.57036263	5.16733316	3.40000000
C	9.16222855	5.03436994	3.40000000
C	8.88044065	3.64125012	3.40000000
C	10.10732591	2.98472039	3.40000000
N	11.11952870	3.92510994	3.40000000
O	9.44043139	0.79545846	3.40000000
H	7.91010651	3.17460086	3.40000000
H	12.10719331	3.69275040	3.40000000
H	11.18201626	6.05786704	3.40000000
N	10.39989136	1.60692215	3.40000000
O	11.61416129	1.25928949	3.40000000
C	8.22023578	6.09225181	3.40000000
C	7.39925373	6.98646408	3.40000000
C	6.41301359	8.06100258	3.40000000
H	5.77063127	8.00843930	4.28873742
H	6.90239143	9.04280928	3.40000000
H	5.77063127	8.00843930	2.51126258
N	5.00124589	-1.00838958	0.00000000
O	4.31439792	3.57512911	0.00000000
N	4.56316571	1.27825998	0.00000000
C	4.08363083	-0.01712178	0.00000000
N	2.77812701	-0.31202208	0.00000000
C	1.98907622	0.78520120	0.00000000

C	2.37521426	2.13786499	0.00000000
C	3.76977416	2.44419170	0.00000000
N	0.61018743	0.81038579	0.00000000
C	0.22256913	2.14418538	0.00000000
N	1.25359689	2.96921021	0.00000000
H	6.01546686	-0.82638110	0.00000000
H	4.66003477	-1.96041300	0.00000000
H	5.59754734	1.42652009	0.00000000
H	0.00000000	0.00000000	0.00000000
H	-0.82080301	2.43110636	0.00000000
O	7.82914031	-0.56146045	0.00000000
N	7.12312219	3.99336689	0.00000000
N	7.44732185	1.71143725	0.00000000
C	9.39682962	3.17534130	0.00000000
C	8.25529553	0.61773344	0.00000000
N	9.64656408	0.82885228	0.00000000
C	10.19907071	2.07631739	0.00000000
C	7.97505830	2.95606283	0.00000000
H	9.81255981	4.17627118	0.00000000
H	10.23317680	0.00000000	0.00000000
H	11.28250062	2.12612238	0.00000000
H	6.09985058	3.83894401	0.00000000
H	7.48159458	4.93915143	0.00000000

Bs–Px//G–C

Total bonding energy = -10149.32 kcal mol^{-1}

H	0.69853704	1.56922549	-3.40000000
H	7.27116835	0.76309341	-3.40000000
H	2.74960871	3.07319555	-3.40000000
C	2.70334271	-3.07269189	-3.40000000
C	2.67652945	-4.49722606	-3.40000000
C	3.93173304	-5.05875707	-3.40000000
S	5.17291745	-3.84609500	-3.40000000
C	3.98378707	-2.53927168	-3.40000000
H	6.52444535	-1.54793107	-3.40000000
H	1.82385110	-2.44093952	-3.40000000
H	1.76282643	-5.08419768	-3.40000000
H	4.20361120	-6.10535557	-3.40000000
C	5.76583627	-0.77134817	-3.40000000
C	6.20314626	0.56414261	-3.40000000
C	5.29811384	1.62821363	-3.40000000
C	3.94405775	1.28326990	-3.40000000
C	3.47628641	-0.06241699	-3.40000000
C	4.40478142	-1.13949290	-3.40000000
N	2.79193136	2.06071076	-3.40000000
C	1.71796430	1.20395591	-3.40000000
N	2.07593697	-0.07068720	-3.40000000
C	10.57803361	-5.19782186	-3.40000000
C	9.17224388	-5.04091235	-3.40000000
C	8.91390572	-3.64306914	-3.40000000
C	10.15208544	-3.00759587	-3.40000000
N	11.14822467	-3.96542633	-3.40000000

O	9.52763584	-0.80551581	-3.40000000
H	7.95050124	-3.16194034	-3.40000000
H	12.13947272	-3.74893998	-3.40000000
H	11.17409012	-6.09890183	-3.40000000
N	10.47068373	-1.63595274	-3.40000000
O	11.69198550	-1.31165206	-3.40000000
C	8.21454143	-6.08453268	-3.40000000
C	7.38278641	-6.96884094	-3.40000000
C	6.38349792	-8.03107100	-3.40000000
H	5.74154755	-7.97043896	-2.51146294
H	5.63076024	2.66256438	-3.40000000
H	5.74154755	-7.97043896	-4.28853706
H	6.86071913	-9.01889336	-3.40000000
N	5.00124589	1.00838958	-0.00000000
O	4.31439792	-3.57512911	-0.00000000
N	4.56316571	-1.27825998	-0.00000000
C	4.08363083	0.01712178	-0.00000000
N	2.77812701	0.31202208	-0.00000000
C	1.98907622	-0.78520120	-0.00000000
C	2.37521426	-2.13786499	-0.00000000
C	3.76977416	-2.44419170	-0.00000000
N	0.61018743	-0.81038579	-0.00000000
C	0.22256913	-2.14418538	-0.00000000
N	1.25359689	-2.96921021	-0.00000000
H	6.01546686	0.82638110	-0.00000000
H	4.66003477	1.96041300	-0.00000000
H	5.59754734	-1.42652009	-0.00000000
H	0.00000000	-0.00000000	-0.00000000
H	-0.82080301	-2.43110636	-0.00000000
O	7.82914031	0.56146045	-0.00000000
N	7.12312219	-3.99336689	-0.00000000
N	7.44732185	-1.71143725	-0.00000000
C	9.39682962	-3.17534130	-0.00000000
C	8.25529553	-0.61773344	-0.00000000
N	9.64656408	-0.82885228	-0.00000000
C	10.19907071	-2.07631739	-0.00000000
C	7.97505830	-2.95606283	-0.00000000
H	9.81255981	-4.17627118	-0.00000000
H	10.23317680	-0.00000000	-0.00000000
H	11.28250062	-2.12612238	-0.00000000
H	6.09985058	-3.83894401	-0.00000000
H	7.48159458	-4.93915143	-0.00000000

Is–Px//G–C

Total bonding energy = -10245.08 kcal mol^{-1}

H	0.60660732	1.67168565	-3.40000000
H	7.16655728	0.55894682	-3.40000000
H	2.77869354	2.95420339	-3.40000000
C	2.64280830	-3.33220178	-3.40000000
C	2.67294472	-4.75734064	-3.40000000
C	3.94858087	-5.26651987	-3.40000000
S	5.13596255	-4.00155411	-3.40000000

C	3.89623074	-2.73706970	-3.40000000
H	6.40228875	-1.74731222	-3.40000000
H	1.71715156	-2.77511117	-3.40000000
H	1.78191821	-5.37809287	-3.40000000
H	4.26443187	-6.30081295	-3.40000000
C	5.64861188	-0.96595702	-3.40000000
C	6.09765344	0.36475593	-3.40000000
C	5.19305791	1.42600213	-3.40000000
C	3.83379321	1.09877519	-3.40000000
C	3.34047819	-0.25135842	-3.40000000
C	4.28618930	-1.32383419	-3.40000000
N	2.73550255	1.94318503	-3.40000000
C	1.57607695	1.19205830	-3.40000000
C	1.90131381	-0.14939894	-3.40000000
C	10.56501608	-5.29107959	-3.40000000
C	9.15704699	-5.15212887	-3.40000000
C	8.88078433	-3.75779156	-3.40000000
C	10.11153699	-3.10667798	-3.40000000
N	11.11944252	-4.05188317	-3.40000000
O	9.46098493	-0.91236134	-3.40000000
H	7.91318092	-3.28355214	-3.40000000
H	12.10815784	-3.82404919	-3.40000000
H	11.17224115	-6.18465334	-3.40000000
N	10.41361875	-1.73155078	-3.40000000
O	11.63112340	-1.39286874	-3.40000000
C	8.21521562	-6.21016955	-3.40000000
C	7.40341824	-7.11257942	-3.40000000
C	6.43050900	-8.19885674	-3.40000000
H	5.52707257	2.46070982	-3.40000000
H	5.78704059	-8.15479334	-4.28846846
H	5.78704059	-8.15479334	-2.51153154
H	1.17292538	-0.94440013	-3.40000000
H	6.93272494	-9.17428851	-3.40000000
N	5.00124589	1.00838958	-0.00000000
O	4.31439792	-3.57512911	-0.00000000
N	4.56316571	-1.27825998	-0.00000000
C	4.08363083	0.01712178	-0.00000000
N	2.77812701	0.31202208	-0.00000000
C	1.98907622	-0.78520120	-0.00000000
C	2.37521426	-2.13786499	-0.00000000
C	3.76977416	-2.44419170	-0.00000000
N	0.61018743	-0.81038579	-0.00000000
C	0.22256913	-2.14418538	-0.00000000
N	1.25359689	-2.96921021	-0.00000000
H	6.01546686	0.82638110	-0.00000000
H	4.66003477	1.96041300	-0.00000000
H	5.59754734	-1.42652009	-0.00000000
H	0.00000000	-0.00000000	-0.00000000
H	-0.82080301	-2.43110636	-0.00000000
O	7.82914031	0.56146045	-0.00000000
N	7.12312219	-3.99336689	-0.00000000
N	7.44732185	-1.71143725	-0.00000000

C	9.39682962	-3.17534130	-0.00000000
C	8.25529553	-0.61773344	-0.00000000
N	9.64656408	-0.82885228	-0.00000000
C	10.19907071	-2.07631739	-0.00000000
C	7.97505830	-2.95606283	-0.00000000
H	9.81255981	-4.17627118	-0.00000000
H	10.23317680	-0.00000000	-0.00000000
H	11.28250062	-2.12612238	-0.00000000
H	6.09985058	-3.83894401	-0.00000000
H	7.48159458	-4.93915143	-0.00000000

Ys–Px//G–C

Total bonding energy = -10145.33 kcal mol^{-1}

H	0.65356843	1.72006561	-3.40000000
H	7.09308299	0.81032616	-3.40000000
H	2.84159443	2.99789883	-3.40000000
C	2.76549300	-3.29701341	-3.40000000
C	2.83461100	-4.71853939	-3.40000000
C	4.12621308	-5.19016998	-3.40000000
S	5.27923397	-3.89843929	-3.40000000
C	4.00487194	-2.67305328	-3.40000000
H	6.49355000	-1.55470064	-3.40000000
H	1.82746505	-2.76004433	-3.40000000
H	1.96259900	-5.36513621	-3.40000000
H	4.46866598	-6.21535191	-3.40000000
C	5.68925818	-0.82638232	-3.40000000
C	6.04223624	0.52813785	-3.40000000
N	5.14889022	1.53933521	-3.40000000
C	3.87293813	1.13855346	-3.40000000
C	3.37382834	-0.21112870	-3.40000000
C	4.34392351	-1.25205401	-3.40000000
N	2.78283840	1.98661888	-3.40000000
C	1.62057223	1.23609966	-3.40000000
C	1.93855037	-0.10777603	-3.40000000
C	10.65669669	-5.26258849	-3.40000000
C	9.25105422	-5.10363567	-3.40000000
C	8.99384410	-3.70586317	-3.40000000
C	10.23346024	-3.07242566	-3.40000000
N	11.22820410	-4.03100360	-3.40000000
O	9.61623186	-0.86822219	-3.40000000
H	8.03194445	-3.22192936	-3.40000000
H	12.21975662	-3.81573483	-3.40000000
H	11.25118045	-6.16465952	-3.40000000
N	10.55618415	-1.70097499	-3.40000000
O	11.77855466	-1.38132411	-3.40000000
C	8.29997058	-6.15353370	-3.40000000
C	7.49397611	-7.06117034	-3.40000000
C	6.53055962	-8.15658074	-3.40000000
H	5.88757326	-8.11900120	-2.51105480
H	1.20725289	-0.90106616	-3.40000000
H	5.88757326	-8.11900120	-4.28894520
H	7.04147534	-9.12730786	-3.40000000

N	5.00124589	1.00838958	-0.00000000
O	4.31439792	-3.57512911	-0.00000000
N	4.56316571	-1.27825998	-0.00000000
C	4.08363083	0.01712178	-0.00000000
N	2.77812701	0.31202208	-0.00000000
C	1.98907622	-0.78520120	-0.00000000
C	2.37521426	-2.13786499	-0.00000000
C	3.76977416	-2.44419170	-0.00000000
N	0.61018743	-0.81038579	-0.00000000
C	0.22256913	-2.14418538	-0.00000000
N	1.25359689	-2.96921021	-0.00000000
H	6.01546686	0.82638110	-0.00000000
H	4.66003477	1.96041300	-0.00000000
H	5.59754734	-1.42652009	-0.00000000
H	0.00000000	-0.00000000	-0.00000000
H	-0.82080301	-2.43110636	-0.00000000
O	7.82914031	0.56146045	-0.00000000
N	7.12312219	-3.99336689	-0.00000000
N	7.44732185	-1.71143725	-0.00000000
C	9.39682962	-3.17534130	-0.00000000
C	8.25529553	-0.61773344	-0.00000000
N	9.64656408	-0.82885228	-0.00000000
C	10.19907071	-2.07631739	-0.00000000
C	7.97505830	-2.95606283	-0.00000000
H	9.81255981	-4.17627118	-0.00000000
H	10.23317680	-0.00000000	-0.00000000
H	11.28250062	-2.12612238	-0.00000000
H	6.09985058	-3.83894401	-0.00000000
H	7.48159458	-4.93915143	-0.00000000

Yi–Px//G–C

Total bonding energy = -10233.58 kcal mol^{-1}

H	0.37484106	-1.69910490	3.40000000
H	6.45561789	0.60775212	3.40000000
H	2.78733369	-2.47104502	3.40000000
N	1.30442125	3.47909306	3.40000000
C	1.11358675	4.84550354	3.40000000
C	2.32966082	5.50026155	3.40000000
N	3.28429599	4.50426546	3.40000000
C	2.63199665	3.28447212	3.40000000
H	5.32930525	2.79917999	3.40000000
H	0.36849131	1.02094634	3.40000000
H	0.12307859	5.28219526	3.40000000
H	2.58846770	6.54764370	3.40000000
C	4.71941998	1.90117894	3.40000000
C	5.36769493	0.65754033	3.40000000
N	4.72509038	-0.52914547	3.40000000
C	3.39075126	-0.43157477	3.40000000
C	2.60490694	0.77137739	3.40000000
C	3.31429290	1.99759541	3.40000000
N	2.51225371	-1.49616411	3.40000000
C	1.21110390	-1.01327459	3.40000000

C	1.22746690	0.36749758	3.40000000
C	9.71668547	5.04612162	3.40000000
C	8.34766959	4.68933015	3.40000000
C	8.29278843	3.26924546	3.40000000
C	9.60895687	2.81853143	3.40000000
N	10.45699652	3.90833313	3.40000000
O	9.30365150	0.55034097	3.40000000
H	7.41526285	2.64558338	3.40000000
H	11.46926589	3.83674794	3.40000000
H	10.17740559	6.02324168	3.40000000
N	10.11975894	1.50463829	3.40000000
O	11.37368965	1.35870271	3.40000000
C	7.26548066	5.60457530	3.40000000
C	6.38682597	6.44354119	3.40000000
C	5.36812486	7.48894908	3.40000000
H	4.72982841	7.42357538	2.50982514
H	4.28852945	4.65449009	3.40000000
H	4.72982841	7.42357538	4.29017486
H	5.83752432	8.48025975	3.40000000
N	5.00124589	-1.00838958	0.00000000
O	4.31439792	3.57512911	0.00000000
N	4.56316571	1.27825998	0.00000000
C	4.08363083	-0.01712178	0.00000000
N	2.77812701	-0.31202208	0.00000000
C	1.98907622	0.78520120	0.00000000
C	2.37521426	2.13786499	0.00000000
C	3.76977416	2.44419170	0.00000000
N	0.61018743	0.81038579	0.00000000
C	0.22256913	2.14418538	0.00000000
N	1.25359689	2.96921021	0.00000000
H	6.01546686	-0.82638110	0.00000000
H	4.66003477	-1.96041300	0.00000000
H	5.59754734	1.42652009	0.00000000
H	0.00000000	0.00000000	0.00000000
H	-0.82080301	2.43110636	0.00000000
O	7.82914031	-0.56146045	0.00000000
N	7.12312219	3.99336689	0.00000000
N	7.44732185	1.71143725	0.00000000
C	9.39682962	3.17534130	0.00000000
C	8.25529553	0.61773344	0.00000000
N	9.64656408	0.82885228	0.00000000
C	10.19907071	2.07631739	0.00000000
C	7.97505830	2.95606283	0.00000000
H	9.81255981	4.17627118	0.00000000
H	10.23317680	0.00000000	0.00000000
H	11.28250062	2.12612238	0.00000000
H	6.09985058	3.83894401	0.00000000
H	7.48159458	4.93915143	0.00000000

Do–Px//G–C

Total bonding energy = -10106.53 kcal mol^{-1}

H	0.82859881	-1.61005640	3.40000000

H	7.30934669	-1.47130924	3.40000000
H	2.77883593	-3.25712028	3.40000000
C	3.29610150	2.95591468	3.40000000
C	3.67909056	4.33503514	3.40000000
C	5.04682650	4.36201132	3.40000000
O	5.54914103	3.07933563	3.40000000
C	4.45585937	2.21115932	3.40000000
H	6.89872295	0.94032312	3.40000000
H	2.29728064	2.54400373	3.40000000
H	3.02191088	5.19486354	3.40000000
H	5.78759029	5.14879079	3.40000000
C	6.04514687	0.27277143	3.40000000
C	6.28344844	-1.10864964	3.40000000
N	5.31768887	-2.05693059	3.40000000
C	4.08143045	-1.54894847	3.40000000
C	3.70466927	-0.17810716	3.40000000
C	4.73226940	0.79343497	3.40000000
N	2.88233950	-2.24819902	3.40000000
C	1.87068182	-1.31622083	3.40000000
N	2.31383165	-0.06572707	3.40000000
C	10.69356206	5.10136714	3.40000000
C	9.28136099	5.02563633	3.40000000
C	8.93843819	3.64476125	3.40000000
C	10.14034028	2.94021308	3.40000000
N	11.19039417	3.83882156	3.40000000
O	9.40852501	0.76948543	3.40000000
H	7.94649973	3.21882276	3.40000000
H	12.16709559	3.56384962	3.40000000
H	11.34016909	5.96685438	3.40000000
N	10.38972578	1.55383513	3.40000000
O	11.59359572	1.16982811	3.40000000
C	8.39260233	6.12889148	3.40000000
C	7.62212661	7.06740370	3.40000000
C	6.68345235	8.18423816	3.40000000
H	6.03843248	8.15800632	2.51210467
H	6.03843248	8.15800632	4.28789533
H	7.21564249	9.14349338	3.40000000
N	5.00124589	-1.00838958	0.00000000
O	4.31439792	3.57512911	0.00000000
N	4.56316571	1.27825998	0.00000000
C	4.08363083	-0.01712178	0.00000000
N	2.77812701	-0.31202208	0.00000000
C	1.98907622	0.78520120	0.00000000
C	2.37521426	2.13786499	0.00000000
C	3.76977416	2.44419170	0.00000000
N	0.61018743	0.81038579	0.00000000
C	0.22256913	2.14418538	0.00000000
N	1.25359689	2.96921021	0.00000000
H	6.01546686	-0.82638110	0.00000000
H	4.66003477	-1.96041300	0.00000000
H	5.59754734	1.42652009	0.00000000
H	0.00000000	0.00000000	0.00000000

H	-0.82080301	2.43110636	0.00000000
O	7.82914031	-0.56146045	0.00000000
N	7.12312219	3.99336689	0.00000000
N	7.44732185	1.71143725	0.00000000
C	9.39682962	3.17534130	0.00000000
C	8.25529553	0.61773344	0.00000000
N	9.64656408	0.82885228	0.00000000
C	10.19907071	2.07631739	0.00000000
C	7.97505830	2.95606283	0.00000000
H	9.81255981	4.17627118	0.00000000
H	10.23317680	0.00000000	0.00000000
H	11.28250062	2.12612238	0.00000000
H	6.09985058	3.83894401	0.00000000
H	7.48159458	4.93915143	0.00000000

Dp–Px//G–C

Total bonding energy = -10308.80 kcal mol^{-1}

H	0.89638086	-1.58870619	3.40000000
H	7.37140625	-1.37066422	3.40000000
H	2.87170741	-3.20496799	3.40000000
C	4.52889651	2.33461377	3.40000000
N	5.63360974	3.11940510	3.40000000
H	6.30795197	8.44233667	4.28722551
H	6.30795197	8.44233667	2.51277449
N	5.52348534	4.45511084	3.40000000
H	6.92398807	1.04687886	3.40000000
C	4.30377705	5.01760504	3.40000000
C	3.12147559	4.27077263	3.40000000
C	3.23235795	2.89065655	3.40000000
C	6.08622513	0.36129068	3.40000000
C	6.34283705	-1.01647237	3.40000000
N	5.38403565	-1.97085807	3.40000000
C	4.14601650	-1.47734451	3.40000000
C	3.75199083	-0.10521822	3.40000000
C	4.77332027	0.87162890	3.40000000
N	2.95851556	-2.19438207	3.40000000
C	1.93413531	-1.28003563	3.40000000
N	2.35623724	-0.02284860	3.40000000
C	10.84037277	5.20735451	3.40000000
C	9.42574201	5.19776897	3.40000000
C	9.01548710	3.83387513	3.40000000
C	10.18631676	3.07488430	3.40000000
N	11.27858898	3.92400183	3.40000000
O	9.37885757	0.92765111	3.40000000
H	7.99969645	3.45949611	3.40000000
H	12.24056719	3.60190708	3.40000000
H	11.52674159	6.04189047	3.40000000
N	10.38431261	1.68168991	3.40000000
O	11.57554413	1.25370801	3.40000000
C	8.59532544	6.34526755	3.40000000
C	7.86349993	7.31312081	3.40000000
C	6.95486679	8.45405292	3.40000000

H	2.15258788	4.76305898	3.40000000
H	2.36578077	2.23964951	3.40000000
H	4.29568885	6.10564291	3.40000000
H	7.50762122	9.40199514	3.40000000
N	5.00124589	-1.00838958	0.00000000
O	4.31439792	3.57512911	0.00000000
N	4.56316571	1.27825998	0.00000000
C	4.08363083	-0.01712178	0.00000000
N	2.77812701	-0.31202208	0.00000000
C	1.98907622	0.78520120	0.00000000
C	2.37521426	2.13786499	0.00000000
C	3.76977416	2.44419170	0.00000000
N	0.61018743	0.81038579	0.00000000
C	0.22256913	2.14418538	0.00000000
N	1.25359689	2.96921021	0.00000000
H	6.01546686	-0.82638110	0.00000000
H	4.66003477	-1.96041300	0.00000000
H	5.59754734	1.42652009	0.00000000
H	0.00000000	0.00000000	0.00000000
H	-0.82080301	2.43110636	0.00000000
O	7.82914031	-0.56146045	0.00000000
N	7.12312219	3.99336689	0.00000000
N	7.44732185	1.71143725	0.00000000
C	9.39682962	3.17534130	0.00000000
C	8.25529553	0.61773344	0.00000000
N	9.64656408	0.82885228	0.00000000
C	10.19907071	2.07631739	0.00000000
C	7.97505830	2.95606283	0.00000000
H	9.81255981	4.17627118	0.00000000
H	10.23317680	0.00000000	0.00000000
H	11.28250062	2.12612238	0.00000000
H	6.09985058	3.83894401	0.00000000
H	7.48159458	4.93915143	0.00000000

Dss–Px//G–C

Total bonding energy = -11079.71 kcal mol^{-1}

H	1.41921568	1.87498650	-3.40000000
H	7.80183590	0.74221165	-3.40000000
H	3.60260523	3.19975129	-3.40000000
C	3.12523032	-2.94734083	-3.40000000
C	3.02221783	-4.35590978	-3.40000000
C	4.24885227	-5.00945017	-3.40000000
S	5.56099711	-3.84040236	-3.40000000
C	4.43299293	-2.47990352	-3.40000000
H	7.03170899	-1.56812783	-3.40000000
H	2.27996725	-2.26977300	-3.40000000
H	2.07544947	-4.88731309	-3.40000000
H	3.87648937	-9.93716405	-3.40000000
C	6.28326976	-0.78232829	-3.40000000
C	6.73237744	0.54397888	-3.40000000
N	5.92152678	1.62802717	-3.40000000
C	4.62331337	1.31062154	-3.40000000

C	4.04042034	0.01221907	-3.40000000
C	4.90788843	-1.10948967	-3.40000000
N	3.54711139	2.18705160	-3.40000000
C	2.40346935	1.42395881	-3.40000000
N	2.64827709	0.12044293	-3.40000000
C	11.86917177	-5.41000071	-3.40000000
C	10.49114229	-5.73156867	-3.40000000
C	9.77829221	-4.50059112	-3.40000000
C	10.73767213	-3.48888392	-3.40000000
N	11.99524176	-4.05992881	-3.40000000
O	9.43906546	-1.60279703	-3.40000000
H	8.70777146	-4.35923014	-3.40000000
H	12.85688563	-3.52387699	-3.40000000
H	12.73035119	-6.06223120	-3.40000000
N	10.59702999	-2.08740815	-3.40000000
O	11.64966298	-1.38816253	-3.40000000
C	9.96993640	-7.04873667	-3.40000000
C	9.58522649	-8.20020242	-3.40000000
C	9.15094442	-9.59245973	-3.40000000
C	4.51236947	-6.42778733	-3.40000000
C	5.72562119	-7.10285858	-3.40000000
C	5.58381678	-8.51865366	-3.40000000
C	4.27037139	-8.92887140	-3.40000000
S	3.17696434	-7.58047037	-3.40000000
H	8.55020308	-9.82241876	-4.28914528
H	8.55020308	-9.82241876	-2.51085472
H	6.68700163	-6.59849652	-3.40000000
H	6.41748370	-9.21007326	-3.40000000
H	10.01696453	-10.26628960	-3.40000000
N	5.00124589	1.00838958	-0.00000000
O	4.31439792	-3.57512911	-0.00000000
N	4.56316571	-1.27825998	-0.00000000
C	4.08363083	0.01712178	-0.00000000
N	2.77812701	0.31202208	-0.00000000
C	1.98907622	-0.78520120	-0.00000000
C	2.37521426	-2.13786499	-0.00000000
C	3.76977416	-2.44419170	-0.00000000
N	0.61018743	-0.81038579	-0.00000000
C	0.22256913	-2.14418538	-0.00000000
N	1.25359689	-2.96921021	-0.00000000
H	6.01546686	0.82638110	-0.00000000
H	4.66003477	1.96041300	-0.00000000
H	5.59754734	-1.42652009	-0.00000000
H	0.00000000	-0.00000000	-0.00000000
H	-0.82080301	-2.43110636	-0.00000000
O	7.82914031	0.56146045	-0.00000000
N	7.12312219	-3.99336689	-0.00000000
N	7.44732185	-1.71143725	-0.00000000
C	9.39682962	-3.17534130	-0.00000000
C	8.25529553	-0.61773344	-0.00000000
N	9.64656408	-0.82885228	-0.00000000
C	10.19907071	-2.07631739	-0.00000000

C	7.97505830	-2.95606283	-0.00000000
H	9.81255981	-4.17627118	-0.00000000
H	10.23317680	-0.00000000	-0.00000000
H	11.28250062	-2.12612238	-0.00000000
H	6.09985058	-3.83894401	-0.00000000
H	7.48159458	-4.93915143	-0.00000000

Dt–Px//G–C

Total bonding energy = -9945.46 kcal mol^{-1}

H	0.69207161	1.68348304	-3.40000000
H	7.11527504	0.83283002	-3.40000000
H	2.81892344	3.09874698	-3.40000000
N	2.67833126	-3.01487858	-3.40000000
C	2.64637386	-4.38905373	-3.40000000
C	3.86938913	-5.01534493	-3.40000000
S	5.13947762	-3.84639072	-3.40000000
C	3.91394840	-2.55285535	-3.40000000
H	6.44625763	-1.50864741	-3.40000000
H	5.72175514	-7.90965179	-4.28881576
H	1.68984893	-4.90180489	-3.40000000
H	4.08227995	-6.07542839	-3.40000000
C	5.66315816	-0.75817751	-3.40000000
C	6.05654284	0.58498073	-3.40000000
N	5.19386735	1.62633975	-3.40000000
C	3.91317856	1.25404953	-3.40000000
C	3.38148675	-0.07299380	-3.40000000
C	4.30544365	-1.14463247	-3.40000000
N	2.80426204	2.08455919	-3.40000000
C	1.69410717	1.27307859	-3.40000000
N	1.98894858	-0.01944500	-3.40000000
C	10.54562241	-5.10502955	-3.40000000
C	9.13790945	-4.96679976	-3.40000000
C	8.86178976	-3.57266138	-3.40000000
C	10.09072004	-2.92055698	-3.40000000
N	11.09937465	-3.86490552	-3.40000000
O	9.42993822	-0.72957655	-3.40000000
H	7.89344366	-3.10272078	-3.40000000
H	12.08776873	-3.63546331	-3.40000000
H	11.15364850	-5.99805109	-3.40000000
N	10.38730652	-1.54367875	-3.40000000
O	11.60238987	-1.19957115	-3.40000000
C	8.19131015	-6.02041986	-3.40000000
C	7.36210162	-6.90694799	-3.40000000
C	6.36336304	-7.96976950	-3.40000000
H	5.72175514	-7.90965179	-2.51118424
H	6.84052224	-8.95759434	-3.40000000
N	5.00124589	1.00838958	-0.00000000
O	4.31439792	-3.57512911	-0.00000000
N	4.56316571	-1.27825998	-0.00000000
C	4.08363083	0.01712178	-0.00000000
N	2.77812701	0.31202208	-0.00000000
C	1.98907622	-0.78520120	-0.00000000

C	2.37521426	-2.13786499	-0.00000000
C	3.76977416	-2.44419170	-0.00000000
N	0.61018743	-0.81038579	-0.00000000
C	0.22256913	-2.14418538	-0.00000000
N	1.25359689	-2.96921021	-0.00000000
H	6.01546686	0.82638110	-0.00000000
H	4.66003477	1.96041300	-0.00000000
H	5.59754734	-1.42652009	-0.00000000
H	0.00000000	-0.00000000	-0.00000000
H	-0.82080301	-2.43110636	-0.00000000
O	7.82914031	0.56146045	-0.00000000
N	7.12312219	-3.99336689	-0.00000000
N	7.44732185	-1.71143725	-0.00000000
C	9.39682962	-3.17534130	-0.00000000
C	8.25529553	-0.61773344	-0.00000000
N	9.64656408	-0.82885228	-0.00000000
C	10.19907071	-2.07631739	-0.00000000
C	7.97505830	-2.95606283	-0.00000000
H	9.81255981	-4.17627118	-0.00000000
H	10.23317680	-0.00000000	-0.00000000
H	11.28250062	-2.12612238	-0.00000000
H	6.09985058	-3.83894401	-0.00000000
H	7.48159458	-4.93915143	-0.00000000

Yo–Px/G–C

Total bonding energy = -10204.04 kcal mol^{-1}

H	0.72923755	-1.69358245	3.40000000
H	7.22454471	-1.31556606	3.40000000
H	2.80154839	-3.15214058	3.40000000
C	3.28074720	3.17335610	3.40000000
C	3.71376226	4.53820010	3.40000000
C	5.08033329	4.51361241	3.40000000
O	5.53159526	3.21272894	3.40000000
C	4.40947298	2.38057668	3.40000000
H	6.81938384	1.10049066	3.40000000
H	2.25744049	2.83082159	3.40000000
H	3.08733844	5.42058711	3.40000000
H	5.84920720	5.27305427	3.40000000
C	5.96484851	0.43449866	3.40000000
C	6.20041662	-0.94707976	3.40000000
N	5.22927542	-1.88489682	3.40000000
C	3.98750959	-1.38570578	3.40000000
C	3.60186491	-0.00309812	3.40000000
C	4.65303820	0.95207017	3.40000000
N	2.82911573	-2.13955549	3.40000000
C	1.73370110	-1.29299227	3.40000000
C	2.16427917	0.01990238	3.40000000
C	10.70175549	5.20174691	3.40000000
C	9.28846354	5.14899986	3.40000000
C	8.92317073	3.77369629	3.40000000
C	10.11330959	3.05000197	3.40000000
N	11.17798748	3.93131431	3.40000000

O	9.34408105	0.89279513	3.40000000
H	7.92539358	3.36158896	3.40000000
H	12.14993351	3.63985877	3.40000000
H	11.36219394	6.05674962	3.40000000
N	10.33874671	1.65949714	3.40000000
O	11.53602521	1.25505356	3.40000000
C	8.41754187	6.26638330	3.40000000
C	7.66068312	7.21564630	3.40000000
C	6.73812275	8.34559883	3.40000000
H	6.09242924	8.32886682	4.28775433
H	6.09242924	8.32886682	2.51224567
H	1.50994471	0.87880309	3.40000000
H	7.28449889	9.29695689	3.40000000
N	5.00124589	-1.00838958	0.00000000
O	4.31439792	3.57512911	0.00000000
N	4.56316571	1.27825998	0.00000000
C	4.08363083	-0.01712178	0.00000000
N	2.77812701	-0.31202208	0.00000000
C	1.98907622	0.78520120	0.00000000
C	2.37521426	2.13786499	0.00000000
C	3.76977416	2.44419170	0.00000000
N	0.61018743	0.81038579	0.00000000
C	0.22256913	2.14418538	0.00000000
N	1.25359689	2.96921021	0.00000000
H	6.01546686	-0.82638110	0.00000000
H	4.66003477	-1.96041300	0.00000000
H	5.59754734	1.42652009	0.00000000
H	0.00000000	0.00000000	0.00000000
H	-0.82080301	2.43110636	0.00000000
O	7.82914031	-0.56146045	0.00000000
N	7.12312219	3.99336689	0.00000000
N	7.44732185	1.71143725	0.00000000
C	9.39682962	3.17534130	0.00000000
C	8.25529553	0.61773344	0.00000000
N	9.64656408	0.82885228	0.00000000
C	10.19907071	2.07631739	0.00000000
C	7.97505830	2.95606283	0.00000000
H	9.81255981	4.17627118	0.00000000
H	10.23317680	0.00000000	0.00000000
H	11.28250062	2.12612238	0.00000000
H	6.09985058	3.83894401	0.00000000
H	7.48159458	4.93915143	0.00000000

Ps–Px//G–C

Total bonding energy = -9947.83 kcal mol^{-1}

H	0.58669695	1.57432000	-3.40000000
H	6.80903106	-0.18585388	-3.40000000
H	2.87118114	2.71943015	-3.40000000
C	2.01946641	-3.44604120	-3.40000000
C	1.91838728	-4.86280185	-3.40000000
C	3.15108254	-5.48269659	-3.40000000
S	4.46008925	-4.34812407	-3.40000000

C	3.33374701	-2.99943819	-3.40000000
H	5.31497243	-7.96111952	-4.28870847
H	1.17642352	-2.76494849	-3.40000000
H	0.97762170	-5.40464621	-3.40000000
H	3.35862555	-6.54475995	-3.40000000
N	5.20308185	-1.48374287	-3.40000000
C	5.72393446	-0.24447406	-3.40000000
N	5.05593516	0.92969282	-3.40000000
C	3.72957556	0.75520507	-3.40000000
C	3.04577663	-0.48790403	-3.40000000
C	3.84967017	-1.64713567	-3.40000000
N	2.73585184	1.71393023	-3.40000000
C	1.52947016	1.04217414	-3.40000000
N	1.66580457	-0.27491482	-3.40000000
C	10.02334591	-5.05426175	-3.40000000
C	8.61879860	-4.88460077	-3.40000000
C	8.36576001	-3.48276615	-3.40000000
C	9.61647306	-2.86224433	-3.40000000
N	10.60360657	-3.82978073	-3.40000000
O	9.06806649	-0.63489226	-3.40000000
H	7.40369819	-2.98619351	-3.40000000
H	11.59623301	-3.61969109	-3.40000000
H	10.60932588	-5.96194173	-3.40000000
N	9.97772306	-1.50115812	-3.40000000
O	11.20985602	-1.21738312	-3.40000000
C	7.68169365	-5.94697689	-3.40000000
C	6.90388959	-6.87876852	-3.40000000
C	5.95889192	-7.98946924	-3.40000000
H	5.31497243	-7.96111952	-2.51129153
H	6.48468148	-8.95230380	-3.40000000
N	5.00124589	1.00838958	-0.00000000
O	4.31439792	-3.57512911	-0.00000000
N	4.56316571	-1.27825998	-0.00000000
C	4.08363083	0.01712178	-0.00000000
N	2.77812701	0.31202208	-0.00000000
C	1.98907622	-0.78520120	-0.00000000
C	2.37521426	-2.13786499	-0.00000000
C	3.76977416	-2.44419170	-0.00000000
N	0.61018743	-0.81038579	-0.00000000
C	0.22256913	-2.14418538	-0.00000000
N	1.25359689	-2.96921021	-0.00000000
H	6.01546686	0.82638110	-0.00000000
H	4.66003477	1.96041300	-0.00000000
H	5.59754734	-1.42652009	-0.00000000
H	0.00000000	-0.00000000	-0.00000000
H	-0.82080301	-2.43110636	-0.00000000
O	7.82914031	0.56146045	-0.00000000
N	7.12312219	-3.99336689	-0.00000000
N	7.44732185	-1.71143725	-0.00000000
C	9.39682962	-3.17534130	-0.00000000
C	8.25529553	-0.61773344	-0.00000000
N	9.64656408	-0.82885228	-0.00000000

C	10.19907071	-2.07631739	-0.00000000
C	7.97505830	-2.95606283	-0.00000000
H	9.81255981	-4.17627118	-0.00000000
H	10.23317680	-0.00000000	-0.00000000
H	11.28250062	-2.12612238	-0.00000000
H	6.09985058	-3.83894401	-0.00000000
H	7.48159458	-4.93915143	-0.00000000

Es–Px//G–C

Total bonding energy = -10046.32 kcal mol^{-1}

H	0.52830361	1.66868676	-3.40000000
H	6.71893861	-0.29375890	-3.40000000
H	2.87785226	2.61427102	-3.40000000
C	2.00602837	-3.66154585	-3.40000000
C	1.97284108	-5.08287005	-3.40000000
C	3.23291167	-5.64155333	-3.40000000
S	4.48304159	-4.44167293	-3.40000000
C	3.29391240	-3.14567895	-3.40000000
H	0.69618830	-1.01648950	-3.40000000
H	1.11336744	-3.04944947	-3.40000000
H	1.05748461	-5.66670290	-3.40000000
H	3.49217841	-6.69220581	-3.40000000
N	5.12216877	-1.60643443	-3.40000000
C	5.63432743	-0.36329244	-3.40000000
N	4.95187685	0.79833742	-3.40000000
C	3.62369065	0.62631998	-3.40000000
C	2.93523205	-0.63117396	-3.40000000
C	3.77080749	-1.77294740	-3.40000000
N	2.67644561	1.62110701	-3.40000000
C	1.41139964	1.04494728	-3.40000000
C	1.52850786	-0.32891833	-3.40000000
C	9.99360780	-5.13705861	-3.40000000
C	8.58716048	-4.98280846	-3.40000000
C	8.31905702	-3.58384418	-3.40000000
C	9.56318652	-2.94989918	-3.40000000
N	10.56067270	-3.90645705	-3.40000000
O	8.99068670	-0.72876856	-3.40000000
H	7.35370463	-3.09367542	-3.40000000
H	11.55130915	-3.68736428	-3.40000000
H	10.58950469	-6.03827747	-3.40000000
N	9.90970932	-1.58483961	-3.40000000
O	11.13887553	-1.28807471	-3.40000000
C	7.66593589	-6.05940461	-3.40000000
C	6.91353667	-7.01197582	-3.40000000
C	6.00461490	-8.15244402	-3.40000000
H	5.35999251	-8.14549529	-2.51133619
H	5.35999251	-8.14549529	-4.28866381
H	6.56222002	-9.09719500	-3.40000000
N	5.00124589	1.00838958	-0.00000000
O	4.31439792	-3.57512911	-0.00000000
N	4.56316571	-1.27825998	-0.00000000
C	4.08363083	0.01712178	-0.00000000

N	2.77812701	0.31202208	-0.00000000
C	1.98907622	-0.78520120	-0.00000000
C	2.37521426	-2.13786499	-0.00000000
C	3.76977416	-2.44419170	-0.00000000
N	0.61018743	-0.81038579	-0.00000000
C	0.22256913	-2.14418538	-0.00000000
N	1.25359689	-2.96921021	-0.00000000
H	6.01546686	0.82638110	-0.00000000
H	4.66003477	1.96041300	-0.00000000
H	5.59754734	-1.42652009	-0.00000000
H	0.00000000	-0.00000000	-0.00000000
H	-0.82080301	-2.43110636	-0.00000000
O	7.82914031	0.56146045	-0.00000000
N	7.12312219	-3.99336689	-0.00000000
N	7.44732185	-1.71143725	-0.00000000
C	9.39682962	-3.17534130	-0.00000000
C	8.25529553	-0.61773344	-0.00000000
N	9.64656408	-0.82885228	-0.00000000
C	10.19907071	-2.07631739	-0.00000000
C	7.97505830	-2.95606283	-0.00000000
H	9.81255981	-4.17627118	-0.00000000
H	10.23317680	-0.00000000	-0.00000000
H	11.28250062	-2.12612238	-0.00000000
H	6.09985058	-3.83894401	-0.00000000
H	7.48159458	-4.93915143	-0.00000000

Ysm–Px//G–C

Total bonding energy = -10514.40 kcal mol^{-1}

H	0.93937617	-1.81151198	3.40000000
H	7.35967169	-0.76518238	3.40000000
H	3.15378955	-3.04363789	3.40000000
C	2.95346139	3.25519769	3.40000000
C	3.00081372	4.67824226	3.40000000
C	4.27822235	5.19323758	3.40000000
S	5.45102360	3.89889142	3.40000000
C	4.19890640	2.64803230	3.40000000
H	6.71447267	1.58361597	3.40000000
H	2.02298040	2.70436866	3.40000000
H	2.11628576	5.30924849	3.40000000
C	4.71520463	6.62780560	3.40000000
C	5.92291801	0.84087881	3.40000000
C	6.30270568	-0.50653087	3.40000000
N	5.43135567	-1.53746329	3.40000000
C	4.14672233	-1.16312138	3.40000000
C	3.62004355	0.17502686	3.40000000
C	4.56780304	1.23685675	3.40000000
N	3.07402374	-2.03383887	3.40000000
C	1.89614039	-1.30754385	3.40000000
C	2.18685958	0.04264137	3.40000000
C	11.16178663	5.40684505	3.40000000
C	9.75017957	5.49762942	3.40000000
C	9.25040105	4.16638291	3.40000000

C	10.35973062	3.32496890	3.40000000
N	11.50770332	4.09400123	3.40000000
O	9.36718129	1.26129492	3.40000000
H	8.21696996	3.85911984	3.40000000
H	12.44580988	3.70741005	3.40000000
H	11.90615069	6.18985731	3.40000000
N	10.43772467	1.91845950	3.40000000
O	11.58554346	1.39009114	3.40000000
C	8.99387450	6.69556560	3.40000000
C	8.34436784	7.72133266	3.40000000
C	7.56765405	8.95606565	3.40000000
H	6.92453127	9.01919928	2.51296580
H	5.32122049	6.86420423	4.28307911
H	3.83738113	7.28174305	3.40000000
H	5.32122049	6.86420423	2.51692089
H	1.44015852	0.82165811	3.40000000
H	6.92453127	9.01919928	4.28703420
H	8.22711834	9.83284872	3.40000000
N	5.00124589	-1.00838958	0.00000000
O	4.31439792	3.57512911	0.00000000
N	4.56316571	1.27825998	0.00000000
C	4.08363083	-0.01712178	0.00000000
N	2.77812701	-0.31202208	0.00000000
C	1.98907622	0.78520120	0.00000000
C	2.37521426	2.13786499	0.00000000
C	3.76977416	2.44419170	0.00000000
N	0.61018743	0.81038579	0.00000000
C	0.22256913	2.14418538	0.00000000
N	1.25359689	2.96921021	0.00000000
H	6.01546686	-0.82638110	0.00000000
H	4.66003477	-1.96041300	0.00000000
H	5.59754734	1.42652009	0.00000000
H	0.00000000	0.00000000	0.00000000
H	-0.82080301	2.43110636	0.00000000
O	7.82914031	-0.56146045	0.00000000
N	7.12312219	3.99336689	0.00000000
N	7.44732185	1.71143725	0.00000000
C	9.39682962	3.17534130	0.00000000
C	8.25529553	0.61773344	0.00000000
N	9.64656408	0.82885228	0.00000000
C	10.19907071	2.07631739	0.00000000
C	7.97505830	2.95606283	0.00000000
H	9.81255981	4.17627118	0.00000000
H	10.23317680	0.00000000	0.00000000
H	11.28250062	2.12612238	0.00000000
H	6.09985058	3.83894401	0.00000000
H	7.48159458	4.93915143	0.00000000

Yss–Px//G–C

Total bonding energy = -11176.45 kcal mol^{-1}

H	1.32319487	2.01494820	-3.40000000
H	7.67007002	0.58624311	-3.40000000

H	3.60797587	3.11198295	-3.40000000
C	3.03396385	-3.15796575	-3.40000000
C	2.98539517	-4.57078072	-3.40000000
C	4.23541254	-5.17425929	-3.40000000
S	5.49703199	-3.95168677	-3.40000000
C	4.31828877	-2.63045530	-3.40000000
H	6.88478198	-1.72004568	-3.40000000
H	2.14207983	-2.54730965	-3.40000000
H	2.05865543	-5.13633918	-3.40000000
C	4.55853553	-6.58049009	-3.40000000
C	6.14092312	-0.93015962	-3.40000000
C	6.59964698	0.39191552	-3.40000000
N	5.79017928	1.47252968	-3.40000000
C	4.48572843	1.17501244	-3.40000000
C	3.87948164	-0.12920114	-3.40000000
C	4.76495719	-1.24508100	-3.40000000
N	3.46725847	2.10883329	-3.40000000
C	2.24832197	1.45510704	-3.40000000
C	2.45726995	0.08951329	-3.40000000
C	11.86546532	-5.47377960	-3.40000000
C	10.50166035	-5.85064451	-3.40000000
C	9.73986592	-4.64911512	-3.40000000
C	10.65767397	-3.59966276	-3.40000000
N	11.93711172	-4.11966404	-3.40000000
O	9.28391176	-1.76764675	-3.40000000
H	8.66472020	-4.54927991	-3.40000000
H	12.77636518	-3.54924179	-3.40000000
H	12.75228660	-6.09069897	-3.40000000
N	10.46025067	-2.20482748	-3.40000000
O	11.48408188	-1.46396795	-3.40000000
C	10.03440007	-7.18802447	-3.40000000
C	9.69697919	-8.35425853	-3.40000000
C	9.31794521	-9.76280445	-3.40000000
C	5.79955996	-7.20200323	-3.40000000
C	5.72053158	-8.62287537	-3.40000000
C	4.42680386	-9.09061979	-3.40000000
S	3.27521447	-7.79055243	-3.40000000
H	6.73830913	-6.65699914	-3.40000000
H	8.72615290	-10.01602709	-4.28897279
H	8.72615290	-10.01602709	-2.51102721
H	6.58539013	-9.27496943	-3.40000000
H	4.07672411	-10.11492851	-3.40000000
H	1.66438256	-0.64240648	-3.40000000
H	10.20942269	-10.40246102	-3.40000000
N	5.00124589	1.00838958	-0.00000000
O	4.31439792	-3.57512911	-0.00000000
N	4.56316571	-1.27825998	-0.00000000
C	4.08363083	0.01712178	-0.00000000
N	2.77812701	0.31202208	-0.00000000
C	1.98907622	-0.78520120	-0.00000000
C	2.37521426	-2.13786499	-0.00000000
C	3.76977416	-2.44419170	-0.00000000

N	0.61018743	-0.81038579	-0.00000000
C	0.22256913	-2.14418538	-0.00000000
N	1.25359689	-2.96921021	-0.00000000
H	6.01546686	0.82638110	-0.00000000
H	4.66003477	1.96041300	-0.00000000
H	5.59754734	-1.42652009	-0.00000000
H	0.00000000	-0.00000000	-0.00000000
H	-0.82080301	-2.43110636	-0.00000000
O	7.82914031	0.56146045	-0.00000000
N	7.12312219	-3.99336689	-0.00000000
N	7.44732185	-1.71143725	-0.00000000
C	9.39682962	-3.17534130	-0.00000000
C	8.25529553	-0.61773344	-0.00000000
N	9.64656408	-0.82885228	-0.00000000
C	10.19907071	-2.07631739	-0.00000000
C	7.97505830	-2.95606283	-0.00000000
H	9.81255981	-4.17627118	-0.00000000
H	10.23317680	-0.00000000	-0.00000000
H	11.28250062	-2.12612238	-0.00000000
H	6.09985058	-3.83894401	-0.00000000
H	7.48159458	-4.93915143	-0.00000000

S11.3. Methanol

Cartesian Coordinates and Total Bonding Energies of W–C base pairs in Methanol

Cartesian coordinates (Å) and total bonding energies (kcal mol^{-1}) of Watson–Crick base pairs optimized at ZORA-BLYP-D3(BJ)/TZ2P level of theory in methanol.

A–T

Total bonding energy = -4510.04 kcal mol^{-1}

N	1.28664527	-0.60410982	0.00000000
C	1.89799075	-1.80541234	0.00000000
N	3.21679087	-2.06144814	0.00000000
C	3.94618187	-0.92381830	0.00000000
C	3.44311964	0.38912680	0.00000000
C	2.03489965	0.53666844	0.00000000
N	5.31905402	-0.77889344	0.00000000
C	5.58607521	0.57541900	0.00000000
N	4.48543219	1.31576287	0.00000000
N	1.40382911	1.72558303	0.00000000
H	1.22882925	-2.66467185	0.00000000
H	6.00573487	-1.52598919	0.00000000
H	6.60008676	0.95315129	0.00000000
H	1.94366363	2.58092030	0.00000000
H	0.37734480	1.76472705	0.00000000
N	-1.57735283	-0.57226485	0.00000000
C	-2.19826557	-1.80536641	0.00000000
N	-3.58211631	-1.73640205	0.00000000
C	-4.28293751	-0.55259183	0.00000000
C	-3.66214619	0.65827888	0.00000000
C	-2.20694062	0.67325553	0.00000000
O	-1.51675499	1.71906837	0.00000000
O	-1.57860908	-2.88057948	0.00000000
C	-4.40989810	1.96393642	0.00000000
H	-0.52427499	-0.58351783	0.00000000
H	-4.08045947	-2.62074514	0.00000000
H	-5.36287022	-0.65721931	0.00000000
H	-4.15140028	2.56305855	0.88203001
H	-5.49025144	1.79101500	0.00000000
H	-4.15140028	2.56305855	-0.88203001

G–C

Total bonding energy = -4420.18 kcal mol^{-1}

N	-2.55817896	-0.57187466	0.00000000
O	1.37057010	1.88794719	0.00000000
N	-0.63512248	0.73949267	0.00000000
C	-2.01484180	0.66332972	0.00000000
N	-2.80613159	1.74449894	0.00000000
C	-2.11611217	2.90650694	0.00000000
C	-0.72165469	3.08742734	0.00000000
C	0.11310526	1.93144668	0.00000000
N	-2.63964147	4.18157665	0.00000000
C	-1.57291872	5.06646976	0.00000000

N	-0.40379199	4.44798502	0.00000000
H	-1.98578337	-1.42964235	0.00000000
H	-3.56751600	-0.64093021	0.00000000
H	-0.08752096	-0.15185567	0.00000000
H	-3.62514608	4.42370706	0.00000000
H	-1.72539663	6.13750969	0.00000000
O	-1.04316267	-2.98947972	0.00000000
N	2.87331468	-0.55631542	0.00000000
N	0.89799715	-1.74748493	0.00000000
C	3.00963065	-2.96659931	0.00000000
C	0.21321735	-2.92144353	0.00000000
N	0.94916860	-4.11519624	0.00000000
C	2.31300264	-4.13565770	0.00000000
C	2.25168482	-1.74665321	0.00000000
H	4.09321175	-2.95546250	0.00000000
H	0.41968547	-4.98185173	0.00000000
H	2.78156609	-5.11325232	0.00000000
H	2.33257638	0.32189540	0.00000000
H	3.88418855	-0.51609365	0.00000000

A–T//G–C

Total bonding energy = -8940.66 kcal mol^{-1}

N	5.25852145	-0.85339338	-3.40000000
C	5.61245179	0.44722354	-3.40000000
N	4.80736243	1.52268586	-3.40000000
C	3.50288987	1.16974928	-3.40000000
C	2.99375112	-0.14085142	-3.40000000
C	3.93795899	-1.19599291	-3.40000000
N	2.38843458	1.98447043	-3.40000000
C	1.28098165	1.16044950	-3.40000000
N	1.59915939	-0.12729544	-3.40000000
N	3.60696268	-2.50068037	-3.40000000
H	6.68533449	0.63437782	-3.40000000
H	2.38148285	2.99917942	-3.40000000
H	0.27602911	1.56166204	-3.40000000
H	2.63252851	-2.77179142	-3.40000000
H	4.34116040	-3.21912036	-3.40000000
N	7.35897579	-2.80059413	-3.40000000
C	8.64720403	-2.30406610	-3.40000000
N	9.62613404	-3.28462460	-3.40000000
C	9.35024209	-4.63237852	-3.40000000
C	8.07703518	-5.11251800	-3.40000000
C	6.98886161	-4.14622309	-3.40000000
O	5.77510404	-4.45746779	-3.40000000
O	8.91028887	-1.09128266	-3.40000000
C	7.75407376	-6.58206570	-3.40000000
H	6.58634275	-2.08495614	-3.40000000
H	10.58931041	-2.96415637	-3.40000000
H	10.22060229	-5.28020156	-3.40000000
H	7.16016001	-6.85231542	-2.51796999
H	8.67061530	-7.17957449	-3.40000000
H	7.16016001	-6.85231542	-4.28203001

N	5.01072929	0.99340199	-0.00000000
O	4.30311303	-3.58754463	-0.00000000
N	4.56576767	-1.29129485	-0.00000000
C	4.09065743	0.00627844	-0.00000000
N	2.78482728	0.30606844	-0.00000000
C	1.98994643	-0.78688802	-0.00000000
C	2.37464339	-2.13938648	-0.00000000
C	3.76637283	-2.44955519	-0.00000000
N	0.61177148	-0.80968027	-0.00000000
C	0.22028517	-2.13921842	-0.00000000
N	1.25033840	-2.96889971	-0.00000000
H	6.02485359	0.80644199	-0.00000000
H	4.67541456	1.94791441	-0.00000000
H	5.60094570	-1.44221118	-0.00000000
H	0.00000000	-0.00000000	-0.00000000
H	-0.82386801	-2.42227350	-0.00000000
O	7.83019934	0.55673593	-0.00000000
N	7.14222402	-4.00240580	-0.00000000
N	7.45611902	-1.71718501	-0.00000000
C	9.41029341	-3.17541626	-0.00000000
C	8.26404832	-0.62432069	-0.00000000
N	9.65144142	-0.82879605	-0.00000000
C	10.20904031	-2.07360344	-0.00000000
C	7.99014840	-2.96108357	-0.00000000
H	9.82814656	-4.17525163	-0.00000000
H	10.23841777	-0.00000000	-0.00000000
H	11.29222369	-2.11783835	-0.00000000
H	6.12182623	-3.85260423	-0.00000000
H	7.50463415	-4.94693884	-0.00000000

Cartesian Coordinates and Total Bonding Energies of Ds variants and Px in Methanol

Cartesian coordinates (Å) and total bonding energies (kcal mol^{-1}) of Ds variants and Px optimized at ZORA-BLYP-D3(BJ)/TZ2P level of theory in methanol.

Ds

Total bonding energy = -3175.18 kcal mol^{-1}

H	6.88743505	-0.60518163	0.00000000
H	1.82036587	3.43264879	0.00000000
H	6.43843586	1.90288600	0.00000000
C	2.13445353	-2.52361803	0.00000000
C	1.13126269	-3.53177124	0.00000000
C	-0.14569018	-3.01495898	0.00000000
S	-0.14084938	-1.28307038	0.00000000
C	1.62296624	-1.23248393	0.00000000
H	0.59169243	1.31896816	0.00000000
H	3.20043821	-2.71609023	0.00000000
H	1.34240588	-4.59685495	0.00000000
H	-1.08951322	-3.54565145	0.00000000
C	1.67755738	1.28249624	0.00000000
C	2.37513263	2.49627193	0.00000000
N	3.72459117	2.60490866	0.00000000
C	4.34626809	1.42122825	0.00000000
C	3.75478500	0.12556818	0.00000000
C	2.34133975	0.03368078	0.00000000
N	5.71345358	1.19310278	0.00000000
C	5.89758376	-0.16706038	0.00000000
N	4.75727263	-0.84727348	0.00000000

Bs

Total bonding energy = -3276.22 kcal mol^{-1}

H	6.87787536	-0.68687742	0.00000000
H	1.70872793	3.44538909	0.00000000
H	6.51566787	1.82563488	0.00000000
C	2.14757881	-2.50588602	0.00000000
C	1.14586655	-3.51963732	0.00000000
C	-0.13209842	-3.01101526	0.00000000
S	-0.12954915	-1.27542952	0.00000000
C	1.63668238	-1.21580154	0.00000000
H	0.56867026	1.28574879	0.00000000
H	3.21345190	-2.69682181	0.00000000
H	1.36320727	-4.58377761	0.00000000
H	-1.07476600	-3.54371564	0.00000000
C	1.65587115	1.29277402	0.00000000
C	2.30819866	2.53846933	0.00000000
C	3.70188326	2.63538422	0.00000000
C	4.40165629	1.42620606	0.00000000
C	3.76584214	0.15098906	0.00000000
C	2.34638455	0.06261082	0.00000000
N	5.76195514	1.14763208	0.00000000
C	5.90310615	-0.21576254	0.00000000
N	4.74074705	-0.85517376	0.00000000

H	4.21046360	3.59542776	0.00000000

Is

Total bonding energy = -3371.03 kcal mol^{-1}

H	7.07343485	-0.50054044	0.00000000
H	1.69277883	3.40624807	0.00000000
H	6.46848311	1.94597801	0.00000000
C	2.07801893	-2.55107580	0.00000000
C	1.04169455	-3.53002963	0.00000000
C	-0.21652754	-2.97755694	0.00000000
S	-0.14952181	-1.24393892	0.00000000
C	1.62162386	-1.24026996	0.00000000
H	0.57038492	1.23711247	0.00000000
H	3.12455597	-2.81969907	0.00000000
H	1.22488569	-4.60050524	0.00000000
H	-1.17740141	-3.47660387	0.00000000
C	1.65746792	1.25095460	0.00000000
C	2.29730505	2.50264867	0.00000000
C	3.68859374	2.59979098	0.00000000
C	4.40591213	1.40000178	0.00000000
C	3.78590967	0.10238491	0.00000000
C	2.35773444	0.02791262	0.00000000
N	5.77635257	1.20705515	0.00000000
C	6.05195261	-0.14521016	0.00000000
C	4.86638473	-0.85381799	0.00000000
H	4.19495849	3.56188375	0.00000000
H	4.81067933	-1.93077888	0.00000000

Ys

Total bonding energy = -3271.26 kcal mol^{-1}

H	7.06895475	-0.42519693	0.00000000
H	1.80472903	3.39356585	0.00000000
H	6.38683701	2.01091541	0.00000000
C	2.06476334	-2.56771043	0.00000000
C	1.02780677	-3.54202513	0.00000000
C	-0.22949953	-2.98263519	0.00000000
S	-0.16147860	-1.25262153	0.00000000
C	1.60618055	-1.25721601	0.00000000
H	0.58970715	1.27052432	0.00000000
H	3.11268962	-2.83290733	0.00000000
H	1.20549455	-4.61311279	0.00000000
H	-1.19123319	-3.48005590	0.00000000
C	1.67566394	1.24043977	0.00000000
C	2.36327404	2.45907940	0.00000000
N	3.70956170	2.56780816	0.00000000
C	4.35109436	1.39266324	0.00000000
C	3.77087886	0.07541322	0.00000000
C	2.35006612	-0.00033625	0.00000000
N	5.72325909	1.24506877	0.00000000
C	6.03734375	-0.10143767	0.00000000
C	4.87530273	-0.84790415	0.00000000
H	4.84973328	-1.92656636	0.00000000

Yi

Total bonding energy = -3362.53 kcal mol^{-1}

H	7.01164227	-0.47851328	0.00000000
H	1.72858066	3.32014120	0.00000000
H	6.33119381	1.95652226	0.00000000
N	2.16684111	-2.56056002	0.00000000
C	1.12195006	-3.46461282	0.00000000
C	-0.08930042	-2.79808096	0.00000000
N	0.23089401	-1.45926390	0.00000000
C	1.60561611	-1.33900175	0.00000000
H	0.52500826	1.19369745	0.00000000
H	-0.44063971	-0.70017883	0.00000000
H	1.29343229	-4.53351348	0.00000000
H	-1.11299311	-3.14321984	0.00000000
C	1.61060362	1.16554421	0.00000000
C	2.29339923	2.38957581	0.00000000
N	3.63906999	2.50874296	0.00000000
C	4.29372032	1.34038132	0.00000000
C	3.72009855	0.02273139	0.00000000
C	2.30551252	-0.06110262	0.00000000
N	5.66558054	1.19249603	0.00000000
C	5.97871635	-0.15825552	0.00000000
C	4.81638725	-0.90483834	0.00000000
H	4.74666908	-1.98191046	0.00000000

Do

Total bonding energy = -3233.49 kcal mol^{-1}

H	0.16963542	4.14736082	0.00000000
H	-3.71390769	-1.04150519	0.00000000
H	-2.32307797	3.62891664	0.00000000
C	2.24831311	-0.61557650	0.00000000
C	3.09600431	-1.76829656	0.00000000
C	2.27073631	-2.85991502	0.00000000
O	0.95240916	-2.46348505	0.00000000
C	0.94573051	-1.06781835	0.00000000
H	-1.55054444	-2.20715192	0.00000000
H	2.54494806	0.42315203	0.00000000
H	4.17804791	-1.78453076	0.00000000
H	2.43944094	-3.92703204	0.00000000
C	-1.56027921	-1.12226887	0.00000000
C	-2.79439366	-0.45898873	0.00000000
N	-2.94566027	0.88664314	0.00000000
C	-1.78355514	1.54880305	0.00000000
C	-0.47223283	0.99839220	0.00000000
C	-0.33970465	-0.40930433	0.00000000
N	-1.59496544	2.92241120	0.00000000
C	-0.24010853	3.14543655	0.00000000
N	0.47316406	2.02475774	0.00000000

Dp

Total bonding energy = -3437.19 kcal mol^{-1}

H	-0.05513510	4.17517625	0.00000000
H	-3.92171768	-1.02086759	0.00000000
H	-2.54475388	3.64141493	0.00000000
N	1.80727603	-3.20038413	0.00000000
C	3.00453044	-2.59032040	0.00000000
C	3.14612746	-1.19931940	0.00000000
C	1.99251164	-0.43521791	0.00000000
H	4.13151431	-0.74215665	0.00000000
H	-1.74635447	-2.18111755	0.00000000
H	2.01873928	0.64782732	0.00000000
H	3.86594704	-3.25437157	0.00000000
C	0.75344889	-1.10950158	0.00000000
C	-1.76534783	-1.09746997	0.00000000
C	-3.00196500	-0.43923679	0.00000000
N	-3.14935186	0.90571870	0.00000000
C	-1.99156913	1.56706157	0.00000000
C	-0.67277965	1.01686463	0.00000000
C	-0.54598284	-0.39051265	0.00000000
N	-1.81124285	2.94041747	0.00000000
C	-0.45993736	3.17130423	0.00000000
N	0.26098786	2.05654273	0.00000000
N	0.68505465	-2.46185166	0.00000000

Dss

Total bonding energy = -4207.45 kcal mol^{-1}

H	-0.51627342	6.88715812	0.00000000
H	3.40513031	1.72785330	0.00000000
H	1.97983925	6.38206006	0.00000000
C	-2.54144709	2.17657162	0.00000000
C	-3.56754495	1.20525293	0.00000000
C	-3.09950187	-0.10393701	0.00000000
S	-1.34321568	-0.12434799	0.00000000
C	-1.25947733	1.63976057	0.00000000
H	1.26476284	0.54745202	0.00000000
H	-2.71166325	3.24633693	0.00000000
H	-4.62524104	1.45024455	0.00000000
C	-3.85058680	-1.33632431	0.00000000
C	1.25297945	1.63391976	0.00000000
C	2.48157501	2.30369477	0.00000000
N	2.62117492	3.65084609	0.00000000
C	1.45164963	4.29970954	0.00000000
C	0.14310792	3.73866053	0.00000000
C	0.01805004	2.32645865	0.00000000
N	1.25514461	5.67205670	0.00000000
C	-0.10036218	5.88783814	0.00000000
N	-0.80625344	4.76295479	0.00000000
C	-3.38520681	-2.64428862	0.00000000
C	-4.42460962	-3.61637816	0.00000000
C	-5.68210641	-3.05568053	0.00000000
S	-5.61349104	-1.32009711	0.00000000
H	-6.64456797	-3.55127589	0.00000000
H	-2.32837286	-2.89323440	0.00000000

H	-4.25044438	-4.68801117	0.00000000

Dt

Total bonding energy = -3074.29 kcal mol^{-1}

H	-0.59290206	6.86331864	0.00000000
H	3.43132545	1.78773423	0.00000000
H	1.91205359	6.40512414	0.00000000
N	-2.43387292	2.10489670	0.00000000
C	-3.41292839	1.13902802	0.00000000
C	-2.96770451	-0.16116131	0.00000000
S	-1.24193013	-0.20158700	0.00000000
C	-1.22277089	1.57411546	0.00000000
H	1.31464883	0.56222653	0.00000000
N	-0.84686866	4.73585365	0.00000000
H	-4.45688087	1.43636514	0.00000000
H	-3.54723287	-1.07515024	0.00000000
C	1.28032782	1.64786060	0.00000000
C	2.49507798	2.34213238	0.00000000
N	2.59989412	3.69119265	0.00000000
C	1.42054870	4.31556459	0.00000000
C	0.11753329	3.72839613	0.00000000
C	0.03657334	2.31650449	0.00000000
N	1.20002862	5.68232567	0.00000000
C	-0.15881430	5.87161036	0.00000000

Yo

Total bonding energy = -3330.05 kcal mol^{-1}

H	0.00434705	4.31104114	0.00000000
H	-3.67954039	-1.05226119	0.00000000
H	-2.41446778	3.57299532	0.00000000
C	2.28596411	-0.69015773	0.00000000
C	3.09656189	-1.86978852	0.00000000
C	2.23648306	-2.93266111	0.00000000
O	0.93284455	-2.49171674	0.00000000
C	0.96715270	-1.09520275	0.00000000
H	-1.50991192	-2.21366909	0.00000000
H	2.64514958	0.32751183	0.00000000
H	4.17756885	-1.91900794	0.00000000
H	2.36996361	-4.00472660	0.00000000
C	-1.52355881	-1.12905882	0.00000000
C	-2.75954488	-0.46991367	0.00000000
N	-2.90569173	0.87327877	0.00000000
C	-1.74983238	1.55052808	0.00000000
C	-0.42067541	1.00594837	0.00000000
C	-0.30921307	-0.40972768	0.00000000
N	-1.63431338	2.92634283	0.00000000
C	-0.29439174	3.27187958	0.00000000
C	0.47923930	2.12702206	0.00000000
H	1.55893005	2.11693862	0.00000000

Ps

Total bonding energy = -3074.31 kcal mol^{-1}

H	6.94907919	-0.53715009	0.00000000
H	1.71268067	3.25107123	0.00000000
H	6.43053630	1.95969804	0.00000000
C	2.13111754	-2.53213277	0.00000000
C	1.09241219	-3.50063362	0.00000000
C	-0.16449167	-2.93103559	0.00000000
S	-0.10263975	-1.20128013	0.00000000
C	1.65701599	-1.22674339	0.00000000
N	4.82803899	-0.84082870	0.00000000
H	3.18999732	-2.76277627	0.00000000
H	1.26226731	-4.57292528	0.00000000
H	-1.12549237	-3.43052624	0.00000000
N	1.68696733	1.18322473	0.00000000
C	2.33715806	2.35895890	0.00000000
N	3.67066620	2.56521678	0.00000000
C	4.35782480	1.41528357	0.00000000
C	3.80450152	0.10832172	0.00000000
C	2.39636540	0.01808018	0.00000000
N	5.72420634	1.23095791	0.00000000
C	5.94676856	-0.12893291	0.00000000

Es

Total bonding energy = -3171.98 kcal mol^{-1}

H	-0.35030229	7.07470818	0.00000000
H	3.20408927	1.64284631	0.00000000
H	2.06584017	6.32258940	0.00000000
C	-2.57982641	2.02903494	0.00000000
C	-3.51842154	0.96171085	0.00000000
C	-2.91257335	-0.27669287	0.00000000
S	-1.18529150	-0.15957771	0.00000000
C	-1.25973792	1.59974099	0.00000000
C	-0.84010473	4.89555625	0.00000000
H	-2.87081730	3.07133037	0.00000000
H	-4.59490362	1.10250670	0.00000000
H	-3.38350157	-1.25200416	0.00000000
N	1.13361780	1.63304102	0.00000000
C	2.31549714	2.27268500	0.00000000
N	2.52468886	3.60241887	0.00000000
C	1.38629813	4.31138267	0.00000000
C	0.05707941	3.77137404	0.00000000
C	-0.02010044	2.35811757	0.00000000
N	1.28250525	5.67917537	0.00000000
C	-0.06030907	6.03332224	0.00000000
H	-1.91957362	4.89263606	0.00000000

Ysm

Total bonding energy = -3639.96 kcal mol^{-1}

H	3.02790120	2.64662083	0.00000000
H	1.43714287	-3.66059380	0.00000000
H	4.06690148	0.33931155	0.00000000
C	-2.19021521	1.07826688	0.00000000
C	-3.60887446	1.15821947	0.00000000

C	-4.23436407	-0.07159204	0.00000000
S	-3.05204133	-1.35355022	0.00000000
C	-1.69601664	-0.21923853	0.00000000
H	-0.85622773	-2.81061496	0.00000000
H	-1.55874526	1.95608674	0.00000000
H	-4.15648378	2.09681485	0.00000000
H	0.36180386	2.37357586	0.00000000
C	-0.04204689	-2.09122598	0.00000000
C	1.26685971	-2.58521326	0.00000000
N	2.37035459	-1.80583772	0.00000000
C	2.10828496	-0.49203033	0.00000000
C	0.81904839	0.14605699	0.00000000
C	-0.32153980	-0.70589844	0.00000000
N	3.06666851	0.50150403	0.00000000
C	2.44423808	1.73645080	0.00000000
C	1.07411675	1.56311322	0.00000000
C	-5.70623090	-0.37848661	0.00000000
H	-6.19119524	0.05042488	0.88562310
H	-5.89439397	-1.45622973	0.00000000
H	-6.19119524	0.05042488	-0.88562310

Yss

Total bonding energy = -4303.50 kcal mol^{-1}

H	-0.27608062	7.07787434	0.00000000
H	3.33702516	1.66954158	0.00000000
H	2.13137371	6.30231549	0.00000000
C	-2.60860437	2.15692096	0.00000000
C	-3.61789808	1.16670121	0.00000000
C	-3.12651392	-0.13202428	0.00000000
S	-1.37089621	-0.11651031	0.00000000
C	-1.31411236	1.65210072	0.00000000
H	1.16997312	0.53645579	0.00000000
H	-2.83496175	3.21394601	0.00000000
H	-4.67921864	1.39518075	0.00000000
C	-3.85353140	-1.37929070	0.00000000
C	1.18137057	1.62285690	0.00000000
C	2.42467938	2.26351299	0.00000000
N	2.58536929	3.60494832	0.00000000
C	1.43541910	4.29145187	0.00000000
C	0.09700540	3.76282307	0.00000000
C	-0.03411932	2.34467574	0.00000000
N	1.34080096	5.66845459	0.00000000
C	0.00705943	6.03440735	0.00000000
C	-0.78306119	4.90116673	0.00000000
C	-3.36238796	-2.67772076	0.00000000
C	-4.38268687	-3.67040584	0.00000000
C	-5.65104088	-3.13492917	0.00000000
S	-5.61603995	-1.39756661	0.00000000
H	-2.30082353	-2.90554018	0.00000000
H	-4.18756160	-4.73849573	0.00000000
H	-6.60353228	-3.64931931	0.00000000
H	-1.86198174	4.91743257	0.00000000

Px

Total bonding energy = -2447.74 kcal mol^{-1}

C	-1.36560804	-3.84051042	0.00000000
H	-0.99184028	-4.36748369	0.88729825
H	-2.46028704	-3.91281507	0.00000000
H	-0.99184028	-4.36748369	-0.88729825
C	-1.02001055	1.20219839	0.00000000
C	-0.16762688	0.07060450	0.00000000
C	1.16624451	0.55798679	0.00000000
C	1.07968552	1.95095421	0.00000000
N	-0.25293965	2.31791058	0.00000000
O	3.29195371	2.54270144	0.00000000
H	2.07515731	-0.02485082	0.00000000
H	-0.57912448	3.27886319	0.00000000
H	-2.09889623	1.25599260	0.00000000
N	2.08982674	2.92261491	0.00000000
O	1.74983975	4.14160363	0.00000000
C	-0.58717231	-1.28263584	0.00000000
C	-0.93736187	-2.44565074	0.00000000

Cartesian Coordinates and Total Bonding Energies of Ds–Px variants in Methanol

Cartesian coordinates (Å) and total bonding energies (kcal mol^{-1}) of Ds–Px variants optimized at ZORA-BLYP-D3(BJ)/TZ2P level of theory in methanol.

Ds–Px

Total bonding energy = -5625.56 kcal mol^{-1}

H	6.91257440	-0.94505651	0.00000000
H	1.96971181	3.24605789	0.00000000
H	6.54231894	1.57527334	0.00000000
C	2.10319801	-2.71644921	0.00000000
C	1.06871366	-3.69242284	0.00000000
C	-0.19125432	-3.13663778	0.00000000
S	-0.13148733	-1.40640045	0.00000000
C	1.63267971	-1.41017034	0.00000000
H	0.68376769	1.18070409	0.00000000
H	3.16232846	-2.94329857	0.00000000
H	1.24573250	-4.76352881	0.00000000
H	-1.14861675	-3.64036539	0.00000000
C	1.76580553	1.10304005	0.00000000
C	2.49847492	2.29551890	0.00000000
N	3.85069984	2.36170246	0.00000000
C	4.43540991	1.15949774	0.00000000
C	3.80416003	-0.11767850	0.00000000
C	2.38879377	-0.16612193	0.00000000
N	5.79492405	0.88917844	0.00000000
C	5.93703680	-0.47598460	0.00000000
N	4.77629499	-1.12073959	0.00000000
C	-4.72510244	1.73120340	0.00000000
C	-3.66883469	0.78737411	0.00000000
C	-2.45730828	1.52681024	0.00000000
C	-2.81118455	2.87483997	0.00000000
N	-4.18962483	2.97610159	0.00000000
O	-0.74891972	3.86665521	0.00000000
H	-1.45526477	1.13260756	0.00000000
H	-4.69695668	3.85493602	0.00000000
H	-5.79390979	1.57454429	0.00000000
N	-1.99995482	4.01899497	0.00000000
O	-2.56148587	5.15290250	0.00000000
C	-3.80765038	-0.62243352	0.00000000
C	-3.91267963	-1.83225618	0.00000000
C	-4.04038864	-3.28531188	0.00000000
H	-3.56831731	-3.72378879	0.88868981
H	-5.09536693	-3.58550909	0.00000000
H	-3.56831731	-3.72378879	-0.88868981

Bs–Px

Total bonding energy = -5726.74 kcal mol^{-1}

H	-1.05695348	6.80934750	0.00000000
H	3.20379203	1.74346179	0.00000000
H	1.46489770	6.51101163	0.00000000
C	-2.75519105	2.03482285	0.00000000

C	-3.74259629	1.00725861	0.00000000
C	-3.20206727	-0.25720801	0.00000000
S	-1.46737166	-0.20956773	0.00000000
C	-1.45242658	1.55736009	0.00000000
H	1.08210210	0.55648453	0.00000000
H	-2.97334060	3.09542470	0.00000000
H	-4.81201881	1.19627932	0.00000000
H	-3.71335237	-1.21022183	0.00000000
C	1.05588812	1.64196288	0.00000000
C	2.28508258	2.32345460	0.00000000
C	2.34602545	3.71932745	0.00000000
C	1.11919330	4.38793498	0.00000000
C	-0.13972698	3.71996337	0.00000000
C	-0.19226292	2.29880336	0.00000000
N	0.80567275	5.74084774	0.00000000
C	-0.56065498	5.84716677	0.00000000
N	-1.17019256	4.66881585	0.00000000
C	1.57566266	-4.87971572	0.00000000
C	0.65645960	-3.80161032	0.00000000
C	1.42368509	-2.60729248	0.00000000
C	2.76334890	-2.99266552	0.00000000
N	2.83217676	-4.37348566	0.00000000
O	3.80688569	-0.95609355	0.00000000
H	1.05147215	-1.59629660	0.00000000
H	3.69941958	-4.90040317	0.00000000
H	1.39342268	-5.94447581	0.00000000
N	3.92707918	-2.21057445	0.00000000
O	5.04680632	-2.80083637	0.00000000
C	-0.75622509	-3.90981334	0.00000000
C	-1.96768378	-3.99282420	0.00000000
C	-3.42252112	-4.09466440	0.00000000
H	-3.85313840	-3.61482698	0.88844665
H	3.29279551	4.25210265	0.00000000
H	-3.85313840	-3.61482698	-0.88844665
H	-3.74100600	-5.14442750	0.00000000

Is–Px

Total bonding energy = -5821.56 kcal mol^{-1}

H	-0.78572403	6.91407289	0.00000000
H	3.20972506	1.59572208	0.00000000
H	1.67116409	6.35123256	0.00000000
C	-2.75153031	1.88138361	0.00000000
C	-3.71055197	0.82641953	0.00000000
C	-3.13536946	-0.42102961	0.00000000
S	-1.40337099	-0.32076188	0.00000000
C	-1.43240925	1.45014969	0.00000000
H	1.06758736	0.44503521	0.00000000
H	-3.04013447	2.92259536	0.00000000
H	-4.78425052	0.98880525	0.00000000
H	-3.61854494	-1.38852691	0.00000000
C	1.05840246	1.53067770	0.00000000
C	2.29947966	2.18908594	0.00000000

C	2.37274706	3.58203612	0.00000000
C	1.16073207	4.27920809	0.00000000
C	-0.12653078	3.63728155	0.00000000
C	-0.17718490	2.20807035	0.00000000
N	0.94425423	5.64652418	0.00000000
C	-0.41260406	5.89890288	0.00000000
C	-1.10089232	4.70128568	0.00000000
C	1.63475247	-5.00143334	0.00000000
C	0.71279788	-3.92533075	0.00000000
C	1.47706233	-2.72938001	0.00000000
C	2.81804877	-3.11182427	0.00000000
N	2.88996195	-4.49240262	0.00000000
O	3.85759358	-1.07326522	0.00000000
H	1.10550323	-1.71800395	0.00000000
H	3.75796216	-5.01812446	0.00000000
H	1.45478295	-6.06657516	0.00000000
N	3.98012240	-2.32753169	0.00000000
O	5.10104097	-2.91556076	0.00000000
C	-0.69935634	-4.03901287	0.00000000
C	-1.91015798	-4.13210487	0.00000000
C	-3.36401212	-4.24885812	0.00000000
H	3.32617564	4.10444358	0.00000000
H	-3.79991657	-3.77380137	-0.88837562
H	-3.79991657	-3.77380137	0.88837562
H	-2.17666529	4.62649572	0.00000000
H	-3.67077345	-5.30209884	0.00000000

Ys–Px

Total bonding energy = -5721.65 kcal mol^{-1}

H	-0.61868301	7.00559804	0.00000000
H	3.27574787	1.79500572	0.00000000
H	1.82731299	6.35963208	0.00000000
C	-2.68788372	1.97093755	0.00000000
C	-3.64663438	0.91925401	0.00000000
C	-3.06966005	-0.32945495	0.00000000
S	-1.34105103	-0.23540769	0.00000000
C	-1.37092302	1.53197434	0.00000000
H	1.17825796	0.55589777	0.00000000
H	-2.96917153	3.01461874	0.00000000
H	-4.72014179	1.08068663	0.00000000
H	-3.55538407	-1.29562134	0.00000000
C	1.12669701	1.63933998	0.00000000
C	2.33577528	2.34287742	0.00000000
N	2.42417533	3.69059112	0.00000000
C	1.23968560	4.31471553	0.00000000
C	-0.06923165	3.71519893	0.00000000
C	-0.12436310	2.29346515	0.00000000
N	1.07156424	5.68462108	0.00000000
C	-0.27972082	5.97879789	0.00000000
C	-1.00873124	4.80551672	0.00000000
C	1.73658653	-4.87450915	0.00000000
C	0.80469520	-3.80722725	0.00000000

C	1.55769213	-2.60449523	0.00000000
C	2.90185214	-2.97373498	0.00000000
N	2.98719972	-4.35344864	0.00000000
O	3.91896938	-0.92380722	0.00000000
H	1.17517262	-1.59807964	0.00000000
H	3.86030639	-4.87062225	0.00000000
H	1.56694059	-5.94133172	0.00000000
N	4.05557675	-2.17663461	0.00000000
O	5.18260358	-2.75230631	0.00000000
C	-0.60628679	-3.93334352	0.00000000
C	-1.81635338	-4.03532765	0.00000000
C	-3.26945463	-4.16169839	0.00000000
H	-3.70785339	-3.68947121	0.88870235
H	-2.08684196	4.76369330	0.00000000
H	-3.70785339	-3.68947121	-0.88870235
H	-3.57058820	-5.21642916	0.00000000

Yi–Px

Total bonding energy = -5812.03 kcal mol^{-1}

H	-6.71449122	-0.86458975	0.00000000
H	-0.47243558	-2.69364110	0.00000000
H	-5.26214436	-2.93351988	0.00000000
N	-2.84063730	2.70715062	0.00000000
C	-2.16227789	3.90986143	0.00000000
C	-0.79858630	3.69097297	0.00000000
N	-0.64541712	2.32113363	0.00000000
C	-1.90188467	1.74628045	0.00000000
H	-0.04775252	-0.26878096	0.00000000
H	-5.07987026	1.30785238	0.00000000
H	-2.68355171	4.85859346	0.00000000
H	0.04305438	4.36602795	0.00000000
C	-1.07599698	-0.61597852	0.00000000
C	-1.31019127	-1.99789722	0.00000000
N	-2.53963517	-2.55708599	0.00000000
C	-3.54616851	-1.67381211	0.00000000
C	-3.44436575	-0.24025118	0.00000000
C	-2.13820796	0.30914118	0.00000000
N	-4.88869455	-1.99153096	0.00000000
C	-5.63390579	-0.82201141	0.00000000
C	-4.78672967	0.26913717	0.00000000
C	4.80665486	-1.16917056	0.00000000
C	3.50080023	-0.61847053	0.00000000
C	2.59546759	-1.71014520	0.00000000
C	3.36729509	-2.87064136	0.00000000
N	4.70370072	-2.51972100	0.00000000
O	1.73738030	-4.47680704	0.00000000
H	1.51993197	-1.67203887	0.00000000
H	5.46917516	-3.18607614	0.00000000
H	5.76654720	-0.67387599	0.00000000
N	2.97033640	-4.21588377	0.00000000
O	3.86859188	-5.10634755	0.00000000
C	3.19829688	0.76601882	0.00000000

C	3.00678959	1.96595626	0.00000000
C	2.82487394	3.41410459	0.00000000
H	2.27640905	3.74642849	-0.89027134
H	0.24425472	1.83153167	0.00000000
H	2.27640905	3.74642849	0.89027134
H	3.79697555	3.92165753	0.00000000

Do–Px

Total bonding energy = -5684.28 kcal mol^{-1}

H	1.09504764	6.93470046	0.00000000
H	-3.49309150	2.35651357	0.00000000
H	-1.44631247	6.77986891	0.00000000
C	2.46677254	1.91712087	0.00000000
C	3.13688211	0.65223705	0.00000000
C	2.16171366	-0.30727777	0.00000000
O	0.91539698	0.27907211	0.00000000
C	1.11210303	1.66122327	0.00000000
H	-1.53072881	0.89555495	0.00000000
H	2.91236768	2.90126708	0.00000000
H	4.20516585	0.47931242	0.00000000
H	2.16536260	-1.38789980	0.00000000
C	-1.37458249	1.96794198	0.00000000
C	-2.50030255	2.80204792	0.00000000
N	-2.45683325	4.15558569	0.00000000
C	-1.21146681	4.64369770	0.00000000
C	0.00706681	3.91046751	0.00000000
C	-0.06496061	2.49836161	0.00000000
N	-0.82726348	5.97608190	0.00000000
C	0.54565842	6.00204859	0.00000000
N	1.09026320	4.79029533	0.00000000
C	-1.42036454	-4.74016882	0.00000000
C	-0.44908544	-3.70883223	0.00000000
C	-1.15334276	-2.47539808	0.00000000
C	-2.51140684	-2.79604685	0.00000000
N	-2.64903104	-4.17190524	0.00000000
O	-3.46977683	-0.71608393	0.00000000
H	-0.73056311	-1.48227948	0.00000000
H	-3.54212465	-4.65373845	0.00000000
H	-1.29062717	-5.81257741	0.00000000
N	-3.64056732	-1.96457622	0.00000000
O	-4.78441026	-2.50705270	0.00000000
C	0.95540411	-3.89439052	0.00000000
C	2.16102791	-4.04185078	0.00000000
C	3.61093274	-4.20260885	0.00000000
H	4.05941647	-3.73823947	-0.88779001
H	4.05941647	-3.73823947	0.88779001
H	3.88684357	-5.26423287	0.00000000

Dp–Px

Total bonding energy = -5886.19 kcal mol^{-1}

H	0.89779815	6.97442272	0.00000000
H	-3.63107379	2.34318811	0.00000000

H	-1.64034054	6.78008286	0.00000000
C	0.98315004	1.62448372	0.00000000
N	0.72287041	0.29501543	0.00000000
H	3.95007469	-3.97354946	0.88716044
H	3.95007469	-3.97354946	-0.88716044
N	1.72365399	-0.59800600	0.00000000
H	-1.63678968	0.90353364	0.00000000
C	2.99532389	-0.16600678	0.00000000
C	3.33752932	1.19001700	0.00000000
C	2.30630901	2.11346285	0.00000000
C	-1.50550456	1.97827391	0.00000000
C	-2.64200967	2.79652367	0.00000000
N	-2.60792718	4.14969298	0.00000000
C	-1.37156194	4.64953017	0.00000000
C	-0.13883224	3.92734338	0.00000000
C	-0.20271181	2.51603579	0.00000000
N	-1.00820930	5.98649124	0.00000000
C	0.36207723	6.03386767	0.00000000
N	0.92676468	4.83224911	0.00000000
C	-1.55397323	-4.84098286	0.00000000
C	-0.54814240	-3.84299008	0.00000000
C	-1.20867686	-2.58375320	0.00000000
C	-2.57812738	-2.86108431	0.00000000
N	-2.76270216	-4.23256358	0.00000000
O	-3.48955728	-0.75679278	0.00000000
H	-0.74616932	-1.60515731	0.00000000
H	-3.67203051	-4.68274925	0.00000000
H	-1.46077504	-5.91735961	0.00000000
N	-3.68541503	-2.00190607	0.00000000
O	-4.84350045	-2.51742103	0.00000000
C	0.84830368	-4.08156581	0.00000000
C	2.04928803	-4.25798803	0.00000000
C	3.49721661	-4.43533108	0.00000000
H	4.37873943	1.50019890	0.00000000
H	2.48763037	3.18167786	0.00000000
H	3.75117649	-0.94832548	0.00000000
H	3.76604957	-5.49900888	0.00000000

Dss–Px

Total bonding energy = -6657.69 kcal mol^{-1}

H	-7.28780978	2.99954815	0.00000000
H	-1.02811199	4.68152455	0.00000000
H	-5.87833363	5.12097198	0.00000000
C	-3.68731337	-0.64929995	0.00000000
C	-3.17758008	-1.96645126	0.00000000
C	-1.78892779	-2.03117925	0.00000000
S	-1.10374356	-0.41291610	0.00000000
C	-2.70585869	0.33379005	0.00000000
H	-0.74409770	2.26309992	0.00000000
H	-4.74177049	-0.40141070	0.00000000
H	-3.80587715	-2.85183516	0.00000000
H	-0.01202824	-6.64155917	0.00000000

C	-1.75496234	2.65671574	0.00000000
C	-1.91163644	4.04708114	0.00000000
N	-3.10690355	4.68477201	0.00000000
C	-4.14899323	3.84634566	0.00000000
C	-4.12308013	2.42276959	0.00000000
C	-2.86158293	1.77565087	0.00000000
N	-5.49433514	4.18192952	0.00000000
C	-6.20544252	3.00795232	0.00000000
N	-5.42985039	1.92989332	0.00000000
C	5.27962401	0.89276485	0.00000000
C	4.17281817	0.00747058	0.00000000
C	3.00126130	0.81005256	0.00000000
C	3.43220888	2.13837939	0.00000000
N	4.81381130	2.16301712	0.00000000
O	1.44301713	3.27211076	0.00000000
H	1.97432100	0.47668338	0.00000000
H	5.36695997	3.01387614	0.00000000
H	6.33760957	0.67497767	0.00000000
N	2.70024275	3.33458177	0.00000000
O	3.33988611	4.42741312	0.00000000
C	4.27084349	-1.40576055	0.00000000
C	4.42013775	-2.61102778	0.00000000
C	4.62716430	-4.05490173	0.00000000
C	-0.94287930	-3.19967721	0.00000000
C	0.44285055	-3.28809698	0.00000000
C	0.92270501	-4.62773431	0.00000000
C	-0.08810520	-5.56172522	0.00000000
S	-1.65403536	-4.81404553	0.00000000
H	4.18327095	-4.52003326	-0.88922480
H	4.18327095	-4.52003326	0.88922480
H	1.09382430	-2.41921459	0.00000000
H	1.97202013	-4.89469469	0.00000000
H	5.69858915	-4.29049719	0.00000000

Dt–Px

Total bonding energy = -5524.45 kcal mol^{-1}

H	-0.97355119	7.01006620	0.00000000
H	3.14111359	2.00574661	0.00000000
H	1.53989325	6.59708756	0.00000000
N	-2.72821956	2.21646023	0.00000000
C	-3.68806152	1.23103499	0.00000000
C	-3.21785585	-0.05984542	0.00000000
S	-1.49245217	-0.06674872	0.00000000
C	-1.50736008	1.71072265	0.00000000
H	1.05464216	0.74897983	0.00000000
H	-3.81212334	-3.41615686	-0.88881855
H	-4.73793646	1.50570858	0.00000000
H	-3.78360296	-0.98123921	0.00000000
C	0.99476106	1.83190830	0.00000000
C	2.19747185	2.54605712	0.00000000
N	2.27703383	3.89678508	0.00000000
C	1.08628296	4.49936484	0.00000000

C	-0.20632114	3.88880809	0.00000000
C	-0.26184730	2.47537871	0.00000000
N	0.84069204	5.86185637	0.00000000
C	-0.52145810	6.02649207	0.00000000
N	-1.18863066	4.87810425	0.00000000
C	1.62408864	-4.66868210	0.00000000
C	0.69403047	-3.59974185	0.00000000
C	1.44941950	-2.39873331	0.00000000
C	2.79288782	-2.76983802	0.00000000
N	2.87568025	-4.14967012	0.00000000
O	3.81093430	-0.72048742	0.00000000
H	1.06958040	-1.39143551	0.00000000
H	3.74774823	-4.66860267	0.00000000
H	1.45271207	-5.73519813	0.00000000
N	3.94710569	-1.97352521	0.00000000
O	5.07368358	-2.54960924	0.00000000
C	-0.71741350	-3.71907545	0.00000000
C	-1.92895385	-3.80115888	0.00000000
C	-3.38427335	-3.89765663	0.00000000
H	-3.81212334	-3.41615686	0.88881855
H	-3.70783304	-4.94569402	0.00000000

Yo–Px

Total bonding energy = -5780.80 kcal mol^{-1}

H	0.87379839	7.02633574	0.00000000
H	-3.52282865	2.22968644	0.00000000
H	-1.62462292	6.63410253	0.00000000
C	2.43387932	1.75817731	0.00000000
C	3.07361027	0.47718835	0.00000000
C	2.07592188	-0.45708007	0.00000000
O	0.84556107	0.16187868	0.00000000
C	1.07170223	1.54084430	0.00000000
H	-1.54818311	0.77888133	0.00000000
H	2.93037436	2.71638418	0.00000000
H	4.13767933	0.28032124	0.00000000
H	2.05344947	-1.53753896	0.00000000
C	-1.40002840	1.85231072	0.00000000
C	-2.53119751	2.67860170	0.00000000
N	-2.48883633	4.02917316	0.00000000
C	-1.24964523	4.53822741	0.00000000
C	-0.01003932	3.81302516	0.00000000
C	-0.09783638	2.39566452	0.00000000
N	-0.94272553	5.88456474	0.00000000
C	0.43270173	6.03910241	0.00000000
C	1.03808516	4.79684000	0.00000000
C	-1.46876571	-4.87838757	0.00000000
C	-0.49408243	-3.85035327	0.00000000
C	-1.19429049	-2.61451100	0.00000000
C	-2.55321339	-2.93070924	0.00000000
N	-2.69565830	-4.30604331	0.00000000
O	-3.50137706	-0.84660595	0.00000000
H	-0.77008913	-1.62195160	0.00000000

H	-3.59033309	-4.78494089	0.00000000
H	-1.34250481	-5.95121779	0.00000000
N	-3.67849172	-2.09404521	0.00000000
O	-4.82485188	-2.63137256	0.00000000
C	0.90983968	-4.04009813	0.00000000
C	2.11523833	-4.18805390	0.00000000
C	3.56518197	-4.34832559	0.00000000
H	4.01343093	-3.88366724	0.88776195
H	4.01343093	-3.88366724	-0.88776195
H	2.10573815	4.63545394	0.00000000
H	3.84140846	-5.40988492	0.00000000

Ps–Px

Total bonding energy = -5525.42 kcal mol^{-1}

H	-0.48896759	6.82891574	0.00000000
H	2.81854842	1.27355227	0.00000000
H	1.95103476	6.09411470	0.00000000
C	-2.90285323	2.18929616	0.00000000
C	-3.94715718	1.22610618	0.00000000
C	-3.47394336	-0.06988311	0.00000000
S	-1.74296449	-0.13915833	0.00000000
C	-1.63876143	1.61546274	0.00000000
H	-3.56729942	-3.35120418	-0.88850893
H	-3.05353988	3.26250901	0.00000000
H	-5.00365864	1.47584156	0.00000000
H	-4.04965655	-0.98644538	0.00000000
N	0.76070651	1.43411827	0.00000000
C	1.98970058	1.97677660	0.00000000
N	2.31173195	3.28886605	0.00000000
C	1.22813559	4.07546952	0.00000000
C	-0.12283103	3.63977083	0.00000000
C	-0.33572475	2.24591529	0.00000000
N	1.16440035	5.45294320	0.00000000
C	-0.17113070	5.79435773	0.00000000
N	-0.97868525	4.74277490	0.00000000
C	1.85490298	-4.47895234	0.00000000
C	0.94722788	-3.39071106	0.00000000
C	1.72329284	-2.19934960	0.00000000
C	3.06126604	-2.60568476	0.00000000
N	3.11458008	-3.98735565	0.00000000
O	4.17588183	-0.60198333	0.00000000
H	1.36331168	-1.17854317	0.00000000
H	3.97691364	-4.52231967	0.00000000
H	1.65928479	-5.54132908	0.00000000
N	4.24850664	-1.86000626	0.00000000
O	5.34889967	-2.48674960	0.00000000
C	-0.46351222	-3.51975537	0.00000000
C	-1.66960830	-3.65942721	0.00000000
C	-3.11974420	-3.81500187	0.00000000
H	-3.56729942	-3.35120418	0.88850893
H	-3.40030342	-4.87531642	0.00000000

Es–Px

Total bonding energy = -5623.08 kcal mol^{-1}

H	-0.25849657	6.87345218	0.00000000
H	2.85064294	1.17204371	0.00000000
H	2.08969603	5.93465147	0.00000000
C	-2.88104558	2.00828185	0.00000000
C	-3.89084517	1.00736561	0.00000000
C	-3.37130457	-0.26935752	0.00000000
S	-1.63885946	-0.27254321	0.00000000
C	-1.59517145	1.48687783	0.00000000
H	-1.99555462	4.81909664	0.00000000
H	-3.09967360	3.06829821	0.00000000
H	-4.95516171	1.22139494	0.00000000
H	-3.91355437	-1.20565132	0.00000000
N	0.79058055	1.33032626	0.00000000
C	2.02000637	1.87310954	0.00000000
N	2.33292877	3.18382834	0.00000000
C	1.25521938	3.98144869	0.00000000
C	-0.11235569	3.54837859	0.00000000
C	-0.30071046	2.14659113	0.00000000
N	1.25909156	5.35353836	0.00000000
C	-0.05235728	5.81226466	0.00000000
C	-0.91897104	4.73928223	0.00000000
C	1.89892340	-4.60196480	0.00000000
C	0.98705591	-3.51698716	0.00000000
C	1.75875665	-2.32288233	0.00000000
C	3.09830050	-2.72450703	0.00000000
N	3.15671825	-4.10587792	0.00000000
O	4.20542330	-0.71688336	0.00000000
H	1.39766500	-1.30256005	0.00000000
H	4.02066510	-4.63819003	0.00000000
H	1.70703502	-5.66504304	0.00000000
N	4.28282722	-1.97451435	0.00000000
O	5.38563220	-2.59733366	0.00000000
C	-0.42297040	-3.65603449	0.00000000
C	-1.62655727	-3.81636456	0.00000000
C	-3.07291351	-4.00337384	0.00000000
H	-3.53089237	-3.55008747	0.88860312
H	-3.53089237	-3.55008747	-0.88860312
H	-3.32888085	-5.06998662	0.00000000

Ysm–Px

Total bonding energy = -6091.16 kcal mol^{-1}

H	0.17623545	7.39487116	0.00000000
H	-3.65430564	2.13687644	0.00000000
H	-2.26172766	6.71933476	0.00000000
C	2.30521526	2.37882912	0.00000000
C	3.27315078	1.33535690	0.00000000
C	2.72846646	0.06975427	0.00000000
S	0.98432547	0.16208014	0.00000000
C	0.99286965	1.93253679	0.00000000
H	-1.54314174	0.92002018	0.00000000

H	2.57836995	3.42504096	0.00000000
H	4.34557239	1.50941115	0.00000000
C	3.43006957	-1.25549979	0.00000000
C	-1.50369184	2.00470748	0.00000000
C	-2.72044196	2.69542211	0.00000000
N	-2.82675618	4.04223836	0.00000000
C	-1.64954466	4.68118207	0.00000000
C	-0.33441646	4.09793152	0.00000000
C	-0.26104476	2.67639759	0.00000000
N	-1.49798690	6.05340411	0.00000000
C	-0.15005422	6.36398721	0.00000000
C	0.59250047	5.19915050	0.00000000
C	-2.03234752	-4.91994052	0.00000000
C	-0.96928284	-3.98299545	0.00000000
C	-1.55798586	-2.69134887	0.00000000
C	-2.93953754	-2.88345755	0.00000000
N	-3.20390358	-4.24043348	0.00000000
O	-3.69229533	-0.72114710	0.00000000
H	-1.04583209	-1.74267687	0.00000000
H	-4.13744776	-4.63827298	0.00000000
H	-2.00274389	-5.99970988	0.00000000
N	-3.98331564	-1.94715047	0.00000000
O	-5.17418158	-2.37699285	0.00000000
C	0.41213864	-4.29765068	0.00000000
C	1.59535433	-4.57131152	0.00000000
C	3.01494184	-4.90644111	0.00000000
H	3.51748082	-4.50039919	-0.88698396
H	3.16709856	-1.84955894	0.88354978
H	4.51344709	-1.09932348	0.00000000
H	3.16709856	-1.84955894	-0.88354978
H	1.67118275	5.16962961	0.00000000
H	3.51748082	-4.50039919	0.88698396
H	3.15898661	-5.99389351	0.00000000

Yss–Px

Total bonding energy = -6753.65 kcal mol^{-1}

H	-7.20408458	3.43577538	0.00000000
H	-0.80510272	4.60996639	0.00000000
H	-5.53949116	5.33968319	0.00000000
C	-3.60397186	-0.65083264	0.00000000
C	-3.09529629	-1.96970284	0.00000000
C	-1.70855550	-2.03531659	0.00000000
S	-1.02564682	-0.41659072	0.00000000
C	-2.62850466	0.33745777	0.00000000
H	-0.62287671	2.18066411	0.00000000
H	-4.66404738	-0.43939454	0.00000000
H	-3.72688906	-2.85269426	0.00000000
C	-0.86103568	-3.20308806	0.00000000
C	-1.61690954	2.61607358	0.00000000
C	-1.71332965	4.01128231	0.00000000
N	-2.88207491	4.68961170	0.00000000
C	-3.96743458	3.90495718	0.00000000

C	-4.01169036	2.46670439	0.00000000
C	-2.76020209	1.78671701	0.00000000
N	-5.26981738	4.36291550	0.00000000
C	-6.13366547	3.28310479	0.00000000
C	-5.40545394	2.10886649	0.00000000
C	5.43276423	0.75470749	0.00000000
C	4.32189441	-0.12521648	0.00000000
C	3.15415394	0.68320683	0.00000000
C	3.59158744	2.00937314	0.00000000
N	4.97323185	2.02727430	0.00000000
O	1.60801206	3.15266928	0.00000000
H	2.12522719	0.35586591	0.00000000
H	5.53054316	2.87541430	0.00000000
H	6.48971704	0.53184667	0.00000000
N	2.86516879	3.20923364	0.00000000
O	3.51035784	4.29894005	0.00000000
C	4.41471635	-1.53887757	0.00000000
C	4.56149835	-2.74449049	0.00000000
C	4.76630124	-4.18877369	0.00000000
C	0.52447313	-3.28898820	0.00000000
C	1.00753486	-4.62790311	0.00000000
C	-0.00105006	-5.56394011	0.00000000
S	-1.56877766	-4.81891018	0.00000000
H	1.17458626	-2.41952636	0.00000000
H	4.32000221	-4.65468926	-0.88777903
H	4.32000221	-4.65468926	0.88777903
H	2.05878051	-4.89227150	0.00000000
H	0.07632298	-6.64369732	0.00000000
H	-5.84745069	1.12443553	0.00000000
H	5.83695260	-4.42725835	0.00000000

Cartesian Coordinates and Total Bonding Energies of Ds–Px//A–T variants in Methanol

Cartesian coordinates (Å) and total bonding energies (kcal mol^{-1}) of Ds–Px//A–T variants optimized at ZORA-BLYP-D3(BJ)-TZ2P level of theory in methanol.

Ds–Px//A–T

Total bonding energy = -10148.33 kcal mol^{-1}

H	0.72912235	-1.61047166	3.40000000
H	7.15897015	-0.80152929	3.40000000
H	2.83659061	-3.04146055	3.40000000
C	2.67511608	3.13094604	3.40000000
C	2.65529017	4.55301823	3.40000000
C	3.91661045	5.10572746	3.40000000
S	5.15117212	3.89199322	3.40000000
C	3.95576314	2.59457389	3.40000000
H	6.50640341	1.54229137	3.40000000
H	1.79195212	2.50385803	3.40000000
H	1.74635043	5.14667825	3.40000000
H	4.19263332	6.15171775	3.40000000
C	5.71768406	0.79746966	3.40000000
C	6.10108310	-0.54856727	3.40000000
N	5.23570647	-1.58972806	3.40000000
C	3.95455175	-1.20785811	3.40000000
C	3.44017875	0.12070350	3.40000000
C	4.36131310	1.19639860	3.40000000
N	2.83628736	-2.02690388	3.40000000
C	1.73426235	-1.20873089	3.40000000
N	2.04385658	0.08246312	3.40000000
C	10.56861393	5.15578985	3.40000000
C	9.15906176	5.01551172	3.40000000
C	8.88490360	3.62288854	3.40000000
C	10.11746216	2.97234111	3.40000000
N	11.12394709	3.91962122	3.40000000
O	9.45415019	0.78221533	3.40000000
H	7.91701394	3.15100237	3.40000000
H	12.11450876	3.69934156	3.40000000
H	11.17572256	6.04927199	3.40000000
N	10.41214343	1.60108356	3.40000000
O	11.62730203	1.24830461	3.40000000
C	8.21405558	6.07087454	3.40000000
C	7.39357236	6.96614505	3.40000000
C	6.40918937	8.04256068	3.40000000
H	5.76695266	7.99112768	4.28868981
H	6.90117970	9.02288872	3.40000000
H	5.76695266	7.99112768	2.51131019
N	4.59205859	1.42571600	-0.00000000
C	4.11391081	0.16545978	-0.00000000
N	2.83043894	-0.23138786	-0.00000000
C	1.98254939	0.82089357	-0.00000000
C	2.34099925	2.18045605	-0.00000000
C	3.72507607	2.47909199	-0.00000000
N	0.60205504	0.81683069	-0.00000000

C	0.19045415	2.13442213	-0.00000000
N	1.20478284	2.98920948	-0.00000000
N	4.22417048	3.72916107	-0.00000000
H	4.87188462	-0.61657584	-0.00000000
H	0.00000000	-0.00000000	-0.00000000
H	-0.85839635	2.40053065	-0.00000000
H	3.59519175	4.52125255	-0.00000000
H	5.24043734	3.87884063	-0.00000000
N	7.43589774	1.76641842	-0.00000000
C	8.18624442	0.60751724	-0.00000000
N	9.55457326	0.82540511	-0.00000000
C	10.12356185	2.07792616	-0.00000000
C	9.37573473	3.21473940	-0.00000000
C	7.92740993	3.07260278	-0.00000000
O	7.12840447	4.03783382	-0.00000000
O	7.68622830	-0.52828256	-0.00000000
C	9.97823192	4.59346043	-0.00000000
H	6.39018301	1.64159743	-0.00000000
H	10.14543281	0.00000000	-0.00000000
H	11.20847888	2.09044112	-0.00000000
H	9.65659441	5.16119078	-0.88203001
H	11.07093646	4.53812560	-0.00000000
H	9.65659441	5.16119078	0.88203001

Bs–Px//A–T

Total bonding energy = -10248.97 kcal mol^{-1}

H	0.69708056	1.57789630	-3.40000000
H	7.26700786	0.76968491	-3.40000000
H	2.74698332	3.07677467	-3.40000000
C	2.70485920	-3.07494384	-3.40000000
C	2.68154503	-4.49983465	-3.40000000
C	3.93845747	-5.05770432	-3.40000000
S	5.17632455	-3.84151209	-3.40000000
C	3.98401356	-2.53742169	-3.40000000
H	6.52162052	-1.54437664	-3.40000000
H	1.82285304	-2.44682710	-3.40000000
H	1.76969123	-5.08967870	-3.40000000
H	4.21303597	-6.10377050	-3.40000000
C	5.76322730	-0.76733730	-3.40000000
C	6.19927354	0.56878158	-3.40000000
C	5.29333020	1.63247407	-3.40000000
C	3.93961358	1.28663528	-3.40000000
C	3.47259224	-0.05982579	-3.40000000
C	4.40191217	-1.13631094	-3.40000000
N	2.78870770	2.06386350	-3.40000000
C	1.71575038	1.21126876	-3.40000000
N	2.07183073	-0.06671940	-3.40000000
C	10.58503531	-5.18917231	-3.40000000
C	9.17773124	-5.02564626	-3.40000000
C	8.92625174	-3.62858215	-3.40000000
C	10.16971540	-2.99849514	-3.40000000
N	11.16043854	-3.96279240	-3.40000000

O	9.54701312	-0.79648781	-3.40000000
H	7.96520671	-3.14170682	-3.40000000
H	12.15434048	-3.75807039	-3.40000000
H	11.17666980	-6.09299424	-3.40000000
N	10.48931566	-1.63328810	-3.40000000
O	11.71124742	-1.30301432	-3.40000000
C	8.21691370	-6.06690299	-3.40000000
C	7.38629749	-6.95267930	-3.40000000
C	6.39027876	-8.01798198	-3.40000000
H	5.74817660	-7.95984413	-2.51155335
H	5.62383242	2.66736114	-3.40000000
H	5.74817660	-7.95984413	-4.28844665
H	6.87192898	-9.00360272	-3.40000000
N	4.59205859	-1.42571600	0.00000000
C	4.11391081	-0.16545978	0.00000000
N	2.83043894	0.23138786	0.00000000
C	1.98254939	-0.82089357	0.00000000
C	2.34099925	-2.18045605	0.00000000
C	3.72507607	-2.47909199	0.00000000
N	0.60205504	-0.81683069	0.00000000
C	0.19045415	-2.13442213	0.00000000
N	1.20478284	-2.98920948	0.00000000
N	4.22417048	-3.72916107	0.00000000
H	4.87188462	0.61657584	0.00000000
H	0.00000000	0.00000000	0.00000000
H	-0.85839635	-2.40053065	0.00000000
H	3.59519175	-4.52125255	0.00000000
H	5.24043734	-3.87884063	0.00000000
N	7.43589774	-1.76641842	0.00000000
C	8.18624442	-0.60751724	0.00000000
N	9.55457326	-0.82540511	0.00000000
C	10.12356185	-2.07792616	0.00000000
C	9.37573473	-3.21473940	0.00000000
C	7.92740993	-3.07260278	0.00000000
O	7.12840447	-4.03783382	0.00000000
O	7.68622830	0.52828256	0.00000000
C	9.97823192	-4.59346043	0.00000000
H	6.39018301	-1.64159743	0.00000000
H	10.14543281	-0.00000000	0.00000000
H	11.20847888	-2.09044112	0.00000000
H	9.65659441	-5.16119078	0.88203001
H	11.07093646	-4.53812560	0.00000000
H	9.65659441	-5.16119078	-0.88203001

Is–Px//A–T

Total bonding energy = -10344.32 kcal mol^{-1}

H	0.60815362	1.67489124	-3.40000000
H	7.16917191	0.57875811	-3.40000000
H	2.77552285	2.96159209	-3.40000000
C	2.65515736	-3.32532190	-3.40000000
C	2.68816887	-4.75065765	-3.40000000
C	3.96495953	-5.25738230	-3.40000000

S	5.15009158	-3.99036441	-3.40000000
C	3.90782169	-2.72793163	-3.40000000
H	6.41140654	-1.73178855	-3.40000000
H	1.72812454	-2.77032242	-3.40000000
H	1.79863530	-5.37349610	-3.40000000
H	4.28225101	-6.29122740	-3.40000000
C	5.65608348	-0.95192250	-3.40000000
C	6.10080029	0.38074437	-3.40000000
C	5.19326458	1.44001641	-3.40000000
C	3.83477033	1.10907461	-3.40000000
C	3.34524086	-0.24350671	-3.40000000
C	4.29415866	-1.31344386	-3.40000000
N	2.73508462	1.94997131	-3.40000000
C	1.57843238	1.19703372	-3.40000000
C	1.90587870	-0.14490763	-3.40000000
C	10.57808324	-5.28488597	-3.40000000
C	9.16832988	-5.14138499	-3.40000000
C	8.89705317	-3.74825640	-3.40000000
C	10.13190605	-3.10045504	-3.40000000
N	11.13604680	-4.05064945	-3.40000000
O	9.47890935	-0.90729026	-3.40000000
H	7.93052111	-3.27207000	-3.40000000
H	12.12717861	-3.83278352	-3.40000000
H	11.18228704	-6.18034891	-3.40000000
N	10.43259879	-1.73110707	-3.40000000
O	11.64985668	-1.38394735	-3.40000000
C	8.22407321	-6.19754904	-3.40000000
C	7.41143277	-7.09994709	-3.40000000
C	6.43909575	-8.18709194	-3.40000000
H	5.52345984	2.47582851	-3.40000000
H	5.79581808	-8.14367029	-4.28837562
H	5.79581808	-8.14367029	-2.51162438
H	1.17840196	-0.94093439	-3.40000000
H	6.94327239	-9.16137344	-3.40000000
N	4.59205859	-1.42571600	0.00000000
C	4.11391081	-0.16545978	0.00000000
N	2.83043894	0.23138786	0.00000000
C	1.98254939	-0.82089357	0.00000000
C	2.34099925	-2.18045605	0.00000000
C	3.72507607	-2.47909199	0.00000000
N	0.60205504	-0.81683069	0.00000000
C	0.19045415	-2.13442213	0.00000000
N	1.20478284	-2.98920948	0.00000000
N	4.22417048	-3.72916107	0.00000000
H	4.87188462	0.61657584	0.00000000
H	0.00000000	0.00000000	0.00000000
H	-0.85839635	-2.40053065	0.00000000
H	3.59519175	-4.52125255	0.00000000
H	5.24043734	-3.87884063	0.00000000
N	7.43589774	-1.76641842	0.00000000
C	8.18624442	-0.60751724	0.00000000
N	9.55457326	-0.82540511	0.00000000

C	10.12356185	-2.07792616	0.00000000
C	9.37573473	-3.21473940	0.00000000
C	7.92740993	-3.07260278	0.00000000
O	7.12840447	-4.03783382	0.00000000
O	7.68622830	0.52828256	0.00000000
C	9.97823192	-4.59346043	0.00000000
H	6.39018301	-1.64159743	0.00000000
H	10.14543281	-0.00000000	0.00000000
H	11.20847888	-2.09044112	0.00000000
H	9.65659441	-5.16119078	0.88203001
H	11.07093646	-4.53812560	0.00000000
H	9.65659441	-5.16119078	-0.88203001

Ys–Px//A–T

Total bonding energy = -10245.04 kcal mol^{-1}

H	0.64192923	1.70283481	-3.40000000
H	7.05811210	0.63070536	-3.40000000
H	2.85575822	2.92722431	-3.40000000
C	2.62777108	-3.36528416	-3.40000000
C	2.66218319	-4.78797770	-3.40000000
C	3.94237034	-5.29126122	-3.40000000
S	5.12626345	-4.02819704	-3.40000000
C	3.88275907	-2.77192143	-3.40000000
H	6.39936880	-1.71469646	-3.40000000
H	1.70296744	-2.80570264	-3.40000000
H	1.77496692	-5.41353821	-3.40000000
H	4.25941847	-6.32513055	-3.40000000
C	5.61305240	-0.96756055	-3.40000000
C	6.00020042	0.37666845	-3.40000000
N	5.13229500	1.41150514	-3.40000000
C	3.84500126	1.04350781	-3.40000000
C	3.31380257	-0.29459251	-3.40000000
C	4.25692001	-1.35990745	-3.40000000
N	2.77641701	1.91702581	-3.40000000
C	1.59673133	1.19532436	-3.40000000
C	1.88119952	-0.15638669	-3.40000000
C	10.55720163	-5.25213568	-3.40000000
C	9.14602194	-5.12531306	-3.40000000
C	8.85852238	-3.73574022	-3.40000000
C	10.08495680	-3.07319759	-3.40000000
N	11.10051831	-4.01102956	-3.40000000
O	9.40255429	-0.88892240	-3.40000000
H	7.88634360	-3.27307195	-3.40000000
H	12.08889570	-3.78104274	-3.40000000
H	11.17220631	-6.14020065	-3.40000000
N	10.36742499	-1.69963973	-3.40000000
O	11.57970300	-1.33636381	-3.40000000
C	8.21378510	-6.19194794	-3.40000000
C	7.41002472	-7.10223974	-3.40000000
C	6.44753277	-8.19817863	-3.40000000
H	5.80430536	-8.16009338	-2.51129765
H	1.13118331	-0.93198169	-3.40000000

H	5.80430536	-8.16009338	-4.28870235
H	6.95917807	-9.16841444	-3.40000000
N	4.59205859	-1.42571600	0.00000000
C	4.11391081	-0.16545978	0.00000000
N	2.83043894	0.23138786	0.00000000
C	1.98254939	-0.82089357	0.00000000
C	2.34099925	-2.18045605	0.00000000
C	3.72507607	-2.47909199	0.00000000
N	0.60205504	-0.81683069	0.00000000
C	0.19045415	-2.13442213	0.00000000
N	1.20478284	-2.98920948	0.00000000
N	4.22417048	-3.72916107	0.00000000
H	4.87188462	0.61657584	0.00000000
H	0.00000000	0.00000000	0.00000000
H	-0.85839635	-2.40053065	0.00000000
H	3.59519175	-4.52125255	0.00000000
H	5.24043734	-3.87884063	0.00000000
N	7.43589774	-1.76641842	0.00000000
C	8.18624442	-0.60751724	0.00000000
N	9.55457326	-0.82540511	0.00000000
C	10.12356185	-2.07792616	0.00000000
C	9.37573473	-3.21473940	0.00000000
C	7.92740993	-3.07260278	0.00000000
O	7.12840447	-4.03783382	0.00000000
O	7.68622830	0.52828256	0.00000000
C	9.97823192	-4.59346043	0.00000000
H	6.39018301	-1.64159743	0.00000000
H	10.14543281	-0.00000000	0.00000000
H	11.20847888	-2.09044112	0.00000000
H	9.65659441	-5.16119078	0.88203001
H	11.07093646	-4.53812560	0.00000000
H	9.65659441	-5.16119078	-0.88203001

Yi–Px//A–T

Total bonding energy = -10333.72 kcal mol^{-1}

H	0.37840100	-1.70419558	3.40000000
H	6.45021521	0.62856237	3.40000000
H	2.78809506	-2.46784329	3.40000000
N	1.29115241	3.48530905	3.40000000
C	1.10079044	4.85295195	3.40000000
C	2.31766591	5.50623675	3.40000000
N	3.27045257	4.51018545	3.40000000
C	2.62034531	3.29095314	3.40000000
H	5.31676422	2.81387499	3.40000000
H	0.35993782	1.01447130	3.40000000
H	0.11084171	5.29092496	3.40000000
H	2.57721276	6.55346819	3.40000000
C	4.70997713	1.91407377	3.40000000
C	5.36215195	0.67342303	3.40000000
N	4.72402458	-0.51696256	3.40000000
C	3.38802282	-0.42541252	3.40000000
C	2.59927242	0.77597566	3.40000000

C	3.30432760	2.00511248	3.40000000
N	2.51350983	-1.49244038	3.40000000
C	1.21159667	-1.01480254	3.40000000
C	1.22311787	0.36656629	3.40000000
C	9.72206930	5.04305861	3.40000000
C	8.34987628	4.68863915	3.40000000
C	8.29244192	3.27157103	3.40000000
C	9.61003993	2.81725340	3.40000000
N	10.45954549	3.90696227	3.40000000
O	9.28870873	0.55161074	3.40000000
H	7.41431274	2.64938288	3.40000000
H	11.47231890	3.84161465	3.40000000
H	10.18467485	6.01912527	3.40000000
N	10.11057304	1.50701680	3.40000000
O	11.36485965	1.34406352	3.40000000
C	7.26948685	5.60574204	3.40000000
C	6.38929497	6.44346942	3.40000000
C	5.36614920	7.48433427	3.40000000
H	4.72853291	7.41578958	2.50972866
H	4.27472983	4.66069542	3.40000000
H	4.72853291	7.41578958	4.29027134
H	5.83102405	8.47755273	3.40000000
N	4.59205859	1.42571600	-0.00000000
C	4.11391081	0.16545978	-0.00000000
N	2.83043894	-0.23138786	-0.00000000
C	1.98254939	0.82089357	-0.00000000
C	2.34099925	2.18045605	-0.00000000
C	3.72507607	2.47909199	-0.00000000
N	0.60205504	0.81683069	-0.00000000
C	0.19045415	2.13442213	-0.00000000
N	1.20478284	2.98920948	-0.00000000
N	4.22417048	3.72916107	-0.00000000
H	4.87188462	-0.61657584	-0.00000000
H	0.00000000	-0.00000000	-0.00000000
H	-0.85839635	2.40053065	-0.00000000
H	3.59519175	4.52125255	-0.00000000
H	5.24043734	3.87884063	-0.00000000
N	7.43589774	1.76641842	-0.00000000
C	8.18624442	0.60751724	-0.00000000
N	9.55457326	0.82540511	-0.00000000
C	10.12356185	2.07792616	-0.00000000
C	9.37573473	3.21473940	-0.00000000
C	7.92740993	3.07260278	-0.00000000
O	7.12840447	4.03783382	-0.00000000
O	7.68622830	-0.52828256	-0.00000000
C	9.97823192	4.59346043	-0.00000000
H	6.39018301	1.64159743	-0.00000000
H	10.14543281	0.00000000	-0.00000000
H	11.20847888	2.09044112	-0.00000000
H	9.65659441	5.16119078	-0.88203001
H	11.07093646	4.53812560	-0.00000000
H	9.65659441	5.16119078	0.88203001

Do–Px//A–T

Total bonding energy = -10205.53 kcal mol^{-1}

H	0.83172790	-1.62048722	3.40000000
H	7.31157005	-1.47061509	3.40000000
H	2.77851671	-3.26137032	3.40000000
C	3.29961009	2.95851830	3.40000000
C	3.68693291	4.33654550	3.40000000
C	5.05482761	4.35859748	3.40000000
O	5.55272907	3.07438349	3.40000000
C	4.45692783	2.20936630	3.40000000
H	6.89852799	0.94074628	3.40000000
H	2.29814538	2.55334276	3.40000000
H	3.03275719	5.19862856	3.40000000
H	5.79757907	5.14350356	3.40000000
C	6.04576234	0.27202634	3.40000000
C	6.28545215	-1.10838199	3.40000000
N	5.32033397	-2.05838251	3.40000000
C	4.08197629	-1.55275303	3.40000000
C	3.70550560	-0.18135939	3.40000000
C	4.73169959	0.79134659	3.40000000
N	2.88475211	-2.25240588	3.40000000
C	1.87282147	-1.32418974	3.40000000
N	2.31436344	-0.07120090	3.40000000
C	10.70604175	5.09723538	3.40000000
C	9.29142167	5.02050039	3.40000000
C	8.95051582	3.64168880	3.40000000
C	10.15495546	2.93707626	3.40000000
N	11.20363978	3.83829096	3.40000000
O	9.41411151	0.77008087	3.40000000
H	7.95938093	3.21427938	3.40000000
H	12.18261481	3.57110689	3.40000000
H	11.35183752	5.96316829	3.40000000
N	10.39895398	1.55620280	3.40000000
O	11.60130650	1.15996320	3.40000000
C	8.40254157	6.12363907	3.40000000
C	7.63136441	7.06202015	3.40000000
C	6.69249613	8.17852968	3.40000000
H	6.04747260	8.15167408	2.51220999
H	6.04747260	8.15167408	4.28779001
H	7.22502077	9.13748215	3.40000000
N	4.59205859	1.42571600	-0.00000000
C	4.11391081	0.16545978	-0.00000000
N	2.83043894	-0.23138786	-0.00000000
C	1.98254939	0.82089357	-0.00000000
C	2.34099925	2.18045605	-0.00000000
C	3.72507607	2.47909199	-0.00000000
N	0.60205504	0.81683069	-0.00000000
C	0.19045415	2.13442213	-0.00000000
N	1.20478284	2.98920948	-0.00000000
N	4.22417048	3.72916107	-0.00000000
H	4.87188462	-0.61657584	-0.00000000
H	0.00000000	-0.00000000	-0.00000000

H	-0.85839635	2.40053065	-0.00000000
H	3.59519175	4.52125255	-0.00000000
H	5.24043734	3.87884063	-0.00000000
N	7.43589774	1.76641842	-0.00000000
C	8.18624442	0.60751724	-0.00000000
N	9.55457326	0.82540511	-0.00000000
C	10.12356185	2.07792616	-0.00000000
C	9.37573473	3.21473940	-0.00000000
C	7.92740993	3.07260278	-0.00000000
O	7.12840447	4.03783382	-0.00000000
O	7.68622830	-0.52828256	-0.00000000
C	9.97823192	4.59346043	-0.00000000
H	6.39018301	1.64159743	-0.00000000
H	10.14543281	0.00000000	-0.00000000
H	11.20847888	2.09044112	-0.00000000
H	9.65659441	5.16119078	-0.88203001
H	11.07093646	4.53812560	-0.00000000
H	9.65659441	5.16119078	0.88203001

Dp–Px//A–T

Total bonding energy = -10407.51 kcal mol^{-1}

H	0.90299203	-1.59974755	3.40000000
H	7.37774878	-1.40859283	3.40000000
H	2.86521489	-3.22135091	3.40000000
C	4.55450798	2.31120675	3.40000000
N	5.66457554	3.08772553	3.40000000
H	6.30360193	8.40064742	4.28716044
H	6.30360193	8.40064742	2.51283956
N	5.56383070	4.42522526	3.40000000
H	6.94110205	1.01196779	3.40000000
C	4.34846871	4.99676957	3.40000000
C	3.16098817	4.25799016	3.40000000
C	3.26253093	2.87746250	3.40000000
C	6.10069119	0.32931707	3.40000000
C	6.35104254	-1.04854386	3.40000000
N	5.38737788	-1.99911170	3.40000000
C	4.15035276	-1.50090987	3.40000000
C	3.76405434	-0.12542821	3.40000000
C	4.78952071	0.84631684	3.40000000
N	2.96087707	-2.21128838	3.40000000
C	1.94145145	-1.29439083	3.40000000
N	2.36884908	-0.03737438	3.40000000
C	10.86828814	5.20524071	3.40000000
C	9.45150753	5.18479713	3.40000000
C	9.05312721	3.81977829	3.40000000
C	10.23154412	3.06901810	3.40000000
N	11.31626402	3.92832362	3.40000000
O	9.42731241	0.92147098	3.40000000
H	8.04097706	3.43622229	3.40000000
H	12.28337796	3.62134510	3.40000000
H	11.54821675	6.04486557	3.40000000
N	10.43245501	1.68196800	3.40000000

O	11.62400283	1.24938123	3.40000000
C	8.61170476	6.32572278	3.40000000
C	7.86948997	7.28624629	3.40000000
C	6.95012538	8.41881703	3.40000000
H	2.19609066	4.75729378	3.40000000
H	2.39062486	2.23423583	3.40000000
H	4.34723337	6.08458121	3.40000000
H	7.49479143	9.37119328	3.40000000
N	4.59205859	1.42571600	-0.00000000
C	4.11391081	0.16545978	-0.00000000
N	2.83043894	-0.23138786	-0.00000000
C	1.98254939	0.82089357	-0.00000000
C	2.34099925	2.18045605	-0.00000000
C	3.72507607	2.47909199	-0.00000000
N	0.60205504	0.81683069	-0.00000000
C	0.19045415	2.13442213	-0.00000000
N	1.20478284	2.98920948	-0.00000000
N	4.22417048	3.72916107	-0.00000000
H	4.87188462	-0.61657584	-0.00000000
H	0.00000000	-0.00000000	-0.00000000
H	-0.85839635	2.40053065	-0.00000000
H	3.59519175	4.52125255	-0.00000000
H	5.24043734	3.87884063	-0.00000000
N	7.43589774	1.76641842	-0.00000000
C	8.18624442	0.60751724	-0.00000000
N	9.55457326	0.82540511	-0.00000000
C	10.12356185	2.07792616	-0.00000000
C	9.37573473	3.21473940	-0.00000000
C	7.92740993	3.07260278	-0.00000000
O	7.12840447	4.03783382	-0.00000000
O	7.68622830	-0.52828256	-0.00000000
C	9.97823192	4.59346043	-0.00000000
H	6.39018301	1.64159743	-0.00000000
H	10.14543281	0.00000000	-0.00000000
H	11.20847888	2.09044112	-0.00000000
H	9.65659441	5.16119078	-0.88203001
H	11.07093646	4.53812560	-0.00000000
H	9.65659441	5.16119078	0.88203001

Dss–Px//A–T

Total bonding energy = -11180.55 kcal mol^{-1}

H	1.42285784	1.88356517	-3.40000000
H	7.79918240	0.71937179	-3.40000000
H	3.60574795	3.19583530	-3.40000000
C	3.11127386	-2.95657100	-3.40000000
C	3.00707564	-4.36506606	-3.40000000
C	4.23387603	-5.01890526	-3.40000000
S	5.54669649	-3.85067777	-3.40000000
C	4.41943413	-2.48919969	-3.40000000
H	7.01890974	-1.58727435	-3.40000000
H	2.26492312	-2.28053896	-3.40000000
H	2.05985877	-4.89557720	-3.40000000

H	3.86255718	-9.94588175	-3.40000000
C	6.27441968	-0.79827797	-3.40000000
C	6.72897184	0.52499262	-3.40000000
N	5.92253006	1.61355348	-3.40000000
C	4.62161242	1.30287205	-3.40000000
C	4.03468819	0.00565956	-3.40000000
C	4.89692225	-1.11981379	-3.40000000
N	3.55006499	2.18284221	-3.40000000
C	2.40430507	1.42711052	-3.40000000
N	2.64279887	0.12063623	-3.40000000
C	11.87341764	-5.40784950	-3.40000000
C	10.49393957	-5.73312686	-3.40000000
C	9.77961271	-4.50576364	-3.40000000
C	10.73844247	-3.49047379	-3.40000000
N	11.99718788	-4.06055471	-3.40000000
O	9.42741384	-1.61339239	-3.40000000
H	8.70891649	-4.36665584	-3.40000000
H	12.86171138	-3.52901577	-3.40000000
H	12.73586185	-6.05819916	-3.40000000
N	10.59001380	-2.09596905	-3.40000000
O	11.63642095	-1.38290800	-3.40000000
C	9.97659897	-7.05191035	-3.40000000
C	9.59473744	-8.20479354	-3.40000000
C	9.16273432	-9.59799320	-3.40000000
C	4.49724805	-6.43729192	-3.40000000
C	5.71125076	-7.11128205	-3.40000000
C	5.57041856	-8.52728162	-3.40000000
C	4.25678803	-8.93770959	-3.40000000
S	3.16263368	-7.59086571	-3.40000000
H	8.56228981	-9.82789541	-4.28922480
H	8.56228981	-9.82789541	-2.51077520
H	6.67187969	-6.60540115	-3.40000000
H	6.40394743	-9.21833856	-3.40000000
H	10.02968500	-10.27019328	-3.40000000
N	4.59205859	-1.42571600	0.00000000
C	4.11391081	-0.16545978	0.00000000
N	2.83043894	0.23138786	0.00000000
C	1.98254939	-0.82089357	0.00000000
C	2.34099925	-2.18045605	0.00000000
C	3.72507607	-2.47909199	0.00000000
N	0.60205504	-0.81683069	0.00000000
C	0.19045415	-2.13442213	0.00000000
N	1.20478284	-2.98920948	0.00000000
N	4.22417048	-3.72916107	0.00000000
H	4.87188462	0.61657584	0.00000000
H	0.00000000	0.00000000	0.00000000
H	-0.85839635	-2.40053065	0.00000000
H	3.59519175	-4.52125255	0.00000000
H	5.24043734	-3.87884063	0.00000000
N	7.43589774	-1.76641842	0.00000000
C	8.18624442	-0.60751724	0.00000000
N	9.55457326	-0.82540511	0.00000000

C	10.12356185	-2.07792616	0.00000000
C	9.37573473	-3.21473940	0.00000000
C	7.92740993	-3.07260278	0.00000000
O	7.12840447	-4.03783382	0.00000000
O	7.68622830	0.52828256	0.00000000
C	9.97823192	-4.59346043	0.00000000
H	6.39018301	-1.64159743	0.00000000
H	10.14543281	-0.00000000	0.00000000
H	11.20847888	-2.09044112	0.00000000
H	9.65659441	-5.16119078	0.88203001
H	11.07093646	-4.53812560	0.00000000
H	9.65659441	-5.16119078	-0.88203001

Dt–Px//A–T

Total bonding energy = -10046.22 kcal mol^{-1}

H	0.69179222	1.69140735	-3.40000000
H	7.11268171	0.82777605	-3.40000000
H	2.81383403	3.10026961	-3.40000000
N	2.67048956	-3.01415065	-3.40000000
C	2.63844411	-4.38940793	-3.40000000
C	3.86174670	-5.01470496	-3.40000000
S	5.13013964	-3.84498496	-3.40000000
C	3.90899368	-2.55330804	-3.40000000
H	6.44023662	-1.51330134	-3.40000000
H	5.71171532	-7.87750018	-4.28881855
H	1.68249435	-4.90306447	-3.40000000
H	4.07474082	-6.07473789	-3.40000000
C	5.65904570	-0.76093138	-3.40000000
C	6.05367331	0.58100410	-3.40000000
N	5.19227437	1.62445415	-3.40000000
C	3.90988496	1.25503920	-3.40000000
C	3.37888636	-0.07223071	-3.40000000
C	4.30058077	-1.14523825	-3.40000000
N	2.80233325	2.08571800	-3.40000000
C	1.69259339	1.27885037	-3.40000000
N	1.98585656	-0.01649148	-3.40000000
C	10.54602627	-5.09349986	-3.40000000
C	9.13703828	-4.94385259	-3.40000000
C	8.87255732	-3.54990713	-3.40000000
C	10.10919314	-2.90697815	-3.40000000
N	11.10931726	-3.86120190	-3.40000000
O	9.45947272	-0.71286788	-3.40000000
H	7.90852253	-3.07077926	-3.40000000
H	12.10134912	-3.64750509	-3.40000000
H	11.14666839	-5.99130575	-3.40000000
N	10.41236133	-1.53788282	-3.40000000
O	11.62971003	-1.19275576	-3.40000000
C	8.18454166	-5.99226318	-3.40000000
C	7.35309274	-6.87728318	-3.40000000
C	6.35291325	-7.93884225	-3.40000000
H	5.71171532	-7.87750018	-2.51118145
H	6.82951428	-8.92673127	-3.40000000

N	4.59205859	-1.42571600	0.00000000
C	4.11391081	-0.16545978	0.00000000
N	2.83043894	0.23138786	0.00000000
C	1.98254939	-0.82089357	0.00000000
C	2.34099925	-2.18045605	0.00000000
C	3.72507607	-2.47909199	0.00000000
N	0.60205504	-0.81683069	0.00000000
C	0.19045415	-2.13442213	0.00000000
N	1.20478284	-2.98920948	0.00000000
N	4.22417048	-3.72916107	0.00000000
H	4.87188462	0.61657584	0.00000000
H	0.00000000	0.00000000	0.00000000
H	-0.85839635	-2.40053065	0.00000000
H	3.59519175	-4.52125255	0.00000000
H	5.24043734	-3.87884063	0.00000000
N	7.43589774	-1.76641842	0.00000000
C	8.18624442	-0.60751724	0.00000000
N	9.55457326	-0.82540511	0.00000000
C	10.12356185	-2.07792616	0.00000000
C	9.37573473	-3.21473940	0.00000000
C	7.92740993	-3.07260278	0.00000000
O	7.12840447	-4.03783382	0.00000000
O	7.68622830	0.52828256	0.00000000
C	9.97823192	-4.59346043	0.00000000
H	6.39018301	-1.64159743	0.00000000
H	10.14543281	-0.00000000	0.00000000
H	11.20847888	-2.09044112	0.00000000
H	9.65659441	-5.16119078	0.88203001
H	11.07093646	-4.53812560	0.00000000
H	9.65659441	-5.16119078	-0.88203001

Yo–Px/A–T

Total bonding energy = -10302.77 kcal mol^{-1}

H	0.73481030	-1.69826134	3.40000000
H	7.23106721	-1.32836962	3.40000000
H	2.79857361	-3.16005370	3.40000000
C	3.29191973	3.16471482	3.40000000
C	3.72712808	4.52881969	3.40000000
C	5.09370591	4.50227429	3.40000000
O	5.54350639	3.20051503	3.40000000
C	4.41954333	2.37021807	3.40000000
H	6.82825093	1.08861187	3.40000000
H	2.26775472	2.82448877	3.40000000
H	3.10203786	5.41214400	3.40000000
H	5.86350723	5.26076117	3.40000000
C	5.97330017	0.42283437	3.40000000
C	6.20739902	-0.95828767	3.40000000
N	5.23492374	-1.89643988	3.40000000
C	3.99192421	-1.39675703	3.40000000
C	3.60961214	-0.01242376	3.40000000
C	4.66123318	0.94188892	3.40000000
N	2.83284288	-2.14732641	3.40000000

C	1.73952973	-1.29859301	3.40000000
C	2.17233392	0.01380385	3.40000000
C	10.71770847	5.19751909	3.40000000
C	9.30220551	5.14083224	3.40000000
C	8.94182294	3.76688834	3.40000000
C	10.13608064	3.04549987	3.40000000
N	11.19754096	3.93157906	3.40000000
O	9.36163707	0.89079849	3.40000000
H	7.94549388	3.35161809	3.40000000
H	12.17265353	3.65061402	3.40000000
H	11.37561885	6.05429235	3.40000000
N	10.35872270	1.66105467	3.40000000
O	11.55493021	1.24638627	3.40000000
C	8.42863781	6.25612586	3.40000000
C	7.66816382	7.20299234	3.40000000
C	6.74106138	8.32927145	3.40000000
H	6.09575171	8.30902424	4.28776195
H	6.09575171	8.30902424	2.51223805
H	1.51992512	0.87420558	3.40000000
H	7.28365717	9.28258100	3.40000000
N	4.59205859	1.42571600	-0.00000000
C	4.11391081	0.16545978	-0.00000000
N	2.83043894	-0.23138786	-0.00000000
C	1.98254939	0.82089357	-0.00000000
C	2.34099925	2.18045605	-0.00000000
C	3.72507607	2.47909199	-0.00000000
N	0.60205504	0.81683069	-0.00000000
C	0.19045415	2.13442213	-0.00000000
N	1.20478284	2.98920948	-0.00000000
N	4.22417048	3.72916107	-0.00000000
H	4.87188462	-0.61657584	-0.00000000
H	0.00000000	-0.00000000	-0.00000000
H	-0.85839635	2.40053065	-0.00000000
H	3.59519175	4.52125255	-0.00000000
H	5.24043734	3.87884063	-0.00000000
N	7.43589774	1.76641842	-0.00000000
C	8.18624442	0.60751724	-0.00000000
N	9.55457326	0.82540511	-0.00000000
C	10.12356185	2.07792616	-0.00000000
C	9.37573473	3.21473940	-0.00000000
C	7.92740993	3.07260278	-0.00000000
O	7.12840447	4.03783382	-0.00000000
O	7.68622830	-0.52828256	-0.00000000
C	9.97823192	4.59346043	-0.00000000
H	6.39018301	1.64159743	-0.00000000
H	10.14543281	0.00000000	-0.00000000
H	11.20847888	2.09044112	-0.00000000
H	9.65659441	5.16119078	-0.88203001
H	11.07093646	4.53812560	-0.00000000
H	9.65659441	5.16119078	0.88203001

Ps–Px//A–T

Total bonding energy = -10048.11 kcal mol^{-1}

H	0.58954481	1.58496551	-3.40000000
H	6.80341692	-0.20095692	-3.40000000
H	2.87130661	2.71947295	-3.40000000
C	2.00562231	-3.44967829	-3.40000000
C	1.90361809	-4.86668246	-3.40000000
C	3.13570315	-5.48755763	-3.40000000
S	4.44502529	-4.35320212	-3.40000000
C	3.31994943	-3.00273638	-3.40000000
H	5.31371529	-7.94358011	-4.28850893
H	1.16115404	-2.77043928	-3.40000000
H	0.96247232	-5.40779957	-3.40000000
H	3.34339014	-6.54981852	-3.40000000
N	5.19331924	-1.49249872	-3.40000000
C	5.71781925	-0.25564618	-3.40000000
N	5.05445633	0.92131285	-3.40000000
C	3.72607096	0.75303214	-3.40000000
C	3.03943753	-0.48933604	-3.40000000
C	3.83833206	-1.65119857	-3.40000000
N	2.73672421	1.71359912	-3.40000000
C	1.52940818	1.04832093	-3.40000000
N	1.66050201	-0.27106727	-3.40000000
C	10.03850755	-5.05421463	-3.40000000
C	8.63189918	-4.88217958	-3.40000000
C	8.38217038	-3.48244586	-3.40000000
C	9.63570504	-2.86282388	-3.40000000
N	10.62032762	-3.83358401	-3.40000000
O	9.07673555	-0.63914655	-3.40000000
H	7.42099634	-2.98467416	-3.40000000
H	11.61516228	-3.63331005	-3.40000000
H	10.62309834	-5.96260024	-3.40000000
N	9.99079866	-1.50654766	-3.40000000
O	11.22200447	-1.21022976	-3.40000000
C	7.69178477	-5.94190830	-3.40000000
C	6.90813272	-6.86930543	-3.40000000
C	5.95745840	-7.97534157	-3.40000000
H	5.31371529	-7.94358011	-2.51149107
H	6.47871464	-8.94036610	-3.40000000
N	4.59205859	-1.42571600	0.00000000
C	4.11391081	-0.16545978	0.00000000
N	2.83043894	0.23138786	0.00000000
C	1.98254939	-0.82089357	0.00000000
C	2.34099925	-2.18045605	0.00000000
C	3.72507607	-2.47909199	0.00000000
N	0.60205504	-0.81683069	0.00000000
C	0.19045415	-2.13442213	0.00000000
N	1.20478284	-2.98920948	0.00000000
N	4.22417048	-3.72916107	0.00000000
H	4.87188462	0.61657584	0.00000000
H	0.00000000	0.00000000	0.00000000
H	-0.85839635	-2.40053065	0.00000000

H	3.59519175	-4.52125255	0.00000000
H	5.24043734	-3.87884063	0.00000000
N	7.43589774	-1.76641842	0.00000000
C	8.18624442	-0.60751724	0.00000000
N	9.55457326	-0.82540511	0.00000000
C	10.12356185	-2.07792616	0.00000000
C	9.37573473	-3.21473940	0.00000000
C	7.92740993	-3.07260278	0.00000000
O	7.12840447	-4.03783382	0.00000000
O	7.68622830	0.52828256	0.00000000
C	9.97823192	-4.59346043	0.00000000
H	6.39018301	-1.64159743	0.00000000
H	10.14543281	-0.00000000	0.00000000
H	11.20847888	-2.09044112	0.00000000
H	9.65659441	-5.16119078	0.88203001
H	11.07093646	-4.53812560	0.00000000
H	9.65659441	-5.16119078	-0.88203001

Es–Px//A–T

Total bonding energy = -10146.25 kcal mol^{-1}

H	0.53120489	1.67307573	-3.40000000
H	6.71628395	-0.30621809	-3.40000000
H	2.87829663	2.61462531	-3.40000000
C	1.99178517	-3.65743559	-3.40000000
C	1.95200508	-5.07868366	-3.40000000
C	3.20918737	-5.64387131	-3.40000000
S	4.46493399	-4.45035690	-3.40000000
C	3.28210578	-3.14713456	-3.40000000
H	0.69233741	-1.01240070	-3.40000000
H	1.10191636	-3.04134598	-3.40000000
H	1.03416244	-5.65846219	-3.40000000
H	3.46310732	-6.69563439	-3.40000000
N	5.11642730	-1.61364653	-3.40000000
C	5.63134872	-0.37229345	-3.40000000
N	4.95304734	0.79209972	-3.40000000
C	3.62269057	0.62534654	-3.40000000
C	2.93207883	-0.63197868	-3.40000000
C	3.76337389	-1.77628377	-3.40000000
N	2.67840807	1.62082413	-3.40000000
C	1.41284560	1.04751836	-3.40000000
C	1.52641434	-0.32704078	-3.40000000
C	10.01315377	-5.14104939	-3.40000000
C	8.60444721	-4.98540676	-3.40000000
C	8.33859745	-3.58872058	-3.40000000
C	9.58507431	-2.95470516	-3.40000000
N	10.58083438	-3.91390278	-3.40000000
O	9.00039533	-0.73785511	-3.40000000
H	7.37304486	-3.09968793	-3.40000000
H	11.57338979	-3.70272965	-3.40000000
H	10.60809812	-6.04271325	-3.40000000
N	9.92448183	-1.59441288	-3.40000000
O	11.15234071	-1.28385645	-3.40000000

C	7.68016989	-6.05928747	-3.40000000
C	6.91995663	-7.00607303	-3.40000000
C	6.00249773	-8.13973435	-3.40000000
H	5.35823661	-8.12786901	-2.51139688
H	5.35823661	-8.12786901	-4.28860312
H	6.55351609	-9.08818618	-3.40000000
N	4.59205859	-1.42571600	0.00000000
C	4.11391081	-0.16545978	0.00000000
N	2.83043894	0.23138786	0.00000000
C	1.98254939	-0.82089357	0.00000000
C	2.34099925	-2.18045605	0.00000000
C	3.72507607	-2.47909199	0.00000000
N	0.60205504	-0.81683069	0.00000000
C	0.19045415	-2.13442213	0.00000000
N	1.20478284	-2.98920948	0.00000000
N	4.22417048	-3.72916107	0.00000000
H	4.87188462	0.61657584	0.00000000
H	0.00000000	0.00000000	0.00000000
H	-0.85839635	-2.40053065	0.00000000
H	3.59519175	-4.52125255	0.00000000
H	5.24043734	-3.87884063	0.00000000
N	7.43589774	-1.76641842	0.00000000
C	8.18624442	-0.60751724	0.00000000
N	9.55457326	-0.82540511	0.00000000
C	10.12356185	-2.07792616	0.00000000
C	9.37573473	-3.21473940	0.00000000
C	7.92740993	-3.07260278	0.00000000
O	7.12840447	-4.03783382	0.00000000
O	7.68622830	0.52828256	0.00000000
C	9.97823192	-4.59346043	0.00000000
H	6.39018301	-1.64159743	0.00000000
H	10.14543281	-0.00000000	0.00000000
H	11.20847888	-2.09044112	0.00000000
H	9.65659441	-5.16119078	0.88203001
H	11.07093646	-4.53812560	0.00000000
H	9.65659441	-5.16119078	-0.88203001

Ysm–Px//A–T

Total bonding energy = -10614.32 kcal mol^{-1}

H	0.94119192	-1.81316196	3.40000000
H	7.36096185	-0.76144552	3.40000000
H	3.15093817	-3.04484173	3.40000000
C	2.94929441	3.25248283	3.40000000
C	2.99332736	4.67508416	3.40000000
C	4.27000436	5.19327996	3.40000000
S	5.44655135	3.90243178	3.40000000
C	4.19685025	2.64830383	3.40000000
H	6.71308737	1.58759914	3.40000000
H	2.01998613	2.69970908	3.40000000
H	2.10776626	5.30450018	3.40000000
C	4.70153215	6.62936108	3.40000000
C	5.92309314	0.84327935	3.40000000

C	6.30394207	-0.50301989	3.40000000
N	5.43356911	-1.53629993	3.40000000
C	4.14687162	-1.16416340	3.40000000
C	3.62051583	0.17475145	3.40000000
C	4.56681961	1.23807232	3.40000000
N	3.07511149	-2.03438941	3.40000000
C	1.89755248	-1.30862144	3.40000000
C	2.18725106	0.04204757	3.40000000
C	11.16343816	5.40058237	3.40000000
C	9.74866040	5.48043521	3.40000000
C	9.26038237	4.14757862	3.40000000
C	10.37864999	3.31387104	3.40000000
N	11.51999256	4.09400579	3.40000000
O	9.39555923	1.24607915	3.40000000
H	8.22948033	3.83210907	3.40000000
H	12.46390031	3.72142132	3.40000000
H	11.90082875	6.18990699	3.40000000
N	10.46387418	1.91427092	3.40000000
O	11.61341146	1.38371442	3.40000000
C	8.98645101	6.67474257	3.40000000
C	8.33651976	7.70064656	3.40000000
C	7.56152733	8.93633554	3.40000000
H	6.91862765	9.00033143	2.51301604
H	5.30598708	6.86746666	4.28354978
H	3.82073112	7.27919755	3.40000000
H	5.30598708	6.86746666	2.51645022
H	1.44023094	0.82075669	3.40000000
H	6.91862765	9.00033143	4.28698396
H	8.22286077	9.81151535	3.40000000
N	4.59205859	1.42571600	-0.00000000
C	4.11391081	0.16545978	-0.00000000
N	2.83043894	-0.23138786	-0.00000000
C	1.98254939	0.82089357	-0.00000000
C	2.34099925	2.18045605	-0.00000000
C	3.72507607	2.47909199	-0.00000000
N	0.60205504	0.81683069	-0.00000000
C	0.19045415	2.13442213	-0.00000000
N	1.20478284	2.98920948	-0.00000000
N	4.22417048	3.72916107	-0.00000000
H	4.87188462	-0.61657584	-0.00000000
H	0.00000000	-0.00000000	-0.00000000
H	-0.85839635	2.40053065	-0.00000000
H	3.59519175	4.52125255	-0.00000000
H	5.24043734	3.87884063	-0.00000000
N	7.43589774	1.76641842	-0.00000000
C	8.18624442	0.60751724	-0.00000000
N	9.55457326	0.82540511	-0.00000000
C	10.12356185	2.07792616	-0.00000000
C	9.37573473	3.21473940	-0.00000000
C	7.92740993	3.07260278	-0.00000000
O	7.12840447	4.03783382	-0.00000000
O	7.68622830	-0.52828256	-0.00000000

C	9.97823192	4.59346043	-0.00000000
H	6.39018301	1.64159743	-0.00000000
H	10.14543281	0.00000000	-0.00000000
H	11.20847888	2.09044112	-0.00000000
H	9.65659441	5.16119078	-0.88203001
H	11.07093646	4.53812560	-0.00000000
H	9.65659441	5.16119078	0.88203001

Yss–Px//A–T

Total bonding energy = -11276.90 kcal mol^{-1}

H	1.32559022	2.01044801	-3.40000000
H	7.66332862	0.54117427	-3.40000000
H	3.61036189	3.09467798	-3.40000000
C	3.00208530	-3.17130275	-3.40000000
C	2.94390124	-4.58367099	-3.40000000
C	4.18999513	-5.19572099	-3.40000000
S	5.46067430	-3.98245591	-3.40000000
C	4.29027684	-2.65282732	-3.40000000
H	6.86375955	-1.76000047	-3.40000000
H	2.11370726	-2.55546946	-3.40000000
H	2.01308622	-5.14238977	-3.40000000
C	4.50279894	-6.60431385	-3.40000000
C	6.12509924	-0.96497619	-3.40000000
C	6.59186815	0.35336786	-3.40000000
N	5.78959586	1.44077551	-3.40000000
C	4.48162546	1.15284884	-3.40000000
C	3.86867012	-0.14900193	-3.40000000
C	4.74619159	-1.27085833	-3.40000000
N	3.46905623	2.09126790	-3.40000000
C	2.24684415	1.44441218	-3.40000000
C	2.44762028	0.07736552	-3.40000000
C	11.85178223	-5.47803345	-3.40000000
C	10.48249604	-5.84320913	-3.40000000
C	9.73291661	-4.63685233	-3.40000000
C	10.66197075	-3.59429607	-3.40000000
N	11.93662407	-4.12770084	-3.40000000
O	9.29720223	-1.75606208	-3.40000000
H	8.65870675	-4.52769411	-3.40000000
H	12.78542362	-3.57139457	-3.40000000
H	12.73274979	-6.10310332	-3.40000000
N	10.47303582	-2.20445692	-3.40000000
O	11.49858668	-1.46150713	-3.40000000
C	10.00508299	-7.17704959	-3.40000000
C	9.65996633	-8.34149880	-3.40000000
C	9.27309922	-9.74799516	-3.40000000
C	5.73968960	-7.23448444	-3.40000000
C	5.65003042	-8.65504877	-3.40000000
C	4.35225079	-9.11240733	-3.40000000
S	3.21049309	-7.80503256	-3.40000000
H	6.68211120	-6.69554799	-3.40000000
H	8.67824982	-9.99782377	-4.28777903
H	8.67824982	-9.99782377	-2.51222097

H	6.50924419	-9.31592678	-3.40000000
H	3.99354236	-10.13377430	-3.40000000
H	1.65037386	-0.64986456	-3.40000000
H	10.16041638	-10.39284981	-3.40000000
N	4.59205859	-1.42571600	0.00000000
C	4.11391081	-0.16545978	0.00000000
N	2.83043894	0.23138786	0.00000000
C	1.98254939	-0.82089357	0.00000000
C	2.34099925	-2.18045605	0.00000000
C	3.72507607	-2.47909199	0.00000000
N	0.60205504	-0.81683069	0.00000000
C	0.19045415	-2.13442213	0.00000000
N	1.20478284	-2.98920948	0.00000000
N	4.22417048	-3.72916107	0.00000000
H	4.87188462	0.61657584	0.00000000
H	0.00000000	0.00000000	0.00000000
H	-0.85839635	-2.40053065	0.00000000
H	3.59519175	-4.52125255	0.00000000
H	5.24043734	-3.87884063	0.00000000
N	7.43589774	-1.76641842	0.00000000
C	8.18624442	-0.60751724	0.00000000
N	9.55457326	-0.82540511	0.00000000
C	10.12356185	-2.07792616	0.00000000
C	9.37573473	-3.21473940	0.00000000
C	7.92740993	-3.07260278	0.00000000
O	7.12840447	-4.03783382	0.00000000
O	7.68622830	0.52828256	0.00000000
C	9.97823192	-4.59346043	0.00000000
H	6.39018301	-1.64159743	0.00000000
H	10.14543281	-0.00000000	0.00000000
H	11.20847888	-2.09044112	0.00000000
H	9.65659441	-5.16119078	0.88203001
H	11.07093646	-4.53812560	0.00000000
H	9.65659441	-5.16119078	-0.88203001

Cartesian Coordinates and Total Bonding Energies of Ds–Px//G–C variants in Methanol

Cartesian coordinates (Å) and total bonding energies (kcal mol^{-1}) of Ds–Px//G–C variants optimized at ZORA-BLYP-D3(BJ)-TZ2P level of theory in methanol.

Ds–Px//G–C

Total bonding energy = -10058.49 kcal mol^{-1}

H	0.72912235	-1.61047166	3.40000000
H	7.15897015	-0.80152929	3.40000000
H	2.83659061	-3.04146055	3.40000000
C	2.67511608	3.13094604	3.40000000
C	2.65529017	4.55301823	3.40000000
C	3.91661045	5.10572746	3.40000000
S	5.15117212	3.89199322	3.40000000
C	3.95576314	2.59457389	3.40000000
H	6.50640341	1.54229137	3.40000000
H	1.79195212	2.50385803	3.40000000
H	1.74635043	5.14667825	3.40000000
H	4.19263332	6.15171775	3.40000000
C	5.71768406	0.79746966	3.40000000
C	6.10108310	-0.54856727	3.40000000
N	5.23570647	-1.58972806	3.40000000
C	3.95455175	-1.20785811	3.40000000
C	3.44017875	0.12070350	3.40000000
C	4.36131310	1.19639860	3.40000000
N	2.83628736	-2.02690388	3.40000000
C	1.73426235	-1.20873089	3.40000000
N	2.04385658	0.08246312	3.40000000
C	10.56861393	5.15578985	3.40000000
C	9.15906176	5.01551172	3.40000000
C	8.88490360	3.62288854	3.40000000
C	10.11746216	2.97234111	3.40000000
N	11.12394709	3.91962122	3.40000000
O	9.45415019	0.78221533	3.40000000
H	7.91701394	3.15100237	3.40000000
H	12.11450876	3.69934156	3.40000000
H	11.17572256	6.04927199	3.40000000
N	10.41214343	1.60108356	3.40000000
O	11.62730203	1.24830461	3.40000000
C	8.21405558	6.07087454	3.40000000
C	7.39357236	6.96614505	3.40000000
C	6.40918937	8.04256068	3.40000000
H	5.76695266	7.99112768	4.28868981
H	6.90117970	9.02288872	3.40000000
H	5.76695266	7.99112768	2.51131019
N	5.01072929	-0.99340199	0.00000000
O	4.30311303	3.58754463	0.00000000
N	4.56576767	1.29129485	0.00000000
C	4.09065743	-0.00627844	0.00000000
N	2.78482728	-0.30606844	0.00000000
C	1.98994643	0.78688802	0.00000000
C	2.37464339	2.13938648	0.00000000

C	3.76637283	2.44955519	0.00000000
N	0.61177148	0.80968027	0.00000000
C	0.22028517	2.13921842	0.00000000
N	1.25033840	2.96889971	0.00000000
H	6.02485359	-0.80644199	0.00000000
H	4.67541456	-1.94791441	0.00000000
H	5.60094570	1.44221118	0.00000000
H	0.00000000	0.00000000	0.00000000
H	-0.82386801	2.42227350	0.00000000
O	7.83019934	-0.55673593	0.00000000
N	7.14222402	4.00240580	0.00000000
N	7.45611902	1.71718501	0.00000000
C	9.41029341	3.17541626	0.00000000
C	8.26404832	0.62432069	0.00000000
N	9.65144142	0.82879605	0.00000000
C	10.20904031	2.07360344	0.00000000
C	7.99014840	2.96108357	0.00000000
H	9.82814656	4.17525163	0.00000000
H	10.23841777	0.00000000	0.00000000
H	11.29222369	2.11783835	0.00000000
H	6.12182623	3.85260423	0.00000000
H	7.50463415	4.94693884	0.00000000

Bs–Px//G–C

Total bonding energy = -10158.99 kcal mol^{-1}

H	0.69708056	1.57789630	-3.40000000
H	7.26700786	0.76968491	-3.40000000
H	2.74698332	3.07677467	-3.40000000
C	2.70485920	-3.07494384	-3.40000000
C	2.68154503	-4.49983465	-3.40000000
C	3.93845747	-5.05770432	-3.40000000
S	5.17632455	-3.84151209	-3.40000000
C	3.98401356	-2.53742169	-3.40000000
H	6.52162052	-1.54437664	-3.40000000
H	1.82285304	-2.44682710	-3.40000000
H	1.76969123	-5.08967870	-3.40000000
H	4.21303597	-6.10377050	-3.40000000
C	5.76322730	-0.76733730	-3.40000000
C	6.19927354	0.56878158	-3.40000000
C	5.29333020	1.63247407	-3.40000000
C	3.93961358	1.28663528	-3.40000000
C	3.47259224	-0.05982579	-3.40000000
C	4.40191217	-1.13631094	-3.40000000
N	2.78870770	2.06386350	-3.40000000
C	1.71575038	1.21126876	-3.40000000
N	2.07183073	-0.06671940	-3.40000000
C	10.58503531	-5.18917231	-3.40000000
C	9.17773124	-5.02564626	-3.40000000
C	8.92625174	-3.62858215	-3.40000000
C	10.16971540	-2.99849514	-3.40000000
N	11.16043854	-3.96279240	-3.40000000
O	9.54701312	-0.79648781	-3.40000000

H	7.96520671	-3.14170682	-3.40000000
H	12.15434048	-3.75807039	-3.40000000
H	11.17666980	-6.09299424	-3.40000000
N	10.48931566	-1.63328810	-3.40000000
O	11.71124742	-1.30301432	-3.40000000
C	8.21691370	-6.06690299	-3.40000000
C	7.38629749	-6.95267930	-3.40000000
C	6.39027876	-8.01798198	-3.40000000
H	5.74817660	-7.95984413	-2.51155335
H	5.62383242	2.66736114	-3.40000000
H	5.74817660	-7.95984413	-4.28844665
H	6.87192898	-9.00360272	-3.40000000
N	5.01072929	0.99340199	-0.00000000
O	4.30311303	-3.58754463	-0.00000000
N	4.56576767	-1.29129485	-0.00000000
C	4.09065743	0.00627844	-0.00000000
N	2.78482728	0.30606844	-0.00000000
C	1.98994643	-0.78688802	-0.00000000
C	2.37464339	-2.13938648	-0.00000000
C	3.76637283	-2.44955519	-0.00000000
N	0.61177148	-0.80968027	-0.00000000
C	0.22028517	-2.13921842	-0.00000000
N	1.25033840	-2.96889971	-0.00000000
H	6.02485359	0.80644199	-0.00000000
H	4.67541456	1.94791441	-0.00000000
H	5.60094570	-1.44221118	-0.00000000
H	0.00000000	-0.00000000	-0.00000000
H	-0.82386801	-2.42227350	-0.00000000
O	7.83019934	0.55673593	-0.00000000
N	7.14222402	-4.00240580	-0.00000000
N	7.45611902	-1.71718501	-0.00000000
C	9.41029341	-3.17541626	-0.00000000
C	8.26404832	-0.62432069	-0.00000000
N	9.65144142	-0.82879605	-0.00000000
C	10.20904031	-2.07360344	-0.00000000
C	7.99014840	-2.96108357	-0.00000000
H	9.82814656	-4.17525163	-0.00000000
H	10.23841777	-0.00000000	-0.00000000
H	11.29222369	-2.11783835	-0.00000000
H	6.12182623	-3.85260423	-0.00000000
H	7.50463415	-4.94693884	-0.00000000

Is–Px//G–C

Total bonding energy = -10254.18 kcal mol^{-1}

H	0.60815362	1.67489124	-3.40000000
H	7.16917191	0.57875811	-3.40000000
H	2.77552285	2.96159209	-3.40000000
C	2.65515736	-3.32532190	-3.40000000
C	2.68816887	-4.75065765	-3.40000000
C	3.96495953	-5.25738230	-3.40000000
S	5.15009158	-3.99036441	-3.40000000
C	3.90782169	-2.72793163	-3.40000000

H	6.41140654	-1.73178855	-3.40000000
H	1.72812454	-2.77032242	-3.40000000
H	1.79863530	-5.37349610	-3.40000000
H	4.28225101	-6.29122740	-3.40000000
C	5.65608348	-0.95192250	-3.40000000
C	6.10080029	0.38074437	-3.40000000
C	5.19326458	1.44001641	-3.40000000
C	3.83477033	1.10907461	-3.40000000
C	3.34524086	-0.24350671	-3.40000000
C	4.29415866	-1.31344386	-3.40000000
N	2.73508462	1.94997131	-3.40000000
C	1.57843238	1.19703372	-3.40000000
C	1.90587870	-0.14490763	-3.40000000
C	10.57808324	-5.28488597	-3.40000000
C	9.16832988	-5.14138499	-3.40000000
C	8.89705317	-3.74825640	-3.40000000
C	10.13190605	-3.10045504	-3.40000000
N	11.13604680	-4.05064945	-3.40000000
O	9.47890935	-0.90729026	-3.40000000
H	7.93052111	-3.27207000	-3.40000000
H	12.12717861	-3.83278352	-3.40000000
H	11.18228704	-6.18034891	-3.40000000
N	10.43259879	-1.73110707	-3.40000000
O	11.64985668	-1.38394735	-3.40000000
C	8.22407321	-6.19754904	-3.40000000
C	7.41143277	-7.09994709	-3.40000000
C	6.43909575	-8.18709194	-3.40000000
H	5.52345984	2.47582851	-3.40000000
H	5.79581808	-8.14367029	-4.28837562
H	5.79581808	-8.14367029	-2.51162438
H	1.17840196	-0.94093439	-3.40000000
H	6.94327239	-9.16137344	-3.40000000
N	5.01072929	0.99340199	-0.00000000
O	4.30311303	-3.58754463	-0.00000000
N	4.56576767	-1.29129485	-0.00000000
C	4.09065743	0.00627844	-0.00000000
N	2.78482728	0.30606844	-0.00000000
C	1.98994643	-0.78688802	-0.00000000
C	2.37464339	-2.13938648	-0.00000000
C	3.76637283	-2.44955519	-0.00000000
N	0.61177148	-0.80968027	-0.00000000
C	0.22028517	-2.13921842	-0.00000000
N	1.25033840	-2.96889971	-0.00000000
H	6.02485359	0.80644199	-0.00000000
H	4.67541456	1.94791441	-0.00000000
H	5.60094570	-1.44221118	-0.00000000
H	0.00000000	-0.00000000	-0.00000000
H	-0.82386801	-2.42227350	-0.00000000
O	7.83019934	0.55673593	-0.00000000
N	7.14222402	-4.00240580	-0.00000000
N	7.45611902	-1.71718501	-0.00000000
C	9.41029341	-3.17541626	-0.00000000

C	8.26404832	-0.62432069	-0.00000000
N	9.65144142	-0.82879605	-0.00000000
C	10.20904031	-2.07360344	-0.00000000
C	7.99014840	-2.96108357	-0.00000000
H	9.82814656	-4.17525163	-0.00000000
H	10.23841777	-0.00000000	-0.00000000
H	11.29222369	-2.11783835	-0.00000000
H	6.12182623	-3.85260423	-0.00000000
H	7.50463415	-4.94693884	-0.00000000

Ys–Px//G–C

Total bonding energy = -10154.95 kcal mol^{-1}

H	0.64192923	1.70283481	-3.40000000
H	7.05811210	0.63070536	-3.40000000
H	2.85575822	2.92722431	-3.40000000
C	2.62777108	-3.36528416	-3.40000000
C	2.66218319	-4.78797770	-3.40000000
C	3.94237034	-5.29126122	-3.40000000
S	5.12626345	-4.02819704	-3.40000000
C	3.88275907	-2.77192143	-3.40000000
H	6.39936880	-1.71469646	-3.40000000
H	1.70296744	-2.80570264	-3.40000000
H	1.77496692	-5.41353821	-3.40000000
H	4.25941847	-6.32513055	-3.40000000
C	5.61305240	-0.96756055	-3.40000000
C	6.00020042	0.37666845	-3.40000000
N	5.13229500	1.41150514	-3.40000000
C	3.84500126	1.04350781	-3.40000000
C	3.31380257	-0.29459251	-3.40000000
C	4.25692001	-1.35990745	-3.40000000
N	2.77641701	1.91702581	-3.40000000
C	1.59673133	1.19532436	-3.40000000
C	1.88119952	-0.15638669	-3.40000000
C	10.55720163	-5.25213568	-3.40000000
C	9.14602194	-5.12531306	-3.40000000
C	8.85852238	-3.73574022	-3.40000000
C	10.08495680	-3.07319759	-3.40000000
N	11.10051831	-4.01102956	-3.40000000
O	9.40255429	-0.88892240	-3.40000000
H	7.88634360	-3.27307195	-3.40000000
H	12.08889570	-3.78104274	-3.40000000
H	11.17220631	-6.14020065	-3.40000000
N	10.36742499	-1.69963973	-3.40000000
O	11.57970300	-1.33636381	-3.40000000
C	8.21378510	-6.19194794	-3.40000000
C	7.41002472	-7.10223974	-3.40000000
C	6.44753277	-8.19817863	-3.40000000
H	5.80430536	-8.16009338	-2.51129765
H	1.13118331	-0.93198169	-3.40000000
H	5.80430536	-8.16009338	-4.28870235
H	6.95917807	-9.16841444	-3.40000000
N	5.01072929	0.99340199	-0.00000000

O	4.30311303	-3.58754463	-0.00000000
N	4.56576767	-1.29129485	-0.00000000
C	4.09065743	0.00627844	-0.00000000
N	2.78482728	0.30606844	-0.00000000
C	1.98994643	-0.78688802	-0.00000000
C	2.37464339	-2.13938648	-0.00000000
C	3.76637283	-2.44955519	-0.00000000
N	0.61177148	-0.80968027	-0.00000000
C	0.22028517	-2.13921842	-0.00000000
N	1.25033840	-2.96889971	-0.00000000
H	6.02485359	0.80644199	-0.00000000
H	4.67541456	1.94791441	-0.00000000
H	5.60094570	-1.44221118	-0.00000000
H	0.00000000	-0.00000000	-0.00000000
H	-0.82386801	-2.42227350	-0.00000000
O	7.83019934	0.55673593	-0.00000000
N	7.14222402	-4.00240580	-0.00000000
N	7.45611902	-1.71718501	-0.00000000
C	9.41029341	-3.17541626	-0.00000000
C	8.26404832	-0.62432069	-0.00000000
N	9.65144142	-0.82879605	-0.00000000
C	10.20904031	-2.07360344	-0.00000000
C	7.99014840	-2.96108357	-0.00000000
H	9.82814656	-4.17525163	-0.00000000
H	10.23841777	-0.00000000	-0.00000000
H	11.29222369	-2.11783835	-0.00000000
H	6.12182623	-3.85260423	-0.00000000
H	7.50463415	-4.94693884	-0.00000000

Yi–Px//G–C

Total bonding energy = -10244.00 kcal mol^{-1}

H	0.37840100	-1.70419558	3.40000000
H	6.45021521	0.62856237	3.40000000
H	2.78809506	-2.46784329	3.40000000
N	1.29115241	3.48530905	3.40000000
C	1.10079044	4.85295195	3.40000000
C	2.31766591	5.50623675	3.40000000
N	3.27045257	4.51018545	3.40000000
C	2.62034531	3.29095314	3.40000000
H	5.31676422	2.81387499	3.40000000
H	0.35993782	1.01447130	3.40000000
H	0.11084171	5.29092496	3.40000000
H	2.57721276	6.55346819	3.40000000
C	4.70997713	1.91407377	3.40000000
C	5.36215195	0.67342303	3.40000000
N	4.72402458	-0.51696256	3.40000000
C	3.38802282	-0.42541252	3.40000000
C	2.59927242	0.77597566	3.40000000
C	3.30432760	2.00511248	3.40000000
N	2.51350983	-1.49244038	3.40000000
C	1.21159667	-1.01480254	3.40000000
C	1.22311787	0.36656629	3.40000000

C	9.72206930	5.04305861	3.40000000
C	8.34987628	4.68863915	3.40000000
C	8.29244192	3.27157103	3.40000000
C	9.61003993	2.81725340	3.40000000
N	10.45954549	3.90696227	3.40000000
O	9.28870873	0.55161074	3.40000000
H	7.41431274	2.64938288	3.40000000
H	11.47231890	3.84161465	3.40000000
H	10.18467485	6.01912527	3.40000000
N	10.11057304	1.50701680	3.40000000
O	11.36485965	1.34406352	3.40000000
C	7.26948685	5.60574204	3.40000000
C	6.38929497	6.44346942	3.40000000
C	5.36614920	7.48433427	3.40000000
H	4.72853291	7.41578958	2.50972866
H	4.27472983	4.66069542	3.40000000
H	4.72853291	7.41578958	4.29027134
H	5.83102405	8.47755273	3.40000000
N	5.01072929	-0.99340199	0.00000000
O	4.30311303	3.58754463	0.00000000
N	4.56576767	1.29129485	0.00000000
C	4.09065743	-0.00627844	0.00000000
N	2.78482728	-0.30606844	0.00000000
C	1.98994643	0.78688802	0.00000000
C	2.37464339	2.13938648	0.00000000
C	3.76637283	2.44955519	0.00000000
N	0.61177148	0.80968027	0.00000000
C	0.22028517	2.13921842	0.00000000
N	1.25033840	2.96889971	0.00000000
H	6.02485359	-0.80644199	0.00000000
H	4.67541456	-1.94791441	0.00000000
H	5.60094570	1.44221118	0.00000000
H	0.00000000	0.00000000	0.00000000
H	-0.82386801	2.42227350	0.00000000
O	7.83019934	-0.55673593	0.00000000
N	7.14222402	4.00240580	0.00000000
N	7.45611902	1.71718501	0.00000000
C	9.41029341	3.17541626	0.00000000
C	8.26404832	0.62432069	0.00000000
N	9.65144142	0.82879605	0.00000000
C	10.20904031	2.07360344	0.00000000
C	7.99014840	2.96108357	0.00000000
H	9.82814656	4.17525163	0.00000000
H	10.23841777	0.00000000	0.00000000
H	11.29222369	2.11783835	0.00000000
H	6.12182623	3.85260423	0.00000000
H	7.50463415	4.94693884	0.00000000

Do–Px//G–C

Total bonding energy = -10116.45 kcal mol^{-1}

H	0.83172790	-1.62048722	3.40000000
H	7.31157005	-1.47061509	3.40000000

H	2.77851671	-3.26137032	3.40000000
C	3.29961009	2.95851830	3.40000000
C	3.68693291	4.33654550	3.40000000
C	5.05482761	4.35859748	3.40000000
O	5.55272907	3.07438349	3.40000000
C	4.45692783	2.20936630	3.40000000
H	6.89852799	0.94074628	3.40000000
H	2.29814538	2.55334276	3.40000000
H	3.03275719	5.19862856	3.40000000
H	5.79757907	5.14350356	3.40000000
C	6.04576234	0.27202634	3.40000000
C	6.28545215	-1.10838199	3.40000000
N	5.32033397	-2.05838251	3.40000000
C	4.08197629	-1.55275303	3.40000000
C	3.70550560	-0.18135939	3.40000000
C	4.73169959	0.79134659	3.40000000
N	2.88475211	-2.25240588	3.40000000
C	1.87282147	-1.32418974	3.40000000
N	2.31436344	-0.07120090	3.40000000
C	10.70604175	5.09723538	3.40000000
C	9.29142167	5.02050039	3.40000000
C	8.95051582	3.64168880	3.40000000
C	10.15495546	2.93707626	3.40000000
N	11.20363978	3.83829096	3.40000000
O	9.41411151	0.77008087	3.40000000
H	7.95938093	3.21427938	3.40000000
H	12.18261481	3.57110689	3.40000000
H	11.35183752	5.96316829	3.40000000
N	10.39895398	1.55620280	3.40000000
O	11.60130650	1.15996320	3.40000000
C	8.40254157	6.12363907	3.40000000
C	7.63136441	7.06202015	3.40000000
C	6.69249613	8.17852968	3.40000000
H	6.04747260	8.15167408	2.51220999
H	6.04747260	8.15167408	4.28779001
H	7.22502077	9.13748215	3.40000000
N	5.01072929	-0.99340199	0.00000000
O	4.30311303	3.58754463	0.00000000
N	4.56576767	1.29129485	0.00000000
C	4.09065743	-0.00627844	0.00000000
N	2.78482728	-0.30606844	0.00000000
C	1.98994643	0.78688802	0.00000000
C	2.37464339	2.13938648	0.00000000
C	3.76637283	2.44955519	0.00000000
N	0.61177148	0.80968027	0.00000000
C	0.22028517	2.13921842	0.00000000
N	1.25033840	2.96889971	0.00000000
H	6.02485359	-0.80644199	0.00000000
H	4.67541456	-1.94791441	0.00000000
H	5.60094570	1.44221118	0.00000000
H	0.00000000	0.00000000	0.00000000
H	-0.82386801	2.42227350	0.00000000

O	7.83019934	-0.55673593	0.00000000
N	7.14222402	4.00240580	0.00000000
N	7.45611902	1.71718501	0.00000000
C	9.41029341	3.17541626	0.00000000
C	8.26404832	0.62432069	0.00000000
N	9.65144142	0.82879605	0.00000000
C	10.20904031	2.07360344	0.00000000
C	7.99014840	2.96108357	0.00000000
H	9.82814656	4.17525163	0.00000000
H	10.23841777	0.00000000	0.00000000
H	11.29222369	2.11783835	0.00000000
H	6.12182623	3.85260423	0.00000000
H	7.50463415	4.94693884	0.00000000

Dp–Px//G–C

Total bonding energy = -10318.48 kcal mol^{-1}

H	0.90299203	-1.59974755	3.40000000
H	7.37774878	-1.40859283	3.40000000
H	2.86521489	-3.22135091	3.40000000
C	4.55450798	2.31120675	3.40000000
N	5.66457554	3.08772553	3.40000000
H	6.30360193	8.40064742	4.28716044
H	6.30360193	8.40064742	2.51283956
N	5.56383070	4.42522526	3.40000000
H	6.94110205	1.01196779	3.40000000
C	4.34846871	4.99676957	3.40000000
C	3.16098817	4.25799016	3.40000000
C	3.26253093	2.87746250	3.40000000
C	6.10069119	0.32931707	3.40000000
C	6.35104254	-1.04854386	3.40000000
N	5.38737788	-1.99911170	3.40000000
C	4.15035276	-1.50090987	3.40000000
C	3.76405434	-0.12542821	3.40000000
C	4.78952071	0.84631684	3.40000000
N	2.96087707	-2.21128838	3.40000000
C	1.94145145	-1.29439083	3.40000000
N	2.36884908	-0.03737438	3.40000000
C	10.86828814	5.20524071	3.40000000
C	9.45150753	5.18479713	3.40000000
C	9.05312721	3.81977829	3.40000000
C	10.23154412	3.06901810	3.40000000
N	11.31626402	3.92832362	3.40000000
O	9.42731241	0.92147098	3.40000000
H	8.04097706	3.43622229	3.40000000
H	12.28337796	3.62134510	3.40000000
H	11.54821675	6.04486557	3.40000000
N	10.43245501	1.68196800	3.40000000
O	11.62400283	1.24938123	3.40000000
C	8.61170476	6.32572278	3.40000000
C	7.86948997	7.28624629	3.40000000
C	6.95012538	8.41881703	3.40000000
H	2.19609066	4.75729378	3.40000000

H	2.39062486	2.23423583	3.40000000
H	4.34723337	6.08458121	3.40000000
H	7.49479143	9.37119328	3.40000000
N	5.01072929	-0.99340199	0.00000000
O	4.30311303	3.58754463	0.00000000
N	4.56576767	1.29129485	0.00000000
C	4.09065743	-0.00627844	0.00000000
N	2.78482728	-0.30606844	0.00000000
C	1.98994643	0.78688802	0.00000000
C	2.37464339	2.13938648	0.00000000
C	3.76637283	2.44955519	0.00000000
N	0.61177148	0.80968027	0.00000000
C	0.22028517	2.13921842	0.00000000
N	1.25033840	2.96889971	0.00000000
H	6.02485359	-0.80644199	0.00000000
H	4.67541456	-1.94791441	0.00000000
H	5.60094570	1.44221118	0.00000000
H	0.00000000	0.00000000	0.00000000
H	-0.82386801	2.42227350	0.00000000
O	7.83019934	-0.55673593	0.00000000
N	7.14222402	4.00240580	0.00000000
N	7.45611902	1.71718501	0.00000000
C	9.41029341	3.17541626	0.00000000
C	8.26404832	0.62432069	0.00000000
N	9.65144142	0.82879605	0.00000000
C	10.20904031	2.07360344	0.00000000
C	7.99014840	2.96108357	0.00000000
H	9.82814656	4.17525163	0.00000000
H	10.23841777	0.00000000	0.00000000
H	11.29222369	2.11783835	0.00000000
H	6.12182623	3.85260423	0.00000000
H	7.50463415	4.94693884	0.00000000

Dss–Px//G–C

Total bonding energy = -11089.89 kcal mol^{-1}

H	1.42285784	1.88356517	-3.40000000
H	7.79918240	0.71937179	-3.40000000
H	3.60574795	3.19583530	-3.40000000
C	3.11127386	-2.95657100	-3.40000000
C	3.00707564	-4.36506606	-3.40000000
C	4.23387603	-5.01890526	-3.40000000
S	5.54669649	-3.85067777	-3.40000000
C	4.41943413	-2.48919969	-3.40000000
H	7.01890974	-1.58727435	-3.40000000
H	2.26492312	-2.28053896	-3.40000000
H	2.05985877	-4.89557720	-3.40000000
H	3.86255718	-9.94588175	-3.40000000
C	6.27441968	-0.79827797	-3.40000000
C	6.72897184	0.52499262	-3.40000000
N	5.92253006	1.61355348	-3.40000000
C	4.62161242	1.30287205	-3.40000000
C	4.03468819	0.00565956	-3.40000000

C	4.89692225	-1.11981379	-3.40000000
N	3.55006499	2.18284221	-3.40000000
C	2.40430507	1.42711052	-3.40000000
N	2.64279887	0.12063623	-3.40000000
C	11.87341764	-5.40784950	-3.40000000
C	10.49393957	-5.73312686	-3.40000000
C	9.77961271	-4.50576364	-3.40000000
C	10.73844247	-3.49047379	-3.40000000
N	11.99718788	-4.06055471	-3.40000000
O	9.42741384	-1.61339239	-3.40000000
H	8.70891649	-4.36665584	-3.40000000
H	12.86171138	-3.52901577	-3.40000000
H	12.73586185	-6.05819916	-3.40000000
N	10.59001380	-2.09596905	-3.40000000
O	11.63642095	-1.38290800	-3.40000000
C	9.97659897	-7.05191035	-3.40000000
C	9.59473744	-8.20479354	-3.40000000
C	9.16273432	-9.59799320	-3.40000000
C	4.49724805	-6.43729192	-3.40000000
C	5.71125076	-7.11128205	-3.40000000
C	5.57041856	-8.52728162	-3.40000000
C	4.25678803	-8.93770959	-3.40000000
S	3.16263368	-7.59086571	-3.40000000
H	8.56228981	-9.82789541	-4.28922480
H	8.56228981	-9.82789541	-2.51077520
H	6.67187969	-6.60540115	-3.40000000
H	6.40394743	-9.21833856	-3.40000000
H	10.02968500	-10.27019328	-3.40000000
N	5.01072929	0.99340199	-0.00000000
O	4.30311303	-3.58754463	-0.00000000
N	4.56576767	-1.29129485	-0.00000000
C	4.09065743	0.00627844	-0.00000000
N	2.78482728	0.30606844	-0.00000000
C	1.98994643	-0.78688802	-0.00000000
C	2.37464339	-2.13938648	-0.00000000
C	3.76637283	-2.44955519	-0.00000000
N	0.61177148	-0.80968027	-0.00000000
C	0.22028517	-2.13921842	-0.00000000
N	1.25033840	-2.96889971	-0.00000000
H	6.02485359	0.80644199	-0.00000000
H	4.67541456	1.94791441	-0.00000000
H	5.60094570	-1.44221118	-0.00000000
H	0.00000000	-0.00000000	-0.00000000
H	-0.82386801	-2.42227350	-0.00000000
O	7.83019934	0.55673593	-0.00000000
N	7.14222402	-4.00240580	-0.00000000
N	7.45611902	-1.71718501	-0.00000000
C	9.41029341	-3.17541626	-0.00000000
C	8.26404832	-0.62432069	-0.00000000
N	9.65144142	-0.82879605	-0.00000000
C	10.20904031	-2.07360344	-0.00000000
C	7.99014840	-2.96108357	-0.00000000

H	9.82814656	-4.17525163	-0.00000000
H	10.23841777	-0.00000000	-0.00000000
H	11.29222369	-2.11783835	-0.00000000
H	6.12182623	-3.85260423	-0.00000000
H	7.50463415	-4.94693884	-0.00000000

Dt–Px//G–C

Total bonding energy = -9956.51 kcal mol^{-1}

H	0.69179222	1.69140735	-3.40000000
H	7.11268171	0.82777605	-3.40000000
H	2.81383403	3.10026961	-3.40000000
N	2.67048956	-3.01415065	-3.40000000
C	2.63844411	-4.38940793	-3.40000000
C	3.86174670	-5.01470496	-3.40000000
S	5.13013964	-3.84498496	-3.40000000
C	3.90899368	-2.55330804	-3.40000000
H	6.44023662	-1.51330134	-3.40000000
H	5.71171532	-7.87750018	-4.28881855
H	1.68249435	-4.90306447	-3.40000000
H	4.07474082	-6.07473789	-3.40000000
C	5.65904570	-0.76093138	-3.40000000
C	6.05367331	0.58100410	-3.40000000
N	5.19227437	1.62445415	-3.40000000
C	3.90988496	1.25503920	-3.40000000
C	3.37888636	-0.07223071	-3.40000000
C	4.30058077	-1.14523825	-3.40000000
N	2.80233325	2.08571800	-3.40000000
C	1.69259339	1.27885037	-3.40000000
N	1.98585656	-0.01649148	-3.40000000
C	10.54602627	-5.09349986	-3.40000000
C	9.13703828	-4.94385259	-3.40000000
C	8.87255732	-3.54990713	-3.40000000
C	10.10919314	-2.90697815	-3.40000000
N	11.10931726	-3.86120190	-3.40000000
O	9.45947272	-0.71286788	-3.40000000
H	7.90852253	-3.07077926	-3.40000000
H	12.10134912	-3.64750509	-3.40000000
H	11.14666839	-5.99130575	-3.40000000
N	10.41236133	-1.53788282	-3.40000000
O	11.62971003	-1.19275576	-3.40000000
C	8.18454166	-5.99226318	-3.40000000
C	7.35309274	-6.87728318	-3.40000000
C	6.35291325	-7.93884225	-3.40000000
H	5.71171532	-7.87750018	-2.51118145
H	6.82951428	-8.92673127	-3.40000000
N	5.01072929	0.99340199	-0.00000000
O	4.30311303	-3.58754463	-0.00000000
N	4.56576767	-1.29129485	-0.00000000
C	4.09065743	0.00627844	-0.00000000
N	2.78482728	0.30606844	-0.00000000
C	1.98994643	-0.78688802	-0.00000000
C	2.37464339	-2.13938648	-0.00000000

C	3.76637283	-2.44955519	-0.00000000
N	0.61177148	-0.80968027	-0.00000000
C	0.22028517	-2.13921842	-0.00000000
N	1.25033840	-2.96889971	-0.00000000
H	6.02485359	0.80644199	-0.00000000
H	4.67541456	1.94791441	-0.00000000
H	5.60094570	-1.44221118	-0.00000000
H	0.00000000	-0.00000000	-0.00000000
H	-0.82386801	-2.42227350	-0.00000000
O	7.83019934	0.55673593	-0.00000000
N	7.14222402	-4.00240580	-0.00000000
N	7.45611902	-1.71718501	-0.00000000
C	9.41029341	-3.17541626	-0.00000000
C	8.26404832	-0.62432069	-0.00000000
N	9.65144142	-0.82879605	-0.00000000
C	10.20904031	-2.07360344	-0.00000000
C	7.99014840	-2.96108357	-0.00000000
H	9.82814656	-4.17525163	-0.00000000
H	10.23841777	-0.00000000	-0.00000000
H	11.29222369	-2.11783835	-0.00000000
H	6.12182623	-3.85260423	-0.00000000
H	7.50463415	-4.94693884	-0.00000000

Yo–Px/G–C

Total bonding energy = -10213.52 kcal mol^{-1}

H	0.73481030	-1.69826134	3.40000000
H	7.23106721	-1.32836962	3.40000000
H	2.79857361	-3.16005370	3.40000000
C	3.29191973	3.16471482	3.40000000
C	3.72712808	4.52881969	3.40000000
C	5.09370591	4.50227429	3.40000000
O	5.54350639	3.20051503	3.40000000
C	4.41954333	2.37021807	3.40000000
H	6.82825093	1.08861187	3.40000000
H	2.26775472	2.82448877	3.40000000
H	3.10203786	5.41214400	3.40000000
H	5.86350723	5.26076117	3.40000000
C	5.97330017	0.42283437	3.40000000
C	6.20739902	-0.95828767	3.40000000
N	5.23492374	-1.89643988	3.40000000
C	3.99192421	-1.39675703	3.40000000
C	3.60961214	-0.01242376	3.40000000
C	4.66123318	0.94188892	3.40000000
N	2.83284288	-2.14732641	3.40000000
C	1.73952973	-1.29859301	3.40000000
C	2.17233392	0.01380385	3.40000000
C	10.71770847	5.19751909	3.40000000
C	9.30220551	5.14083224	3.40000000
C	8.94182294	3.76688834	3.40000000
C	10.13608064	3.04549987	3.40000000
N	11.19754096	3.93157906	3.40000000
O	9.36163707	0.89079849	3.40000000

H	7.94549388	3.35161809	3.40000000
H	12.17265353	3.65061402	3.40000000
H	11.37561885	6.05429235	3.40000000
N	10.35872270	1.66105467	3.40000000
O	11.55493021	1.24638627	3.40000000
C	8.42863781	6.25612586	3.40000000
C	7.66816382	7.20299234	3.40000000
C	6.74106138	8.32927145	3.40000000
H	6.09575171	8.30902424	4.28776195
H	6.09575171	8.30902424	2.51223805
H	1.51992512	0.87420558	3.40000000
H	7.28365717	9.28258100	3.40000000
N	5.01072929	-0.99340199	0.00000000
O	4.30311303	3.58754463	0.00000000
N	4.56576767	1.29129485	0.00000000
C	4.09065743	-0.00627844	0.00000000
N	2.78482728	-0.30606844	0.00000000
C	1.98994643	0.78688802	0.00000000
C	2.37464339	2.13938648	0.00000000
C	3.76637283	2.44955519	0.00000000
N	0.61177148	0.80968027	0.00000000
C	0.22028517	2.13921842	0.00000000
N	1.25033840	2.96889971	0.00000000
H	6.02485359	-0.80644199	0.00000000
H	4.67541456	-1.94791441	0.00000000
H	5.60094570	1.44221118	0.00000000
H	0.00000000	0.00000000	0.00000000
H	-0.82386801	2.42227350	0.00000000
O	7.83019934	-0.55673593	0.00000000
N	7.14222402	4.00240580	0.00000000
N	7.45611902	1.71718501	0.00000000
C	9.41029341	3.17541626	0.00000000
C	8.26404832	0.62432069	0.00000000
N	9.65144142	0.82879605	0.00000000
C	10.20904031	2.07360344	0.00000000
C	7.99014840	2.96108357	0.00000000
H	9.82814656	4.17525163	0.00000000
H	10.23841777	0.00000000	0.00000000
H	11.29222369	2.11783835	0.00000000
H	6.12182623	3.85260423	0.00000000
H	7.50463415	4.94693884	0.00000000

Ps–Px//G–C

Total bonding energy = -9957.89 kcal mol^{-1}

H	0.58954481	1.58496551	-3.40000000
H	6.80341692	-0.20095692	-3.40000000
H	2.87130661	2.71947295	-3.40000000
C	2.00562231	-3.44967829	-3.40000000
C	1.90361809	-4.86668246	-3.40000000
C	3.13570315	-5.48755763	-3.40000000
S	4.44502529	-4.35320212	-3.40000000
C	3.31994943	-3.00273638	-3.40000000

H	5.31371529	-7.94358011	-4.28850893
H	1.16115404	-2.77043928	-3.40000000
H	0.96247232	-5.40779957	-3.40000000
H	3.34339014	-6.54981852	-3.40000000
N	5.19331924	-1.49249872	-3.40000000
C	5.71781925	-0.25564618	-3.40000000
N	5.05445633	0.92131285	-3.40000000
C	3.72607096	0.75303214	-3.40000000
C	3.03943753	-0.48933604	-3.40000000
C	3.83833206	-1.65119857	-3.40000000
N	2.73672421	1.71359912	-3.40000000
C	1.52940818	1.04832093	-3.40000000
N	1.66050201	-0.27106727	-3.40000000
C	10.03850755	-5.05421463	-3.40000000
C	8.63189918	-4.88217958	-3.40000000
C	8.38217038	-3.48244586	-3.40000000
C	9.63570504	-2.86282388	-3.40000000
N	10.62032762	-3.83358401	-3.40000000
O	9.07673555	-0.63914655	-3.40000000
H	7.42099634	-2.98467416	-3.40000000
H	11.61516228	-3.63331005	-3.40000000
H	10.62309834	-5.96260024	-3.40000000
N	9.99079866	-1.50654766	-3.40000000
O	11.22200447	-1.21022976	-3.40000000
C	7.69178477	-5.94190830	-3.40000000
C	6.90813272	-6.86930543	-3.40000000
C	5.95745840	-7.97534157	-3.40000000
H	5.31371529	-7.94358011	-2.51149107
H	6.47871464	-8.94036610	-3.40000000
N	5.01072929	0.99340199	-0.00000000
O	4.30311303	-3.58754463	-0.00000000
N	4.56576767	-1.29129485	-0.00000000
C	4.09065743	0.00627844	-0.00000000
N	2.78482728	0.30606844	-0.00000000
C	1.98994643	-0.78688802	-0.00000000
C	2.37464339	-2.13938648	-0.00000000
C	3.76637283	-2.44955519	-0.00000000
N	0.61177148	-0.80968027	-0.00000000
C	0.22028517	-2.13921842	-0.00000000
N	1.25033840	-2.96889971	-0.00000000
H	6.02485359	0.80644199	-0.00000000
H	4.67541456	1.94791441	-0.00000000
H	5.60094570	-1.44221118	-0.00000000
H	0.00000000	-0.00000000	-0.00000000
H	-0.82386801	-2.42227350	-0.00000000
O	7.83019934	0.55673593	-0.00000000
N	7.14222402	-4.00240580	-0.00000000
N	7.45611902	-1.71718501	-0.00000000
C	9.41029341	-3.17541626	-0.00000000
C	8.26404832	-0.62432069	-0.00000000
N	9.65144142	-0.82879605	-0.00000000
C	10.20904031	-2.07360344	-0.00000000

C	7.99014840	-2.96108357	-0.00000000
H	9.82814656	-4.17525163	-0.00000000
H	10.23841777	-0.00000000	-0.00000000
H	11.29222369	-2.11783835	-0.00000000
H	6.12182623	-3.85260423	-0.00000000
H	7.50463415	-4.94693884	-0.00000000

Es–Px//G–C

Total bonding energy = -10055.97 kcal mol^{-1}

H	0.53120489	1.67307573	-3.40000000
H	6.71628395	-0.30621809	-3.40000000
H	2.87829663	2.61462531	-3.40000000
C	1.99178517	-3.65743559	-3.40000000
C	1.95200508	-5.07868366	-3.40000000
C	3.20918737	-5.64387131	-3.40000000
S	4.46493399	-4.45035690	-3.40000000
C	3.28210578	-3.14713456	-3.40000000
H	0.69233741	-1.01240070	-3.40000000
H	1.10191636	-3.04134598	-3.40000000
H	1.03416244	-5.65846219	-3.40000000
H	3.46310732	-6.69563439	-3.40000000
N	5.11642730	-1.61364653	-3.40000000
C	5.63134872	-0.37229345	-3.40000000
N	4.95304734	0.79209972	-3.40000000
C	3.62269057	0.62534654	-3.40000000
C	2.93207883	-0.63197868	-3.40000000
C	3.76337389	-1.77628377	-3.40000000
N	2.67840807	1.62082413	-3.40000000
C	1.41284560	1.04751836	-3.40000000
C	1.52641434	-0.32704078	-3.40000000
C	10.01315377	-5.14104939	-3.40000000
C	8.60444721	-4.98540676	-3.40000000
C	8.33859745	-3.58872058	-3.40000000
C	9.58507431	-2.95470516	-3.40000000
N	10.58083438	-3.91390278	-3.40000000
O	9.00039533	-0.73785511	-3.40000000
H	7.37304486	-3.09968793	-3.40000000
H	11.57338979	-3.70272965	-3.40000000
H	10.60809812	-6.04271325	-3.40000000
N	9.92448183	-1.59441288	-3.40000000
O	11.15234071	-1.28385645	-3.40000000
C	7.68016989	-6.05928747	-3.40000000
C	6.91995663	-7.00607303	-3.40000000
C	6.00249773	-8.13973435	-3.40000000
H	5.35823661	-8.12786901	-2.51139688
H	5.35823661	-8.12786901	-4.28860312
H	6.55351609	-9.08818618	-3.40000000
N	5.01072929	0.99340199	-0.00000000
O	4.30311303	-3.58754463	-0.00000000
N	4.56576767	-1.29129485	-0.00000000
C	4.09065743	0.00627844	-0.00000000
N	2.78482728	0.30606844	-0.00000000

C	1.98994643	-0.78688802	-0.00000000
C	2.37464339	-2.13938648	-0.00000000
C	3.76637283	-2.44955519	-0.00000000
N	0.61177148	-0.80968027	-0.00000000
C	0.22028517	-2.13921842	-0.00000000
N	1.25033840	-2.96889971	-0.00000000
H	6.02485359	0.80644199	-0.00000000
H	4.67541456	1.94791441	-0.00000000
H	5.60094570	-1.44221118	-0.00000000
H	0.00000000	-0.00000000	-0.00000000
H	-0.82386801	-2.42227350	-0.00000000
O	7.83019934	0.55673593	-0.00000000
N	7.14222402	-4.00240580	-0.00000000
N	7.45611902	-1.71718501	-0.00000000
C	9.41029341	-3.17541626	-0.00000000
C	8.26404832	-0.62432069	-0.00000000
N	9.65144142	-0.82879605	-0.00000000
C	10.20904031	-2.07360344	-0.00000000
C	7.99014840	-2.96108357	-0.00000000
H	9.82814656	-4.17525163	-0.00000000
H	10.23841777	-0.00000000	-0.00000000
H	11.29222369	-2.11783835	-0.00000000
H	6.12182623	-3.85260423	-0.00000000
H	7.50463415	-4.94693884	-0.00000000

Ysm–Px//G–C

Total bonding energy = -10523.94 kcal mol^{-1}

H	0.94119192	-1.81316196	3.40000000
H	7.36096185	-0.76144552	3.40000000
H	3.15093817	-3.04484173	3.40000000
C	2.94929441	3.25248283	3.40000000
C	2.99332736	4.67508416	3.40000000
C	4.27000436	5.19327996	3.40000000
S	5.44655135	3.90243178	3.40000000
C	4.19685025	2.64830383	3.40000000
H	6.71308737	1.58759914	3.40000000
H	2.01998613	2.69970908	3.40000000
H	2.10776626	5.30450018	3.40000000
C	4.70153215	6.62936108	3.40000000
C	5.92309314	0.84327935	3.40000000
C	6.30394207	-0.50301989	3.40000000
N	5.43356911	-1.53629993	3.40000000
C	4.14687162	-1.16416340	3.40000000
C	3.62051583	0.17475145	3.40000000
C	4.56681961	1.23807232	3.40000000
N	3.07511149	-2.03438941	3.40000000
C	1.89755248	-1.30862144	3.40000000
C	2.18725106	0.04204757	3.40000000
C	11.16343816	5.40058237	3.40000000
C	9.74866040	5.48043521	3.40000000
C	9.26038237	4.14757862	3.40000000
C	10.37864999	3.31387104	3.40000000

N	11.51999256	4.09400579	3.40000000
O	9.39555923	1.24607915	3.40000000
H	8.22948033	3.83210907	3.40000000
H	12.46390031	3.72142132	3.40000000
H	11.90082875	6.18990699	3.40000000
N	10.46387418	1.91427092	3.40000000
O	11.61341146	1.38371442	3.40000000
C	8.98645101	6.67474257	3.40000000
C	8.33651976	7.70064656	3.40000000
C	7.56152733	8.93633554	3.40000000
H	6.91862765	9.00033143	2.51301604
H	5.30598708	6.86746666	4.28354978
H	3.82073112	7.27919755	3.40000000
H	5.30598708	6.86746666	2.51645022
H	1.44023094	0.82075669	3.40000000
H	6.91862765	9.00033143	4.28698396
H	8.22286077	9.81151535	3.40000000
N	5.01072929	-0.99340199	0.00000000
O	4.30311303	3.58754463	0.00000000
N	4.56576767	1.29129485	0.00000000
C	4.09065743	-0.00627844	0.00000000
N	2.78482728	-0.30606844	0.00000000
C	1.98994643	0.78688802	0.00000000
C	2.37464339	2.13938648	0.00000000
C	3.76637283	2.44955519	0.00000000
N	0.61177148	0.80968027	0.00000000
C	0.22028517	2.13921842	0.00000000
N	1.25033840	2.96889971	0.00000000
H	6.02485359	-0.80644199	0.00000000
H	4.67541456	-1.94791441	0.00000000
H	5.60094570	1.44221118	0.00000000
H	0.00000000	0.00000000	0.00000000
H	-0.82386801	2.42227350	0.00000000
O	7.83019934	-0.55673593	0.00000000
N	7.14222402	4.00240580	0.00000000
N	7.45611902	1.71718501	0.00000000
C	9.41029341	3.17541626	0.00000000
C	8.26404832	0.62432069	0.00000000
N	9.65144142	0.82879605	0.00000000
C	10.20904031	2.07360344	0.00000000
C	7.99014840	2.96108357	0.00000000
H	9.82814656	4.17525163	0.00000000
H	10.23841777	0.00000000	0.00000000
H	11.29222369	2.11783835	0.00000000
H	6.12182623	3.85260423	0.00000000
H	7.50463415	4.94693884	0.00000000

Yss–Px//G–C

Total bonding energy = -11186.18 kcal mol^{-1}

H	1.32559022	2.01044801	-3.40000000
H	7.66332862	0.54117427	-3.40000000
H	3.61036189	3.09467798	-3.40000000

C	3.00208530	-3.17130275	-3.40000000
C	2.94390124	-4.58367099	-3.40000000
C	4.18999513	-5.19572099	-3.40000000
S	5.46067430	-3.98245591	-3.40000000
C	4.29027684	-2.65282732	-3.40000000
H	6.86375955	-1.76000047	-3.40000000
H	2.11370726	-2.55546946	-3.40000000
H	2.01308622	-5.14238977	-3.40000000
C	4.50279894	-6.60431385	-3.40000000
C	6.12509924	-0.96497619	-3.40000000
C	6.59186815	0.35336786	-3.40000000
N	5.78959586	1.44077551	-3.40000000
C	4.48162546	1.15284884	-3.40000000
C	3.86867012	-0.14900193	-3.40000000
C	4.74619159	-1.27085833	-3.40000000
N	3.46905623	2.09126790	-3.40000000
C	2.24684415	1.44441218	-3.40000000
C	2.44762028	0.07736552	-3.40000000
C	11.85178223	-5.47803345	-3.40000000
C	10.48249604	-5.84320913	-3.40000000
C	9.73291661	-4.63685233	-3.40000000
C	10.66197075	-3.59429607	-3.40000000
N	11.93662407	-4.12770084	-3.40000000
O	9.29720223	-1.75606208	-3.40000000
H	8.65870675	-4.52769411	-3.40000000
H	12.78542362	-3.57139457	-3.40000000
H	12.73274979	-6.10310332	-3.40000000
N	10.47303582	-2.20445692	-3.40000000
O	11.49858668	-1.46150713	-3.40000000
C	10.00508299	-7.17704959	-3.40000000
C	9.65996633	-8.34149880	-3.40000000
C	9.27309922	-9.74799516	-3.40000000
C	5.73968960	-7.23448444	-3.40000000
C	5.65003042	-8.65504877	-3.40000000
C	4.35225079	-9.11240733	-3.40000000
S	3.21049309	-7.80503256	-3.40000000
H	6.68211120	-6.69554799	-3.40000000
H	8.67824982	-9.99782377	-4.28777903
H	8.67824982	-9.99782377	-2.51222097
H	6.50924419	-9.31592678	-3.40000000
H	3.99354236	-10.13377430	-3.40000000
H	1.65037386	-0.64986456	-3.40000000
H	10.16041638	-10.39284981	-3.40000000
N	5.01072929	0.99340199	-0.00000000
O	4.30311303	-3.58754463	-0.00000000
N	4.56576767	-1.29129485	-0.00000000
C	4.09065743	0.00627844	-0.00000000
N	2.78482728	0.30606844	-0.00000000
C	1.98994643	-0.78688802	-0.00000000
C	2.37464339	-2.13938648	-0.00000000
C	3.76637283	-2.44955519	-0.00000000
N	0.61177148	-0.80968027	-0.00000000

C	0.22028517	-2.13921842	-0.00000000
N	1.25033840	-2.96889971	-0.00000000
H	6.02485359	0.80644199	-0.00000000
H	4.67541456	1.94791441	-0.00000000
H	5.60094570	-1.44221118	-0.00000000
H	0.00000000	-0.00000000	-0.00000000
H	-0.82386801	-2.42227350	-0.00000000
O	7.83019934	0.55673593	-0.00000000
N	7.14222402	-4.00240580	-0.00000000
N	7.45611902	-1.71718501	-0.00000000
C	9.41029341	-3.17541626	-0.00000000
C	8.26404832	-0.62432069	-0.00000000
N	9.65144142	-0.82879605	-0.00000000
C	10.20904031	-2.07360344	-0.00000000
C	7.99014840	-2.96108357	-0.00000000
H	9.82814656	-4.17525163	-0.00000000
H	10.23841777	-0.00000000	-0.00000000
H	11.29222369	-2.11783835	-0.00000000
H	6.12182623	-3.85260423	-0.00000000
H	7.50463415	-4.94693884	-0.00000000

S11.4. Water

Cartesian Coordinates and Total Bonding Energies of W–C base pairs in Water

Cartesian coordinates (Å) and total bonding energies (kcal mol^{-1}) of Watson–Crick base pairs optimized at ZORA-BLYP-D3(BJ)/TZ2P level of theory in water.

A–T

Total bonding energy = -4511.46 kcal mol^{-1}

N	1.28858795	-0.60540280	0.00000000
C	1.90052610	-1.80617620	0.00000000
N	3.21953885	-2.06184995	0.00000000
C	3.94894520	-0.92387928	0.00000000
C	3.44496835	0.38865004	0.00000000
C	2.03673659	0.53568791	0.00000000
N	5.32149058	-0.77857719	0.00000000
C	5.58830621	0.57514970	0.00000000
N	4.48722281	1.31556078	0.00000000
N	1.40495228	1.72410539	0.00000000
H	1.23211496	-2.66600643	0.00000000
H	6.00888793	-1.52504551	0.00000000
H	6.60221800	0.95296960	0.00000000
H	1.94354225	2.58024523	0.00000000
H	0.37866657	1.76242513	0.00000000
N	-1.57931416	-0.57122313	0.00000000
C	-2.20061046	-1.80369398	0.00000000
N	-3.58349330	-1.73545773	0.00000000
C	-4.28445190	-0.55215882	0.00000000
C	-3.66371366	0.65891279	0.00000000
C	-2.20918153	0.67373526	0.00000000
O	-1.51824393	1.71996141	0.00000000
O	-1.57986727	-2.87937702	0.00000000
C	-4.41237171	1.96407562	0.00000000
H	-0.52672437	-0.58263298	0.00000000
H	-4.08242115	-2.61951485	0.00000000
H	-5.36426944	-0.65720400	0.00000000
H	-4.15478111	2.56342671	0.88220861
H	-5.49247954	1.78986770	0.00000000
H	-4.15478111	2.56342671	-0.88220861

G–C

Total bonding energy = -4421.74 kcal mol^{-1}

N	-2.55667082	-0.57076076	0.00000000
O	1.36972302	1.89196095	0.00000000
N	-0.63494844	0.74177981	0.00000000
C	-2.01465881	0.66462684	0.00000000
N	-2.80669552	1.74557126	0.00000000
C	-2.11763928	2.90819140	0.00000000
C	-0.72333927	3.08893903	0.00000000
C	0.11142047	1.93358613	0.00000000
N	-2.64117627	4.18300578	0.00000000
C	-1.57533994	5.06785881	0.00000000

N	-0.40556027	4.44989177	0.00000000
H	-1.98306781	-1.42772052	0.00000000
H	-3.56584598	-0.64231978	0.00000000
H	-0.08750607	-0.14938627	0.00000000
H	-3.62626191	4.42691203	0.00000000
H	-1.72792585	6.13884831	0.00000000
O	-1.04286758	-2.99050014	0.00000000
N	2.87451533	-0.55873011	0.00000000
N	0.89870364	-1.74943198	0.00000000
C	3.01012102	-2.96869376	0.00000000
C	0.21462009	-2.92364408	0.00000000
N	0.94922349	-4.11645527	0.00000000
C	2.31321997	-4.13755110	0.00000000
C	2.25273353	-1.74899506	0.00000000
H	4.09373298	-2.95764483	0.00000000
H	0.42128989	-4.98407105	0.00000000
H	2.78084744	-5.11548300	0.00000000
H	2.33394192	0.31878740	0.00000000
H	3.88541094	-0.51857194	0.00000000

A–T//G–C

Total bonding energy = -8943.89 kcal mol^{-1}

N	5.26125720	-0.85009895	-3.40000000
C	5.61428583	0.45055313	-3.40000000
N	4.80870712	1.52582363	-3.40000000
C	3.50402339	1.17253727	-3.40000000
C	2.99594959	-0.13841158	-3.40000000
C	3.94059150	-1.19310988	-3.40000000
N	2.38948970	1.98666759	-3.40000000
C	1.28265026	1.16285116	-3.40000000
N	1.60121532	-0.12521310	-3.40000000
N	3.61056545	-2.49793566	-3.40000000
H	6.68698043	0.63872266	-3.40000000
H	2.38150207	3.00139232	-3.40000000
H	0.27767972	1.56384893	-3.40000000
H	2.63653675	-2.77055752	-3.40000000
H	4.34522883	-3.21557101	-3.40000000
N	7.36319668	-2.80147826	-3.40000000
C	8.65124458	-2.30556892	-3.40000000
N	9.63002729	-3.28485721	-3.40000000
C	9.35469164	-4.63234731	-3.40000000
C	8.08142873	-5.11277625	-3.40000000
C	6.99377801	-4.14690910	-3.40000000
O	5.77921138	-4.45805508	-3.40000000
O	8.91373991	-1.09168577	-3.40000000
C	7.75959208	-6.58259258	-3.40000000
H	6.59097159	-2.08611551	-3.40000000
H	10.59341839	-2.96491412	-3.40000000
H	10.22530044	-5.27971176	-3.40000000
H	7.16621903	-6.85367019	-2.51779139
H	8.67686498	-7.17890773	-3.40000000
H	7.16621903	-6.85367019	-4.28220861

N	5.01365243	0.99199182	-0.00000000
O	4.30260090	-3.58796152	-0.00000000
N	4.56716532	-1.29195847	-0.00000000
C	4.09292299	0.00598124	-0.00000000
N	2.78699383	0.30650083	-0.00000000
C	1.99120808	-0.78584029	-0.00000000
C	2.37604854	-2.13811247	-0.00000000
C	3.76721199	-2.44848064	-0.00000000
N	0.61326544	-0.80857250	-0.00000000
C	0.22151194	-2.13729412	-0.00000000
N	1.25137639	-2.96774397	-0.00000000
H	6.02751807	0.80363867	-0.00000000
H	4.68066827	1.94733299	-0.00000000
H	5.60211828	-1.44276463	-0.00000000
H	0.00000000	-0.00000000	-0.00000000
H	-0.82262775	-2.42026635	-0.00000000
O	7.83461905	0.55738136	-0.00000000
N	7.14844079	-4.00206529	-0.00000000
N	7.46163154	-1.71656440	-0.00000000
C	9.41590662	-3.17447096	-0.00000000
C	8.27003082	-0.62421145	-0.00000000
N	9.65603357	-0.82777239	-0.00000000
C	10.21432266	-2.07245899	-0.00000000
C	7.99620203	-2.96060279	-0.00000000
H	9.83388744	-4.17428544	-0.00000000
H	10.24447549	0.00000000	-0.00000000
H	11.29744761	-2.11566317	-0.00000000
H	6.12873984	-3.85217674	-0.00000000
H	7.51095075	-4.94658058	-0.00000000

Cartesian Coordinates and Total Bonding Energies of Ds variants and Px in Water

Cartesian coordinates (Å) and total bonding energies (kcal mol^{-1}) of Ds variants and Px optimized at ZORA-BLYP-D3(BJ)/TZ2P level of theory in water.

Ds

Total bonding energy = -3175.81 kcal mol^{-1}

H	6.88876363	-0.60317674	0.00000000
H	1.81957063	3.43185413	0.00000000
H	6.43885933	1.90335582	0.00000000
C	2.13400825	-2.52429134	0.00000000
C	1.13029861	-3.53200543	0.00000000
C	-0.14638484	-3.01442363	0.00000000
S	-0.14048493	-1.28262934	0.00000000
C	1.62329642	-1.23279592	0.00000000
H	0.59183689	1.31791002	0.00000000
H	3.19973037	-2.71796784	0.00000000
H	1.34094313	-4.59719408	0.00000000
H	-1.09059243	-3.54442549	0.00000000
C	1.67767832	1.28183132	0.00000000
C	2.37471036	2.49572644	0.00000000
N	3.72434515	2.60481188	0.00000000
C	4.34672818	1.42126731	0.00000000
C	3.75530627	0.12555820	0.00000000
C	2.34195529	0.03323778	0.00000000
N	5.71372977	1.19367830	0.00000000
C	5.89869135	-0.16573108	0.00000000
N	4.75839722	-0.84684522	0.00000000

Bs

Total bonding energy = -3276.76 kcal mol^{-1}

H	6.87939419	-0.68464422	0.00000000
H	1.70852416	3.44487365	0.00000000
H	6.51539302	1.82688956	0.00000000
C	2.14698916	-2.50663178	0.00000000
C	1.14476426	-3.52002964	0.00000000
C	-0.13293547	-3.01071266	0.00000000
S	-0.12937213	-1.27516940	0.00000000
C	1.63684230	-1.21619105	0.00000000
H	0.56890324	1.28489709	0.00000000
H	3.21260375	-2.69884106	0.00000000
H	1.36167431	-4.58426565	0.00000000
H	-1.07597498	-3.54275128	0.00000000
C	1.65609499	1.29219733	0.00000000
C	2.30819127	2.53806895	0.00000000
C	3.70189662	2.63517697	0.00000000
C	4.40189508	1.42616691	0.00000000
C	3.76636077	0.15077462	0.00000000
C	2.34690598	0.06212700	0.00000000
N	5.76211155	1.14834163	0.00000000
C	5.90440421	-0.21413071	0.00000000
N	4.74204694	-0.85484007	0.00000000

H	4.21070957	3.59506153	0.00000000

Is

Total bonding energy = -3371.36 kcal mol^{-1}

H	7.07346627	-0.50007977	0.00000000
H	1.69292875	3.40641330	0.00000000
H	6.46838917	1.94603875	0.00000000
C	2.07806441	-2.55102804	0.00000000
C	1.04171736	-3.53010357	0.00000000
C	-0.21659083	-2.97772284	0.00000000
S	-0.14966537	-1.24410601	0.00000000
C	1.62146653	-1.24024803	0.00000000
H	0.57023657	1.23723184	0.00000000
H	3.12464111	-2.81959585	0.00000000
H	1.22503396	-4.60057007	0.00000000
H	-1.17751489	-3.47668120	0.00000000
C	1.65732641	1.25103858	0.00000000
C	2.29737868	2.50273821	0.00000000
C	3.68874994	2.59976217	0.00000000
C	4.40603755	1.39992610	0.00000000
C	3.78583018	0.10225397	0.00000000
C	2.35760522	0.02793741	0.00000000
N	5.77627257	1.20699901	0.00000000
C	6.05191536	-0.14497934	0.00000000
C	4.86630062	-0.85396743	0.00000000
H	4.19544933	3.56165688	0.00000000
H	4.81062177	-1.93097000	0.00000000

Ys

Total bonding energy = -3271.71 kcal mol^{-1}

H	7.06878366	-0.42519132	0.00000000
H	1.80432136	3.39351771	0.00000000
H	6.38762472	2.01018221	0.00000000
C	2.06500936	-2.56753686	0.00000000
C	1.02807836	-3.54194951	0.00000000
C	-0.22933150	-2.98262526	0.00000000
S	-0.16141566	-1.25267319	0.00000000
C	1.60620455	-1.25707076	0.00000000
H	0.58949869	1.27057023	0.00000000
H	3.11295531	-2.83269231	0.00000000
H	1.20589870	-4.61302547	0.00000000
H	-1.19109970	-3.47997911	0.00000000
C	1.67543573	1.24053875	0.00000000
C	2.36293749	2.45909149	0.00000000
N	3.70949036	2.56794739	0.00000000
C	4.35140640	1.39269002	0.00000000
C	3.77076484	0.07554557	0.00000000
C	2.35001501	-0.00022171	0.00000000
N	5.72332191	1.24488763	0.00000000
C	6.03715634	-0.10155873	0.00000000
C	4.87498991	-0.84801230	0.00000000
H	4.84908346	-1.92668198	0.00000000

Yi

Total bonding energy = -3363.35 kcal mol^{-1}

H	7.01335709	-0.47567052	0.00000000
H	1.72695729	3.31834166	0.00000000
H	6.33125969	1.95780285	0.00000000
N	2.16568125	-2.56237696	0.00000000
C	1.11935199	-3.46527751	0.00000000
C	-0.09074332	-2.79672949	0.00000000
N	0.23143660	-1.45870938	0.00000000
C	1.60572780	-1.33977896	0.00000000
H	0.52510601	1.19092000	0.00000000
H	-0.43908843	-0.69867132	0.00000000
H	1.28942264	-4.53438197	0.00000000
H	-1.11490949	-3.14029972	0.00000000
C	1.61065096	1.16386709	0.00000000
C	2.29245031	2.38821830	0.00000000
N	3.63827733	2.50828174	0.00000000
C	4.29399499	1.34028510	0.00000000
C	3.72101134	0.02219710	0.00000000
C	2.30642297	-0.06231394	0.00000000
N	5.66578362	1.19357712	0.00000000
C	5.98011471	-0.15654152	0.00000000
C	4.81839365	-0.90433965	0.00000000
H	4.75132376	-1.98161921	0.00000000

Do

Total bonding energy = -3234.14 kcal mol^{-1}

H	0.16764396	4.14858705	0.00000000
H	-3.71316771	-1.04219248	0.00000000
H	-2.32354461	3.62940302	0.00000000
C	2.24904692	-0.61615272	0.00000000
C	3.09615328	-1.76937061	0.00000000
C	2.27038532	-2.86053616	0.00000000
O	0.95217587	-2.46321176	0.00000000
C	0.94612290	-1.06742834	0.00000000
H	-1.54993785	-2.20691442	0.00000000
H	2.54729954	0.42208537	0.00000000
H	4.17819000	-1.78613127	0.00000000
H	2.43850804	-3.92773284	0.00000000
C	-1.55967797	-1.12205409	0.00000000
C	-2.79390892	-0.45932360	0.00000000
N	-2.94559414	0.88644523	0.00000000
C	-1.78365414	1.54928297	0.00000000
C	-0.47227909	0.99889402	0.00000000
C	-0.33926903	-0.40868667	0.00000000
N	-1.59559279	2.92269047	0.00000000
C	-0.24151111	3.14650307	0.00000000
N	0.47261150	2.02584382	0.00000000

Dp

Total bonding energy = -3438.10 kcal mol^{-1}

H	-0.05629802	4.17572296	0.00000000
H	-3.92168457	-1.02091319	0.00000000
H	-2.54440287	3.64183759	0.00000000
N	1.80786539	-3.20054738	0.00000000
C	3.00501743	-2.59059275	0.00000000
C	3.14626249	-1.19952962	0.00000000
C	1.99258837	-0.43579996	0.00000000
H	4.13151833	-0.74223131	0.00000000
H	-1.74746630	-2.18094601	0.00000000
H	2.01911176	0.64709933	0.00000000
H	3.86656567	-3.25438086	0.00000000
C	0.75334632	-1.10977755	0.00000000
C	-1.76549363	-1.09731079	0.00000000
C	-3.00198890	-0.43922681	0.00000000
N	-3.14927916	0.90587407	0.00000000
C	-1.99142408	1.56742312	0.00000000
C	-0.67264363	1.01684272	0.00000000
C	-0.54593033	-0.39050303	0.00000000
N	-1.81124582	2.94044334	0.00000000
C	-0.46059307	3.17169560	0.00000000
N	0.26089947	2.05682244	0.00000000
N	0.68527509	-2.46200193	0.00000000

Dss

Total bonding energy = -4208.16 kcal mol^{-1}

H	-0.51372605	6.88848559	0.00000000
H	3.40393227	1.72640722	0.00000000
H	1.98074623	6.38213936	0.00000000
C	-2.54246168	2.17668624	0.00000000
C	-3.56834180	1.20510812	0.00000000
C	-3.09968497	-0.10389446	0.00000000
S	-1.34350075	-0.12377155	0.00000000
C	-1.26023213	1.64031703	0.00000000
H	1.26308506	0.54736747	0.00000000
H	-2.71372380	3.24623936	0.00000000
H	-4.62613562	1.44962250	0.00000000
C	-3.85046813	-1.33642952	0.00000000
C	1.25190668	1.63380588	0.00000000
C	2.48073278	2.30278268	0.00000000
N	2.62105955	3.65008803	0.00000000
C	1.45179217	4.29986110	0.00000000
C	0.14308862	3.73912862	0.00000000
C	0.01728775	2.32704765	0.00000000
N	1.25608279	5.67206459	0.00000000
C	-0.09864625	5.88889303	0.00000000
N	-0.80564682	4.76419836	0.00000000
C	-3.38460866	-2.64424436	0.00000000
C	-4.42379177	-3.61666589	0.00000000
C	-5.68150167	-3.05627851	0.00000000
S	-5.61332172	-1.32073579	0.00000000
H	-6.64390097	-3.55199983	0.00000000
H	-2.32766449	-2.89270895	0.00000000

H	-4.24930877	-4.68826009	0.00000000

Dt

Total bonding energy = -3075.29 kcal mol^{-1}

H	-0.59308317	6.86298661	0.00000000
H	3.43166023	1.78777525	0.00000000
H	1.91052480	6.40575150	0.00000000
N	-2.43334305	2.10526241	0.00000000
C	-3.41297316	1.13958001	0.00000000
C	-2.96789361	-0.16053528	0.00000000
S	-1.24228979	-0.20113420	0.00000000
C	-1.22209591	1.57350973	0.00000000
H	1.31525924	0.56180270	0.00000000
N	-0.84697672	4.73505747	0.00000000
H	-4.45683352	1.43715304	0.00000000
H	-3.54750408	-1.07442085	0.00000000
C	1.28085974	1.64738836	0.00000000
C	2.49532298	2.34195182	0.00000000
N	2.59983481	3.69124392	0.00000000
C	1.42032119	4.31569205	0.00000000
C	0.11786272	3.72773324	0.00000000
C	0.03709976	2.31593262	0.00000000
N	1.19922853	5.68219121	0.00000000
C	-0.15887482	5.87142922	0.00000000

Yo

Total bonding energy = -3330.51 kcal mol^{-1}

H	0.00471033	4.31064923	0.00000000
H	-3.67971454	-1.05249413	0.00000000
H	-2.41359512	3.57381523	0.00000000
C	2.28564040	-0.68984206	0.00000000
C	3.09654288	-1.86929877	0.00000000
C	2.23689645	-2.93251839	0.00000000
O	0.93298671	-2.49185688	0.00000000
C	0.96694813	-1.09516782	0.00000000
H	-1.51068214	-2.21385670	0.00000000
H	2.64455287	0.32793332	0.00000000
H	4.17756955	-1.91816136	0.00000000
H	2.37056903	-4.00455450	0.00000000
C	-1.52388904	-1.12924271	0.00000000
C	-2.75975008	-0.47012616	0.00000000
N	-2.90589530	0.87331217	0.00000000
C	-1.74987582	1.55084430	0.00000000
C	-0.42085158	1.00570464	0.00000000
C	-0.30944618	-0.40990837	0.00000000
N	-1.63401850	2.92637934	0.00000000
C	-0.29411325	3.27154125	0.00000000
C	0.47938865	2.12647908	0.00000000
H	1.55908982	2.11596402	0.00000000

Ps

Total bonding energy = -3075.09 kcal mol^{-1}

H	6.94959371	-0.53589536	0.00000000
H	1.71280873	3.25122373	0.00000000
H	6.43090017	1.95955190	0.00000000
C	2.13078776	-2.53245175	0.00000000
C	1.09196931	-3.50091909	0.00000000
C	-0.16491108	-2.93132348	0.00000000
S	-0.10263305	-1.20166741	0.00000000
C	1.65708015	-1.22689665	0.00000000
N	4.82833835	-0.84050494	0.00000000
H	3.18942961	-2.76394933	0.00000000
H	1.26181184	-4.57320806	0.00000000
H	-1.12607460	-3.43045663	0.00000000
N	1.68710607	1.18330214	0.00000000
C	2.33724810	2.35910371	0.00000000
N	3.67069994	2.56522981	0.00000000
C	4.35823130	1.41529551	0.00000000
C	3.80454431	0.10841521	0.00000000
C	2.39660388	0.01791030	0.00000000
N	5.72422561	1.23113133	0.00000000
C	5.94721982	-0.12804287	0.00000000

Es

Total bonding energy = -3172.59 kcal mol^{-1}

H	-0.35159523	7.07357157	0.00000000
H	3.20551970	1.64368278	0.00000000
H	2.06432500	6.32341863	0.00000000
C	-2.57885274	2.02949332	0.00000000
C	-3.51833795	0.96289753	0.00000000
C	-2.91370421	-0.27609136	0.00000000
S	-1.18637731	-0.16026320	0.00000000
C	-1.25905940	1.59923116	0.00000000
C	-0.84046590	4.89414060	0.00000000
H	-2.86903432	3.07197808	0.00000000
H	-4.59469288	1.10465153	0.00000000
H	-3.38517521	-1.25112387	0.00000000
N	1.13495310	1.63277144	0.00000000
C	2.31659826	2.27304465	0.00000000
N	2.52509526	3.60285167	0.00000000
C	1.38629928	4.31167750	0.00000000
C	0.05739421	3.77057788	0.00000000
C	-0.01938022	2.35747963	0.00000000
N	1.28172559	5.67902986	0.00000000
C	-0.06115463	6.03235452	0.00000000
H	-1.91992772	4.89052816	0.00000000

Ysm

Total bonding energy = -3640.41 kcal mol^{-1}

H	3.02778106	2.64650426	0.00000000
H	1.43672112	-3.66082077	0.00000000
H	4.06697213	0.34032774	0.00000000
C	-2.19000796	1.07828077	0.00000000
C	-3.60869667	1.15830174	0.00000000

C	-4.23425868	-0.07157359	0.00000000
S	-3.05210615	-1.35355849	0.00000000
C	-1.69598647	-0.21936241	0.00000000
H	-0.85639468	-2.81079332	0.00000000
H	-1.55848805	1.95607319	0.00000000
H	-4.15632927	2.09688882	0.00000000
H	0.36124104	2.37331674	0.00000000
C	-0.04221112	-2.09144070	0.00000000
C	1.26654329	-2.58543934	0.00000000
N	2.37033144	-1.80599872	0.00000000
C	2.10847305	-0.49184554	0.00000000
C	0.81899321	0.14590344	0.00000000
C	-0.32158779	-0.70599671	0.00000000
N	3.06654844	0.50166775	0.00000000
C	2.44400229	1.73642784	0.00000000
C	1.07378817	1.56302793	0.00000000
C	-5.70601731	-0.37848292	0.00000000
H	-6.19084166	0.05059707	0.88561925
H	-5.89387787	-1.45624245	0.00000000
H	-6.19084166	0.05059707	-0.88561925

Yss

Total bonding energy = -4304.03 kcal mol^{-1}

H	-0.27599868	7.07774298	0.00000000
H	3.33685026	1.66905656	0.00000000
H	2.13070069	6.30302376	0.00000000
C	-2.60856934	2.15704222	0.00000000
C	-3.61784363	1.16678729	0.00000000
C	-3.12629180	-0.13194485	0.00000000
S	-1.37075746	-0.11652982	0.00000000
C	-1.31406262	1.65203943	0.00000000
H	1.16988689	0.53622151	0.00000000
H	-2.83494461	3.21407587	0.00000000
H	-4.67920057	1.39505239	0.00000000
C	-3.85340047	-1.37912142	0.00000000
C	1.18134933	1.62260188	0.00000000
C	2.42457270	2.26311127	0.00000000
N	2.58545204	3.60480838	0.00000000
C	1.43539377	4.29170146	0.00000000
C	0.09706191	3.76269605	0.00000000
C	-0.03415031	2.34460026	0.00000000
N	1.34062969	5.66846429	0.00000000
C	0.00694849	6.03425060	0.00000000
C	-0.78320943	4.90091152	0.00000000
C	-3.36219152	-2.67756171	0.00000000
C	-4.38257765	-3.67026280	0.00000000
C	-5.65097772	-3.13470683	0.00000000
S	-5.61586635	-1.39740022	0.00000000
H	-2.30059406	-2.90522454	0.00000000
H	-4.18747221	-4.73836914	0.00000000
H	-6.60356042	-3.64893451	0.00000000
H	-1.86215345	4.91683218	0.00000000

Px

Total bonding energy = -2448.45 kcal mol^{-1}

C	-1.36664376	-3.84013539	0.00000000
H	-0.99239393	-4.36669075	0.88731795
H	-2.46125376	-3.91306676	0.00000000
H	-0.99239393	-4.36669075	-0.88731795
C	-1.01923169	1.20155805	0.00000000
C	-0.16572394	0.07020231	0.00000000
C	1.16735927	0.55826219	0.00000000
C	1.07955386	1.95162935	0.00000000
N	-0.25346565	2.31735169	0.00000000
O	3.29201854	2.54301966	0.00000000
H	2.07653325	-0.02414642	0.00000000
H	-0.58177513	3.27758874	0.00000000
H	-2.09812534	1.25465602	0.00000000
N	2.08874456	2.92240516	0.00000000
O	1.75064346	4.14233320	0.00000000
C	-0.58429667	-1.28337580	0.00000000
C	-0.93954921	-2.44490051	0.00000000

Cartesian Coordinates and Total Bonding Energies of Ds–Px variants in Water

Cartesian coordinates (Å) and total bonding energies (kcal mol^{-1}) of Ds–Px variants optimized at ZORA-BLYP-D3(BJ)/TZ2P level of theory in water.

Ds–Px

Total bonding energy = -5626.89 kcal mol^{-1}

H	6.91653187	-0.94310660	0.00000000
H	1.96645627	3.23930712	0.00000000
H	6.54271447	1.57496647	0.00000000
C	2.10690861	-2.72312215	0.00000000
C	1.07263990	-3.69950227	0.00000000
C	-0.18747873	-3.14407029	0.00000000
S	-0.12821480	-1.41373383	0.00000000
C	1.63603058	-1.41698859	0.00000000
H	0.68359557	1.17009316	0.00000000
H	3.16577671	-2.95090695	0.00000000
H	1.25029701	-4.77050777	0.00000000
H	-1.14447981	-3.64806773	0.00000000
C	1.76586979	1.09598578	0.00000000
C	2.49644983	2.28942907	0.00000000
N	3.84869616	2.35786286	0.00000000
C	4.43580435	1.15653938	0.00000000
C	3.80633381	-0.12150407	0.00000000
C	2.39103013	-0.17219729	0.00000000
N	5.79547799	0.88866023	0.00000000
C	5.94025912	-0.47575055	0.00000000
N	4.78034604	-1.12279504	0.00000000
C	-4.72933434	1.73519924	0.00000000
C	-3.67013283	0.79322804	0.00000000
C	-2.46115988	1.53487996	0.00000000
C	-2.81804986	2.88287152	0.00000000
N	-4.19703272	2.98039040	0.00000000
O	-0.75796458	3.87934757	0.00000000
H	-1.45860173	1.14204683	0.00000000
H	-4.70801910	3.85709352	0.00000000
H	-5.79767649	1.57578053	0.00000000
N	-2.01042085	4.02765653	0.00000000
O	-2.57317326	5.16166019	0.00000000
C	-3.80683062	-0.61670978	0.00000000
C	-3.90976099	-1.82682029	0.00000000
C	-4.03400485	-3.28014579	0.00000000
H	-3.56034213	-3.71701390	0.88859404
H	-5.08819853	-3.58304161	0.00000000
H	-3.56034213	-3.71701390	-0.88859404

Bs–Px

Total bonding energy = -5727.96 kcal mol^{-1}

H	-1.05077268	6.81158940	0.00000000
H	3.19739353	1.73602256	0.00000000
H	1.46902524	6.50671849	0.00000000
C	-2.76146713	2.03715525	0.00000000

C	-3.74987323	1.01038122	0.00000000
C	-3.21037990	-0.25454504	0.00000000
S	-1.47564802	-0.20839645	0.00000000
C	-1.45910779	1.55853130	0.00000000
H	1.07225305	0.55321202	0.00000000
H	-2.97984874	3.09767913	0.00000000
H	-4.81909762	1.20061067	0.00000000
H	-3.72252317	-1.20723369	0.00000000
C	1.04899638	1.63870280	0.00000000
C	2.27965767	2.31766983	0.00000000
C	2.34355442	3.71338956	0.00000000
C	1.11815979	4.38453231	0.00000000
C	-0.14224067	3.71932930	0.00000000
C	-0.19777607	2.29825179	0.00000000
N	0.80811224	5.73792130	0.00000000
C	-0.55722214	5.84807425	0.00000000
N	-1.17034216	4.67093812	0.00000000
C	1.57986673	-4.87993781	0.00000000
C	0.66136682	-3.80034432	0.00000000
C	1.42929429	-2.60738251	0.00000000
C	2.76917690	-2.99446607	0.00000000
N	2.83631814	-4.37538517	0.00000000
O	3.81343829	-0.95821334	0.00000000
H	1.05973183	-1.59549489	0.00000000
H	3.70223779	-4.90452905	0.00000000
H	1.39646698	-5.94444350	0.00000000
N	3.93221347	-2.21374876	0.00000000
O	5.05294406	-2.80301480	0.00000000
C	-0.75127086	-3.90780305	0.00000000
C	-1.96291066	-3.98936352	0.00000000
C	-3.41798475	-4.08853830	0.00000000
H	-3.84715356	-3.60748262	0.88843669
H	3.29111884	4.24467645	0.00000000
H	-3.84715356	-3.60748262	-0.88843669
H	-3.73855390	-5.13758020	0.00000000

Is–Px

Total bonding energy = -5822.59 kcal mol^{-1}

H	-0.78492173	6.91411248	0.00000000
H	3.20663290	1.59345812	0.00000000
H	1.67128632	6.34943376	0.00000000
C	-2.75471652	1.88309217	0.00000000
C	-3.71461268	0.82886718	0.00000000
C	-3.14037774	-0.41912794	0.00000000
S	-1.40843880	-0.32035964	0.00000000
C	-1.43595329	1.45051121	0.00000000
H	1.06294401	0.44357915	0.00000000
H	-3.04246842	2.92456891	0.00000000
H	-4.78819545	0.99206244	0.00000000
H	-3.62435867	-1.38626788	0.00000000
C	1.05498903	1.52923298	0.00000000
C	2.29649414	2.18706883	0.00000000

C	2.37078368	3.58003817	0.00000000
C	1.15923621	4.27804303	0.00000000
C	-0.12853775	3.63677492	0.00000000
C	-0.18016885	2.20758564	0.00000000
N	0.94374515	5.64527439	0.00000000
C	-0.41265362	5.89864453	0.00000000
C	-1.10214412	4.70148247	0.00000000
C	1.63670106	-5.00217191	0.00000000
C	0.71612312	-3.92423459	0.00000000
C	1.48190864	-2.72982524	0.00000000
C	2.82274092	-3.11470407	0.00000000
N	2.89206879	-4.49554388	0.00000000
O	3.86491649	-1.07744903	0.00000000
H	1.11185710	-1.71789992	0.00000000
H	3.75848782	-5.02383939	0.00000000
H	1.45494339	-6.06697057	0.00000000
N	3.98480744	-2.33286797	0.00000000
O	5.10619158	-2.92107748	0.00000000
C	-0.69617004	-4.03638395	0.00000000
C	-1.90714593	-4.12840506	0.00000000
C	-3.36112189	-4.24367922	0.00000000
H	3.32441692	4.10204584	0.00000000
H	-3.79631891	-3.76795646	-0.88828124
H	-3.79631891	-3.76795646	0.88828124
H	-2.17800426	4.62752789	0.00000000
H	-3.66864717	-5.29668352	0.00000000

Ys–Px

Total bonding energy = -5722.81 kcal mol^{-1}

H	-0.62480155	7.00741017	0.00000000
H	3.27696228	1.80219780	0.00000000
H	1.82143991	6.36581472	0.00000000
C	-2.68693536	1.97008134	0.00000000
C	-3.64414783	0.91702794	0.00000000
C	-3.06537036	-0.33094531	0.00000000
S	-1.33688804	-0.23438854	0.00000000
C	-1.36928503	1.53280404	0.00000000
H	1.18111178	0.56008852	0.00000000
H	-2.96964153	3.01340534	0.00000000
H	-4.71790084	1.07691141	0.00000000
H	-3.54926938	-1.29801834	0.00000000
C	1.12812323	1.64350085	0.00000000
C	2.33609613	2.34862027	0.00000000
N	2.42294744	3.69672637	0.00000000
C	1.23743384	4.31955922	0.00000000
C	-0.07048717	3.71778580	0.00000000
C	-0.12390420	2.29604173	0.00000000
N	1.06723649	5.68901332	0.00000000
C	-0.28434063	5.98111731	0.00000000
C	-1.01176552	4.80675410	0.00000000
C	1.73515893	-4.87821038	0.00000000
C	0.80462046	-3.80891255	0.00000000

C	1.55935799	-2.60778234	0.00000000
C	2.90350009	-2.97970800	0.00000000
N	2.98590235	-4.35975457	0.00000000
O	3.92377546	-0.93134359	0.00000000
H	1.17939875	-1.60006954	0.00000000
H	3.85739022	-4.87965915	0.00000000
H	1.56358289	-5.94469729	0.00000000
N	4.05729475	-2.18550812	0.00000000
O	5.18475874	-2.76159786	0.00000000
C	-0.60663964	-3.93309306	0.00000000
C	-1.81693831	-4.03362026	0.00000000
C	-3.27009317	-4.15806051	0.00000000
H	-3.70776920	-3.68493281	0.88857966
H	-2.08979069	4.76284716	0.00000000
H	-3.70776920	-3.68493281	-0.88857966
H	-3.57235394	-5.21247247	0.00000000

Yi–Px

Total bonding energy = -5813.57 kcal mol^{-1}

H	-6.71699636	-0.86607072	0.00000000
H	-0.47429301	-2.69474842	0.00000000
H	-5.26545651	-2.93440931	0.00000000
N	-2.83932701	2.70732182	0.00000000
C	-2.15847058	3.90911366	0.00000000
C	-0.79524061	3.68683393	0.00000000
N	-0.64518858	2.31722571	0.00000000
C	-1.90213750	1.74433841	0.00000000
H	-0.05032953	-0.27012268	0.00000000
H	-5.08378966	1.30615083	0.00000000
H	-2.67789774	4.85880421	0.00000000
H	0.04696169	4.36126677	0.00000000
C	-1.07812234	-0.61795685	0.00000000
C	-1.31267416	-1.99963936	0.00000000
N	-2.54241960	-2.55870229	0.00000000
C	-3.54899757	-1.67532168	0.00000000
C	-3.44665869	-0.24133727	0.00000000
C	-2.14036571	0.30741498	0.00000000
N	-4.89130210	-1.99265989	0.00000000
C	-5.63642795	-0.82357867	0.00000000
C	-4.78926948	0.26779644	0.00000000
C	4.80901679	-1.16746785	0.00000000
C	3.50243523	-0.61616109	0.00000000
C	2.59729479	-1.70734008	0.00000000
C	3.36955605	-2.86841541	0.00000000
N	4.70611913	-2.51700433	0.00000000
O	1.73872151	-4.47316282	0.00000000
H	1.52125994	-1.66700877	0.00000000
H	5.47170223	-3.18323869	0.00000000
H	5.76840040	-0.67131169	0.00000000
N	2.97267953	-4.21194534	0.00000000
O	3.86936851	-5.10468955	0.00000000
C	3.20066812	0.76826384	0.00000000

C	3.00826359	1.96797576	0.00000000
C	2.82403640	3.41541091	0.00000000
H	2.27490983	3.74667451	-0.89020576
H	0.24379809	1.82596242	0.00000000
H	2.27490983	3.74667451	0.89020576
H	3.79526304	3.92506402	0.00000000

Do–Px

Total bonding energy = -5685.59 kcal mol^{-1}

H	1.09599546	6.93752578	0.00000000
H	-3.49177232	2.35930959	0.00000000
H	-1.44409175	6.78280167	0.00000000
C	2.46803880	1.91641598	0.00000000
C	3.13632015	0.65055753	0.00000000
C	2.15978884	-0.30756618	0.00000000
O	0.91439196	0.28071193	0.00000000
C	1.11289460	1.66266308	0.00000000
H	-1.52901978	0.89799594	0.00000000
H	2.91610048	2.89941160	0.00000000
H	4.20429401	0.47583711	0.00000000
H	2.16206303	-1.38813697	0.00000000
C	-1.37330753	1.97046471	0.00000000
C	-2.49881797	2.80449341	0.00000000
N	-2.45524384	4.15821759	0.00000000
C	-1.20972613	4.64651242	0.00000000
C	0.00855186	3.91277700	0.00000000
C	-0.06347841	2.50075249	0.00000000
N	-0.82552950	5.97858277	0.00000000
C	0.54675682	6.00489357	0.00000000
N	1.09168545	4.79281356	0.00000000
C	-1.42113914	-4.74372247	0.00000000
C	-0.45030252	-3.71124564	0.00000000
C	-1.15526078	-2.47883689	0.00000000
C	-2.51355269	-2.80078249	0.00000000
N	-2.64972224	-4.17688726	0.00000000
O	-3.47130989	-0.72072339	0.00000000
H	-0.73333678	-1.48539487	0.00000000
H	-3.54168114	-4.66079767	0.00000000
H	-1.29047172	-5.81596090	0.00000000
N	-3.64160881	-1.97020964	0.00000000
O	-4.78677353	-2.51098981	0.00000000
C	0.95432739	-3.89575441	0.00000000
C	2.16029141	-4.04110163	0.00000000
C	3.61052026	-4.19874329	0.00000000
H	4.05770374	-3.73306775	-0.88772757
H	4.05770374	-3.73306775	0.88772757
H	3.88871833	-5.25974872	0.00000000

Dp–Px

Total bonding energy = -5887.77 kcal mol^{-1}

H	0.89090346	6.98162934	0.00000000
H	-3.62916826	2.34232988	0.00000000

H	-1.64549835	6.78277414	0.00000000
C	0.98614149	1.63064594	0.00000000
N	0.72822227	0.30084228	0.00000000
H	3.94944359	-3.99241763	0.88716398
H	3.94944359	-3.99241763	-0.88716398
N	1.73113625	-0.59030418	0.00000000
H	-1.63270056	0.90621945	0.00000000
C	3.00204518	-0.15610620	0.00000000
C	3.34148837	1.20056922	0.00000000
C	2.30849293	2.12174114	0.00000000
C	-1.50303369	1.98111558	0.00000000
C	-2.64083282	2.79722264	0.00000000
N	-2.60894207	4.15055761	0.00000000
C	-1.37330225	4.65266410	0.00000000
C	-0.13941190	3.93210592	0.00000000
C	-0.20095746	2.52073402	0.00000000
N	-1.01241414	5.98988643	0.00000000
C	0.35720938	6.03999177	0.00000000
N	0.92439724	4.83914083	0.00000000
C	-1.55616066	-4.84473659	0.00000000
C	-0.54959224	-3.84680089	0.00000000
C	-1.20942872	-2.58786024	0.00000000
C	-2.57940688	-2.86478234	0.00000000
N	-2.76405562	-4.23616293	0.00000000
O	-3.48678079	-0.75913697	0.00000000
H	-0.74695870	-1.60937596	0.00000000
H	-3.67289315	-4.68742962	0.00000000
H	-1.46333933	-5.92108014	0.00000000
N	-3.68450241	-2.00477717	0.00000000
O	-4.84442193	-2.51676640	0.00000000
C	0.84670236	-4.08663173	0.00000000
C	2.04722376	-4.26714419	0.00000000
C	3.49431381	-4.45183293	0.00000000
H	4.38204545	1.51262837	0.00000000
H	2.48835770	3.19004207	0.00000000
H	3.75964947	-0.93659993	0.00000000
H	3.75721176	-5.51693418	0.00000000

Dss–Px
Total bonding energy = -6659.13 kcal mol^{-1}

H	-7.28649966	3.00579581	0.00000000
H	-1.02324845	4.67473178	0.00000000
H	-5.87325188	5.12306376	0.00000000
C	-3.69144462	-0.65272678	0.00000000
C	-3.18302044	-1.97040324	0.00000000
C	-1.79428782	-2.03626359	0.00000000
S	-1.10738436	-0.41884205	0.00000000
C	-2.70890065	0.32952196	0.00000000
H	-0.74330076	2.25388971	0.00000000
H	-4.74595896	-0.40541789	0.00000000
H	-3.81255646	-2.85488011	0.00000000
H	-0.02486721	-6.64952932	0.00000000

C	-1.75401023	2.65039449	0.00000000
C	-1.90806943	4.04145812	0.00000000
N	-3.10213041	4.68147596	0.00000000
C	-4.14626124	3.84507150	0.00000000
C	-4.12268787	2.42149063	0.00000000
C	-2.86252442	1.77157976	0.00000000
N	-5.49055324	4.18339069	0.00000000
C	-6.20413544	3.01162291	0.00000000
N	-5.43071640	1.93148450	0.00000000
C	5.28602158	0.90230444	0.00000000
C	4.17986654	0.01512795	0.00000000
C	3.00759866	0.81567550	0.00000000
C	3.43714195	2.14503395	0.00000000
N	4.81871633	2.17133046	0.00000000
O	1.44523851	3.27447520	0.00000000
H	1.98126219	0.48024504	0.00000000
H	5.37231808	3.02192634	0.00000000
H	6.34423780	0.68597476	0.00000000
N	2.70343219	3.33865700	0.00000000
O	3.33904583	4.43437005	0.00000000
C	4.27941435	-1.39794898	0.00000000
C	4.42754615	-2.60347224	0.00000000
C	4.63174369	-4.04731392	0.00000000
C	-0.95001131	-3.20589245	0.00000000
C	0.43561525	-3.29660467	0.00000000
C	0.91324437	-4.63709100	0.00000000
C	-0.09909005	-5.56956455	0.00000000
S	-1.66368641	-4.81902723	0.00000000
H	4.18580443	-4.51250560	-0.88825653
H	4.18580443	-4.51250560	0.88825653
H	1.08851913	-2.42905635	0.00000000
H	1.96341129	-4.90523877	0.00000000
H	5.70261497	-4.28580794	0.00000000

Dt–Px

Total bonding energy = -5526.16 kcal mol^{-1}

H	-0.97105773	7.00744595	0.00000000
H	3.13751808	1.99838784	0.00000000
H	1.54063841	6.59208465	0.00000000
N	-2.73152632	2.21680344	0.00000000
C	-3.69280628	1.23260425	0.00000000
C	-3.22402789	-0.05885178	0.00000000
S	-1.49901081	-0.06766377	0.00000000
C	-1.51092190	1.70844874	0.00000000
H	1.04921408	0.74379117	0.00000000
H	-3.81017752	-3.41061687	-0.88865412
H	-4.74240605	1.50830081	0.00000000
H	-3.79077913	-0.97955837	0.00000000
C	0.99106724	1.82671815	0.00000000
C	2.19436400	2.53954637	0.00000000
N	2.27560405	3.89039910	0.00000000
C	1.08549190	4.49459433	0.00000000

C	-0.20731912	3.88490068	0.00000000
C	-0.26476624	2.47172485	0.00000000
N	0.84110553	5.85712617	0.00000000
C	-0.52009163	6.02346787	0.00000000
N	-1.18875055	4.87534910	0.00000000
C	1.62501291	-4.66432855	0.00000000
C	0.69667516	-3.59316581	0.00000000
C	1.45401478	-2.39399156	0.00000000
C	2.79720705	-2.76802460	0.00000000
N	2.87694362	-4.14821170	0.00000000
O	3.81914960	-0.72049829	0.00000000
H	1.07619660	-1.38604112	0.00000000
H	3.74747398	-4.66972927	0.00000000
H	1.45148939	-5.73047163	0.00000000
N	3.95184351	-1.97480325	0.00000000
O	5.07867623	-2.55166957	0.00000000
C	-0.71497569	-3.71087463	0.00000000
C	-1.92664440	-3.79388920	0.00000000
C	-3.38177174	-3.89181932	0.00000000
H	-3.81017752	-3.41061687	0.88865412
H	-3.70374425	-4.94039209	0.00000000

Yo–Px

Total bonding energy = -5781.91 kcal mol^{-1}

H	0.87827884	7.02778574	0.00000000
H	-3.52331804	2.23567923	0.00000000
H	-1.61975986	6.63899629	0.00000000
C	2.43304894	1.75854322	0.00000000
C	3.07145668	0.47686608	0.00000000
C	2.07287863	-0.45651363	0.00000000
O	0.84312632	0.16368939	0.00000000
C	1.07063071	1.54252274	0.00000000
H	-1.54958794	0.78268489	0.00000000
H	2.93055375	2.71625623	0.00000000
H	4.13528037	0.27869307	0.00000000
H	2.04956198	-1.53689761	0.00000000
C	-1.40085133	1.85609256	0.00000000
C	-2.53112596	2.68337876	0.00000000
N	-2.48757487	4.03416653	0.00000000
C	-1.24771431	4.54237523	0.00000000
C	-0.00899803	3.81551213	0.00000000
C	-0.09802219	2.39831719	0.00000000
N	-0.93927270	5.88810030	0.00000000
C	0.43623359	6.04100737	0.00000000
C	1.04033110	4.79801747	0.00000000
C	-1.46891301	-4.88369483	0.00000000
C	-0.49471208	-3.85445421	0.00000000
C	-1.19568988	-2.61967933	0.00000000
C	-2.55483435	-2.93727207	0.00000000
N	-2.69575854	-4.31286313	0.00000000
O	-3.50258082	-0.85309416	0.00000000
H	-0.77235832	-1.62678362	0.00000000

H	-3.58928791	-4.79387123	0.00000000
H	-1.34165750	-5.95635203	0.00000000
N	-3.67903065	-2.10159237	0.00000000
O	-4.82664847	-2.63729751	0.00000000
C	0.90934017	-4.04301772	0.00000000
C	2.11512805	-4.18882668	0.00000000
C	3.56541463	-4.34568814	0.00000000
H	4.01228702	-3.87964297	0.88770667
H	4.01228702	-3.87964297	-0.88770667
H	2.10774859	4.63507916	0.00000000
H	3.84411022	-5.40657950	0.00000000

Ps–Px

Total bonding energy = -5526.77 kcal mol^{-1}

H	-0.48454746	6.83071146	0.00000000
H	2.81069306	1.26868603	0.00000000
H	1.95271870	6.09074596	0.00000000
C	-2.90934883	2.19596332	0.00000000
C	-3.95605868	1.23542473	0.00000000
C	-3.48622330	-0.06179172	0.00000000
S	-1.75542718	-0.13519894	0.00000000
C	-1.64648050	1.61932110	0.00000000
H	-3.55925540	-3.34122221	-0.88860261
H	-3.05794696	3.26941614	0.00000000
H	-5.01194214	1.48775126	0.00000000
H	-4.06394303	-0.97698767	0.00000000
N	0.75291997	1.43301455	0.00000000
C	1.98291890	1.97328011	0.00000000
N	2.30770839	3.28457996	0.00000000
C	1.22568935	4.07374390	0.00000000
C	-0.12609532	3.64043681	0.00000000
C	-0.34215274	2.24717810	0.00000000
N	1.16493551	5.45095512	0.00000000
C	-0.16906832	5.79550268	0.00000000
N	-0.97938212	4.74546676	0.00000000
C	1.86149405	-4.48583298	0.00000000
C	0.95400415	-3.39680393	0.00000000
C	1.73050906	-2.20639890	0.00000000
C	3.06858836	-2.61340416	0.00000000
N	3.12090558	-3.99520462	0.00000000
O	4.18017952	-0.60838993	0.00000000
H	1.37067470	-1.18573428	0.00000000
H	3.98224574	-4.53178397	0.00000000
H	1.66527059	-5.54804375	0.00000000
N	4.25393868	-1.86725233	0.00000000
O	5.35640786	-2.49117966	0.00000000
C	-0.45693501	-3.52370095	0.00000000
C	-1.66367656	-3.65828117	0.00000000
C	-3.11442084	-3.80733245	0.00000000
H	-3.55925540	-3.34122221	0.88860261
H	-3.39964223	-4.86641223	0.00000000

Es–Px

Total bonding energy = -5624.27 kcal mol^{-1}

H	-0.26267043	6.87630216	0.00000000
H	2.85274213	1.17849102	0.00000000
H	2.08615317	5.94075831	0.00000000
C	-2.87995331	2.00986991	0.00000000
C	-3.88935891	1.00852521	0.00000000
C	-3.36972108	-0.26816238	0.00000000
S	-1.63700441	-0.27047094	0.00000000
C	-1.59387110	1.48903220	0.00000000
H	-1.99756173	4.82005382	0.00000000
H	-3.09898582	3.06976997	0.00000000
H	-4.95377016	1.22215603	0.00000000
H	-3.91115568	-1.20481528	0.00000000
N	0.79236637	1.33451346	0.00000000
C	2.02112044	1.87861425	0.00000000
N	2.33281906	3.18962671	0.00000000
C	1.25421669	3.98655416	0.00000000
C	-0.11290996	3.55159626	0.00000000
C	-0.30002223	2.14983285	0.00000000
N	1.25646873	5.35826072	0.00000000
C	-0.05536671	5.81537236	0.00000000
C	-0.92089640	4.74142079	0.00000000
C	1.90004152	-4.60947150	0.00000000
C	0.98790913	-3.52401502	0.00000000
C	1.75963148	-2.33063551	0.00000000
C	3.09949651	-2.73235193	0.00000000
N	3.15736926	-4.11383836	0.00000000
O	4.20292498	-0.72302253	0.00000000
H	1.39813045	-1.31060928	0.00000000
H	4.02062496	-4.64727827	0.00000000
H	1.70787393	-5.67244379	0.00000000
N	4.28178174	-1.98147489	0.00000000
O	5.38683752	-2.60111864	0.00000000
C	-0.42227539	-3.66144292	0.00000000
C	-1.62649377	-3.81755329	0.00000000
C	-3.07367307	-3.99920969	0.00000000
H	-3.52966109	-3.54398790	0.88853924
H	-3.52966109	-3.54398790	-0.88853924
H	-3.33349589	-5.06486016	0.00000000

Ysm–Px

Total bonding energy = -6092.30 kcal mol^{-1}

H	0.18093433	7.39605214	0.00000000
H	-3.65469264	2.14183702	0.00000000
H	-2.25672649	6.72371505	0.00000000
C	2.30513575	2.37916419	0.00000000
C	3.27236570	1.33514357	0.00000000
C	2.72688680	0.06973236	0.00000000
S	0.98270131	0.16283791	0.00000000
C	0.99253990	1.93323040	0.00000000
H	-1.54333252	0.92236098	0.00000000

H	2.57902322	3.42518450	0.00000000
H	4.34490829	1.50848593	0.00000000
C	3.42847237	-1.25538490	0.00000000
C	-1.50398159	2.00717011	0.00000000
C	-2.72002415	2.69903896	0.00000000
N	-2.82521331	4.04618115	0.00000000
C	-1.64730351	4.68448926	0.00000000
C	-0.33280613	4.09964342	0.00000000
C	-0.26066158	2.67808527	0.00000000
N	-1.49424545	6.05626775	0.00000000
C	-0.14605528	6.36541379	0.00000000
C	0.59538839	5.19981340	0.00000000
C	-2.03092919	-4.92292437	0.00000000
C	-0.96962971	-3.98313836	0.00000000
C	-1.56095558	-2.69329868	0.00000000
C	-2.94260650	-2.88887645	0.00000000
N	-3.20344957	-4.24661409	0.00000000
O	-3.69970709	-0.72800258	0.00000000
H	-1.05120769	-1.74343492	0.00000000
H	-4.13542612	-4.64814336	0.00000000
H	-1.99905406	-6.00264813	0.00000000
N	-3.98734269	-1.95582234	0.00000000
O	-5.17840297	-2.38656743	0.00000000
C	0.41234715	-4.29556599	0.00000000
C	1.59568352	-4.56868826	0.00000000
C	3.01542049	-4.90298631	0.00000000
H	3.51733075	-4.49638996	-0.88705714
H	3.16575786	-1.84938914	0.88363540
H	4.51176884	-1.09873675	0.00000000
H	3.16575786	-1.84938914	-0.88363540
H	1.67403311	5.16895179	0.00000000
H	3.51733075	-4.49638996	0.88705714
H	3.15998112	-5.99032381	0.00000000

Yss–Px

Total bonding energy = -6754.93 kcal mol^{-1}

H	-7.28609299	3.41418958	0.00000000
H	-0.90990891	4.70523575	0.00000000
H	-5.65689665	5.34762187	0.00000000
C	-3.61209933	-0.60766684	0.00000000
C	-3.07855491	-1.91659681	0.00000000
C	-1.69052597	-1.95536991	0.00000000
S	-1.03886223	-0.32432772	0.00000000
C	-2.65506745	0.39892353	0.00000000
H	-0.68404129	2.27625454	0.00000000
H	-4.67591642	-0.41565117	0.00000000
H	-3.69229344	-2.81209234	0.00000000
C	-0.81910749	-3.10533839	0.00000000
C	-1.68494378	2.69523867	0.00000000
C	-1.80649543	4.08830962	0.00000000
N	-2.98746878	4.74595335	0.00000000
C	-4.05888852	3.94189568	0.00000000

C	-4.07683752	2.50284664	0.00000000
C	-2.81354132	1.84538128	0.00000000
N	-5.36912358	4.37597796	0.00000000
C	-6.21315648	3.28071361	0.00000000
C	-5.46378184	2.11974359	0.00000000
C	5.45347618	0.65910619	0.00000000
C	4.35517003	-0.23752494	0.00000000
C	3.17628645	0.55350053	0.00000000
C	3.59440435	1.88642408	0.00000000
N	4.97577796	1.92420205	0.00000000
O	1.59274880	2.99859721	0.00000000
H	2.15181316	0.21294226	0.00000000
H	5.52192530	2.77957989	0.00000000
H	6.51350013	0.45170530	0.00000000
N	2.85024439	3.07339748	0.00000000
O	3.47678016	4.17442171	0.00000000
C	4.46869551	-1.64981161	0.00000000
C	4.63209590	-2.85339950	0.00000000
C	4.85273741	-4.29557046	0.00000000
C	0.56810597	-3.15974805	0.00000000
C	1.08212934	-4.48653817	0.00000000
C	0.09492614	-5.44553297	0.00000000
S	-1.48987882	-4.73647122	0.00000000
H	1.19690352	-2.27520110	0.00000000
H	4.41255785	-4.76529037	-0.88875568
H	4.41255785	-4.76529037	0.88875568
H	2.13858538	-4.72597904	0.00000000
H	0.19710512	-6.52323424	0.00000000
H	-5.88735111	1.12726084	0.00000000
H	5.92621509	-4.52138846	0.00000000

Cartesian Coordinates and Total Bonding Energies of Ds–Px//A–T variants in Water

Cartesian coordinates (Å) and total bonding energies (kcal mol^{-1}) of Ds–Px//A–T variants optimized at ZORA-BLYP-D3(BJ)-TZ2P level of theory in water.

Ds–Px//A–T

Total bonding energy = -10151.18 kcal mol^{-1}

H	0.72835180	-1.61491941	3.40000000
H	7.15665469	-0.79470301	3.40000000
H	2.83658218	-3.04173268	3.40000000
C	2.66810159	3.13253275	3.40000000
C	2.64781333	4.55472061	3.40000000
C	3.90896866	5.10779465	3.40000000
S	5.14395799	3.89437888	3.40000000
C	3.94889118	2.59653926	3.40000000
H	6.49913093	1.54944726	3.40000000
H	1.78443319	2.50625987	3.40000000
H	1.73850902	5.14783148	3.40000000
H	4.18453588	6.15370439	3.40000000
C	5.71288154	0.80203754	3.40000000
C	6.09841983	-0.54310717	3.40000000
N	5.23469936	-1.58581531	3.40000000
C	3.95256826	-1.20632359	3.40000000
C	3.43633743	0.12150696	3.40000000
C	4.35575875	1.19868788	3.40000000
N	2.83600395	-2.02714987	3.40000000
C	1.73271958	-1.21146491	3.40000000
N	2.04005101	0.08066988	3.40000000
C	10.57480611	5.15611150	3.40000000
C	9.16464152	5.01239854	3.40000000
C	8.89385902	3.62015575	3.40000000
C	10.12843458	2.97186964	3.40000000
N	11.13251700	3.92209180	3.40000000
O	9.47005675	0.78019117	3.40000000
H	7.92663630	3.14695291	3.40000000
H	12.12398134	3.70595778	3.40000000
H	11.17955605	6.05112367	3.40000000
N	10.42603070	1.60284302	3.40000000
O	11.64209002	1.25091364	3.40000000
C	8.21809502	6.06627741	3.40000000
C	7.39597007	6.96018605	3.40000000
C	6.40903286	8.03421887	3.40000000
H	5.76690644	7.98051793	4.28859404
H	6.89849248	9.01579896	3.40000000
H	5.76690644	7.98051793	2.51140596
N	4.59362061	1.42324430	-0.00000000
C	4.11472266	0.16348963	-0.00000000
N	2.83096765	-0.23291518	-0.00000000
C	1.98311285	0.81977333	-0.00000000
C	2.34262891	2.17899152	-0.00000000
C	3.72679637	2.47701379	-0.00000000
N	0.60290236	0.81623453	-0.00000000

C	0.19167759	2.13329993	-0.00000000
N	1.20650731	2.98811796	-0.00000000
N	4.22675705	3.72662446	-0.00000000
H	4.87194754	-0.61925678	-0.00000000
H	0.00000000	-0.00000000	-0.00000000
H	-0.85706123	2.39959278	-0.00000000
H	3.59899438	4.51969988	-0.00000000
H	5.24292768	3.87537936	-0.00000000
N	7.44111734	1.76645429	-0.00000000
C	8.19168180	0.60815965	-0.00000000
N	9.55914485	0.82510648	-0.00000000
C	10.12842843	2.07708710	-0.00000000
C	9.38072615	3.21416744	-0.00000000
C	7.93307577	3.07206955	-0.00000000
O	7.13335774	4.03769629	-0.00000000
O	7.69054235	-0.52818334	-0.00000000
C	9.98429120	4.59244466	-0.00000000
H	6.39589445	1.64161619	-0.00000000
H	10.15048679	-0.00000000	-0.00000000
H	11.21327702	2.08908493	-0.00000000
H	9.66357774	5.16052698	-0.88220861
H	11.07688581	4.53571428	-0.00000000
H	9.66357774	5.16052698	0.88220861

Bs–Px//A–T
Total bonding energy = -10251.75 kcal mol^{-1}

H	0.69490344	1.58059634	-3.40000000
H	7.26230042	0.75743674	-3.40000000
H	2.74759204	3.07351205	-3.40000000
C	2.69400053	-3.08044515	-3.40000000
C	2.66956884	-4.50544151	-3.40000000
C	3.92609607	-5.06421769	-3.40000000
S	5.16487461	-3.84895964	-3.40000000
C	3.97359118	-2.54391135	-3.40000000
H	6.51179803	-1.55600420	-3.40000000
H	1.81180988	-2.45263858	-3.40000000
H	1.75710044	-5.09436379	-3.40000000
H	4.19993745	-6.11060064	-3.40000000
C	5.75547804	-0.77702335	-3.40000000
C	6.19417385	0.55829288	-3.40000000
C	5.29038309	1.62378742	-3.40000000
C	3.93602967	1.28063817	-3.40000000
C	3.46618398	-0.06485408	-3.40000000
C	4.39336743	-1.14322152	-3.40000000
N	2.78725872	2.06045644	-3.40000000
C	1.71250946	1.21122992	-3.40000000
N	2.06527701	-0.06827025	-3.40000000
C	10.58381796	-5.18855736	-3.40000000
C	9.17599765	-5.02361443	-3.40000000
C	8.92580519	-3.62709225	-3.40000000
C	10.17052614	-2.99797474	-3.40000000
N	11.16018558	-3.96338641	-3.40000000

O	9.54833460	-0.79577476	-3.40000000
H	7.96604111	-3.13786237	-3.40000000
H	12.15461291	-3.76108868	-3.40000000
H	11.17452735	-6.09291915	-3.40000000
N	10.49040698	-1.63421162	-3.40000000
O	11.71235964	-1.30239357	-3.40000000
C	8.21481982	-6.06439768	-3.40000000
C	7.38317862	-6.94932481	-3.40000000
C	6.38528599	-8.01294437	-3.40000000
H	5.74340846	-7.95299715	-2.51156331
H	5.62236909	2.65816133	-3.40000000
H	5.74340846	-7.95299715	-4.28843669
H	6.86502507	-8.99940465	-3.40000000
N	4.59362061	-1.42324430	0.00000000
C	4.11472266	-0.16348963	0.00000000
N	2.83096765	0.23291518	0.00000000
C	1.98311285	-0.81977333	0.00000000
C	2.34262891	-2.17899152	0.00000000
C	3.72679637	-2.47701379	0.00000000
N	0.60290236	-0.81623453	0.00000000
C	0.19167759	-2.13329993	0.00000000
N	1.20650731	-2.98811796	0.00000000
N	4.22675705	-3.72662446	0.00000000
H	4.87194754	0.61925678	0.00000000
H	0.00000000	0.00000000	0.00000000
H	-0.85706123	-2.39959278	0.00000000
H	3.59899438	-4.51969988	0.00000000
H	5.24292768	-3.87537936	0.00000000
N	7.44111734	-1.76645429	0.00000000
C	8.19168180	-0.60815965	0.00000000
N	9.55914485	-0.82510648	0.00000000
C	10.12842843	-2.07708710	0.00000000
C	9.38072615	-3.21416744	0.00000000
C	7.93307577	-3.07206955	0.00000000
O	7.13335774	-4.03769629	0.00000000
O	7.69054235	0.52818334	0.00000000
C	9.98429120	-4.59244466	0.00000000
H	6.39589445	-1.64161619	0.00000000
H	10.15048679	0.00000000	0.00000000
H	11.21327702	-2.08908493	0.00000000
H	9.66357774	-5.16052698	0.88220861
H	11.07688581	-4.53571428	0.00000000
H	9.66357774	-5.16052698	-0.88220861

Is–Px//A–T
Total bonding energy = -10346.98 kcal mol^{-1}

H	0.60687107	1.67569398	-3.40000000
H	7.16668714	0.57538297	-3.40000000
H	2.77498112	2.96065268	-3.40000000
C	2.64996964	-3.32600625	-3.40000000
C	2.68187640	-4.75140897	-3.40000000
C	3.95837099	-5.25914822	-3.40000000

S	5.14445999	-3.99322542	-3.40000000
C	3.90328777	-2.72980461	-3.40000000
H	6.40730329	-1.73566872	-3.40000000
H	1.72335636	-2.77025199	-3.40000000
H	1.79188510	-5.37360520	-3.40000000
H	4.27486047	-6.29328149	-3.40000000
C	5.65284215	-0.95496653	-3.40000000
C	6.09822782	0.37759287	-3.40000000
C	5.19139068	1.43755934	-3.40000000
C	3.83266948	1.10750666	-3.40000000
C	3.34235209	-0.24496346	-3.40000000
C	4.29057598	-1.31553304	-3.40000000
N	2.73373423	1.94899302	-3.40000000
C	1.57675123	1.19705927	-3.40000000
C	1.90304906	-0.14537281	-3.40000000
C	10.57835411	-5.28358878	-3.40000000
C	9.16832854	-5.13784760	-3.40000000
C	8.89917894	-3.74479353	-3.40000000
C	10.13558177	-3.09882837	-3.40000000
N	11.13805614	-4.05096810	-3.40000000
O	9.48533062	-0.90481117	-3.40000000
H	7.93334705	-3.26719563	-3.40000000
H	12.12981195	-3.83602976	-3.40000000
H	11.18105038	-6.18002018	-3.40000000
N	10.43792670	-1.73125613	-3.40000000
O	11.65563685	-1.38387336	-3.40000000
C	8.22294254	-6.19302283	-3.40000000
C	7.40946161	-7.09478735	-3.40000000
C	6.43604551	-8.18097119	-3.40000000
H	5.52198207	2.47323192	-3.40000000
H	5.79281966	-8.13659663	-4.28828124
H	5.79281966	-8.13659663	-2.51171876
H	1.17495444	-0.94087425	-3.40000000
H	6.93953201	-9.15559481	-3.40000000
N	4.59362061	-1.42324430	0.00000000
C	4.11472266	-0.16348963	0.00000000
N	2.83096765	0.23291518	0.00000000
C	1.98311285	-0.81977333	0.00000000
C	2.34262891	-2.17899152	0.00000000
C	3.72679637	-2.47701379	0.00000000
N	0.60290236	-0.81623453	0.00000000
C	0.19167759	-2.13329993	0.00000000
N	1.20650731	-2.98811796	0.00000000
N	4.22675705	-3.72662446	0.00000000
H	4.87194754	0.61925678	0.00000000
H	0.00000000	0.00000000	0.00000000
H	-0.85706123	-2.39959278	0.00000000
H	3.59899438	-4.51969988	0.00000000
H	5.24292768	-3.87537936	0.00000000
N	7.44111734	-1.76645429	0.00000000
C	8.19168180	-0.60815965	0.00000000
N	9.55914485	-0.82510648	0.00000000

C	10.12842843	-2.07708710	0.00000000
C	9.38072615	-3.21416744	0.00000000
C	7.93307577	-3.07206955	0.00000000
O	7.13335774	-4.03769629	0.00000000
O	7.69054235	0.52818334	0.00000000
C	9.98429120	-4.59244466	0.00000000
H	6.39589445	-1.64161619	0.00000000
H	10.15048679	0.00000000	0.00000000
H	11.21327702	-2.08908493	0.00000000
H	9.66357774	-5.16052698	0.88220861
H	11.07688581	-4.53571428	0.00000000
H	9.66357774	-5.16052698	-0.88220861

Ys–Px//A–T

Total bonding energy = -10247.75 kcal mol^{-1}

H	0.64341530	1.70458051	-3.40000000
H	7.06115818	0.64129638	-3.40000000
H	2.85442130	2.93225899	-3.40000000
C	2.63611980	-3.36061508	-3.40000000
C	2.67256562	-4.78323550	-3.40000000
C	3.95353817	-5.28476308	-3.40000000
S	5.13562671	-4.01999392	-3.40000000
C	3.89045079	-2.76557927	-3.40000000
H	6.40563363	-1.70512906	-3.40000000
H	1.71054805	-2.80225616	-3.40000000
H	1.78623206	-5.41006967	-3.40000000
H	4.27251374	-6.31803124	-3.40000000
C	5.61831941	-0.95898806	-3.40000000
C	6.00359842	0.38561300	-3.40000000
N	5.13432071	1.41967754	-3.40000000
C	3.84717376	1.05006154	-3.40000000
C	3.31823190	-0.28897137	-3.40000000
C	4.26257663	-1.35312483	-3.40000000
N	2.77741688	1.92183667	-3.40000000
C	1.59894071	1.19845616	-3.40000000
C	1.88527906	-0.15294544	-3.40000000
C	10.56576786	-5.25130253	-3.40000000
C	9.15417409	-5.12206356	-3.40000000
C	8.86906369	-3.73243966	-3.40000000
C	10.09735362	-3.07187131	-3.40000000
N	11.11100098	-4.01199774	-3.40000000
O	9.41835167	-0.88653039	-3.40000000
H	7.89784593	-3.26704718	-3.40000000
H	12.10010085	-3.78512041	-3.40000000
H	11.17913054	-6.14047006	-3.40000000
N	10.38190156	-1.70036556	-3.40000000
O	11.59479076	-1.33711050	-3.40000000
C	8.22037947	-6.18747592	-3.40000000
C	7.41542833	-7.09686156	-3.40000000
C	6.45154395	-8.19142600	-3.40000000
H	5.80821680	-8.15217927	-2.51142034
H	1.13675165	-0.92997363	-3.40000000

H	5.80821680	-8.15217927	-4.28857966
H	6.96213757	-9.16221965	-3.40000000
N	4.59362061	-1.42324430	0.00000000
C	4.11472266	-0.16348963	0.00000000
N	2.83096765	0.23291518	0.00000000
C	1.98311285	-0.81977333	0.00000000
C	2.34262891	-2.17899152	0.00000000
C	3.72679637	-2.47701379	0.00000000
N	0.60290236	-0.81623453	0.00000000
C	0.19167759	-2.13329993	0.00000000
N	1.20650731	-2.98811796	0.00000000
N	4.22675705	-3.72662446	0.00000000
H	4.87194754	0.61925678	0.00000000
H	0.00000000	0.00000000	0.00000000
H	-0.85706123	-2.39959278	0.00000000
H	3.59899438	-4.51969988	0.00000000
H	5.24292768	-3.87537936	0.00000000
N	7.44111734	-1.76645429	0.00000000
C	8.19168180	-0.60815965	0.00000000
N	9.55914485	-0.82510648	0.00000000
C	10.12842843	-2.07708710	0.00000000
C	9.38072615	-3.21416744	0.00000000
C	7.93307577	-3.07206955	0.00000000
O	7.13335774	-4.03769629	0.00000000
O	7.69054235	0.52818334	0.00000000
C	9.98429120	-4.59244466	0.00000000
H	6.39589445	-1.64161619	0.00000000
H	10.15048679	0.00000000	0.00000000
H	11.21327702	-2.08908493	0.00000000
H	9.66357774	-5.16052698	0.88220861
H	11.07688581	-4.53571428	0.00000000
H	9.66357774	-5.16052698	-0.88220861

Yi–Px//A–T

Total bonding energy = -10336.82 kcal mol^{-1}

H	0.37998460	-1.70606234	3.40000000
H	6.45292600	0.62520127	3.40000000
H	2.78840340	-2.47059574	3.40000000
N	1.29663477	3.48674098	3.40000000
C	1.10930758	4.85523564	3.40000000
C	2.32810725	5.50510609	3.40000000
N	3.27791672	4.50700577	3.40000000
C	2.62579611	3.28927872	3.40000000
H	5.31983108	2.81029874	3.40000000
H	0.36150850	1.01157804	3.40000000
H	0.12040366	5.29544667	3.40000000
H	2.58885462	6.55208993	3.40000000
C	4.71346553	1.91048349	3.40000000
C	5.36476652	0.66956926	3.40000000
N	4.72589493	-0.52066950	3.40000000
C	3.38982598	-0.42858109	3.40000000
C	2.60167730	0.77375255	3.40000000

C	3.30767021	2.00220866	3.40000000
N	2.51487456	-1.49485733	3.40000000
C	1.21346698	-1.01704799	3.40000000
C	1.22533367	0.36448605	3.40000000
C	9.72801902	5.04331332	3.40000000
C	8.35475266	4.68942914	3.40000000
C	8.29666085	3.27289247	3.40000000
C	9.61479146	2.81790339	3.40000000
N	10.46451658	3.90779230	3.40000000
O	9.29105395	0.55294654	3.40000000
H	7.41656178	2.65248676	3.40000000
H	11.47727959	3.84224232	3.40000000
H	10.19004866	6.01959016	3.40000000
N	10.11388019	1.50889839	3.40000000
O	11.36824523	1.34273072	3.40000000
C	7.27531769	5.60731580	3.40000000
C	6.39485048	6.44463634	3.40000000
C	5.37067354	7.48390039	3.40000000
H	4.73314941	7.41434070	2.50979424
H	4.28271061	4.65541795	3.40000000
H	4.73314941	7.41434070	4.29020576
H	5.83393638	8.47809103	3.40000000
N	4.59362061	1.42324430	-0.00000000
C	4.11472266	0.16348963	-0.00000000
N	2.83096765	-0.23291518	-0.00000000
C	1.98311285	0.81977333	-0.00000000
C	2.34262891	2.17899152	-0.00000000
C	3.72679637	2.47701379	-0.00000000
N	0.60290236	0.81623453	-0.00000000
C	0.19167759	2.13329993	-0.00000000
N	1.20650731	2.98811796	-0.00000000
N	4.22675705	3.72662446	-0.00000000
H	4.87194754	-0.61925678	-0.00000000
H	0.00000000	-0.00000000	-0.00000000
H	-0.85706123	2.39959278	-0.00000000
H	3.59899438	4.51969988	-0.00000000
H	5.24292768	3.87537936	-0.00000000
N	7.44111734	1.76645429	-0.00000000
C	8.19168180	0.60815965	-0.00000000
N	9.55914485	0.82510648	-0.00000000
C	10.12842843	2.07708710	-0.00000000
C	9.38072615	3.21416744	-0.00000000
C	7.93307577	3.07206955	-0.00000000
O	7.13335774	4.03769629	-0.00000000
O	7.69054235	-0.52818334	-0.00000000
C	9.98429120	4.59244466	-0.00000000
H	6.39589445	1.64161619	-0.00000000
H	10.15048679	-0.00000000	-0.00000000
H	11.21327702	2.08908493	-0.00000000
H	9.66357774	5.16052698	-0.88220861
H	11.07688581	4.53571428	-0.00000000
H	9.66357774	5.16052698	0.88220861

Do–Px//A–T

Total bonding energy = -10208.38 kcal mol^{-1}

H	0.83237044	-1.62353839	3.40000000
H	7.31196462	-1.47341866	3.40000000
H	2.77815600	-3.26363020	3.40000000
C	3.30247820	2.95823137	3.40000000
C	3.69180329	4.33570135	3.40000000
C	5.05972525	4.35579979	3.40000000
O	5.55562480	3.07082191	3.40000000
C	4.45865680	2.20719382	3.40000000
H	6.89889628	0.93847057	3.40000000
H	2.30001948	2.55559486	3.40000000
H	3.03909460	5.19887385	3.40000000
H	5.80344031	5.13971708	3.40000000
C	6.04638544	0.26939595	3.40000000
C	6.28597042	-1.11081297	3.40000000
N	5.32064340	-2.06087170	3.40000000
C	4.08205241	-1.55526456	3.40000000
C	3.70612160	-0.18367972	3.40000000
C	4.73226598	0.78896065	3.40000000
N	2.88504635	-2.25468929	3.40000000
C	1.87334279	-1.32715606	3.40000000
N	2.31488143	-0.07370929	3.40000000
C	10.71236233	5.09756921	3.40000000
C	9.29727574	5.01970994	3.40000000
C	8.95757835	3.64115883	3.40000000
C	10.16307424	2.93732253	3.40000000
N	11.21087946	3.83971418	3.40000000
O	9.42171032	0.77068360	3.40000000
H	7.96683791	3.21292959	3.40000000
H	12.19046454	3.57481225	3.40000000
H	11.35737134	5.96401748	3.40000000
N	10.40688612	1.55785972	3.40000000
O	11.60902372	1.15947470	3.40000000
C	8.40757515	6.12218966	3.40000000
C	7.63469843	7.05927910	3.40000000
C	6.69345103	8.17376012	3.40000000
H	6.04846780	8.14506491	2.51227243
H	6.04846780	8.14506491	4.28772757
H	7.22389739	9.13383998	3.40000000
N	4.59362061	1.42324430	-0.00000000
C	4.11472266	0.16348963	-0.00000000
N	2.83096765	-0.23291518	-0.00000000
C	1.98311285	0.81977333	-0.00000000
C	2.34262891	2.17899152	-0.00000000
C	3.72679637	2.47701379	-0.00000000
N	0.60290236	0.81623453	-0.00000000
C	0.19167759	2.13329993	-0.00000000
N	1.20650731	2.98811796	-0.00000000
N	4.22675705	3.72662446	-0.00000000
H	4.87194754	-0.61925678	-0.00000000
H	0.00000000	-0.00000000	-0.00000000

H	-0.85706123	2.39959278	-0.00000000
H	3.59899438	4.51969988	-0.00000000
H	5.24292768	3.87537936	-0.00000000
N	7.44111734	1.76645429	-0.00000000
C	8.19168180	0.60815965	-0.00000000
N	9.55914485	0.82510648	-0.00000000
C	10.12842843	2.07708710	-0.00000000
C	9.38072615	3.21416744	-0.00000000
C	7.93307577	3.07206955	-0.00000000
O	7.13335774	4.03769629	-0.00000000
O	7.69054235	-0.52818334	-0.00000000
C	9.98429120	4.59244466	-0.00000000
H	6.39589445	1.64161619	-0.00000000
H	10.15048679	-0.00000000	-0.00000000
H	11.21327702	2.08908493	-0.00000000
H	9.66357774	5.16052698	-0.88220861
H	11.07688581	4.53571428	-0.00000000
H	9.66357774	5.16052698	0.88220861

Dp–Px//A–T

Total bonding energy = -10411.09 kcal mol^{-1}

H	0.90392683	-1.60454672	3.40000000
H	7.37804042	-1.40454385	3.40000000
H	2.86726696	-3.22262370	3.40000000
C	4.55090521	2.31229414	3.40000000
N	5.65987461	3.09016750	3.40000000
H	6.32286956	8.41640305	4.28716398
H	6.32286956	8.41640305	2.51283602
N	5.55692815	4.42784478	3.40000000
H	6.93850775	1.01518909	3.40000000
C	4.34085782	4.99785386	3.40000000
C	3.15456288	4.25724992	3.40000000
C	3.25830826	2.87707813	3.40000000
C	6.09883004	0.33170060	3.40000000
C	6.35094791	-1.04563530	3.40000000
N	5.38829544	-1.99738778	3.40000000
C	4.15045269	-1.50073698	3.40000000
C	3.76283958	-0.12543862	3.40000000
C	4.78713103	0.84748765	3.40000000
N	2.96223287	-2.21245143	3.40000000
C	1.94182383	-1.29749541	3.40000000
N	2.36748429	-0.03949770	3.40000000
C	10.87666721	5.20687774	3.40000000
C	9.45938592	5.18765829	3.40000000
C	9.06006267	3.82352608	3.40000000
C	10.23821963	3.07154724	3.40000000
N	11.32333103	3.93021664	3.40000000
O	9.42911222	0.92622325	3.40000000
H	8.04783550	3.44050368	3.40000000
H	12.29069691	3.62389892	3.40000000
H	11.55724148	6.04589497	3.40000000
N	10.43632199	1.68532919	3.40000000

O	11.62653656	1.24836750	3.40000000
C	8.62110403	6.32977952	3.40000000
C	7.88251742	7.29327638	3.40000000
C	6.96939324	8.43098626	3.40000000
H	2.18906837	4.75520605	3.40000000
H	2.38708624	2.23319119	3.40000000
H	4.33760985	6.08556897	3.40000000
H	7.51976959	9.38000780	3.40000000
N	4.59362061	1.42324430	-0.00000000
C	4.11472266	0.16348963	-0.00000000
N	2.83096765	-0.23291518	-0.00000000
C	1.98311285	0.81977333	-0.00000000
C	2.34262891	2.17899152	-0.00000000
C	3.72679637	2.47701379	-0.00000000
N	0.60290236	0.81623453	-0.00000000
C	0.19167759	2.13329993	-0.00000000
N	1.20650731	2.98811796	-0.00000000
N	4.22675705	3.72662446	-0.00000000
H	4.87194754	-0.61925678	-0.00000000
H	0.00000000	-0.00000000	-0.00000000
H	-0.85706123	2.39959278	-0.00000000
H	3.59899438	4.51969988	-0.00000000
H	5.24292768	3.87537936	-0.00000000
N	7.44111734	1.76645429	-0.00000000
C	8.19168180	0.60815965	-0.00000000
N	9.55914485	0.82510648	-0.00000000
C	10.12842843	2.07708710	-0.00000000
C	9.38072615	3.21416744	-0.00000000
C	7.93307577	3.07206955	-0.00000000
O	7.13335774	4.03769629	-0.00000000
O	7.69054235	-0.52818334	-0.00000000
C	9.98429120	4.59244466	-0.00000000
H	6.39589445	1.64161619	-0.00000000
H	10.15048679	-0.00000000	-0.00000000
H	11.21327702	2.08908493	-0.00000000
H	9.66357774	5.16052698	-0.88220861
H	11.07688581	4.53571428	-0.00000000
H	9.66357774	5.16052698	0.88220861

Dss–Px//A–T

Total bonding energy = -11183.48 kcal mol^{-1}

H	1.42311347	1.88501196	-3.40000000
H	7.79644854	0.70422280	-3.40000000
H	3.60830377	3.19078231	-3.40000000
C	3.09996296	-2.96239808	-3.40000000
C	2.99362964	-4.37075160	-3.40000000
C	4.21967879	-5.02628143	-3.40000000
S	5.53429393	-3.86023004	-3.40000000
C	4.40898718	-2.49692969	-3.40000000
H	7.01027434	-1.60245818	-3.40000000
H	2.25366064	-2.28642872	-3.40000000
H	2.04540923	-4.89942255	-3.40000000

H	3.83782220	-9.95246205	-3.40000000
C	6.26757188	-0.81053474	-3.40000000
C	6.72546877	0.51200928	-3.40000000
N	5.92167733	1.60257050	-3.40000000
C	4.61962365	1.29526312	-3.40000000
C	4.02991432	-0.00064625	-3.40000000
C	4.88916405	-1.12851413	-3.40000000
N	3.55065304	2.17780644	-3.40000000
C	2.40321604	1.42572425	-3.40000000
N	2.63817993	0.11818153	-3.40000000
C	11.87591471	-5.41100836	-3.40000000
C	10.49604882	-5.73755271	-3.40000000
C	9.78084047	-4.51135315	-3.40000000
C	10.73936877	-3.49502405	-3.40000000
N	11.99850626	-4.06424462	-3.40000000
O	9.42501904	-1.61997695	-3.40000000
H	8.70987690	-4.37381342	-3.40000000
H	12.86360040	-3.53358431	-3.40000000
H	12.73885688	-6.06058584	-3.40000000
N	10.58897829	-2.10202416	-3.40000000
O	11.63335065	-1.38517121	-3.40000000
C	9.97946862	-7.05658307	-3.40000000
C	9.59585199	-8.20900124	-3.40000000
C	9.16058818	-9.60073427	-3.40000000
C	4.48023251	-6.44506480	-3.40000000
C	5.69280981	-7.12170796	-3.40000000
C	5.54887211	-8.53744632	-3.40000000
C	4.23430439	-8.94517123	-3.40000000
S	3.14327686	-7.59575396	-3.40000000
H	8.55815132	-9.82950263	-4.28825653
H	8.55815132	-9.82950263	-2.51174347
H	6.65487057	-6.61835651	-3.40000000
H	6.38230375	-9.23037164	-3.40000000
H	10.02544758	-10.27576236	-3.40000000
N	4.59362061	-1.42324430	0.00000000
C	4.11472266	-0.16348963	0.00000000
N	2.83096765	0.23291518	0.00000000
C	1.98311285	-0.81977333	0.00000000
C	2.34262891	-2.17899152	0.00000000
C	3.72679637	-2.47701379	0.00000000
N	0.60290236	-0.81623453	0.00000000
C	0.19167759	-2.13329993	0.00000000
N	1.20650731	-2.98811796	0.00000000
N	4.22675705	-3.72662446	0.00000000
H	4.87194754	0.61925678	0.00000000
H	0.00000000	0.00000000	0.00000000
H	-0.85706123	-2.39959278	0.00000000
H	3.59899438	-4.51969988	0.00000000
H	5.24292768	-3.87537936	0.00000000
N	7.44111734	-1.76645429	0.00000000
C	8.19168180	-0.60815965	0.00000000
N	9.55914485	-0.82510648	0.00000000

C	10.12842843	-2.07708710	0.00000000
C	9.38072615	-3.21416744	0.00000000
C	7.93307577	-3.07206955	0.00000000
O	7.13335774	-4.03769629	0.00000000
O	7.69054235	0.52818334	0.00000000
C	9.98429120	-4.59244466	0.00000000
H	6.39589445	-1.64161619	0.00000000
H	10.15048679	0.00000000	0.00000000
H	11.21327702	-2.08908493	0.00000000
H	9.66357774	-5.16052698	0.88220861
H	11.07688581	-4.53571428	0.00000000
H	9.66357774	-5.16052698	-0.88220861

Dt–Px//A–T

Total bonding energy = -10049.57 kcal mol^{-1}

H	0.69028491	1.69134856	-3.40000000
H	7.10996047	0.82024353	-3.40000000
H	2.81263731	3.09732252	-3.40000000
N	2.66282096	-3.01594438	-3.40000000
C	2.62891808	-4.39128354	-3.40000000
C	3.85158116	-5.01794679	-3.40000000
S	5.12096458	-3.84985976	-3.40000000
C	3.90291000	-2.55716468	-3.40000000
H	6.43474763	-1.52050721	-3.40000000
H	5.70446324	-7.87184388	-4.28865412
H	1.67248474	-4.90402474	-3.40000000
H	4.06339550	-6.07815540	-3.40000000
C	5.65481116	-0.76697485	-3.40000000
C	6.05073731	0.57440118	-3.40000000
N	5.19045924	1.61906605	-3.40000000
C	3.90744587	1.25124067	-3.40000000
C	3.37573754	-0.07554979	-3.40000000
C	4.29587624	-1.14965913	-3.40000000
N	2.80073046	2.08274456	-3.40000000
C	1.69054488	1.27775061	-3.40000000
N	1.98256505	-0.01839972	-3.40000000
C	10.53877202	-5.08930069	-3.40000000
C	9.12952746	-4.93688558	-3.40000000
C	8.86769308	-3.54296128	-3.40000000
C	10.10610631	-2.90233747	-3.40000000
N	11.10425525	-3.85887970	-3.40000000
O	9.46043272	-0.70692493	-3.40000000
H	7.90468337	-3.06200081	-3.40000000
H	12.09691634	-3.64810104	-3.40000000
H	11.13760774	-5.98828193	-3.40000000
N	10.41165559	-1.53521454	-3.40000000
O	11.62971591	-1.19045987	-3.40000000
C	8.17579632	-5.98426822	-3.40000000
C	7.34490705	-6.87007604	-3.40000000
C	6.34586715	-7.93257553	-3.40000000
H	5.70446324	-7.87184388	-2.51134588
H	6.82401709	-8.91976538	-3.40000000

N	4.59362061	-1.42324430	0.00000000
C	4.11472266	-0.16348963	0.00000000
N	2.83096765	0.23291518	0.00000000
C	1.98311285	-0.81977333	0.00000000
C	2.34262891	-2.17899152	0.00000000
C	3.72679637	-2.47701379	0.00000000
N	0.60290236	-0.81623453	0.00000000
C	0.19167759	-2.13329993	0.00000000
N	1.20650731	-2.98811796	0.00000000
N	4.22675705	-3.72662446	0.00000000
H	4.87194754	0.61925678	0.00000000
H	0.00000000	0.00000000	0.00000000
H	-0.85706123	-2.39959278	0.00000000
H	3.59899438	-4.51969988	0.00000000
H	5.24292768	-3.87537936	0.00000000
N	7.44111734	-1.76645429	0.00000000
C	8.19168180	-0.60815965	0.00000000
N	9.55914485	-0.82510648	0.00000000
C	10.12842843	-2.07708710	0.00000000
C	9.38072615	-3.21416744	0.00000000
C	7.93307577	-3.07206955	0.00000000
O	7.13335774	-4.03769629	0.00000000
O	7.69054235	0.52818334	0.00000000
C	9.98429120	-4.59244466	0.00000000
H	6.39589445	-1.64161619	0.00000000
H	10.15048679	0.00000000	0.00000000
H	11.21327702	-2.08908493	0.00000000
H	9.66357774	-5.16052698	0.88220861
H	11.07688581	-4.53571428	0.00000000
H	9.66357774	-5.16052698	-0.88220861

Yo–Px/A–T

Total bonding energy = -10305.47 kcal mol^{-1}

H	0.73648535	-1.70017431	3.40000000
H	7.23308988	-1.33621070	3.40000000
H	2.79775108	-3.16391546	3.40000000
C	3.29756253	3.16018684	3.40000000
C	3.73403244	4.52391509	3.40000000
C	5.10063116	4.49627928	3.40000000
O	5.54928677	3.19410775	3.40000000
C	4.42454051	2.36471911	3.40000000
H	6.83216127	1.08165185	3.40000000
H	2.27305995	2.82089387	3.40000000
H	3.10992099	5.40792769	3.40000000
H	5.87089255	5.25421767	3.40000000
C	5.97689002	0.41619133	3.40000000
C	6.20982246	-0.96499138	3.40000000
N	5.23645825	-1.90258745	3.40000000
C	3.99350770	-1.40198360	3.40000000
C	3.61282244	-0.01712739	3.40000000
C	4.66509065	0.93633913	3.40000000
N	2.83385452	-2.15119432	3.40000000

C	1.74151837	-1.30137089	3.40000000
C	2.17559113	0.01070325	3.40000000
C	10.72657033	5.19844012	3.40000000
C	9.31057857	5.14037946	3.40000000
C	8.95165993	3.76661954	3.40000000
C	10.14713718	3.04622174	3.40000000
N	11.20757875	3.93367544	3.40000000
O	9.37260585	0.89166328	3.40000000
H	7.95577035	3.35037991	3.40000000
H	12.18337688	3.65514084	3.40000000
H	11.38354263	6.05586349	3.40000000
N	10.36985955	1.66326381	3.40000000
O	11.56588748	1.24670188	3.40000000
C	8.43595739	6.25481057	3.40000000
C	7.67359143	7.20031721	3.40000000
C	6.74372706	8.32427848	3.40000000
H	6.09843904	8.30199850	4.28770667
H	6.09843904	8.30199850	2.51229333
H	1.52432866	0.87197335	3.40000000
H	7.28397113	9.27889781	3.40000000
N	4.59362061	1.42324430	-0.00000000
C	4.11472266	0.16348963	-0.00000000
N	2.83096765	-0.23291518	-0.00000000
C	1.98311285	0.81977333	-0.00000000
C	2.34262891	2.17899152	-0.00000000
C	3.72679637	2.47701379	-0.00000000
N	0.60290236	0.81623453	-0.00000000
C	0.19167759	2.13329993	-0.00000000
N	1.20650731	2.98811796	-0.00000000
N	4.22675705	3.72662446	-0.00000000
H	4.87194754	-0.61925678	-0.00000000
H	0.00000000	-0.00000000	-0.00000000
H	-0.85706123	2.39959278	-0.00000000
H	3.59899438	4.51969988	-0.00000000
H	5.24292768	3.87537936	-0.00000000
N	7.44111734	1.76645429	-0.00000000
C	8.19168180	0.60815965	-0.00000000
N	9.55914485	0.82510648	-0.00000000
C	10.12842843	2.07708710	-0.00000000
C	9.38072615	3.21416744	-0.00000000
C	7.93307577	3.07206955	-0.00000000
O	7.13335774	4.03769629	-0.00000000
O	7.69054235	-0.52818334	-0.00000000
C	9.98429120	4.59244466	-0.00000000
H	6.39589445	1.64161619	-0.00000000
H	10.15048679	-0.00000000	-0.00000000
H	11.21327702	2.08908493	-0.00000000
H	9.66357774	5.16052698	-0.88220861
H	11.07688581	4.53571428	-0.00000000
H	9.66357774	5.16052698	0.88220861

Ps–Px//A–T

Total bonding energy = -10050.94 kcal mol^{-1}

H	0.58825288	1.58808676	-3.40000000
H	6.79732989	-0.21249637	-3.40000000
H	2.87180978	2.71644095	-3.40000000
C	1.99190091	-3.45079448	-3.40000000
C	1.88600813	-4.86748874	-3.40000000
C	3.11632936	-5.49184878	-3.40000000
S	4.42860193	-4.36092678	-3.40000000
C	3.30735893	-3.00703458	-3.40000000
H	5.30731286	-7.93307579	-4.28860261
H	1.14894463	-2.76976065	-3.40000000
H	0.94341765	-5.40608111	-3.40000000
H	3.32137847	-6.55453338	-3.40000000
N	5.18441610	-1.50088414	-3.40000000
C	5.71156544	-0.26520707	-3.40000000
N	5.05101966	0.91321339	-3.40000000
C	3.72199424	0.74817893	-3.40000000
C	3.03284726	-0.49285019	-3.40000000
C	3.82876423	-1.65662285	-3.40000000
N	2.73521787	1.71081809	-3.40000000
C	1.52672126	1.04914205	-3.40000000
N	1.65444673	-0.27103601	-3.40000000
C	10.04323741	-5.05806674	-3.40000000
C	8.63626384	-4.88501306	-3.40000000
C	8.38782630	-3.48561934	-3.40000000
C	9.64203672	-2.86669631	-3.40000000
N	10.62580199	-3.83845528	-3.40000000
O	9.08046528	-0.64400549	-3.40000000
H	7.42696872	-2.98763365	-3.40000000
H	11.62106291	-3.64026354	-3.40000000
H	10.62706824	-5.96687747	-3.40000000
N	9.99573420	-1.51144872	-3.40000000
O	11.22659361	-1.21193459	-3.40000000
C	7.69429565	-5.94310006	-3.40000000
C	6.90647927	-6.86704938	-3.40000000
C	5.95064753	-7.96853062	-3.40000000
H	5.30731286	-7.93307579	-2.51139739
H	6.46744146	-8.93596293	-3.40000000
N	4.59362061	-1.42324430	0.00000000
C	4.11472266	-0.16348963	0.00000000
N	2.83096765	0.23291518	0.00000000
C	1.98311285	-0.81977333	0.00000000
C	2.34262891	-2.17899152	0.00000000
C	3.72679637	-2.47701379	0.00000000
N	0.60290236	-0.81623453	0.00000000
C	0.19167759	-2.13329993	0.00000000
N	1.20650731	-2.98811796	0.00000000
N	4.22675705	-3.72662446	0.00000000
H	4.87194754	0.61925678	0.00000000
H	0.00000000	0.00000000	0.00000000
H	-0.85706123	-2.39959278	0.00000000

H	3.59899438	-4.51969988	0.00000000
H	5.24292768	-3.87537936	0.00000000
N	7.44111734	-1.76645429	0.00000000
C	8.19168180	-0.60815965	0.00000000
N	9.55914485	-0.82510648	0.00000000
C	10.12842843	-2.07708710	0.00000000
C	9.38072615	-3.21416744	0.00000000
C	7.93307577	-3.07206955	0.00000000
O	7.13335774	-4.03769629	0.00000000
O	7.69054235	0.52818334	0.00000000
C	9.98429120	-4.59244466	0.00000000
H	6.39589445	-1.64161619	0.00000000
H	10.15048679	0.00000000	0.00000000
H	11.21327702	-2.08908493	0.00000000
H	9.66357774	-5.16052698	0.88220861
H	11.07688581	-4.53571428	0.00000000
H	9.66357774	-5.16052698	-0.88220861

Es–Px//A–T

Total bonding energy = -10148.95 kcal mol^{-1}

H	0.53297295	1.67460949	-3.40000000
H	6.71997679	-0.29816285	-3.40000000
H	2.87833601	2.61879523	-3.40000000
C	1.99788041	-3.65327740	-3.40000000
C	1.95858663	-5.07456093	-3.40000000
C	3.21577717	-5.63973935	-3.40000000
S	4.47119426	-4.44548631	-3.40000000
C	3.28799465	-3.14250877	-3.40000000
H	0.69680120	-1.01075183	-3.40000000
H	1.10784015	-3.03749196	-3.40000000
H	1.04091196	-5.65463216	-3.40000000
H	3.47046491	-6.69121652	-3.40000000
N	5.12136638	-1.60733665	-3.40000000
C	5.63497490	-0.36552808	-3.40000000
N	4.95566271	0.79827803	-3.40000000
C	3.62512692	0.63049559	-3.40000000
C	2.93605903	-0.62784026	-3.40000000
C	3.76816049	-1.77132564	-3.40000000
N	2.68000270	1.62464051	-3.40000000
C	1.41523679	1.04998367	-3.40000000
C	1.53016748	-0.32453607	-3.40000000
C	10.02544626	-5.14398788	-3.40000000
C	8.61622787	-4.98808791	-3.40000000
C	8.35098724	-3.59189399	-3.40000000
C	9.59780198	-2.95780616	-3.40000000
N	10.59318362	-3.91752976	-3.40000000
O	9.00941983	-0.74223276	-3.40000000
H	7.38537526	-3.10329393	-3.40000000
H	11.58603136	-3.70771576	-3.40000000
H	10.62005548	-6.04580735	-3.40000000
N	9.93506758	-1.59844359	-3.40000000
O	11.16238405	-1.28411742	-3.40000000

C	7.69064692	-6.06084450	-3.40000000
C	6.92700129	-7.00496193	-3.40000000
C	6.00517686	-8.13525689	-3.40000000
H	5.36102132	-8.12057412	-2.51146076
H	5.36102132	-8.12057412	-4.28853924
H	6.55267807	-9.08571006	-3.40000000
N	4.59362061	-1.42324430	0.00000000
C	4.11472266	-0.16348963	0.00000000
N	2.83096765	0.23291518	0.00000000
C	1.98311285	-0.81977333	0.00000000
C	2.34262891	-2.17899152	0.00000000
C	3.72679637	-2.47701379	0.00000000
N	0.60290236	-0.81623453	0.00000000
C	0.19167759	-2.13329993	0.00000000
N	1.20650731	-2.98811796	0.00000000
N	4.22675705	-3.72662446	0.00000000
H	4.87194754	0.61925678	0.00000000
H	0.00000000	0.00000000	0.00000000
H	-0.85706123	-2.39959278	0.00000000
H	3.59899438	-4.51969988	0.00000000
H	5.24292768	-3.87537936	0.00000000
N	7.44111734	-1.76645429	0.00000000
C	8.19168180	-0.60815965	0.00000000
N	9.55914485	-0.82510648	0.00000000
C	10.12842843	-2.07708710	0.00000000
C	9.38072615	-3.21416744	0.00000000
C	7.93307577	-3.07206955	0.00000000
O	7.13335774	-4.03769629	0.00000000
O	7.69054235	0.52818334	0.00000000
C	9.98429120	-4.59244466	0.00000000
H	6.39589445	-1.64161619	0.00000000
H	10.15048679	0.00000000	0.00000000
H	11.21327702	-2.08908493	0.00000000
H	9.66357774	-5.16052698	0.88220861
H	11.07688581	-4.53571428	0.00000000
H	9.66357774	-5.16052698	-0.88220861

Ysm–Px//A–T

Total bonding energy = -10617.11 kcal mol^{-1}

H	0.94225144	-1.81487081	3.40000000
H	7.36293034	-0.76907291	3.40000000
H	3.14960136	-3.04849708	3.40000000
C	2.95407785	3.24812740	3.40000000
C	2.99892198	4.67062585	3.40000000
C	4.27600233	5.18819570	3.40000000
S	5.45210253	3.89682435	3.40000000
C	4.20159235	2.64358428	3.40000000
H	6.71663042	1.58193959	3.40000000
H	2.02441214	2.69595488	3.40000000
H	2.11374107	5.30058611	3.40000000
C	4.70736957	6.62419026	3.40000000
C	5.92666095	0.83742123	3.40000000

C	6.30626767	-0.50918218	3.40000000
N	5.43492035	-1.54195063	3.40000000
C	4.14815233	-1.16894017	3.40000000
C	3.62329432	0.17063890	3.40000000
C	4.57043160	1.23316579	3.40000000
N	3.07568247	-2.03785410	3.40000000
C	1.89891067	-1.31094548	3.40000000
C	2.18986409	0.03950226	3.40000000
C	11.16937151	5.40008156	3.40000000
C	9.75385048	5.47659657	3.40000000
C	9.26877995	4.14315768	3.40000000
C	10.38959954	3.31190960	3.40000000
N	11.52892770	4.09512218	3.40000000
O	9.41071979	1.24203723	3.40000000
H	8.23877022	3.82509475	3.40000000
H	12.47433124	3.72631574	3.40000000
H	11.90507094	6.19100872	3.40000000
N	10.47786555	1.91395653	3.40000000
O	11.62820364	1.38396767	3.40000000
C	8.98961713	6.66966759	3.40000000
C	8.33916671	7.69523816	3.40000000
C	7.56341762	8.93039871	3.40000000
H	6.92057261	8.99352390	2.51294286
H	5.31159059	6.86246930	4.28363540
H	3.82625998	7.27358674	3.40000000
H	5.31159059	6.86246930	2.51636460
H	1.44377069	0.81909927	3.40000000
H	6.92057261	8.99352390	4.28705714
H	8.22425628	9.80589451	3.40000000
N	4.59362061	1.42324430	-0.00000000
C	4.11472266	0.16348963	-0.00000000
N	2.83096765	-0.23291518	-0.00000000
C	1.98311285	0.81977333	-0.00000000
C	2.34262891	2.17899152	-0.00000000
C	3.72679637	2.47701379	-0.00000000
N	0.60290236	0.81623453	-0.00000000
C	0.19167759	2.13329993	-0.00000000
N	1.20650731	2.98811796	-0.00000000
N	4.22675705	3.72662446	-0.00000000
H	4.87194754	-0.61925678	-0.00000000
H	0.00000000	-0.00000000	-0.00000000
H	-0.85706123	2.39959278	-0.00000000
H	3.59899438	4.51969988	-0.00000000
H	5.24292768	3.87537936	-0.00000000
N	7.44111734	1.76645429	-0.00000000
C	8.19168180	0.60815965	-0.00000000
N	9.55914485	0.82510648	-0.00000000
C	10.12842843	2.07708710	-0.00000000
C	9.38072615	3.21416744	-0.00000000
C	7.93307577	3.07206955	-0.00000000
O	7.13335774	4.03769629	-0.00000000
O	7.69054235	-0.52818334	-0.00000000

C	9.98429120	4.59244466	-0.00000000
H	6.39589445	1.64161619	-0.00000000
H	10.15048679	-0.00000000	-0.00000000
H	11.21327702	2.08908493	-0.00000000
H	9.66357774	5.16052698	-0.88220861
H	11.07688581	4.53571428	-0.00000000
H	9.66357774	5.16052698	0.88220861

Yss–Px//A–T

Total bonding energy = -11279.53 kcal mol^{-1}

H	1.34421292	2.05010415	-3.40000000
H	7.71655353	0.74021916	-3.40000000
H	3.60058918	3.19080888	-3.40000000
C	3.14909318	-3.08954231	-3.40000000
C	3.12724784	-4.50286820	-3.40000000
C	4.38915645	-5.08227681	-3.40000000
S	5.62766917	-3.83686504	-3.40000000
C	4.42395165	-2.53829821	-3.40000000
H	6.97286198	-1.58311654	-3.40000000
H	2.24551520	-2.49614222	-3.40000000
H	2.21176592	-5.08637492	-3.40000000
C	4.74045235	-6.48170129	-3.40000000
C	6.21608108	-0.80553299	-3.40000000
C	6.64995325	0.52381889	-3.40000000
N	5.82098323	1.59152766	-3.40000000
C	4.52021719	1.27143908	-3.40000000
C	3.93998224	-0.04556954	-3.40000000
C	4.84476572	-1.14536263	-3.40000000
N	3.48474009	2.18408935	-3.40000000
C	2.27911331	1.50699290	-3.40000000
C	2.51380950	0.14525451	-3.40000000
C	11.98637971	-5.47527910	-3.40000000
C	10.62461211	-5.87001039	-3.40000000
C	9.84981463	-4.68039842	-3.40000000
C	10.75666419	-3.61779235	-3.40000000
N	12.04243682	-4.12416078	-3.40000000
O	9.35065449	-1.81039347	-3.40000000
H	8.77381025	-4.59242100	-3.40000000
H	12.88001410	-3.55108795	-3.40000000
H	12.88044082	-6.08134892	-3.40000000
N	10.53695225	-2.23417154	-3.40000000
O	11.54472066	-1.46657511	-3.40000000
C	10.17582782	-7.21389816	-3.40000000
C	9.85468625	-8.38530398	-3.40000000
C	9.49275064	-9.79864837	-3.40000000
C	5.99548533	-7.07517733	-3.40000000
C	5.94868269	-8.49728899	-3.40000000
C	4.66470886	-8.99292232	-3.40000000
S	3.48432361	-7.71971142	-3.40000000
H	6.92053384	-6.50764327	-3.40000000
H	8.90374641	-10.05839605	-4.28875568
H	8.90374641	-10.05839605	-2.51124432

H	6.82690998	-9.13144048	-3.40000000
H	4.33655243	-10.02452008	-3.40000000
H	1.73530554	-0.60198342	-3.40000000
H	10.39197570	-10.42693365	-3.40000000
N	4.59362061	-1.42324430	0.00000000
C	4.11472266	-0.16348963	0.00000000
N	2.83096765	0.23291518	0.00000000
C	1.98311285	-0.81977333	0.00000000
C	2.34262891	-2.17899152	0.00000000
C	3.72679637	-2.47701379	0.00000000
N	0.60290236	-0.81623453	0.00000000
C	0.19167759	-2.13329993	0.00000000
N	1.20650731	-2.98811796	0.00000000
N	4.22675705	-3.72662446	0.00000000
H	4.87194754	0.61925678	0.00000000
H	0.00000000	0.00000000	0.00000000
H	-0.85706123	-2.39959278	0.00000000
H	3.59899438	-4.51969988	0.00000000
H	5.24292768	-3.87537936	0.00000000
N	7.44111734	-1.76645429	0.00000000
C	8.19168180	-0.60815965	0.00000000
N	9.55914485	-0.82510648	0.00000000
C	10.12842843	-2.07708710	0.00000000
C	9.38072615	-3.21416744	0.00000000
C	7.93307577	-3.07206955	0.00000000
O	7.13335774	-4.03769629	0.00000000
O	7.69054235	0.52818334	0.00000000
C	9.98429120	-4.59244466	0.00000000
H	6.39589445	-1.64161619	0.00000000
H	10.15048679	0.00000000	0.00000000
H	11.21327702	-2.08908493	0.00000000
H	9.66357774	-5.16052698	0.88220861
H	11.07688581	-4.53571428	0.00000000
H	9.66357774	-5.16052698	-0.88220861

Cartesian Coordinates and Total Bonding Energies of Ds–Px//G–C variants in Water

Cartesian coordinates (Å) and total bonding energies (kcal mol^{-1}) of Ds–Px//G–C variants optimized at ZORA-BLYP-D3(BJ)-TZ2P level of theory in water.

Ds–Px//G–C

Total bonding energy = -10061.59 kcal mol^{-1}

H	0.72835180	-1.61491941	3.40000000
H	7.15665469	-0.79470301	3.40000000
H	2.83658218	-3.04173268	3.40000000
C	2.66810159	3.13253275	3.40000000
C	2.64781333	4.55472061	3.40000000
C	3.90896866	5.10779465	3.40000000
S	5.14395799	3.89437888	3.40000000
C	3.94889118	2.59653926	3.40000000
H	6.49913093	1.54944726	3.40000000
H	1.78443319	2.50625987	3.40000000
H	1.73850902	5.14783148	3.40000000
H	4.18453588	6.15370439	3.40000000
C	5.71288154	0.80203754	3.40000000
C	6.09841983	-0.54310717	3.40000000
N	5.23469936	-1.58581531	3.40000000
C	3.95256826	-1.20632359	3.40000000
C	3.43633743	0.12150696	3.40000000
C	4.35575875	1.19868788	3.40000000
N	2.83600395	-2.02714987	3.40000000
C	1.73271958	-1.21146491	3.40000000
N	2.04005101	0.08066988	3.40000000
C	10.57480611	5.15611150	3.40000000
C	9.16464152	5.01239854	3.40000000
C	8.89385902	3.62015575	3.40000000
C	10.12843458	2.97186964	3.40000000
N	11.13251700	3.92209180	3.40000000
O	9.47005675	0.78019117	3.40000000
H	7.92663630	3.14695291	3.40000000
H	12.12398134	3.70595778	3.40000000
H	11.17955605	6.05112367	3.40000000
N	10.42603070	1.60284302	3.40000000
O	11.64209002	1.25091364	3.40000000
C	8.21809502	6.06627741	3.40000000
C	7.39597007	6.96018605	3.40000000
C	6.40903286	8.03421887	3.40000000
H	5.76690644	7.98051793	4.28859404
H	6.89849248	9.01579896	3.40000000
H	5.76690644	7.98051793	2.51140596
N	5.01365243	-0.99199182	0.00000000
O	4.30260090	3.58796152	0.00000000
N	4.56716532	1.29195847	0.00000000
C	4.09292299	-0.00598124	0.00000000
N	2.78699383	-0.30650083	0.00000000
C	1.99120808	0.78584029	0.00000000
C	2.37604854	2.13811247	0.00000000

C	3.76721199	2.44848064	0.00000000
N	0.61326544	0.80857250	0.00000000
C	0.22151194	2.13729412	0.00000000
N	1.25137639	2.96774397	0.00000000
H	6.02751807	-0.80363867	0.00000000
H	4.68066827	-1.94733299	0.00000000
H	5.60211828	1.44276463	0.00000000
H	0.00000000	0.00000000	0.00000000
H	-0.82262775	2.42026635	0.00000000
O	7.83461905	-0.55738136	0.00000000
N	7.14844079	4.00206529	0.00000000
N	7.46163154	1.71656440	0.00000000
C	9.41590662	3.17447096	0.00000000
C	8.27003082	0.62421145	0.00000000
N	9.65603357	0.82777239	0.00000000
C	10.21432266	2.07245899	0.00000000
C	7.99620203	2.96060279	0.00000000
H	9.83388744	4.17428544	0.00000000
H	10.24447549	-0.00000000	0.00000000
H	11.29744761	2.11566317	0.00000000
H	6.12873984	3.85217674	0.00000000
H	7.51095075	4.94658058	0.00000000

Bs–Px//G–C

Total bonding energy = -10162.06 kcal mol^{-1}

H	0.69490344	1.58059634	-3.40000000
H	7.26230042	0.75743674	-3.40000000
H	2.74759204	3.07351205	-3.40000000
C	2.69400053	-3.08044515	-3.40000000
C	2.66956884	-4.50544151	-3.40000000
C	3.92609607	-5.06421769	-3.40000000
S	5.16487461	-3.84895964	-3.40000000
C	3.97359118	-2.54391135	-3.40000000
H	6.51179803	-1.55600420	-3.40000000
H	1.81180988	-2.45263858	-3.40000000
H	1.75710044	-5.09436379	-3.40000000
H	4.19993745	-6.11060064	-3.40000000
C	5.75547804	-0.77702335	-3.40000000
C	6.19417385	0.55829288	-3.40000000
C	5.29038309	1.62378742	-3.40000000
C	3.93602967	1.28063817	-3.40000000
C	3.46618398	-0.06485408	-3.40000000
C	4.39336743	-1.14322152	-3.40000000
N	2.78725872	2.06045644	-3.40000000
C	1.71250946	1.21122992	-3.40000000
N	2.06527701	-0.06827025	-3.40000000
C	10.58381796	-5.18855736	-3.40000000
C	9.17599765	-5.02361443	-3.40000000
C	8.92580519	-3.62709225	-3.40000000
C	10.17052614	-2.99797474	-3.40000000
N	11.16018558	-3.96338641	-3.40000000
O	9.54833460	-0.79577476	-3.40000000

H	7.96604111	-3.13786237	-3.40000000
H	12.15461291	-3.76108868	-3.40000000
H	11.17452735	-6.09291915	-3.40000000
N	10.49040698	-1.63421162	-3.40000000
O	11.71235964	-1.30239357	-3.40000000
C	8.21481982	-6.06439768	-3.40000000
C	7.38317862	-6.94932481	-3.40000000
C	6.38528599	-8.01294437	-3.40000000
H	5.74340846	-7.95299715	-2.51156331
H	5.62236909	2.65816133	-3.40000000
H	5.74340846	-7.95299715	-4.28843669
H	6.86502507	-8.99940465	-3.40000000
N	5.01365243	0.99199182	-0.00000000
O	4.30260090	-3.58796152	-0.00000000
N	4.56716532	-1.29195847	-0.00000000
C	4.09292299	0.00598124	-0.00000000
N	2.78699383	0.30650083	-0.00000000
C	1.99120808	-0.78584029	-0.00000000
C	2.37604854	-2.13811247	-0.00000000
C	3.76721199	-2.44848064	-0.00000000
N	0.61326544	-0.80857250	-0.00000000
C	0.22151194	-2.13729412	-0.00000000
N	1.25137639	-2.96774397	-0.00000000
H	6.02751807	0.80363867	-0.00000000
H	4.68066827	1.94733299	-0.00000000
H	5.60211828	-1.44276463	-0.00000000
H	0.00000000	-0.00000000	-0.00000000
H	-0.82262775	-2.42026635	-0.00000000
O	7.83461905	0.55738136	-0.00000000
N	7.14844079	-4.00206529	-0.00000000
N	7.46163154	-1.71656440	-0.00000000
C	9.41590662	-3.17447096	-0.00000000
C	8.27003082	-0.62421145	-0.00000000
N	9.65603357	-0.82777239	-0.00000000
C	10.21432266	-2.07245899	-0.00000000
C	7.99620203	-2.96060279	-0.00000000
H	9.83388744	-4.17428544	-0.00000000
H	10.24447549	0.00000000	-0.00000000
H	11.29744761	-2.11566317	-0.00000000
H	6.12873984	-3.85217674	-0.00000000
H	7.51095075	-4.94658058	-0.00000000

Is–Px//G–C
Total bonding energy = -10257.07 kcal mol^{-1}

H	0.60687107	1.67569398	-3.40000000
H	7.16668714	0.57538297	-3.40000000
H	2.77498112	2.96065268	-3.40000000
C	2.64996964	-3.32600625	-3.40000000
C	2.68187640	-4.75140897	-3.40000000
C	3.95837099	-5.25914822	-3.40000000
S	5.14445999	-3.99322542	-3.40000000
C	3.90328777	-2.72980461	-3.40000000

H	6.40730329	-1.73566872	-3.40000000
H	1.72335636	-2.77025199	-3.40000000
H	1.79188510	-5.37360520	-3.40000000
H	4.27486047	-6.29328149	-3.40000000
C	5.65284215	-0.95496653	-3.40000000
C	6.09822782	0.37759287	-3.40000000
C	5.19139068	1.43755934	-3.40000000
C	3.83266948	1.10750666	-3.40000000
C	3.34235209	-0.24496346	-3.40000000
C	4.29057598	-1.31553304	-3.40000000
N	2.73373423	1.94899302	-3.40000000
C	1.57675123	1.19705927	-3.40000000
C	1.90304906	-0.14537281	-3.40000000
C	10.57835411	-5.28358878	-3.40000000
C	9.16832854	-5.13784760	-3.40000000
C	8.89917894	-3.74479353	-3.40000000
C	10.13558177	-3.09882837	-3.40000000
N	11.13805614	-4.05096810	-3.40000000
O	9.48533062	-0.90481117	-3.40000000
H	7.93334705	-3.26719563	-3.40000000
H	12.12981195	-3.83602976	-3.40000000
H	11.18105038	-6.18002018	-3.40000000
N	10.43792670	-1.73125613	-3.40000000
O	11.65563685	-1.38387336	-3.40000000
C	8.22294254	-6.19302283	-3.40000000
C	7.40946161	-7.09478735	-3.40000000
C	6.43604551	-8.18097119	-3.40000000
H	5.52198207	2.47323192	-3.40000000
H	5.79281966	-8.13659663	-4.28828124
H	5.79281966	-8.13659663	-2.51171876
H	1.17495444	-0.94087425	-3.40000000
H	6.93953201	-9.15559481	-3.40000000
N	5.01365243	0.99199182	-0.00000000
O	4.30260090	-3.58796152	-0.00000000
N	4.56716532	-1.29195847	-0.00000000
C	4.09292299	0.00598124	-0.00000000
N	2.78699383	0.30650083	-0.00000000
C	1.99120808	-0.78584029	-0.00000000
C	2.37604854	-2.13811247	-0.00000000
C	3.76721199	-2.44848064	-0.00000000
N	0.61326544	-0.80857250	-0.00000000
C	0.22151194	-2.13729412	-0.00000000
N	1.25137639	-2.96774397	-0.00000000
H	6.02751807	0.80363867	-0.00000000
H	4.68066827	1.94733299	-0.00000000
H	5.60211828	-1.44276463	-0.00000000
H	0.00000000	-0.00000000	-0.00000000
H	-0.82262775	-2.42026635	-0.00000000
O	7.83461905	0.55738136	-0.00000000
N	7.14844079	-4.00206529	-0.00000000
N	7.46163154	-1.71656440	-0.00000000
C	9.41590662	-3.17447096	-0.00000000

C	8.27003082	-0.62421145	-0.00000000
N	9.65603357	-0.82777239	-0.00000000
C	10.21432266	-2.07245899	-0.00000000
C	7.99620203	-2.96060279	-0.00000000
H	9.83388744	-4.17428544	-0.00000000
H	10.24447549	0.00000000	-0.00000000
H	11.29744761	-2.11566317	-0.00000000
H	6.12873984	-3.85217674	-0.00000000
H	7.51095075	-4.94658058	-0.00000000

Ys–Px//G–C

Total bonding energy = -10157.93 kcal mol^{-1}

H	0.64341530	1.70458051	-3.40000000
H	7.06115818	0.64129638	-3.40000000
H	2.85442130	2.93225899	-3.40000000
C	2.63611980	-3.36061508	-3.40000000
C	2.67256562	-4.78323550	-3.40000000
C	3.95353817	-5.28476308	-3.40000000
S	5.13562671	-4.01999392	-3.40000000
C	3.89045079	-2.76557927	-3.40000000
H	6.40563363	-1.70512906	-3.40000000
H	1.71054805	-2.80225616	-3.40000000
H	1.78623206	-5.41006967	-3.40000000
H	4.27251374	-6.31803124	-3.40000000
C	5.61831941	-0.95898806	-3.40000000
C	6.00359842	0.38561300	-3.40000000
N	5.13432071	1.41967754	-3.40000000
C	3.84717376	1.05006154	-3.40000000
C	3.31823190	-0.28897137	-3.40000000
C	4.26257663	-1.35312483	-3.40000000
N	2.77741688	1.92183667	-3.40000000
C	1.59894071	1.19845616	-3.40000000
C	1.88527906	-0.15294544	-3.40000000
C	10.56576786	-5.25130253	-3.40000000
C	9.15417409	-5.12206356	-3.40000000
C	8.86906369	-3.73243966	-3.40000000
C	10.09735362	-3.07187131	-3.40000000
N	11.11100098	-4.01199774	-3.40000000
O	9.41835167	-0.88653039	-3.40000000
H	7.89784593	-3.26704718	-3.40000000
H	12.10010085	-3.78512041	-3.40000000
H	11.17913054	-6.14047006	-3.40000000
N	10.38190156	-1.70036556	-3.40000000
O	11.59479076	-1.33711050	-3.40000000
C	8.22037947	-6.18747592	-3.40000000
C	7.41542833	-7.09686156	-3.40000000
C	6.45154395	-8.19142600	-3.40000000
H	5.80821680	-8.15217927	-2.51142034
H	1.13675165	-0.92997363	-3.40000000
H	5.80821680	-8.15217927	-4.28857966
H	6.96213757	-9.16221965	-3.40000000
N	5.01365243	0.99199182	-0.00000000

O	4.30260090	-3.58796152	-0.00000000
N	4.56716532	-1.29195847	-0.00000000
C	4.09292299	0.00598124	-0.00000000
N	2.78699383	0.30650083	-0.00000000
C	1.99120808	-0.78584029	-0.00000000
C	2.37604854	-2.13811247	-0.00000000
C	3.76721199	-2.44848064	-0.00000000
N	0.61326544	-0.80857250	-0.00000000
C	0.22151194	-2.13729412	-0.00000000
N	1.25137639	-2.96774397	-0.00000000
H	6.02751807	0.80363867	-0.00000000
H	4.68066827	1.94733299	-0.00000000
H	5.60211828	-1.44276463	-0.00000000
H	0.00000000	-0.00000000	-0.00000000
H	-0.82262775	-2.42026635	-0.00000000
O	7.83461905	0.55738136	-0.00000000
N	7.14844079	-4.00206529	-0.00000000
N	7.46163154	-1.71656440	-0.00000000
C	9.41590662	-3.17447096	-0.00000000
C	8.27003082	-0.62421145	-0.00000000
N	9.65603357	-0.82777239	-0.00000000
C	10.21432266	-2.07245899	-0.00000000
C	7.99620203	-2.96060279	-0.00000000
H	9.83388744	-4.17428544	-0.00000000
H	10.24447549	0.00000000	-0.00000000
H	11.29744761	-2.11566317	-0.00000000
H	6.12873984	-3.85217674	-0.00000000
H	7.51095075	-4.94658058	-0.00000000

Yi–Px//G–C

Total bonding energy = -10247.37 kcal mol^{-1}

H	0.37998460	-1.70606234	3.40000000
H	6.45292600	0.62520127	3.40000000
H	2.78840340	-2.47059574	3.40000000
N	1.29663477	3.48674098	3.40000000
C	1.10930758	4.85523564	3.40000000
C	2.32810725	5.50510609	3.40000000
N	3.27791672	4.50700577	3.40000000
C	2.62579611	3.28927872	3.40000000
H	5.31983108	2.81029874	3.40000000
H	0.36150850	1.01157804	3.40000000
H	0.12040366	5.29544667	3.40000000
H	2.58885462	6.55208993	3.40000000
C	4.71346553	1.91048349	3.40000000
C	5.36476652	0.66956926	3.40000000
N	4.72589493	-0.52066950	3.40000000
C	3.38982598	-0.42858109	3.40000000
C	2.60167730	0.77375255	3.40000000
C	3.30767021	2.00220866	3.40000000
N	2.51487456	-1.49485733	3.40000000
C	1.21346698	-1.01704799	3.40000000
C	1.22533367	0.36448605	3.40000000

C	9.72801902	5.04331332	3.40000000
C	8.35475266	4.68942914	3.40000000
C	8.29666085	3.27289247	3.40000000
C	9.61479146	2.81790339	3.40000000
N	10.46451658	3.90779230	3.40000000
O	9.29105395	0.55294654	3.40000000
H	7.41656178	2.65248676	3.40000000
H	11.47727959	3.84224232	3.40000000
H	10.19004866	6.01959016	3.40000000
N	10.11388019	1.50889839	3.40000000
O	11.36824523	1.34273072	3.40000000
C	7.27531769	5.60731580	3.40000000
C	6.39485048	6.44463634	3.40000000
C	5.37067354	7.48390039	3.40000000
H	4.73314941	7.41434070	2.50979424
H	4.28271061	4.65541795	3.40000000
H	4.73314941	7.41434070	4.29020576
H	5.83393638	8.47809103	3.40000000
N	5.01365243	-0.99199182	0.00000000
O	4.30260090	3.58796152	0.00000000
N	4.56716532	1.29195847	0.00000000
C	4.09292299	-0.00598124	0.00000000
N	2.78699383	-0.30650083	0.00000000
C	1.99120808	0.78584029	0.00000000
C	2.37604854	2.13811247	0.00000000
C	3.76721199	2.44848064	0.00000000
N	0.61326544	0.80857250	0.00000000
C	0.22151194	2.13729412	0.00000000
N	1.25137639	2.96774397	0.00000000
H	6.02751807	-0.80363867	0.00000000
H	4.68066827	-1.94733299	0.00000000
H	5.60211828	1.44276463	0.00000000
H	0.00000000	0.00000000	0.00000000
H	-0.82262775	2.42026635	0.00000000
O	7.83461905	-0.55738136	0.00000000
N	7.14844079	4.00206529	0.00000000
N	7.46163154	1.71656440	0.00000000
C	9.41590662	3.17447096	0.00000000
C	8.27003082	0.62421145	0.00000000
N	9.65603357	0.82777239	0.00000000
C	10.21432266	2.07245899	0.00000000
C	7.99620203	2.96060279	0.00000000
H	9.83388744	4.17428544	0.00000000
H	10.24447549	-0.00000000	0.00000000
H	11.29744761	2.11566317	0.00000000
H	6.12873984	3.85217674	0.00000000
H	7.51095075	4.94658058	0.00000000

Do–Px//G–C

Total bonding energy = -10119.51 kcal mol^{-1}

H	0.83237044	-1.62353839	3.40000000
H	7.31196462	-1.47341866	3.40000000

H	2.77815600	-3.26363020	3.40000000
C	3.30247820	2.95823137	3.40000000
C	3.69180329	4.33570135	3.40000000
C	5.05972525	4.35579979	3.40000000
O	5.55562480	3.07082191	3.40000000
C	4.45865680	2.20719382	3.40000000
H	6.89889628	0.93847057	3.40000000
H	2.30001948	2.55559486	3.40000000
H	3.03909460	5.19887385	3.40000000
H	5.80344031	5.13971708	3.40000000
C	6.04638544	0.26939595	3.40000000
C	6.28597042	-1.11081297	3.40000000
N	5.32064340	-2.06087170	3.40000000
C	4.08205241	-1.55526456	3.40000000
C	3.70612160	-0.18367972	3.40000000
C	4.73226598	0.78896065	3.40000000
N	2.88504635	-2.25468929	3.40000000
C	1.87334279	-1.32715606	3.40000000
N	2.31488143	-0.07370929	3.40000000
C	10.71236233	5.09756921	3.40000000
C	9.29727574	5.01970994	3.40000000
C	8.95757835	3.64115883	3.40000000
C	10.16307424	2.93732253	3.40000000
N	11.21087946	3.83971418	3.40000000
O	9.42171032	0.77068360	3.40000000
H	7.96683791	3.21292959	3.40000000
H	12.19046454	3.57481225	3.40000000
H	11.35737134	5.96401748	3.40000000
N	10.40688612	1.55785972	3.40000000
O	11.60902372	1.15947470	3.40000000
C	8.40757515	6.12218966	3.40000000
C	7.63469843	7.05927910	3.40000000
C	6.69345103	8.17376012	3.40000000
H	6.04846780	8.14506491	2.51227243
H	6.04846780	8.14506491	4.28772757
H	7.22389739	9.13383998	3.40000000
N	5.01365243	-0.99199182	0.00000000
O	4.30260090	3.58796152	0.00000000
N	4.56716532	1.29195847	0.00000000
C	4.09292299	-0.00598124	0.00000000
N	2.78699383	-0.30650083	0.00000000
C	1.99120808	0.78584029	0.00000000
C	2.37604854	2.13811247	0.00000000
C	3.76721199	2.44848064	0.00000000
N	0.61326544	0.80857250	0.00000000
C	0.22151194	2.13729412	0.00000000
N	1.25137639	2.96774397	0.00000000
H	6.02751807	-0.80363867	0.00000000
H	4.68066827	-1.94733299	0.00000000
H	5.60211828	1.44276463	0.00000000
H	0.00000000	0.00000000	0.00000000
H	-0.82262775	2.42026635	0.00000000

O	7.83461905	-0.55738136	0.00000000
N	7.14844079	4.00206529	0.00000000
N	7.46163154	1.71656440	0.00000000
C	9.41590662	3.17447096	0.00000000
C	8.27003082	0.62421145	0.00000000
N	9.65603357	0.82777239	0.00000000
C	10.21432266	2.07245899	0.00000000
C	7.99620203	2.96060279	0.00000000
H	9.83388744	4.17428544	0.00000000
H	10.24447549	-0.00000000	0.00000000
H	11.29744761	2.11566317	0.00000000
H	6.12873984	3.85217674	0.00000000
H	7.51095075	4.94658058	0.00000000

Dp–Px//G–C

Total bonding energy = -10321.82 kcal mol^{-1}

H	0.90392683	-1.60454672	3.40000000
H	7.37804042	-1.40454385	3.40000000
H	2.86726696	-3.22262370	3.40000000
C	4.55090521	2.31229414	3.40000000
N	5.65987461	3.09016750	3.40000000
H	6.32286956	8.41640305	4.28716398
H	6.32286956	8.41640305	2.51283602
N	5.55692815	4.42784478	3.40000000
H	6.93850775	1.01518909	3.40000000
C	4.34085782	4.99785386	3.40000000
C	3.15456288	4.25724992	3.40000000
C	3.25830826	2.87707813	3.40000000
C	6.09883004	0.33170060	3.40000000
C	6.35094791	-1.04563530	3.40000000
N	5.38829544	-1.99738778	3.40000000
C	4.15045269	-1.50073698	3.40000000
C	3.76283958	-0.12543862	3.40000000
C	4.78713103	0.84748765	3.40000000
N	2.96223287	-2.21245143	3.40000000
C	1.94182383	-1.29749541	3.40000000
N	2.36748429	-0.03949770	3.40000000
C	10.87666721	5.20687774	3.40000000
C	9.45938592	5.18765829	3.40000000
C	9.06006267	3.82352608	3.40000000
C	10.23821963	3.07154724	3.40000000
N	11.32333103	3.93021664	3.40000000
O	9.42911222	0.92622325	3.40000000
H	8.04783550	3.44050368	3.40000000
H	12.29069691	3.62389892	3.40000000
H	11.55724148	6.04589497	3.40000000
N	10.43632199	1.68532919	3.40000000
O	11.62653656	1.24836750	3.40000000
C	8.62110403	6.32977952	3.40000000
C	7.88251742	7.29327638	3.40000000
C	6.96939324	8.43098626	3.40000000
H	2.18906837	4.75520605	3.40000000

H	2.38708624	2.23319119	3.40000000
H	4.33760985	6.08556897	3.40000000
H	7.51976959	9.38000780	3.40000000
N	5.01365243	-0.99199182	0.00000000
O	4.30260090	3.58796152	0.00000000
N	4.56716532	1.29195847	0.00000000
C	4.09292299	-0.00598124	0.00000000
N	2.78699383	-0.30650083	0.00000000
C	1.99120808	0.78584029	0.00000000
C	2.37604854	2.13811247	0.00000000
C	3.76721199	2.44848064	0.00000000
N	0.61326544	0.80857250	0.00000000
C	0.22151194	2.13729412	0.00000000
N	1.25137639	2.96774397	0.00000000
H	6.02751807	-0.80363867	0.00000000
H	4.68066827	-1.94733299	0.00000000
H	5.60211828	1.44276463	0.00000000
H	0.00000000	0.00000000	0.00000000
H	-0.82262775	2.42026635	0.00000000
O	7.83461905	-0.55738136	0.00000000
N	7.14844079	4.00206529	0.00000000
N	7.46163154	1.71656440	0.00000000
C	9.41590662	3.17447096	0.00000000
C	8.27003082	0.62421145	0.00000000
N	9.65603357	0.82777239	0.00000000
C	10.21432266	2.07245899	0.00000000
C	7.99620203	2.96060279	0.00000000
H	9.83388744	4.17428544	0.00000000
H	10.24447549	-0.00000000	0.00000000
H	11.29744761	2.11566317	0.00000000
H	6.12873984	3.85217674	0.00000000
H	7.51095075	4.94658058	0.00000000

Dss–Px//G–C

Total bonding energy = -11093.23 kcal mol^{-1}

H	1.42311347	1.88501196	-3.40000000
H	7.79644854	0.70422280	-3.40000000
H	3.60830377	3.19078231	-3.40000000
C	3.09996296	-2.96239808	-3.40000000
C	2.99362964	-4.37075160	-3.40000000
C	4.21967879	-5.02628143	-3.40000000
S	5.53429393	-3.86023004	-3.40000000
C	4.40898718	-2.49692969	-3.40000000
H	7.01027434	-1.60245818	-3.40000000
H	2.25366064	-2.28642872	-3.40000000
H	2.04540923	-4.89942255	-3.40000000
H	3.83782220	-9.95246205	-3.40000000
C	6.26757188	-0.81053474	-3.40000000
C	6.72546877	0.51200928	-3.40000000
N	5.92167733	1.60257050	-3.40000000
C	4.61962365	1.29526312	-3.40000000
C	4.02991432	-0.00064625	-3.40000000

C	4.88916405	-1.12851413	-3.40000000
N	3.55065304	2.17780644	-3.40000000
C	2.40321604	1.42572425	-3.40000000
N	2.63817993	0.11818153	-3.40000000
C	11.87591471	-5.41100836	-3.40000000
C	10.49604882	-5.73755271	-3.40000000
C	9.78084047	-4.51135315	-3.40000000
C	10.73936877	-3.49502405	-3.40000000
N	11.99850626	-4.06424462	-3.40000000
O	9.42501904	-1.61997695	-3.40000000
H	8.70987690	-4.37381342	-3.40000000
H	12.86360040	-3.53358431	-3.40000000
H	12.73885688	-6.06058584	-3.40000000
N	10.58897829	-2.10202416	-3.40000000
O	11.63335065	-1.38517121	-3.40000000
C	9.97946862	-7.05658307	-3.40000000
C	9.59585199	-8.20900124	-3.40000000
C	9.16058818	-9.60073427	-3.40000000
C	4.48023251	-6.44506480	-3.40000000
C	5.69280981	-7.12170796	-3.40000000
C	5.54887211	-8.53744632	-3.40000000
C	4.23430439	-8.94517123	-3.40000000
S	3.14327686	-7.59575396	-3.40000000
H	8.55815132	-9.82950263	-4.28825653
H	8.55815132	-9.82950263	-2.51174347
H	6.65487057	-6.61835651	-3.40000000
H	6.38230375	-9.23037164	-3.40000000
H	10.02544758	-10.27576236	-3.40000000
N	5.01365243	0.99199182	-0.00000000
O	4.30260090	-3.58796152	-0.00000000
N	4.56716532	-1.29195847	-0.00000000
C	4.09292299	0.00598124	-0.00000000
N	2.78699383	0.30650083	-0.00000000
C	1.99120808	-0.78584029	-0.00000000
C	2.37604854	-2.13811247	-0.00000000
C	3.76721199	-2.44848064	-0.00000000
N	0.61326544	-0.80857250	-0.00000000
C	0.22151194	-2.13729412	-0.00000000
N	1.25137639	-2.96774397	-0.00000000
H	6.02751807	0.80363867	-0.00000000
H	4.68066827	1.94733299	-0.00000000
H	5.60211828	-1.44276463	-0.00000000
H	0.00000000	-0.00000000	-0.00000000
H	-0.82262775	-2.42026635	-0.00000000
O	7.83461905	0.55738136	-0.00000000
N	7.14844079	-4.00206529	-0.00000000
N	7.46163154	-1.71656440	-0.00000000
C	9.41590662	-3.17447096	-0.00000000
C	8.27003082	-0.62421145	-0.00000000
N	9.65603357	-0.82777239	-0.00000000
C	10.21432266	-2.07245899	-0.00000000
C	7.99620203	-2.96060279	-0.00000000

H	9.83388744	-4.17428544	-0.00000000
H	10.24447549	0.00000000	-0.00000000
H	11.29744761	-2.11566317	-0.00000000
H	6.12873984	-3.85217674	-0.00000000
H	7.51095075	-4.94658058	-0.00000000

Dt–Px//G–C

Total bonding energy = -9960.13 kcal mol^{-1}

H	0.69028491	1.69134856	-3.40000000
H	7.10996047	0.82024353	-3.40000000
H	2.81263731	3.09732252	-3.40000000
N	2.66282096	-3.01594438	-3.40000000
C	2.62891808	-4.39128354	-3.40000000
C	3.85158116	-5.01794679	-3.40000000
S	5.12096458	-3.84985976	-3.40000000
C	3.90291000	-2.55716468	-3.40000000
H	6.43474763	-1.52050721	-3.40000000
H	5.70446324	-7.87184388	-4.28865412
H	1.67248474	-4.90402474	-3.40000000
H	4.06339550	-6.07815540	-3.40000000
C	5.65481116	-0.76697485	-3.40000000
C	6.05073731	0.57440118	-3.40000000
N	5.19045924	1.61906605	-3.40000000
C	3.90744587	1.25124067	-3.40000000
C	3.37573754	-0.07554979	-3.40000000
C	4.29587624	-1.14965913	-3.40000000
N	2.80073046	2.08274456	-3.40000000
C	1.69054488	1.27775061	-3.40000000
N	1.98256505	-0.01839972	-3.40000000
C	10.53877202	-5.08930069	-3.40000000
C	9.12952746	-4.93688558	-3.40000000
C	8.86769308	-3.54296128	-3.40000000
C	10.10610631	-2.90233747	-3.40000000
N	11.10425525	-3.85887970	-3.40000000
O	9.46043272	-0.70692493	-3.40000000
H	7.90468337	-3.06200081	-3.40000000
H	12.09691634	-3.64810104	-3.40000000
H	11.13760774	-5.98828193	-3.40000000
N	10.41165559	-1.53521454	-3.40000000
O	11.62971591	-1.19045987	-3.40000000
C	8.17579632	-5.98426822	-3.40000000
C	7.34490705	-6.87007604	-3.40000000
C	6.34586715	-7.93257553	-3.40000000
H	5.70446324	-7.87184388	-2.51134588
H	6.82401709	-8.91976538	-3.40000000
N	5.01365243	0.99199182	-0.00000000
O	4.30260090	-3.58796152	-0.00000000
N	4.56716532	-1.29195847	-0.00000000
C	4.09292299	0.00598124	-0.00000000
N	2.78699383	0.30650083	-0.00000000
C	1.99120808	-0.78584029	-0.00000000
C	2.37604854	-2.13811247	-0.00000000

C	3.76721199	-2.44848064	-0.00000000
N	0.61326544	-0.80857250	-0.00000000
C	0.22151194	-2.13729412	-0.00000000
N	1.25137639	-2.96774397	-0.00000000
H	6.02751807	0.80363867	-0.00000000
H	4.68066827	1.94733299	-0.00000000
H	5.60211828	-1.44276463	-0.00000000
H	0.00000000	-0.00000000	-0.00000000
H	-0.82262775	-2.42026635	-0.00000000
O	7.83461905	0.55738136	-0.00000000
N	7.14844079	-4.00206529	-0.00000000
N	7.46163154	-1.71656440	-0.00000000
C	9.41590662	-3.17447096	-0.00000000
C	8.27003082	-0.62421145	-0.00000000
N	9.65603357	-0.82777239	-0.00000000
C	10.21432266	-2.07245899	-0.00000000
C	7.99620203	-2.96060279	-0.00000000
H	9.83388744	-4.17428544	-0.00000000
H	10.24447549	0.00000000	-0.00000000
H	11.29744761	-2.11566317	-0.00000000
H	6.12873984	-3.85217674	-0.00000000
H	7.51095075	-4.94658058	-0.00000000

Yo–Px/G–C

Total bonding energy = -10216.36 kcal mol^{-1}

H	0.73648535	-1.70017431	3.40000000
H	7.23308988	-1.33621070	3.40000000
H	2.79775108	-3.16391546	3.40000000
C	3.29756253	3.16018684	3.40000000
C	3.73403244	4.52391509	3.40000000
C	5.10063116	4.49627928	3.40000000
O	5.54928677	3.19410775	3.40000000
C	4.42454051	2.36471911	3.40000000
H	6.83216127	1.08165185	3.40000000
H	2.27305995	2.82089387	3.40000000
H	3.10992099	5.40792769	3.40000000
H	5.87089255	5.25421767	3.40000000
C	5.97689002	0.41619133	3.40000000
C	6.20982246	-0.96499138	3.40000000
N	5.23645825	-1.90258745	3.40000000
C	3.99350770	-1.40198360	3.40000000
C	3.61282244	-0.01712739	3.40000000
C	4.66509065	0.93633913	3.40000000
N	2.83385452	-2.15119432	3.40000000
C	1.74151837	-1.30137089	3.40000000
C	2.17559113	0.01070325	3.40000000
C	10.72657033	5.19844012	3.40000000
C	9.31057857	5.14037946	3.40000000
C	8.95165993	3.76661954	3.40000000
C	10.14713718	3.04622174	3.40000000
N	11.20757875	3.93367544	3.40000000
O	9.37260585	0.89166328	3.40000000

H	7.95577035	3.35037991	3.40000000
H	12.18337688	3.65514084	3.40000000
H	11.38354263	6.05586349	3.40000000
N	10.36985955	1.66326381	3.40000000
O	11.56588748	1.24670188	3.40000000
C	8.43595739	6.25481057	3.40000000
C	7.67359143	7.20031721	3.40000000
C	6.74372706	8.32427848	3.40000000
H	6.09843904	8.30199850	4.28770667
H	6.09843904	8.30199850	2.51229333
H	1.52432866	0.87197335	3.40000000
H	7.28397113	9.27889781	3.40000000
N	5.01365243	-0.99199182	0.00000000
O	4.30260090	3.58796152	0.00000000
N	4.56716532	1.29195847	0.00000000
C	4.09292299	-0.00598124	0.00000000
N	2.78699383	-0.30650083	0.00000000
C	1.99120808	0.78584029	0.00000000
C	2.37604854	2.13811247	0.00000000
C	3.76721199	2.44848064	0.00000000
N	0.61326544	0.80857250	0.00000000
C	0.22151194	2.13729412	0.00000000
N	1.25137639	2.96774397	0.00000000
H	6.02751807	-0.80363867	0.00000000
H	4.68066827	-1.94733299	0.00000000
H	5.60211828	1.44276463	0.00000000
H	0.00000000	0.00000000	0.00000000
H	-0.82262775	2.42026635	0.00000000
O	7.83461905	-0.55738136	0.00000000
N	7.14844079	4.00206529	0.00000000
N	7.46163154	1.71656440	0.00000000
C	9.41590662	3.17447096	0.00000000
C	8.27003082	0.62421145	0.00000000
N	9.65603357	0.82777239	0.00000000
C	10.21432266	2.07245899	0.00000000
C	7.99620203	2.96060279	0.00000000
H	9.83388744	4.17428544	0.00000000
H	10.24447549	-0.00000000	0.00000000
H	11.29744761	2.11566317	0.00000000
H	6.12873984	3.85217674	0.00000000
H	7.51095075	4.94658058	0.00000000

Ps–Px//G–C

Total bonding energy = -9961.03 kcal mol^{-1}

H	0.58825288	1.58808676	-3.40000000
H	6.79732989	-0.21249637	-3.40000000
H	2.87180978	2.71644095	-3.40000000
C	1.99190091	-3.45079448	-3.40000000
C	1.88600813	-4.86748874	-3.40000000
C	3.11632936	-5.49184878	-3.40000000
S	4.42860193	-4.36092678	-3.40000000
C	3.30735893	-3.00703458	-3.40000000

H	5.30731286	-7.93307579	-4.28860261
H	1.14894463	-2.76976065	-3.40000000
H	0.94341765	-5.40608111	-3.40000000
H	3.32137847	-6.55453338	-3.40000000
N	5.18441610	-1.50088414	-3.40000000
C	5.71156544	-0.26520707	-3.40000000
N	5.05101966	0.91321339	-3.40000000
C	3.72199424	0.74817893	-3.40000000
C	3.03284726	-0.49285019	-3.40000000
C	3.82876423	-1.65662285	-3.40000000
N	2.73521787	1.71081809	-3.40000000
C	1.52672126	1.04914205	-3.40000000
N	1.65444673	-0.27103601	-3.40000000
C	10.04323741	-5.05806674	-3.40000000
C	8.63626384	-4.88501306	-3.40000000
C	8.38782630	-3.48561934	-3.40000000
C	9.64203672	-2.86669631	-3.40000000
N	10.62580199	-3.83845528	-3.40000000
O	9.08046528	-0.64400549	-3.40000000
H	7.42696872	-2.98763365	-3.40000000
H	11.62106291	-3.64026354	-3.40000000
H	10.62706824	-5.96687747	-3.40000000
N	9.99573420	-1.51144872	-3.40000000
O	11.22659361	-1.21193459	-3.40000000
C	7.69429565	-5.94310006	-3.40000000
C	6.90647927	-6.86704938	-3.40000000
C	5.95064753	-7.96853062	-3.40000000
H	5.30731286	-7.93307579	-2.51139739
H	6.46744146	-8.93596293	-3.40000000
N	5.01365243	0.99199182	-0.00000000
O	4.30260090	-3.58796152	-0.00000000
N	4.56716532	-1.29195847	-0.00000000
C	4.09292299	0.00598124	-0.00000000
N	2.78699383	0.30650083	-0.00000000
C	1.99120808	-0.78584029	-0.00000000
C	2.37604854	-2.13811247	-0.00000000
C	3.76721199	-2.44848064	-0.00000000
N	0.61326544	-0.80857250	-0.00000000
C	0.22151194	-2.13729412	-0.00000000
N	1.25137639	-2.96774397	-0.00000000
H	6.02751807	0.80363867	-0.00000000
H	4.68066827	1.94733299	-0.00000000
H	5.60211828	-1.44276463	-0.00000000
H	0.00000000	-0.00000000	-0.00000000
H	-0.82262775	-2.42026635	-0.00000000
O	7.83461905	0.55738136	-0.00000000
N	7.14844079	-4.00206529	-0.00000000
N	7.46163154	-1.71656440	-0.00000000
C	9.41590662	-3.17447096	-0.00000000
C	8.27003082	-0.62421145	-0.00000000
N	9.65603357	-0.82777239	-0.00000000
C	10.21432266	-2.07245899	-0.00000000

C	7.99620203	-2.96060279	-0.00000000
H	9.83388744	-4.17428544	-0.00000000
H	10.24447549	0.00000000	-0.00000000
H	11.29744761	-2.11566317	-0.00000000
H	6.12873984	-3.85217674	-0.00000000
H	7.51095075	-4.94658058	-0.00000000

Es–Px//G–C

Total bonding energy = -10059.00 kcal mol^{-1}

H	0.53297295	1.67460949	-3.40000000
H	6.71997679	-0.29816286	-3.40000000
H	2.87833601	2.61879523	-3.40000000
C	1.99788041	-3.65327740	-3.40000000
C	1.95858663	-5.07456093	-3.40000000
C	3.21577716	-5.63973935	-3.40000000
S	4.47119425	-4.44548631	-3.40000000
C	3.28799465	-3.14250877	-3.40000000
H	0.69680121	-1.01075182	-3.40000000
H	1.10784014	-3.03749196	-3.40000000
H	1.04091196	-5.65463216	-3.40000000
H	3.47046491	-6.69121651	-3.40000000
N	5.12136638	-1.60733665	-3.40000000
C	5.63497490	-0.36552808	-3.40000000
N	4.95566272	0.79827802	-3.40000000
C	3.62512693	0.63049558	-3.40000000
C	2.93605903	-0.62784026	-3.40000000
C	3.76816049	-1.77132564	-3.40000000
N	2.68000269	1.62464051	-3.40000000
C	1.41523679	1.04998368	-3.40000000
C	1.53016747	-0.32453608	-3.40000000
C	10.02544626	-5.14398788	-3.40000000
C	8.61622787	-4.98808791	-3.40000000
C	8.35098724	-3.59189400	-3.40000000
C	9.59780198	-2.95780616	-3.40000000
N	10.59318363	-3.91752976	-3.40000000
O	9.00941983	-0.74223276	-3.40000000
H	7.38537527	-3.10329392	-3.40000000
H	11.58603137	-3.70771576	-3.40000000
H	10.62005548	-6.04580735	-3.40000000
N	9.93506758	-1.59844359	-3.40000000
O	11.16238405	-1.28411742	-3.40000000
C	7.69064692	-6.06084451	-3.40000000
C	6.92700130	-7.00496193	-3.40000000
C	6.00517686	-8.13525689	-3.40000000
H	5.36102131	-8.12057412	-2.51146076
H	5.36102131	-8.12057412	-4.28853924
H	6.55267807	-9.08571006	-3.40000000
N	5.01365243	0.99199182	-0.00000000
O	4.30260090	-3.58796152	-0.00000000
N	4.56716532	-1.29195847	-0.00000000
C	4.09292299	0.00598124	-0.00000000
N	2.78699383	0.30650083	-0.00000000

C	1.99120808	-0.78584029	-0.00000000
C	2.37604854	-2.13811247	-0.00000000
C	3.76721199	-2.44848064	-0.00000000
N	0.61326544	-0.80857250	-0.00000000
C	0.22151194	-2.13729412	-0.00000000
N	1.25137639	-2.96774397	-0.00000000
H	6.02751807	0.80363867	-0.00000000
H	4.68066827	1.94733299	-0.00000000
H	5.60211828	-1.44276463	-0.00000000
H	0.00000000	-0.00000000	-0.00000000
H	-0.82262775	-2.42026635	-0.00000000
O	7.83461905	0.55738136	-0.00000000
N	7.14844079	-4.00206529	-0.00000000
N	7.46163154	-1.71656440	-0.00000000
C	9.41590662	-3.17447096	-0.00000000
C	8.27003082	-0.62421145	-0.00000000
N	9.65603357	-0.82777239	-0.00000000
C	10.21432266	-2.07245899	-0.00000000
C	7.99620203	-2.96060279	-0.00000000
H	9.83388744	-4.17428544	-0.00000000
H	10.24447549	0.00000000	-0.00000000
H	11.29744761	-2.11566317	-0.00000000
H	6.12873984	-3.85217674	-0.00000000
H	7.51095075	-4.94658058	-0.00000000

Ysm–Px//G–C

Total bonding energy = -10526.88 kcal mol^{-1}

H	0.94225144	-1.81487081	3.40000000
H	7.36293034	-0.76907291	3.40000000
H	3.14960136	-3.04849708	3.40000000
C	2.95407785	3.24812740	3.40000000
C	2.99892198	4.67062585	3.40000000
C	4.27600233	5.18819570	3.40000000
S	5.45210253	3.89682435	3.40000000
C	4.20159235	2.64358428	3.40000000
H	6.71663042	1.58193959	3.40000000
H	2.02441214	2.69595488	3.40000000
H	2.11374107	5.30058611	3.40000000
C	4.70736957	6.62419026	3.40000000
C	5.92666095	0.83742123	3.40000000
C	6.30626767	-0.50918218	3.40000000
N	5.43492035	-1.54195063	3.40000000
C	4.14815233	-1.16894017	3.40000000
C	3.62329432	0.17063890	3.40000000
C	4.57043160	1.23316579	3.40000000
N	3.07568247	-2.03785410	3.40000000
C	1.89891067	-1.31094548	3.40000000
C	2.18986409	0.03950226	3.40000000
C	11.16937151	5.40008156	3.40000000
C	9.75385048	5.47659657	3.40000000
C	9.26877995	4.14315768	3.40000000
C	10.38959954	3.31190960	3.40000000

N	11.52892770	4.09512218	3.40000000
O	9.41071979	1.24203723	3.40000000
H	8.23877022	3.82509475	3.40000000
H	12.47433124	3.72631574	3.40000000
H	11.90507094	6.19100872	3.40000000
N	10.47786555	1.91395653	3.40000000
O	11.62820364	1.38396767	3.40000000
C	8.98961713	6.66966759	3.40000000
C	8.33916671	7.69523816	3.40000000
C	7.56341762	8.93039871	3.40000000
H	6.92057261	8.99352390	2.51294286
H	5.31159059	6.86246930	4.28363540
H	3.82625998	7.27358674	3.40000000
H	5.31159059	6.86246930	2.51636460
H	1.44377069	0.81909927	3.40000000
H	6.92057261	8.99352390	4.28705714
H	8.22425628	9.80589451	3.40000000
N	5.01365243	-0.99199182	0.00000000
O	4.30260090	3.58796152	0.00000000
N	4.56716532	1.29195847	0.00000000
C	4.09292299	-0.00598124	0.00000000
N	2.78699383	-0.30650083	0.00000000
C	1.99120808	0.78584029	0.00000000
C	2.37604854	2.13811247	0.00000000
C	3.76721199	2.44848064	0.00000000
N	0.61326544	0.80857250	0.00000000
C	0.22151194	2.13729412	0.00000000
N	1.25137639	2.96774397	0.00000000
H	6.02751807	-0.80363867	0.00000000
H	4.68066827	-1.94733299	0.00000000
H	5.60211828	1.44276463	0.00000000
H	0.00000000	0.00000000	0.00000000
H	-0.82262775	2.42026635	0.00000000
O	7.83461905	-0.55738136	0.00000000
N	7.14844079	4.00206529	0.00000000
N	7.46163154	1.71656440	0.00000000
C	9.41590662	3.17447096	0.00000000
C	8.27003082	0.62421145	0.00000000
N	9.65603357	0.82777239	0.00000000
C	10.21432266	2.07245899	0.00000000
C	7.99620203	2.96060279	0.00000000
H	9.83388744	4.17428544	0.00000000
H	10.24447549	-0.00000000	0.00000000
H	11.29744761	2.11566317	0.00000000
H	6.12873984	3.85217674	0.00000000
H	7.51095075	4.94658058	0.00000000

Yss–Px//G–C

Total bonding energy = -11189.34 kcal mol^{-1}

H	1.34421292	2.05010415	-3.40000000
H	7.71655353	0.74021916	-3.40000000
H	3.60058918	3.19080888	-3.40000000

C	3.14909318	-3.08954231	-3.40000000
C	3.12724784	-4.50286820	-3.40000000
C	4.38915645	-5.08227681	-3.40000000
S	5.62766917	-3.83686504	-3.40000000
C	4.42395165	-2.53829821	-3.40000000
H	6.97286198	-1.58311654	-3.40000000
H	2.24551520	-2.49614222	-3.40000000
H	2.21176592	-5.08637492	-3.40000000
C	4.74045235	-6.48170129	-3.40000000
C	6.21608108	-0.80553299	-3.40000000
C	6.64995325	0.52381889	-3.40000000
N	5.82098323	1.59152766	-3.40000000
C	4.52021719	1.27143908	-3.40000000
C	3.93998224	-0.04556954	-3.40000000
C	4.84476572	-1.14536263	-3.40000000
N	3.48474009	2.18408935	-3.40000000
C	2.27911331	1.50699290	-3.40000000
C	2.51380950	0.14525451	-3.40000000
C	11.98637971	-5.47527910	-3.40000000
C	10.62461211	-5.87001039	-3.40000000
C	9.84981463	-4.68039842	-3.40000000
C	10.75666419	-3.61779235	-3.40000000
N	12.04243682	-4.12416078	-3.40000000
O	9.35065449	-1.81039347	-3.40000000
H	8.77381025	-4.59242100	-3.40000000
H	12.88001410	-3.55108795	-3.40000000
H	12.88044082	-6.08134892	-3.40000000
N	10.53695225	-2.23417154	-3.40000000
O	11.54472066	-1.46657511	-3.40000000
C	10.17582782	-7.21389816	-3.40000000
C	9.85468625	-8.38530398	-3.40000000
C	9.49275064	-9.79864837	-3.40000000
C	5.99548533	-7.07517733	-3.40000000
C	5.94868269	-8.49728899	-3.40000000
C	4.66470886	-8.99292232	-3.40000000
S	3.48432361	-7.71971142	-3.40000000
H	6.92053384	-6.50764327	-3.40000000
H	8.90374641	-10.05839605	-4.28875568
H	8.90374641	-10.05839605	-2.51124432
H	6.82690998	-9.13144048	-3.40000000
H	4.33655243	-10.02452008	-3.40000000
H	1.73530554	-0.60198342	-3.40000000
H	10.39197570	-10.42693365	-3.40000000
N	5.01365243	0.99199182	-0.00000000
O	4.30260090	-3.58796152	-0.00000000
N	4.56716532	-1.29195847	-0.00000000
C	4.09292299	0.00598124	-0.00000000
N	2.78699383	0.30650083	-0.00000000
C	1.99120808	-0.78584029	-0.00000000
C	2.37604854	-2.13811247	-0.00000000
C	3.76721199	-2.44848064	-0.00000000
N	0.61326544	-0.80857250	-0.00000000

C	0.22151194	-2.13729412	-0.00000000
N	1.25137639	-2.96774397	-0.00000000
H	6.02751807	0.80363867	-0.00000000
H	4.68066827	1.94733299	-0.00000000
H	5.60211828	-1.44276463	-0.00000000
H	0.00000000	-0.00000000	-0.00000000
H	-0.82262775	-2.42026635	-0.00000000
O	7.83461905	0.55738136	-0.00000000
N	7.14844079	-4.00206529	-0.00000000
N	7.46163154	-1.71656440	-0.00000000
C	9.41590662	-3.17447096	-0.00000000
C	8.27003082	-0.62421145	-0.00000000
N	9.65603357	-0.82777239	-0.00000000
C	10.21432266	-2.07245899	-0.00000000
C	7.99620203	-2.96060279	-0.00000000
H	9.83388744	-4.17428544	-0.00000000
H	10.24447549	0.00000000	-0.00000000
H	11.29744761	-2.11566317	-0.00000000
H	6.12873984	-3.85217674	-0.00000000
H	7.51095075	-4.94658058	-0.00000000

S12. Benchmark analysis of Ds–Px and Es–Px Base Pairs

Cartesian coordinates (in Å) of Ds-Px and Es-Px base pairs, together with Ds, Es and Px bases alone, optimized at different levels of theory, and only constrained in C_s symmetry. ADF total energies (in kcal mol^{-1}) included in parentheses. Computed at different levels of theory in gas phase.

Ds-Px

BLYP-D3(BJ)/TZ2P (-5608.35)

1	H	6.90640985	-0.97313142	0.00000000
2	H	2.01381217	3.28015157	0.00000000
3	H	6.55461732	1.57664121	0.00000000
4	C	2.11505685	-2.68417554	0.00000000
5	C	1.08184570	-3.66123572	0.00000000
6	C	-0.17930837	-3.11031654	0.00000000
7	S	-0.12245010	-1.37903198	0.00000000
8	C	1.64106964	-1.38071599	0.00000000
9	H	0.70700236	1.22762427	0.00000000
10	H	3.17669467	-2.90169428	0.00000000
11	H	1.26255235	-4.73188206	0.00000000
12	H	-1.13625369	-3.61413888	0.00000000
13	C	1.78844568	1.13680117	0.00000000
14	C	2.53458530	2.32506162	0.00000000
15	N	3.88418750	2.37710697	0.00000000
16	C	4.45030890	1.17044656	0.00000000
17	C	3.81433221	-0.10216214	0.00000000
18	C	2.39796795	-0.13715289	0.00000000
19	N	5.81240197	0.88673345	0.00000000
20	C	5.93694440	-0.48909962	0.00000000
21	N	4.77418147	-1.11581455	0.00000000
22	C	-4.74270942	1.72332349	0.00000000
23	C	-3.70977621	0.76419694	0.00000000
24	C	-2.47791291	1.48285806	0.00000000
25	C	-2.80093400	2.83114314	0.00000000
26	N	-4.17540845	2.96527595	0.00000000
27	O	-0.73178570	3.81302189	0.00000000
28	H	-1.48611829	1.06378282	0.00000000
29	H	-4.63875334	3.86751048	0.00000000
30	H	-5.81465550	1.58647569	0.00000000
31	N	-1.96904896	3.98552139	0.00000000
32	O	-2.54189977	5.10583781	0.00000000
33	C	-3.86571145	-0.64278287	0.00000000
34	C	-3.97121722	-1.85067903	0.00000000
35	C	-4.09561990	-3.30306788	0.00000000
36	H	-3.62159186	-3.74164895	0.88853840
37	H	-5.14966930	-3.60913519	0.00000000
38	H	-3.62159186	-3.74164895	-0.88853840

M06-2X/TZ2P (-8219.50)

1	H	6.85262473	-0.95352295	0.00000000
2	H	2.00880785	3.25601904	0.00000000
3	H	6.50580273	1.56735479	0.00000000
4	C	2.11490924	-2.65942536	0.00000000
5	C	1.08390349	-3.63356470	0.00000000
6	C	-0.15888840	-3.07980864	0.00000000
7	S	-0.09257705	-1.37488679	0.00000000
8	C	1.63638386	-1.37683611	0.00000000
9	H	0.70836010	1.21494696	0.00000000
10	H	3.17098637	-2.87682776	0.00000000
11	H	1.26034265	-4.69818886	0.00000000
12	H	-1.11348813	-3.57801027	0.00000000

13	C	1.78499487	1.12501129	0.00000000
14	C	2.52894962	2.30706220	0.00000000
15	N	3.85682700	2.35721004	0.00000000
16	C	4.42097494	1.16263824	0.00000000
17	C	3.79508916	-0.09488774	0.00000000
18	C	2.39284855	-0.12870245	0.00000000
19	N	5.76758488	0.88498734	0.00000000
20	C	5.88711875	-0.47322603	0.00000000
21	N	4.74674388	-1.09755130	0.00000000
22	C	-4.69949191	1.71135777	0.00000000
23	C	-3.68252076	0.76762171	0.00000000
24	C	-2.45775348	1.47582847	0.00000000
25	C	-2.78772917	2.80171569	0.00000000
26	N	-4.14103496	2.94206648	0.00000000
27	O	-0.75205243	3.77868822	0.00000000
28	H	-1.46793404	1.06184814	0.00000000
29	H	-4.60473514	3.83642573	0.00000000
30	H	-5.76569446	1.57320005	0.00000000
31	N	-1.95276518	3.95414652	0.00000000
32	O	-2.51030326	5.03925249	0.00000000
33	C	-3.83664752	-0.64624190	0.00000000
34	C	-3.94942807	-1.84036536	0.00000000
35	C	-4.10409926	-3.28997585	0.00000000
36	H	-3.64353681	-3.72795565	0.88503019
37	H	-5.15903580	-3.55944783	0.00000000
38	H	-3.64353681	-3.72795565	-0.88503019

wB97X-D/TZ2P (-9491.80)

1	H	6.78411445	-0.98629880	0.00000000
2	H	1.99065578	3.28423439	0.00000000
3	H	6.46676010	1.53797721	0.00000000
4	C	2.02725871	-2.62749125	0.00000000
5	C	0.98415388	-3.58888552	0.00000000
6	C	-0.24906454	-3.01969621	0.00000000
7	S	-0.15919043	-1.32063946	0.00000000
8	C	1.56264561	-1.34302146	0.00000000
9	H	0.66753862	1.26437719	0.00000000
10	H	3.08226144	-2.85704374	0.00000000
11	H	1.14822076	-4.65688156	0.00000000
12	H	-1.21025799	-3.50827283	0.00000000
13	C	1.74370404	1.15678826	0.00000000
14	C	2.50138242	2.32788535	0.00000000
15	N	3.82799250	2.36081015	0.00000000
16	C	4.37794664	1.15986079	0.00000000
17	C	3.73623168	-0.08433180	0.00000000
18	C	2.33627206	-0.10178433	0.00000000
19	N	5.72090406	0.86643927	0.00000000
20	C	5.82386465	-0.49216008	0.00000000
21	N	4.67739171	-1.10085924	0.00000000
22	C	-4.63640628	1.67931713	0.00000000
23	C	-3.61668333	0.74494771	0.00000000
24	C	-2.39626229	1.46096373	0.00000000
25	C	-2.73111414	2.78261377	0.00000000
26	N	-4.08566832	2.91359399	0.00000000
27	O	-0.69787118	3.76737690	0.00000000
28	H	-1.40586348	1.04696881	0.00000000
29	H	-4.55732360	3.80152538	0.00000000
30	H	-5.70333437	1.53480126	0.00000000
31	N	-1.89935927	3.93739162	0.00000000
32	O	-2.45839799	5.02132757	0.00000000

33	C	-3.75882022	-0.67248075	0.00000000
34	C	-3.85567626	-1.86582111	0.00000000
35	C	-3.98210247	-3.31802170	0.00000000
36	H	-3.51217571	-3.74725960	0.88583278
37	H	-5.03155152	-3.61099146	0.00000000
38	H	-3.51217571	-3.74725960	-0.88583278

B3LYP-D/TZ2P (-6751.37)

1	H	6.86643895	-0.96224648	0.00000000
2	H	2.00520686	3.25566736	0.00000000
3	H	6.51568373	1.56592212	0.00000000
4	C	2.11097806	-2.66748282	0.00000000
5	C	1.08204150	-3.64114112	0.00000000
6	C	-0.16749197	-3.09172422	0.00000000
7	S	-0.10707892	-1.37665929	0.00000000
8	C	1.63632359	-1.37707273	0.00000000
9	H	0.70842688	1.21474183	0.00000000
10	H	3.16662497	-2.88485800	0.00000000
11	H	1.26202319	-4.70579318	0.00000000
12	H	-1.11918419	-3.59391269	0.00000000
13	C	1.78388523	1.12512308	0.00000000
14	C	2.52548602	2.30685454	0.00000000
15	N	3.86059780	2.35653986	0.00000000
16	C	4.42717300	1.16217394	0.00000000
17	C	3.79727042	-0.09990559	0.00000000
18	C	2.39194106	-0.13624810	0.00000000
19	N	5.77799695	0.88241516	0.00000000
20	C	5.90168402	-0.48093760	0.00000000
21	N	4.75301902	-1.10317102	0.00000000
22	C	-4.71566396	1.71828088	0.00000000
23	C	-3.69446125	0.76719403	0.00000000
24	C	-2.46878529	1.47873110	0.00000000
25	C	-2.79139533	2.81467464	0.00000000
26	N	-4.15309354	2.95024246	0.00000000
27	O	-0.74553717	3.78781573	0.00000000
28	H	-1.48217984	1.06118854	0.00000000
29	H	-4.61669765	3.84403475	0.00000000
30	H	-5.78197830	1.58120452	0.00000000
31	N	-1.96077879	3.96032541	0.00000000
32	O	-2.52173373	5.06071383	0.00000000
33	C	-3.85032550	-0.63830143	0.00000000
34	C	-3.95694879	-1.83519999	0.00000000
35	C	-4.08293604	-3.28259922	0.00000000
36	H	-3.61233826	-3.71701448	0.88425620
37	H	-5.13185443	-3.58256138	0.00000000
38	H	-3.61233826	-3.71701448	-0.88425620

BLYP-D3(BJ)/QZ4P (-5626.44)

1	H	6.90648088	-0.97212456	0.00000000
2	H	2.01502086	3.28111586	0.00000000
3	H	6.55416214	1.57677412	0.00000000
4	C	2.11455923	-2.68404207	0.00000000
5	C	1.08132470	-3.66123623	0.00000000
6	C	-0.18022355	-3.10917090	0.00000000
7	S	-0.12149724	-1.38005020	0.00000000
8	C	1.63995750	-1.37983914	0.00000000
9	H	0.70802364	1.22876467	0.00000000
10	H	3.17553486	-2.90135805	0.00000000
11	H	1.26188310	-4.73096424	0.00000000
12	H	-1.13646883	-3.61256000	0.00000000

13	C	1.78880519	1.13830285	0.00000000
14	C	2.53498540	2.32667065	0.00000000
15	N	3.88517177	2.37846044	0.00000000
16	C	4.45069226	1.17104058	0.00000000
17	C	3.81444325	-0.10162036	0.00000000
18	C	2.39775082	-0.13629077	0.00000000
19	N	5.81305020	0.88751163	0.00000000
20	C	5.93796089	-0.48846454	0.00000000
21	N	4.77467730	-1.11578869	0.00000000
22	C	-4.74219875	1.72258782	0.00000000
23	C	-3.70898276	0.76310911	0.00000000
24	C	-2.47686809	1.48188771	0.00000000
25	C	-2.80015101	2.83077121	0.00000000
26	N	-4.17479624	2.96478747	0.00000000
27	O	-0.73219958	3.81350647	0.00000000
28	H	-1.48586134	1.06278953	0.00000000
29	H	-4.63755562	3.86598970	0.00000000
30	H	-5.81339881	1.58639750	0.00000000
31	N	-1.96802246	3.98541682	0.00000000
32	O	-2.53962141	5.10486734	0.00000000
33	C	-3.86471541	-0.64407273	0.00000000
34	C	-3.97070047	-1.85214358	0.00000000
35	C	-4.09823154	-3.30412350	0.00000000
36	H	-3.62540560	-3.74421852	0.88768757
37	H	-5.15217967	-3.60846490	0.00000000
38	H	-3.62540560	-3.74421852	-0.88768757

Ds

BLYP-D3(BJ)/TZ2P (-3166.82)

1	H	6.86713855	-0.63597620	0.00000000
2	H	1.83437061	3.44406752	0.00000000
3	H	6.42695764	1.89964821	0.00000000
4	C	2.14055686	-2.51319658	0.00000000
5	C	1.14486891	-3.52744637	0.00000000
6	C	-0.13526251	-3.02192699	0.00000000
7	S	-0.14400521	-1.28928608	0.00000000
8	C	1.61922961	-1.22765570	0.00000000
9	H	0.59039530	1.33122756	0.00000000
10	H	3.20950094	-2.69112614	0.00000000
11	H	1.36400153	-4.59076630	0.00000000
12	H	-1.07365514	-3.56208988	0.00000000
13	C	1.67655829	1.29151430	0.00000000
14	C	2.38271970	2.50324774	0.00000000
15	N	3.72900961	2.60598342	0.00000000
16	C	4.33811528	1.42025120	0.00000000
17	C	3.74682687	0.12591487	0.00000000
18	C	2.33256241	0.03986001	0.00000000
19	N	5.70881496	1.18466070	0.00000000
20	C	5.88135946	-0.18622303	0.00000000
21	N	4.74132331	-0.85293717	0.00000000

M06-2X/TZ2P (-4588.64)

1	H	6.82554009	-0.62832548	0.00000000
2	H	1.84595594	3.41228252	0.00000000
3	H	6.39356670	1.87904590	0.00000000
4	C	2.14971758	-2.49594808	0.00000000
5	C	1.15329830	-3.50469790	0.00000000
6	C	-0.10718537	-2.99236902	0.00000000
7	S	-0.10076538	-1.28594232	0.00000000
8	C	1.62741078	-1.23029302	0.00000000

9	H	0.60728715	1.31400391	0.00000000
10	H	3.21253450	-2.67689345	0.00000000
11	H	1.36519002	-4.56263745	0.00000000
12	H	-1.04341977	-3.52459126	0.00000000
13	C	1.68753520	1.27323395	0.00000000
14	C	2.39279207	2.47792190	0.00000000
15	N	3.71755304	2.57700724	0.00000000
16	C	4.32370620	1.40324565	0.00000000
17	C	3.74083249	0.12482133	0.00000000
18	C	2.34092915	0.04151856	0.00000000
19	N	5.67864020	1.17232056	0.00000000
20	C	5.84419786	-0.18141999	0.00000000
21	N	4.72607020	-0.84453843	0.00000000

wB97X-D/TZ2P (-5313.25)

1	H	6.82525764	-0.63118810	0.00000000
2	H	1.84532709	3.41121060	0.00000000
3	H	6.39093234	1.87531271	0.00000000
4	C	2.14966506	-2.49129602	0.00000000
5	C	1.15367045	-3.50126530	0.00000000
6	C	-0.10401487	-2.98898731	0.00000000
7	S	-0.09717452	-1.28697512	0.00000000
8	C	1.62514351	-1.23027394	0.00000000
9	H	0.60637382	1.31256778	0.00000000
10	H	3.21436486	-2.67000730	0.00000000
11	H	1.36681342	-4.56033371	0.00000000
12	H	-1.04016720	-3.52419951	0.00000000
13	C	1.68799409	1.27218094	0.00000000
14	C	2.39236855	2.47519664	0.00000000
15	N	3.71551810	2.57284296	0.00000000
16	C	4.32222733	1.39995848	0.00000000
17	C	3.73886885	0.12690736	0.00000000
18	C	2.34188765	0.04343759	0.00000000
19	N	5.67691533	1.16997969	0.00000000
20	C	5.84294101	-0.18266795	0.00000000
21	N	4.72647444	-0.84465538	0.00000000

B3LYP-D/TZ2P (-3792.33)

1	H	6.83708012	-0.63289621	0.00000000
2	H	1.84212413	3.41822841	0.00000000
3	H	6.40188285	1.88177330	0.00000000
4	C	2.14531210	-2.49772782	0.00000000
5	C	1.15306100	-3.50766957	0.00000000
6	C	-0.11496021	-3.00232441	0.00000000
7	S	-0.11842593	-1.28573839	0.00000000
8	C	1.62497944	-1.22485957	0.00000000
9	H	0.60513434	1.31991986	0.00000000
10	H	3.20804900	-2.67664918	0.00000000
11	H	1.37056427	-4.56510338	0.00000000
12	H	-1.04820630	-3.53978507	0.00000000
13	C	1.68505093	1.27916851	0.00000000
14	C	2.38845524	2.48327262	0.00000000
15	N	3.72034687	2.58162605	0.00000000
16	C	4.32816578	1.40771462	0.00000000
17	C	3.74100642	0.12496570	0.00000000
18	C	2.33791601	0.03929864	0.00000000
19	N	5.68723484	1.17420886	0.00000000
20	C	5.85665758	-0.18451610	0.00000000
21	N	4.72995849	-0.84516175	0.00000000

BLYP-D3(BJ)/QZ4P (-3177.45)

1	H	6.86728347	-0.63474360	0.00000000
2	H	1.83475067	3.44378710	0.00000000
3	H	6.42588286	1.89986039	0.00000000
4	C	2.14045646	-2.51458858	0.00000000
5	C	1.14439075	-3.52841339	0.00000000
6	C	-0.13614183	-3.02118085	0.00000000
7	S	-0.14190964	-1.29109270	0.00000000
8	C	1.61874441	-1.22805901	0.00000000
9	H	0.59107672	1.33129882	0.00000000
10	H	3.20860738	-2.69283256	0.00000000
11	H	1.36317195	-4.59071989	0.00000000
12	H	-1.07389370	-3.56034388	0.00000000
13	C	1.67638333	1.29177671	0.00000000
14	C	2.38237054	2.50378084	0.00000000
15	N	3.72919485	2.60677162	0.00000000
16	C	4.33802349	1.42029939	0.00000000
17	C	3.74682747	0.12600235	0.00000000
18	C	2.33240640	0.03964087	0.00000000
19	N	5.70915495	1.18542822	0.00000000
20	C	5.88244040	-0.18559808	0.00000000
21	N	4.74216602	-0.85332868	0.00000000

Px

BLYP-D3(BJ)/TZ2P (-2437.64)

1	C	-1.36341372	-3.84182990	0.00000000
2	H	-0.98473265	-4.36706296	0.88712736
3	H	-2.45765575	-3.92982949	0.00000000
4	H	-0.98473265	-4.36706296	-0.88712736
5	C	-1.02534710	1.19991619	0.00000000
6	C	-0.18266991	0.07048664	0.00000000
7	C	1.16073892	0.55460264	0.00000000
8	C	1.08357640	1.94028916	0.00000000
9	N	-0.24385352	2.31861559	0.00000000
10	O	3.29130033	2.55650606	0.00000000
11	H	2.06796225	-0.03075735	0.00000000
12	H	-0.53432443	3.29048273	0.00000000
13	H	-2.10442079	1.25757997	0.00000000
14	N	2.10294085	2.93557160	0.00000000
15	O	1.72684587	4.13758043	0.00000000
16	C	-0.60141195	-1.28181390	0.00000000
17	C	-0.95080218	-2.44327447	0.00000000

M06-2X/TZ2P (-3629.10)

1	C	-1.35795808	-3.82208419	0.00000000
2	H	-0.98068295	-4.33665267	0.88279028
3	H	-2.44427959	-3.89705203	0.00000000
4	H	-0.98068295	-4.33665267	-0.88279028
5	C	-1.00733918	1.19250857	0.00000000
6	C	-0.17657637	0.08153351	0.00000000
7	C	1.15766023	0.55770385	0.00000000
8	C	1.06847220	1.92287177	0.00000000
9	N	-0.23778901	2.30215760	0.00000000
10	O	3.24026290	2.54484740	0.00000000
11	H	2.06410283	-0.01969824	0.00000000
12	H	-0.53133648	3.26586638	0.00000000
13	H	-2.08106781	1.24655882	0.00000000
14	N	2.08751326	2.91797412	0.00000000
15	O	1.71827913	4.08239505	0.00000000
16	C	-0.59720483	-1.27678415	0.00000000

17	C	-0.94137334	-2.42549312	0.00000000

wB97X-D/TZ2P (-4172.40)

1	C	-1.35386420	-3.82056105	0.00000000
2	H	-0.97521847	-4.33464282	0.88336081
3	H	-2.44074099	-3.89913597	0.00000000
4	H	-0.97521847	-4.33464282	-0.88336081
5	C	-1.00944277	1.19367336	0.00000000
6	C	-0.18193310	0.08585248	0.00000000
7	C	1.15283303	0.56002092	0.00000000
8	C	1.06724688	1.92281345	0.00000000
9	N	-0.23874143	2.30269477	0.00000000
10	O	3.24168142	2.53788157	0.00000000
11	H	2.05781696	-0.02233423	0.00000000
12	H	-0.53410015	3.26381099	0.00000000
13	H	-2.08462823	1.24895664	0.00000000
14	N	2.09024725	2.91400514	0.00000000
15	O	1.72666370	4.07967757	0.00000000
16	C	-0.60094930	-1.27529069	0.00000000
17	C	-0.94165219	-2.42277932	0.00000000

B3LYP-D/TZ2P (-2955.13)

1	C	-1.35510149	-3.82002716	0.00000000
2	H	-0.97874524	-4.34051877	0.88273363
3	H	-2.44319137	-3.90315865	0.00000000
4	H	-0.97874524	-4.34051877	-0.88273363
5	C	-1.01452643	1.19455357	0.00000000
6	C	-0.18068146	0.07594551	0.00000000
7	C	1.15451890	0.55563254	0.00000000
8	C	1.07427715	1.92896465	0.00000000
9	N	-0.24075856	2.30499976	0.00000000
10	O	3.25664973	2.54090915	0.00000000
11	H	2.05784676	-0.02584166	0.00000000
12	H	-0.53439785	3.26815618	0.00000000
13	H	-2.08816963	1.24994747	0.00000000
14	N	2.08976866	2.91619601	0.00000000
15	O	1.72170637	4.09651123	0.00000000
16	C	-0.59828432	-1.27499639	0.00000000
17	C	-0.94216600	-2.42675468	0.00000000

BLYP-D3(BJ)/QZ4P (-2445.38)

1	C	-1.36437245	-3.84212321	0.00000000
2	H	-0.98618182	-4.36771100	0.88657379
3	H	-2.45815403	-3.93024680	0.00000000
4	H	-0.98618182	-4.36771100	-0.88657379
5	C	-1.02486032	1.19945304	0.00000000
6	C	-0.18168441	0.06998392	0.00000000
7	C	1.16174460	0.55473289	0.00000000
8	C	1.08386551	1.94092430	0.00000000
9	N	-0.24382369	2.31867618	0.00000000
10	O	3.28999933	2.55915879	0.00000000
11	H	2.06873162	-0.02988183	0.00000000
12	H	-0.53442971	3.28956109	0.00000000
13	H	-2.10340245	1.25689753	0.00000000
14	N	2.10269653	2.93675582	0.00000000
15	O	1.72768498	4.13785259	0.00000000
16	C	-0.60022258	-1.28253834	0.00000000
17	C	-0.95140933	-2.44378396	0.00000000

Es-Px

BLYP-D3(BJ)/TZ2P (-5607.06)

1	H	-0.28472303	6.87645512	0.00000000
2	H	2.91358210	1.22356040	0.00000000
3	H	2.09209391	5.95086007	0.00000000
4	C	-2.83250128	1.95871967	0.00000000
5	C	-3.82055943	0.93754741	0.00000000
6	C	-3.27212391	-0.32694617	0.00000000
7	S	-1.54112154	-0.29340991	0.00000000
8	C	-1.53690234	1.46362252	0.00000000
9	H	-1.98745295	4.80113944	0.00000000
10	H	-3.07026866	3.01469517	0.00000000
11	H	-4.88951418	1.12808201	0.00000000
12	H	-3.79395478	-1.27418694	0.00000000
13	N	0.84803083	1.34958864	0.00000000
14	C	2.07216492	1.91071966	0.00000000
15	N	2.36055751	3.22533972	0.00000000
16	C	1.27126432	3.99750124	0.00000000
17	C	-0.08897470	3.55225899	0.00000000
18	C	-0.25312836	2.14535706	0.00000000
19	N	1.25892585	5.37569797	0.00000000
20	C	-0.05971747	5.81868158	0.00000000
21	C	-0.90996299	4.73420618	0.00000000
22	C	1.83624118	-4.55614389	0.00000000
23	C	0.92763631	-3.47789342	0.00000000
24	C	1.70022437	-2.27441465	0.00000000
25	C	3.03374688	-2.67199762	0.00000000
26	N	3.10063449	-4.05188605	0.00000000
27	O	4.18406664	-0.68391699	0.00000000
28	H	1.33955660	-1.25294197	0.00000000
29	H	3.98272177	-4.55263310	0.00000000
30	H	1.64332857	-5.61945617	0.00000000
31	N	4.24843176	-1.93004558	0.00000000
32	O	5.32146944	-2.59116733	0.00000000
33	C	-0.48082176	-3.62948485	0.00000000
34	C	-1.67696912	-3.82780212	0.00000000
35	C	-3.11589404	-4.06222295	0.00000000
36	H	-3.59076787	-3.62516380	0.88882571
37	H	-3.59076787	-3.62516380	-0.88882571
38	H	-3.33855131	-5.13715553	0.00000000

M06-2X/TZ2P (-8217.47)

1	H	-0.34840487	6.84266686	0.00000000
2	H	2.97940469	1.33828740	0.00000000
3	H	2.03106723	5.98475271	0.00000000
4	C	-2.73440458	1.90461658	0.00000000
5	C	-3.69483339	0.86362937	0.00000000
6	C	-3.11440987	-0.36973034	0.00000000
7	S	-1.41281410	-0.28429219	0.00000000
8	C	-1.44772849	1.43702168	0.00000000
9	H	-1.98771234	4.75573089	0.00000000
10	H	-2.99323190	2.94989441	0.00000000
11	H	-4.76179912	1.02511228	0.00000000
12	H	-3.60480008	-1.32851002	0.00000000
13	N	0.92503021	1.40519424	0.00000000
14	C	2.12233787	1.99734166	0.00000000
15	N	2.36571841	3.29821471	0.00000000
16	C	1.26319775	4.03283470	0.00000000
17	C	-0.06498133	3.55463396	0.00000000

18	C	-0.17799252	2.15680251	0.00000000
19	N	1.21819095	5.39393968	0.00000000
20	C	-0.09670199	5.79645354	0.00000000
21	C	-0.91528242	4.71001893	0.00000000
22	C	1.77127796	-4.52583787	0.00000000
23	C	0.87747366	-3.46395786	0.00000000
24	C	1.64077194	-2.26779790	0.00000000
25	C	2.95012351	-2.67170454	0.00000000
26	N	3.02500131	-4.03074509	0.00000000
27	O	4.07706280	-0.71051642	0.00000000
28	H	1.29327583	-1.24608870	0.00000000
29	H	3.89730675	-4.53434030	0.00000000
30	H	1.57652867	-5.58323239	0.00000000
31	N	4.15854356	-1.92006954	0.00000000
32	O	5.20185782	-2.55530719	0.00000000
33	C	-0.53765908	-3.61917279	0.00000000
34	C	-1.72114361	-3.81667714	0.00000000
35	C	-3.15637571	-4.07476355	0.00000000
36	H	-3.62781508	-3.64884863	0.88558474
37	H	-3.62781508	-3.64884863	-0.88558474
38	H	-3.34826546	-5.14670506	0.00000000

wB97X-D/TZ2P (-9490.23)

1	H	-0.34414681	6.80655864	0.00000000
2	H	2.97778106	1.29594267	0.00000000
3	H	2.03478695	5.94508070	0.00000000
4	C	-2.72990566	1.87997941	0.00000000
5	C	-3.69407656	0.84241769	0.00000000
6	C	-3.11773249	-0.39010026	0.00000000
7	S	-1.42051701	-0.30900175	0.00000000
8	C	-1.44845068	1.40606502	0.00000000
9	H	-1.98330138	4.71828095	0.00000000
10	H	-2.98527257	2.92756179	0.00000000
11	H	-4.76177819	1.00774875	0.00000000
12	H	-3.61412980	-1.34725401	0.00000000
13	N	0.92385622	1.36949511	0.00000000
14	C	2.12185139	1.95916745	0.00000000
15	N	2.36737571	3.25706033	0.00000000
16	C	1.26662339	3.99571165	0.00000000
17	C	-0.05694341	3.51800773	0.00000000
18	C	-0.17500414	2.12446047	0.00000000
19	N	1.22336655	5.35506673	0.00000000
20	C	-0.09232397	5.75881136	0.00000000
21	C	-0.90886586	4.67559806	0.00000000
22	C	1.76836309	-4.48982127	0.00000000
23	C	0.87779506	-3.43097757	0.00000000
24	C	1.64086142	-2.23401028	0.00000000
25	C	2.94860643	-2.63577940	0.00000000
26	N	3.02196353	-3.99519385	0.00000000
27	O	4.08087209	-0.67541812	0.00000000
28	H	1.28998863	-1.21160742	0.00000000
29	H	3.89087910	-4.50074565	0.00000000
30	H	1.57310113	-5.54867565	0.00000000
31	N	4.15759404	-1.88505530	0.00000000
32	O	5.20051573	-2.52021040	0.00000000
33	C	-0.53947184	-3.58687425	0.00000000
34	C	-1.72048369	-3.78346750	0.00000000
35	C	-3.15836112	-4.02391041	0.00000000
36	H	-3.62440093	-3.59076432	0.88600735
37	H	-3.62440093	-3.59076432	-0.88600735

38	H	-3.36661459	-5.09338279	0.00000000

B3LYP-D/TZ2P (-6749.90)

1	H	-0.28330591	6.83650818	0.00000000
2	H	2.89285856	1.22418438	0.00000000
3	H	2.07648594	5.91446680	0.00000000
4	C	-2.81124841	1.95170081	0.00000000
5	C	-3.79671862	0.93653445	0.00000000
6	C	-3.25101512	-0.31720127	0.00000000
7	S	-1.53699430	-0.28122946	0.00000000
8	C	-1.52879306	1.45511326	0.00000000
9	H	-1.97678766	4.78141521	0.00000000
10	H	-3.04592849	3.00233227	0.00000000
11	H	-4.85947911	1.12724648	0.00000000
12	H	-3.77140506	-1.25901694	0.00000000
13	N	0.84145082	1.35307769	0.00000000
14	C	2.05632192	1.90825052	0.00000000
15	N	2.33886806	3.20874070	0.00000000
16	C	1.25966116	3.97780452	0.00000000
17	C	-0.08788395	3.53464148	0.00000000
18	C	-0.24826761	2.13843654	0.00000000
19	N	1.24922082	5.34379629	0.00000000
20	C	-0.05882965	5.78427666	0.00000000
21	C	-0.90530903	4.71273694	0.00000000
22	C	1.83288436	-4.53376804	0.00000000
23	C	0.93021585	-3.46899787	0.00000000
24	C	1.69391807	-2.27052440	0.00000000
25	C	3.01544610	-2.66459480	0.00000000
26	N	3.08662043	-4.03178250	0.00000000
27	O	4.14900751	-0.69607866	0.00000000
28	H	1.33175187	-1.25550465	0.00000000
29	H	3.95997146	-4.53267839	0.00000000
30	H	1.64210295	-5.59180525	0.00000000
31	N	4.21906614	-1.92030049	0.00000000
32	O	5.27464106	-2.56493436	0.00000000
33	C	-0.47657078	-3.62155501	0.00000000
34	C	-1.66275095	-3.81368753	0.00000000
35	C	-3.09754130	-4.04295288	0.00000000
36	H	-3.56597398	-3.60674267	0.88457699
37	H	-3.56597398	-3.60674267	-0.88457699
38	H	-3.31971623	-5.11116534	0.00000000

BLYP-D3(BJ)/QZ4P (-5625.25)

1	H	-0.28182099	6.87628211	0.00000000
2	H	2.91170490	1.22178192	0.00000000
3	H	2.09220379	5.94871268	0.00000000
4	C	-2.83461541	1.95861953	0.00000000
5	C	-3.82254988	0.93715524	0.00000000
6	C	-3.27297255	-0.32767255	0.00000000
7	S	-1.54392965	-0.29181745	0.00000000
8	C	-1.53845497	1.46301609	0.00000000
9	H	-1.98688171	4.80150634	0.00000000
10	H	-3.07218423	3.01391719	0.00000000
11	H	-4.89059117	1.12751015	0.00000000
12	H	-3.79418586	-1.27427023	0.00000000
13	N	0.84654516	1.34782996	0.00000000
14	C	2.07108660	1.90870559	0.00000000
15	N	2.36071058	3.22370693	0.00000000
16	C	1.27108207	3.99615817	0.00000000
17	C	-0.08958777	3.55184088	0.00000000

18	C	-0.25437677	2.14475798	0.00000000
19	N	1.25978326	5.37460572	0.00000000
20	C	-0.05884847	5.81897212	0.00000000
21	C	-0.91019446	4.73476323	0.00000000
22	C	1.83697547	-4.55604951	0.00000000
23	C	0.92852994	-3.47702431	0.00000000
24	C	1.70190996	-2.27385148	0.00000000
25	C	3.03568163	-2.67225221	0.00000000
26	N	3.10197585	-4.05228758	0.00000000
27	O	4.18457586	-0.68491573	0.00000000
28	H	1.34131557	-1.25331253	0.00000000
29	H	3.98246620	-4.55341404	0.00000000
30	H	1.64442099	-5.61856701	0.00000000
31	N	4.25001913	-1.92952048	0.00000000
32	O	5.32267768	-2.58839756	0.00000000
33	C	-0.48020651	-3.62764040	0.00000000
34	C	-1.67669276	-3.82633488	0.00000000
35	C	-3.11535892	-4.06058909	0.00000000
36	H	-3.59063082	-3.62361473	0.88796528
37	H	-3.59063082	-3.62361473	-0.88796528
38	H	-3.33895103	-5.13469536	0.00000000

Es
BLYP-D3(BJ)/TZ2P (-3163.85)

1	H	-0.33984893	7.08684406	0.00000000
2	H	3.18790288	1.63674602	0.00000000
3	H	2.08674476	6.30539801	0.00000000
4	C	-2.58916710	2.02342012	0.00000000
5	C	-3.51885866	0.94954422	0.00000000
6	C	-2.89978630	-0.28188839	0.00000000
7	S	-1.17349211	-0.15173266	0.00000000
8	C	-1.26653236	1.60465330	0.00000000
9	C	-0.83652707	4.91115118	0.00000000
10	H	-2.88516412	3.06456434	0.00000000
11	H	-4.59665669	1.08015584	0.00000000
12	H	-3.36586178	-1.25970545	0.00000000
13	N	1.11860084	1.63749070	0.00000000
14	C	2.30253300	2.27131194	0.00000000
15	N	2.51899685	3.59939094	0.00000000
16	C	1.38445214	4.30582088	0.00000000
17	C	0.05288340	3.77998088	0.00000000
18	C	-0.02774841	2.36464550	0.00000000
19	N	1.29020593	5.68059024	0.00000000
20	C	-0.05247005	6.04428648	0.00000000
21	H	-1.91605355	4.91323391	0.00000000

M06-2X/TZ2P (-4584.90)

1	H	-0.34193720	7.04650547	0.00000000
2	H	3.16115979	1.65537507	0.00000000
3	H	2.06355146	6.26731350	0.00000000
4	C	-2.56308334	2.03170672	0.00000000
5	C	-3.49441442	0.96536070	0.00000000
6	C	-2.87852898	-0.25045310	0.00000000
7	S	-1.17978926	-0.11866589	0.00000000
8	C	-1.26277790	1.60302751	0.00000000
9	C	-0.83878865	4.89637543	0.00000000
10	H	-2.85005858	3.06952922	0.00000000
11	H	-4.56539455	1.09612912	0.00000000
12	H	-3.34254524	-1.22283729	0.00000000
13	N	1.10739609	1.65437347	0.00000000

14	C	2.28067710	2.28571991	0.00000000
15	N	2.48724320	3.59238976	0.00000000
16	C	1.36028682	4.29106848	0.00000000
17	C	0.04822744	3.76919326	0.00000000
18	C	-0.01950700	2.36725359	0.00000000
19	N	1.27088140	5.64974651	0.00000000
20	C	-0.05632109	6.00910530	0.00000000
21	H	-1.91212440	4.90768534	0.00000000

wB97X-D/TZ2P (-5309.53)

1	H	-0.34709790	7.04414389	0.00000000
2	H	3.16047127	1.65410152	0.00000000
3	H	2.05949110	6.26547803	0.00000000
4	C	-2.55895526	2.03300982	0.00000000
5	C	-3.49137857	0.96724101	0.00000000
6	C	-2.87606899	-0.24600243	0.00000000
7	S	-1.18191615	-0.11411487	0.00000000
8	C	-1.26243608	1.60197316	0.00000000
9	C	-0.83813270	4.89492908	0.00000000
10	H	-2.84563518	3.07233375	0.00000000
11	H	-4.56356677	1.09911734	0.00000000
12	H	-3.34246721	-1.21872056	0.00000000
13	N	1.10651974	1.65503803	0.00000000
14	C	2.27932941	2.28618779	0.00000000
15	N	2.48560944	3.59027914	0.00000000
16	C	1.35895659	4.29070655	0.00000000
17	C	0.05248339	3.76698003	0.00000000
18	C	-0.01753889	2.36884218	0.00000000
19	N	1.26918424	5.64754292	0.00000000
20	C	-0.05942945	6.00573913	0.00000000
21	H	-1.91326934	4.90109656	0.00000000

B3LYP-D/TZ2P (-3789.14)

1	H	-0.34163414	7.05751589	0.00000000
2	H	3.16352953	1.64776311	0.00000000
3	H	2.06834007	6.27921717	0.00000000
4	C	-2.57131876	2.02868759	0.00000000
5	C	-3.49903584	0.96099557	0.00000000
6	C	-2.88356999	-0.25982371	0.00000000
7	S	-1.17418447	-0.12898224	0.00000000
8	C	-1.26203073	1.60791146	0.00000000
9	C	-0.83527885	4.90025241	0.00000000
10	H	-2.86406842	3.06463178	0.00000000
11	H	-4.57061303	1.09226867	0.00000000
12	H	-3.34887072	-1.23165980	0.00000000
13	N	1.10813934	1.65182430	0.00000000
14	C	2.28339328	2.27909240	0.00000000
15	N	2.49374881	3.59281619	0.00000000
16	C	1.36952836	4.29662118	0.00000000
17	C	0.05071394	3.77322941	0.00000000
18	C	-0.02645501	2.36889099	0.00000000
19	N	1.27738254	5.65930700	0.00000000
20	C	-0.05467303	6.02063220	0.00000000
21	H	-1.90889020	4.90471052	0.00000000

BLYP-D3(BJ)/QZ4P (-3174.62)

1	H	-0.33683819	7.08798523	0.00000000
2	H	3.18609331	1.63626886	0.00000000
3	H	2.08734605	6.30428200	0.00000000
4	C	-2.59138373	2.02319799	0.00000000

5	C	-3.51981575	0.94786838	0.00000000
6	C	-2.89816300	-0.28321114	0.00000000
7	S	-1.17424083	-0.14882019	0.00000000
8	C	-1.26779642	1.60544943	0.00000000
9	C	-0.83650732	4.91274595	0.00000000
10	H	-2.88837306	3.06334295	0.00000000
11	H	-4.59675165	1.07729361	0.00000000
12	H	-3.36230835	-1.26077729	0.00000000
13	N	1.11755288	1.63720137	0.00000000
14	C	2.30208055	2.27070865	0.00000000
15	N	2.51984020	3.59908037	0.00000000
16	C	1.38479177	4.30568591	0.00000000
17	C	0.05267579	3.78086813	0.00000000
18	C	-0.02876642	2.36554650	0.00000000
19	N	1.29138106	5.68078808	0.00000000
20	C	-0.05147967	6.04584085	0.00000000
21	H	-1.91518455	4.91455646	0.00000000

TABLE S14. Base-pair binding energies (ΔE, in kcal mol^{-1}) of Ds-Px and Es-Px computed at different levels of theory in the gas phase.

**Ds-Px**	ΔE
BLYP-D3(BJ)/TZ2P	-3.9
M06-2X/TZ2P	-1.8
wB97X-D/TZ2P	-6.1
B3LYP-D/TZ2P	-3.9
BLYP-D3(BJ)/QZ4P	-3.6

**Es-Px**	ΔE
BLYP-D3(BJ)/TZ2P	-5.6
M06-2X/TZ2P	-3.5
wB97X-D/TZ2P	-8.3
B3LYP-D/TZ2P	-5.6
BLYP-D3(BJ)/QZ4P	-5.3

S13. Imaginary frequencies of UBPs and their bases constrained in C_s symmetry

TABLE S15. Number of imaginary frequencies, their values (in cm^{-1}), and the maximum intensity for the series of UBPs under analysis. Computed at ZORA-BLYP-D3(BJ)/TZ2P in gas phase.

UBP	N. Imag. Freq.	Imag. Freq. (in cm^{-1})	Max. Intensity (km/mol)
Ds–Px	2	-28.56, -6.8	1.4316
Bs–Px	2	-24.2, -2.9	2.5870
Is–Px	2	-55.3, -23.5	2.5817
Ys–Px	3	-46.0, -25.3, -9.0	1.8662
Yi–Px	3	-37.5, -10.0, -6.0	2.8206
Do–Px	0		
Dp–Px	1	-6.0	2.3318
Dss–Px	2	-14.4, -13.5	3.0705
Dt–Px	3	-32.9, -25.6, -8.7	2.0220
Yo–Px	0		
Ps–Px	1	-16.3	0.4798
Es–Px	1	-19.5	0.1247
Ysm–Px	3	-42.1, -18.0, -11.8	2.1723
Yss–Px	4	-41.5, -26.2 -14.5, -12.0	3.2294

TABLE S16. Number of imaginary frequencies, their values (in cm^{-1}), and the maximum intensity, for the series of bases under analysis. Computed at ZORA-BLYP-D3(BJ)/TZ2P in gas phase.

Base	N. Imag. Freq.	Imag. Freq. (in cm^{-1})	Max. Intensity (km/mol)
Ds	0		
Bs	0		
Is	1	-56.9	0.0245
Ys	1	-45.8	0.0005
Yi	0		
Do	0		
Dp	0		
Dss	1	-4.0	0.2633
Dt	1	-29.0	0.5525
Yo	0		
Ps	0		
Es	0		
Ysm	2	-102.4, -41.3	0.0150
Yss	2	-42.0, -9.5	0.4756
Px	0		
